# Supplementary material for: Photocatalytic defluoroalkylation and hydrodefluorination of trifluoromethyls using o-phosphinophenolate
Source: Nat Commun. 2022 Jan 17;13:354. doi: 10.1038/s41467-022-28007-2 (PMC8764036; doi:10.1038/s41467-022-28007-2)
Supplement: Supplementary file 1 — Supplementary Information [file 41467_2022_28007_MOESM1_ESM.pdf]

Supplementary Information

Photocatalytic Defluoroalkylation and Hydrodefluorination of Trifluoromethyls using *o*-Phosphinophenolate

Can Liu,[1] Ni Shen,[1] Rui Shang,*[1,2]

[1]Department of Chemistry, University of Science and Technology of China, Hefei 230026, China; [2]Department of Chemistry, School of Science, The University of Tokyo, 7-3-1 Hongo, Bunkyo-ku, Tokyo 113-0033, Japan.

rui@chem.s.u-tokyo.ac.jp

Table of Contents

1. Supplementary Methods

1-1 General Information S3

1-2 Preparation of Catalysts S4

1-3 Preparation of Substrates S5

2. Supplementary Discussion

2-1 Investigation of the Key Reaction Parameters S6

2-2 Experimental Procedures and Spectral Data S9

2-3 Experimental Studies on Mechanism S36

2-4 ^{1}H-NMR, ^{13}C-NMR, and ^{19}F-NMR Spectra S44

3. Supplementary References S128

1. Supplementary Methods

1-1 General Information

A. Materials:

All reactions were carried out in oven-dried Schlenk tubes under argon atmosphere (purity≥99.999%) unless otherwise mentioned. Commercial reagents were purchased from Adamas-beta, TCI and Aldrich. Organic solutions were concentrated under reduced pressure on Buchi rotary evaporator. The LED lamps were purchased from Kessil (PR160-427 nm, 440 nm, 456 nm, 467 nm). The Photo Reaction Setup was purchased from HepatoChem.

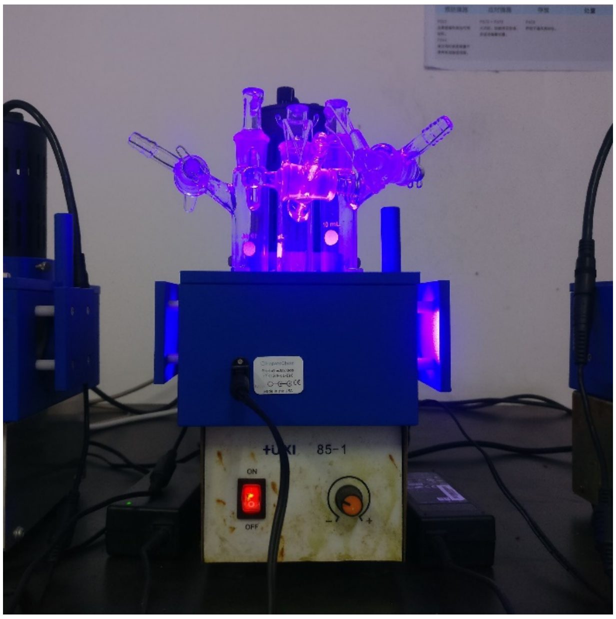

Supplementary Figure 1. The Photo Reaction Setup and violet LED lamps

B. Analytical Methods:

^{1}H-NMR, ^{19}F-NMR and ^{13}C-NMR spectra were recorded on a Bruker Avance 400 spectrometer at ambient temperature. Data for ^{1}H-NMR are reported as follows: chemical shift (ppm, scale), multiplicity (s = singlet, d = doublet, t = triplet, q = quartet, m = multiplet and/or multiplet resonances, br = broad), coupling constant (Hz), and integration. Data for ^{13}C- NMR are reported in terms of chemical shift (ppm, scale), multiplicity, and coupling constant (Hz). HRMS analysis was performed on Finnigan LCQ advantage Max Series MS System. ESI-mass data were acquired using

a Thermo LTQ Orbitrap XL Instrument equipped with an ESI source and controlled by Xcalibur software. UV-Vis spectrum was measured by UV-3600. Flash column chromatographic purification of products was accomplished using forced-flow chromatography on Silica Gel (200-300 mesh).

1-2 Preparation of Catalysts

1-2-1 General Procedure for preparation of catalyst

PO catalysts **PO1**, **PO3**, **PO4**, **PO5**, **PO6**, **PO7** were known compounds and prepared according to the literature procedures.[1-6]

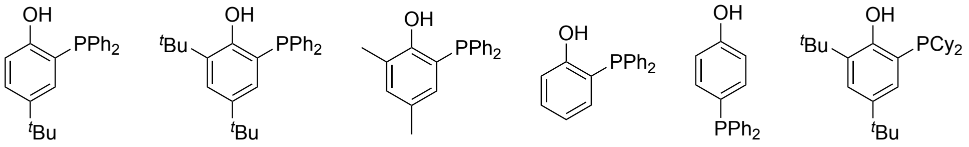

PO 1 **PO 3** **PO 4** **PO 5** **PO 6** **PO 7**

Other **PO** catalysts used in this work were prepared by the following method：

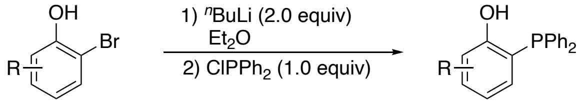

The Catalyst *o*-Phosphinophenol (PO) was prepared according to a previous published protocol.[7] *n*-Butyl lithium (1.6 M in hexane, 20 mmol) was added into a solution of *o*-bromophenol (10 mmol) in Et_2O (22 mL) at -78 °C, giving immediately a white suspension. The cold bath was removed and stirring was continued at room temperature for 30 mins. Chlorodiphenylphosphine (10 mmol) was added into this solution at -78 °C. After stirring for 3 h, the cold bath was removed and stirring was continued at 0 °C for 18 h. The mixture was extracted with aqueous solutions of NaH_2PO_4 (0.1 M), and the combined organic layers were washed with brine, dried over Na_2SO_4, filtered and concentrated in vacuo. The crude residue was purified by flash column chromatography on silica gel to give desired product.

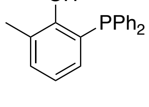

2-(diphenylphosphaneyl)-6-methylphenol (PO2): Following the general procedure, obtained in 75% yield as white solid after silica gel chromatography. (2.19 g, eluent: petroleum ether/ethyl acetate = 10/1).

Mp = 143 – 145 °C

^{1}H NMR (400 MHz, $CDCl_3$) δ 7.42 – 7.27 (m, 10H), 7.16 (dd, J = 7.1, 1.1 Hz, 1H), 6.94 – 6.71 (m, 2H), 6.41 (d, J = 7.9 Hz, 1H), 2.26 (s, 3H).

^{13}C NMR (101 MHz, $CDCl_3$) δ 157.6 (d, J = 18.8 Hz), 135.1 (d, J = 3.8 Hz), 133.4 (d, J = 18.6 Hz), 132.9, 132.33 (d, J = 3.4 Hz), 129.0, 128.7 (d, J = 7.3 Hz), 124.5 (d, J = 1.8 Hz), 120.7 (d, J = 2.9 Hz), 120.0 (d, J = 3.0 Hz), 16.3 (d, J = 2.6 Hz).

^{31}P NMR (162 MHz, $CDCl_3$) δ -29.9.

HRMS (ESI) *m/z*: $[M+H]^+$ Calcd. for $C_{19}H_{18}OP$, 293.1090; found, 293.1092.

1-3 Preparation of Substrates

1-3-1 General Procedure for preparation of trifluoroacetamides and pentafluoropropionamides

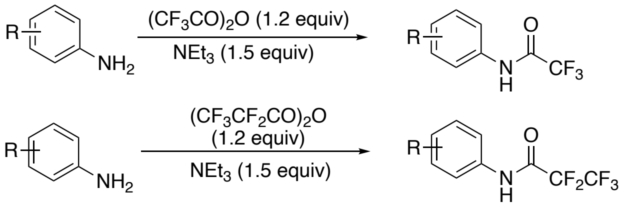

General Procedure 1: Trifluoroacetamides and Pentafluoropanamides.[8] To a solution of aniline and Et_3N (1.5 equiv) in CH_2Cl_2 was added trifluoroacetic anhydride (1.2 equiv) dropwise at 0 °C. After addition, the mixture was stirred at room temperature until TLC to indicate aniline disappeared. The reaction was then quenched with H_2O and extracted three times with CH_2Cl_2. The combined organic layers were washed with brine, dried over Na_2SO_4, filtered and concentrated in vacuo. The crude residue was purified by flash column chromatography on silica gel to give desired products.

1-3-2 General Procedure for preparation of trifluoroacetates

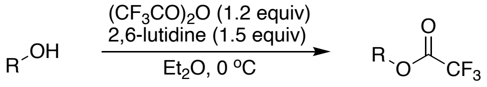

General Procedure 2: Trifluoroacetates.[8] To a solution of alcohol and 2,6-lutidine (1.5 equiv) in Et_2O was added trifluoroacetic anhydride (1.3 equiv) dropwise at 0 °C. After addition, the mixture was stirred at 0 °C until TLC indicated that alcohol disappeared. The reaction was then poured into H_2O and extracted with 1N HCl (×2), saturated aqueous $NaHCO_3$, brine, dried over Na_2SO_4, filtered, and concentrated in vacuo. The crude residue was purified by flash column chromatography on silica gel to give desired products.

2. Supplementary Discussion

2-1 Investigation of the Key Reaction Parameters

Supplementary Table 1: Parameters affecting defluoroalkylation of trifluoroacetamides

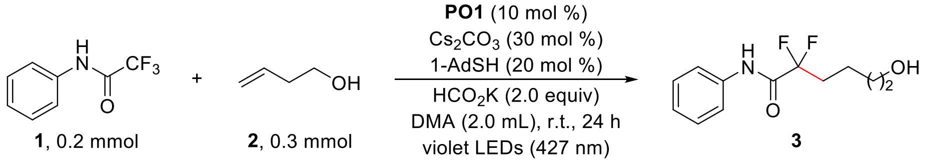

Entry	Variations from standard conditions	Yield (%)[a]
1	none	90
2	DMF instead of DMA	89
3	DMSO instead of DMA	91
4	NMP instead of DMA	80
5	MeCN instead of DMA	23
6	THF instead of DMA	4
7	Acetone instead of DMA	7
8	1,4-Dioxane instead of DMA	0
9	Toluene instead of DMA	0
10	DCM instead of DMA	0
11	**PO2** instead of **PO1**	90
12	**PO3** instead of **PO1**	87
13	**PO4** instead of **PO1**	93
14	**PO5** instead of **PO1**	65

15	**PO6** instead of **PO1**	15
16	**PO7** instead of **PO1**	< 5
17	CySH instead of 1-Adamantanethiol	75
18	DABCO instead of 1-Adamantanethiol	0
19	Triethylsilane instead of HCO_2K	30
20	HCO_2Na instead of HCO_2K	82
21	HCO_2Li instead of HCO_2K	92
22	HCO_2Cs instead of HCO_2K	70
23	**2** (0.4 mmol) instead of **2** (0.3 mmol)	96
24	**PO1** (2 mol%) instead of **PO1** (10 mol%)	52
25	**PO1** (5 mol%) instead of **PO1** (10 mol%)	67
26	1-AdSH (5 mol%) instead of 1-AdSH (20 mol%)	59
27	1-AdSH (10 mol%) instead of 1-AdSH (20 mol%)	72
28	440 nm instead of 427 nm	70
29	456 nm instead of 427 nm	trace
30	467 nm instead of 427 nm	trace
31	PPh_3 instead of **PO1**	trace
32	4-*t*-Bu-C_6H_4-OH instead of **PO1**	0
33	PPh_3 + 4-*t*-Bu-C_6H_4-OH (1:1, 10 mol%) instead of **PO1** (10 mol%)	10
34	*w/o* **PO1**	0
35	*w/o* 1-AdSH	trace
36	*w/o* HCO_2K	18
37	*w/o* light	0
38	Under air	trace

[a]Yield determined by ^{1}H-NMR using diphenylmethane as an internal standard.

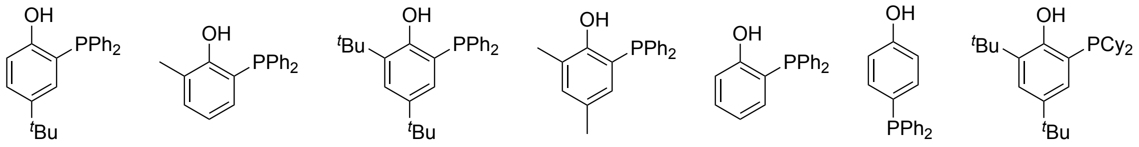

PO 1 **PO 2** **PO 3** **PO 4** **PO 5** **PO 6** **PO 7**

Supplementary Table 2: Parameters affecting hydrodefluorination of trifluoroacetamides

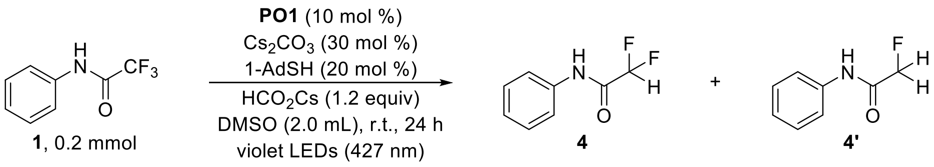

Entry	Variations from standard conditions	Yield 4 (%)[a]	Yield 4'(%)[a]
1	none	94	< 5

2	HCO_2Cs (2.0 equiv) instead of HCO_2Cs (1.2 equiv)	79	17
3	HCO_2Cs (3.0 equiv) instead of HCO_2Cs (1.2 equiv)	60	38
4	DMA instead of DMSO	65	trace
5	DMF instead of DMSO	50	trace
6	*w/o* 1-AdSH	12	n.d.
7	*w/o* HCO_2Cs	10	n.d.

[a]Yield determined by ^{1}H-NMR using diphenylmethane as internal standard.

2-2 Experimental Procedures and Spectral Data

2-2-1 General Procedure

General Procedure A: trifluoroacetamide (1.0 equiv, 0.2 mmol) (if solid), Alkene (1.5 equiv, 0.3 mmol) (if solid), **PO1** (10 mol%), Cs_2CO_3 (30 mol%), 1-adamananethiol (20 mol%), HCO_2K (2.0 equiv, 0.4 mmol) were placed in a transparent Schlenk tube equipped with a stirring bar. The tube was evacuated and filled with argon (three times). To these solids, trifluoroacetamide (1.0 equiv, 0.2 mmol) (if liquid), alkene (1.5 equiv, 0.3 mmol) (if liquid), and anhydrous DMA (2 mL) were added via a gastight syringe under argon atmosphere. The reaction mixture was stirred under irradiation with violet LEDs (427 nm) in a photoreactor at room temperature for 24 h. The mixture was quenched with brine and extracted with ethyl acetate (3 × 10 mL). The organic layers were combined and concentrated under vacuo. The product was purified by flash column chromatography on silica gel.

General Procedure B: trifluoacatete (2.0 equiv, 0.4 mmol) (if solid), alkene (1.0 equiv, 0.2 mmol) (if solid), **PO1** (10 mol%), Cs_2CO_3 (30 mol%), 1-adamananethiol (20 mol%), HCO_2K (2.0 equiv, 0.4 mmol) were placed in a transparent Schlenk tube equipped with a stirring bar. The tube was evacuated and filled with argon (three times). To these solids, trifluoacetate (2.0 equiv, 0.4 mmol) (if liquid), alkene (1.0 equiv, 0.2 mmol) (if liquid), and anhydrous DMA (2 mL) were added via a gastight syringe under argon atmosphere. The reaction mixture was stirred under irradiation with violet LEDs (427 nm) in a photoreactor at room temperature for 24 h. The mixture was quenched with brine and extracted with ethyl acetate (3 × 10 mL). The organic layers were combined and concentrated under vacuo. The product was purified by flash column chromatography on silica.

General Procedure C: trifluoroacetamide (1.0 equiv, 0.2 mmol) (if solid), **PO1** (10 mol%), Cs_2CO_3 (30 mol%), 1-adamananethiol (20 mol%), HCO_2Cs (1.2 equiv, 0.24 mmol) were placed in a transparent Schlenk tube equipped with a stirring bar. The tube was evacuated and filled with argon (three times). To these solids,

trifluoroacetamide (1.0 equiv, 0.2 mmol) (if liquid) and anhydrous DMSO (2 mL) were added via a gastight syringe under argon atmosphere. The reaction mixture was stirred under irradiation with violet LEDs (427 nm) in a photoreactor at room temperature for 24 h. The mixture was quenched with brine and extracted with ethyl acetate (3 × 10 mL). The organic layers were combined and concentrated under vacuo. The product was purified by flash column chromatography on silica.

General Procedure D: trifluoromethyl (hetero) arenes (1.0 equiv, 0.2 mmol) (if solid), alkene (1.5 equiv, 0.3 mmol) (if solid), **PO1** (10 mol%), Cs_2CO_3 (30 mol%), 1-adamananethiol (20 mol%), HCO_2Li (2.0 equiv, 0.4 mmol) were placed in a transparent Schlenk tube equipped with a stirring bar. The tube was evacuated and filled with argon (three times). To these solids, trifluomethyl (hetero) arenes (1.0 equiv, 0.2 mmol) (if liquid), alkene (1.5 equiv, 0.3 mmol) (if liquid), and anhydrous DMSO (2 mL) were added via a gastight syringe under argon atmosphere. The reaction mixture was stirred under irradiation with violet LEDs (427 nm) in a photoreactor at room temperature for 24 h. The mixture was quenched with brine and extracted with ethyl acetate (3 × 10 mL). The organic layers were combined and concentrated under vacuo. The product was purified by flash column chromatography on silica.

2-2-2 Spectral Data

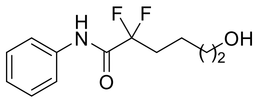

2,2-difluoro-6-hydroxy-*N*-phenylhexanamide (3): Following the general procedure A, obtained in 86% yield as white solid after silica gel chromatography. (41.9 mg, eluent: petroleum ether/ethyl acetate = 2/1).

Mp = 61 – 63 °C

^{1}H NMR (400 MHz, $CDCl_3$) δ 8.09 (s, 1H), 7.57 (d, *J* = 7.9 Hz, 2H), 7.37 (t, *J* = 7.9 Hz, 2H), 7.19 (t, *J* = 7.4 Hz, 1H), 3.66 (t, *J* = 5.7 Hz, 2H), 2.32 – 2.12 (m, 2H), 1.73 (s, 1H), 1.62 (dd, *J* = 7.0, 4.0 Hz, 4H).

^{13}C NMR (101 MHz, $CDCl_3$) δ 162.2 (t, *J* = 28.6 Hz), 136.0, 129.2, 125.6, 120.3, 118.3 (t, *J* = 253.6 Hz), 62.2, 33.5 (t, *J* = 23.2 Hz), 31.9, 18.1 (t, *J* = 4.5 Hz).

^{19}F NMR (376 MHz, $CDCl_3$) δ -105.5 (2F, td, *J* = 17.3, 2.9 Hz).

HRMS (ESI) *m/z*: $[M+Na]^+$ Calcd. for $C_{12}H_{15}F_2NO_2Na$, 266.0963; found, 266.0962.

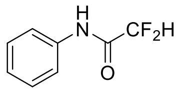

2,2-difluoro-*N*-phenylacetamide (4): Following the general procedure C, obtained in 94% yield as white solid after silica gel chromatography. (32.1 mg, eluent: petroleum ether/ethyl acetate = 15/1). The compound data was in agreement with the literature (*Science* **2021**, *371*, 1232–1240).

^{1}H NMR (400 MHz, $CDCl_3$) δ 7.92 (s, 1H), 7.57 (d, *J* = 8.0 Hz, 2H), 7.38 (t, *J* = 7.9 Hz, 2H), 7.21 (t, *J* = 7.4 Hz, 1H), 6.02 (t, *J* = 54.4 Hz, 1H).

^{13}C NMR (101 MHz, $CDCl_3$) δ 160.3 (t, *J* = 24.1 Hz), 135.7, 129.3, 125.9, 120.3, 108.6 (t, *J* = 254.2 Hz).

^{19}F NMR (376 MHz, $CDCl_3$) δ -125.5 (2F, dd, *J* = 54.4, 2.3 Hz).

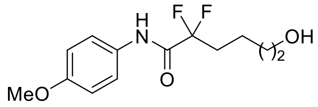

2,2-difluoro-6-hydroxy-*N*-(4-methoxyphenyl)hexanamide (5): Following the general procedure A, obtained in 80% yield as white solid after silica gel chromatography. (43.5 mg, eluent: petroleum ether/ethyl acetate = 2/1).

Mp = 90 – 92 °C

^{1}H NMR (400 MHz, *d*$_6$-DMSO) δ 10.38 (s, 1H), 7.59 (d, *J* = 9.1 Hz, 2H), 6.93 (d, *J* = 9.1 Hz, 2H), 3.74 (s, 3H), 3.41 (t, *J* = 5.7 Hz, 2H), 2.25 – 2.04 (m, 2H), 1.52 – 1.42 (m, 4H).

^{13}C NMR (101 MHz, *d*$_6$-DMSO) δ 162.3 (t, *J* = 29.4 Hz), 156.8, 130.7, 122.9, 118.7 (t, *J* = 251.5 Hz), 114.3, 60.7, 55.7, 34.1 (t, *J* = 23.3 Hz), 32.2, 18.6 (t, *J* = 4.3 Hz).

^{19}F NMR (376 MHz, *d*$_6$-DMSO) δ -104.1 (2F, t, *J* = 17.3 Hz).

HRMS (ESI) *m/z*: [M+Na]$^+$ Calcd. for $C_{13}H_{17}F_2NO_3Na$, 296.1069; found, 296.1072.

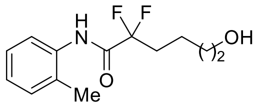

2,2-difluoro-6-hydroxy-*N*-(o-tolyl)hexanamide (6): Following the general procedure A, obtained in 70% yield as colorless oil after silica gel chromatography. (36.0 mg, eluent: petroleum ether/ethyl acetate = 2/1).

^{1}H NMR (400 MHz, CDCl$_3$) δ 7.91 (s, 1H), 7.83 (d, *J* = 7.9 Hz, 1H), 7.23 (dd, *J* = 7.5, 3.6 Hz, 2H), 7.15 (t, *J* = 7.1 Hz, 1H), 3.67 (t, *J* = 5.7 Hz, 2H), 2.29 (s, 3H), 2.27 – 2.14 (m, 2H), 1.71 (s, 1H), 1.68 – 1.59 (m, 4H).

^{13}C NMR (101 MHz, CDCl$_3$) δ 162.2 (t, *J* = 28.6 Hz), 133.8, 130.7, 129.4, 127.0, 126.3, 122.9, 118.5 (t, *J* = 253.4 Hz), 62.2, 33.5 (t, *J* = 23.3 Hz), 31.9, 18.1 (t, *J* = 4.5 Hz), 17.5.

^{19}F NMR (376 MHz, CDCl$_3$) δ -105.4 (2F, td, *J* = 17.2, 3.2 Hz).

HRMS (ESI) *m/z*: [M+Na]$^+$ Calcd. for $C_{13}H_{17}F_2NO_2Na$, 280.1120; found, 280.1124.

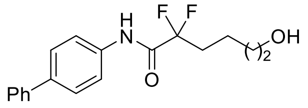

***N*-([1,1'-biphenyl]-4-yl)-2,2-difluoro-6-hydroxyhexanamide (7):** Following the general procedure A, obtained in 85% yield as white solid after silica gel chromatography. (54.3 mg, eluent: petroleum ether/ethyl acetate = 2/1).

Mp = 162 – 164 °C

^{1}H NMR (400 MHz, Acetone-d_6) δ 9.79 (s, 1H), 7.88 (d, *J* = 8.3 Hz, 2H), 7.83 – 7.61 (m, 4H), 7.46 (dd, *J* = 7.9, 7.4 Hz, 2H), 7.42 – 7.22 (m, 1H), 3.58 (s, 2H), 2.36 – 2.11 (m, 2H), 1.71 – 1.48 (m, 4H).

^{13}C NMR (101 MHz, Acetone-d_6) δ 162.2 (t, *J* = 28.6 Hz), 140.2, 137.5, 136.9, 128.9, 127.2, 127.2, 126.6, 120.9, 118.6 (t, *J* = 272.0 Hz), 61.0, 33.8 (t, *J* = 23.4 Hz), 32.2, 18.3 (t, *J* = 4.6 Hz).

^{19}F NMR (376 MHz, Acetone-d_6) δ -106.1 (2F, td, *J* = 17.1, 1.4 Hz).

HRMS (ESI) *m/z*: [M+Na]$^+$ Calcd. for $C_{18}H_{19}F_2NO_2Na$, 342.1276; found, 342.1277.

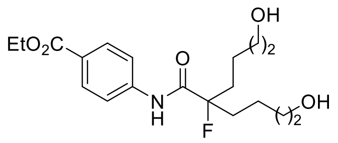

ethyl 4-(2-fluoro-6-hydroxy-2-(4-hydroxybutyl)hexanamido)benzoate (8): Following the general procedure A, obtained in 63% yield as white solid after silica gel chromatography. (46.5 mg, eluent: petroleum ether/ethyl acetate = 1/1).

Mp = 90 – 92 °C

^{1}H NMR (400 MHz, $CDCl_3$) δ 8.41 (d, *J* = 8.6 Hz, 1H), 8.02 (d, *J* = 8.7 Hz, 2H), 7.67 (d, *J* = 8.7 Hz, 2H), 4.36 (q, *J* = 7.1 Hz, 2H), 3.60 (t, *J* = 5.8 Hz, 4H), 2.15 – 1.96 (m, 4H), 1.94 – 1.72 (m, 2H), 1.69 – 1.49 (m, 6H), 1.39 (t, *J* = 7.1 Hz, 3H).

^{13}C NMR (101 MHz, $CDCl_3$) δ 170.6 (d, *J* = 20.4 Hz), 166.1, 140.8, 130.8, 126.6, 119.2, 101.1 (d, *J* = 188.8 Hz), 62.2, 61.0, 36.9 (d, *J* = 22.0 Hz), 32.4, 19.5 (d, *J* = 3.1 Hz), 14.3.

^{19}F NMR (376 MHz, $CDCl_3$) δ -164.3 – -164.6 (1F, m).

HRMS (ESI) *m/z*: [M+Na]$^+$ Calcd. for $C_{19}H_{28}FNO_5Na$, 392.1844; found, 392.1843.

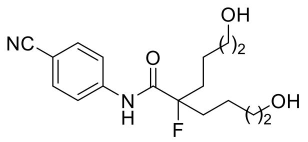

***N*-(4-cyanophenyl)-2-fluoro-6-hydroxy-2-(4-hydroxybutyl)hexanamide (9):** Following the general procedure A, obtained in 50% yield as colorless oil after silica gel chromatography. (32.2 mg, eluent: petroleum ether/ethyl acetate = 1/1).

^{1}H NMR (400 MHz, $CDCl_3$) δ 8.32 (d, *J* = 9.0 Hz, 1H), 7.73 (d, *J* = 8.9 Hz, 2H), 7.65 (d, *J* = 8.8 Hz, 2H), 3.79 – 3.54 (m, 4H), 2.16 – 1.96 (m, 3H), 1.96 – 1.77 (m, 3H), 1.62 – 1.53 (m, 6H).

^{13}C NMR (101 MHz, $CDCl_3$) δ 170.7 (d, *J* = 20.4 Hz), 140.7, 133.4, 119.8, 118.6, 107.9, 101.2 (d, *J* = 188.7 Hz), 62.4, 36.9 (d, *J* = 22.1 Hz), 32.4, 19.5 (d, *J* = 3.2 Hz).

^{19}F NMR (376 MHz, $CDCl_3$) δ -164.0 – -164.8 (1F, m).

HRMS (ESI) *m/z*: $[M+H]^+$ Calcd. for $C_{17}H_{24}FN_2O_3$, 323.1765; found, 323.1763.

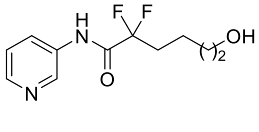

2,2-difluoro-6-hydroxy-*N*-(pyridin-3-yl)hexanamide (10): Following the general procedure A, obtained in 68% yield as colorless oil after silica gel chromatography. (33.2 mg, eluent: petroleum ether/ethyl acetate = 2/1).

^{1}H NMR (400 MHz, $CDCl_3$) δ 8.69 (s, 1H), 8.47 (s, 1H), 8.43 (d, *J* = 4.4 Hz, 1H), 8.19 (d, *J* = 8.4 Hz, 1H), 7.34 (dd, *J* = 8.4, 4.7 Hz, 1H), 3.68 (t, *J* = 5.7 Hz, 2H), 2.31 – 2.15 (m, 2H), 1.95 (s, 1H), 1.68 – 1.60 (m, 4H).

^{13}C NMR (101 MHz, $CDCl_3$) δ 162.8 (t, *J* = 29.5 Hz), 146.4, 141.6, 133.3, 127.9, 123.9, 118.1 (t, *J* = 253.4 Hz), 62.1, 33.5 (t, *J* = 23.1 Hz), 31.9, 18.1 (t, *J* = 4.4 Hz).

^{19}F NMR (376 MHz, $CDCl_3$) δ -105.2 (2F, td, *J* = 17.3, 2.5 Hz).

HRMS (ESI) *m/z*: $[M+H]^+$ Calcd. for $C_{11}H_{15}F_2N_2O_2$, 245.1096; found, 245.1104.

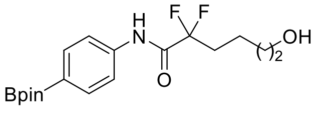

2,2-difluoro-6-hydroxy-*N*-(4-(4,4,5,5-tetramethyl-1,3,2-dioxaborolan-2-yl)phenyl) hexanamide (11): Following the general procedure A, obtained in 88% yield as colorless oil after silica gel chromatography. (64.9 mg, eluent: petroleum ether/ethyl acetate = 2/1).

^{1}H NMR (400 MHz, $CDCl_3$) δ 8.08 (s, 1H), 7.81 (d, *J* = 8.5 Hz, 2H), 7.59 (d, *J* = 8.5 Hz, 2H), 3.67 (t, *J* = 5.9 Hz, 2H), 2.35 – 2.11 (m, 2H), 1.74 (s, 1H), 1.67 – 1.60 (m, 4H), 1.34 (s, 12H).

^{13}C NMR (101 MHz, $CDCl_3$) δ 162.1 (t, *J* = 28.7 Hz), 138.6, 135.9, 119.1, 118.2 (t, *J* = 253.8 Hz), 83.9, 62.2, 33.4 (t, *J* = 23.1 Hz), 31.9, 24.9, 18.1 (t, *J* = 4.4 Hz).

^{19}F NMR (376 MHz, CDCl$_3$) δ -105.4 (2F, td, *J* = 17.3, 3.0 Hz).

HRMS (ESI) *m/z*: [M+Na]$^+$ Calcd. for $C_{18}H_{26}BF_2NO_4Na$, 392.1815; found, 392.1818.

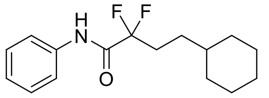

4-cyclohexyl-2,2-difluoro-*N*-phenylbutanamide (15): Following the general procedure A, obtained in 84% yield as white solid after silica gel chromatography. (47.0 mg, eluent: petroleum ether/ethyl acetate = 30/1).

Mp = 70 – 72 °C

^{1}H NMR (400 MHz, CDCl$_3$) δ 7.98 (s, 1H), 7.58 (d, *J* = 7.6 Hz, 2H), 7.37 (t, *J* = 7.9 Hz, 2H), 7.19 (t, *J* = 7.4 Hz, 1H), 2.35 – 2.05 (m, 2H), 1.68 (t, *J* = 17.5 Hz, 5H), 1.46 – 1.33 (m, 2H), 1.31 – 1.08 (m, 4H), 0.90 (dd, *J* = 21.8, 11.0 Hz, 2H).

^{13}C NMR (101 MHz, CDCl$_3$) δ 162.2 (t, *J* = 28.9 Hz), 136.1, 129.2, 125.5, 120.2, 118.7 (t, *J* = 253.4 Hz), 37.2, 33.0, 31.4 (t, *J* = 23.1 Hz), 28.8 (t, *J* = 3.8 Hz), 26.5, 26.2.

^{19}F NMR (376 MHz, CDCl$_3$) δ -105.6 (2F, td, *J* = 17.4, 2.6 Hz).

HRMS (ESI) *m/z*: [M+Na]$^+$ Calcd. for $C_{16}H_{21}F_2NONa$, 304.1483; found, 304.1490.

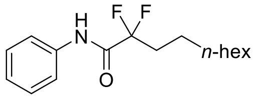

2,2-difluoro-*N*-phenyldecanamide (16): Following the general procedure A, obtained in 83% yield as white solid after silica gel chromatography. (46.1 mg, eluent: petroleum ether/ethyl acetate = 30/1).

Mp = 45 – 47 °C

^{1}H NMR (400 MHz, CDCl$_3$) δ 7.97 (s, 1H), 7.58 (d, *J* = 8.0 Hz, 2H), 7.37 (t, *J* = 7.9 Hz, 2H), 7.19 (t, *J* = 7.4 Hz, 1H), 2.28 – 2.04 (m, 2H), 1.57 – 1.46 (m, 2H), 1.39 – 1.21 (m, 10H), 0.87 (t, *J* = 6.7 Hz, 3H).

^{13}C NMR (101 MHz, CDCl$_3$) δ 162.1 (t, *J* = 28.5 Hz), 136.1, 129.2, 125.5, 120.2, 118.5 (t, *J* = 253.3 Hz), 33.8 (t, *J* = 23.1 Hz), 31.8, 29.2, 29.14, 29.07, 22.6, 21.6 (t, *J* = 4.3 Hz), 14.1.

^{19}F NMR (376 MHz, CDCl$_3$) δ -105.5 (2F, td, *J* = 17.5, 3.0 Hz).

HRMS (ESI) *m/z*: [M+H]$^+$ Calcd. for $C_{16}H_{24}F_2NO$, 284.1820; found, 284.1829.

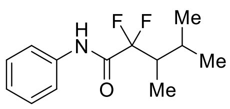

2,2-difluoro-3,4-dimethyl-*N*-phenylpentanamide (17): Following the general procedure A, obtained in 85% yield as colorless oil after silica gel chromatography. (41.0 mg, eluent: petroleum ether/ethyl acetate = 30/1).

^{1}H NMR (400 MHz, $CDCl_3$) δ 8.07 (s, 1H), 7.58 (dd, *J* = 8.6, 1.0 Hz, 2H), 7.43 – 7.30 (m, 2H), 7.24 – 7.13 (m, 1H), 2.55 – 2.31 (m, 1H), 2.19 – 1.99 (m, 1H), 1.05 – 0.96 (m, 6H), 0.92 (d, *J* = 6.8 Hz, 3H).

^{13}C NMR (101 MHz, $CDCl_3$) δ 162.6 (t, *J* = 28.9 Hz), 136.1, 129.2, 125.5, 120.3, 120.2 (t, *J* = 257.2 Hz), 41.4 (t, *J* = 20.6 Hz), 26.3 (t, *J* = 2.5 Hz), 21.9, 17.4 (t, *J* = 1.6 Hz), 6.8 (dd, *J* = 5.6, 4.2 Hz).

^{19}F NMR (376 MHz, $CDCl_3$) δ -109.8 (1F, dd, *J* = 252.6, 19.4 Hz), -111.4 (1F, dd, *J* = 252.6, 16.9 Hz).

HRMS (ESI) *m/z*: $[M+H]^+$ Calcd. for $C_{13}H_{18}F_2NO$, 242.1351; found, 242.1357.

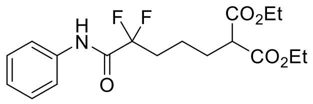

diethyl 2-(4,4-difluoro-5-oxo-5-(phenylamino)pentyl)malonate (18): Following the general procedure A, obtained in 80% yield as colorless oil after silica gel chromatography. (59.5 mg, eluent: petroleum ether/ethyl acetate = 5/1).

^{1}H NMR (400 MHz, $CDCl_3$) δ 8.01 (s, 1H), 7.62 – 7.54 (m, 2H), 7.37 (t, J = 8.0 Hz, 2H), 7.19 (t, J = 7.4 Hz, 1H), 4.19 (qd, J = 7.1, 1.5 Hz, 4H), 3.34 (t, J = 7.4 Hz, 1H), 2.22 (ddd, J = 25.4, 17.2, 8.0 Hz, 2H), 1.98 (dd, J = 15.9, 7.6 Hz, 2H), 1.64 – 1.54 (m, 2H), 1.25 (t, J = 7.1 Hz, 6H).

^{13}C NMR (101 MHz, $CDCl_3$) δ 169.1, 161.9 (t, J = 28.6 Hz), 136.0, 129.2, 125.6, 120.2, 118.0 (t, J = 253.7 Hz), 61.5, 51.7, 33.3 (t, J = 23.4 Hz), 28.1, 19.5 (t, J = 4.4 Hz), 14.0.

^{19}F NMR (376 MHz, CDCl3) δ -105.5 (2F, td, J = 17.3, 2.9 Hz).

HRMS (ESI) *m/z*: $[M+Na]^+$ Calcd. for $C_{18}H_{23}F_2NO_5Na$, 394.1437; found, 394.1443.

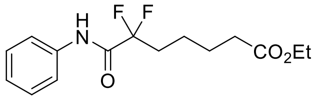

ethyl 6,6-difluoro-7-oxo-7-(phenylamino)heptanoate (19): Following the general procedure A, obtained in 85% yield as colorless oil after silica gel chromatography. (52.6 mg, eluent: petroleum ether/ethyl acetate = 5/1).

^{1}H NMR (400 MHz, $CDCl_3$) δ 8.02 (s, 1H), 7.58 (d, J = 8.2 Hz, 2H), 7.37 (t, J = 7.8 Hz, 2H), 7.19 (t, J = 7.4 Hz, 1H), 4.12 (q, J = 7.1 Hz, 2H), 2.33 (t, J = 7.3 Hz, 2H), 2.28 – 2.13 (m, 2H), 1.77 – 1.65 (m, 2H), 1.63 – 1.49 (m, 2H), 1.24 (t, J = 7.1 Hz, 3H).

^{13}C NMR (101 MHz, $CDCl_3$) δ 173.2, 162.0 (t, J = 28.8 Hz), 136.0, 129.2, 125.6, 120.2, 118.2 (t, J = 253.7 Hz), 60.4, 33.9, 33.5 (t, J = 23.3 Hz), 24.4, 21.2 (t, J = 4.4 Hz), 14.2.

^{19}F NMR (376 MHz, $CDCl_3$) δ -105.5 (2F, td, J = 17.4, 3.1 Hz).

HRMS (ESI) *m/z*: $[M+Na]^+$ Calcd. for $C_{15}H_{19}F_2NO_3Na$, 322.1225; found, 322.1231.

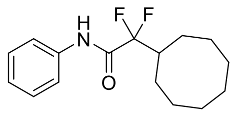

2-cyclooctyl-2,2-difluoro-*N*-phenylacetamide (20): Following the general procedure A, obtained in 80% yield as white solid after silica gel chromatography. (47.0 mg, eluent: petroleum ether/ethyl acetate = 30/1).

Mp = 52 – 54 °C

^{1}H NMR (400 MHz, $CDCl_3$) δ 8.03 (s, 1H), 7.58 (d, J = 8.0 Hz, 2H), 7.37 (t, J = 7.8 Hz, 2H), 7.19 (t, J = 7.4 Hz, 1H), 2.89 – 2.39 (m, 1H), 1.99 – 1.69 (m, 4H), 1.67 – 1.36 (m, 10H).

^{13}C NMR (101 MHz, $CDCl_3$) δ 162.5 (t, J = 29.1 Hz), 136.1, 129.2, 125.5, 120.23 (t, J = 253.4 Hz), 120.21, 40.6 (t, J = 20.5 Hz), 26.5, 26.2, 25.5 (t, J = 3.7 Hz), 25.4.

^{19}F NMR (376 MHz, $CDCl_3$) δ -112.21 (2F, dd, J = 17.5, 2.1 Hz).

HRMS (ESI) *m/z*: $[M+H]^+$ Calcd. for $C_{16}H_{22}F_2NO$, 282.1664; found, 282.1670.

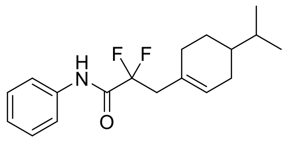

2,2-difluoro-3-(4-isopropylcyclohex-1-en-1-yl)-*N*-phenylpropanamide (21): Following the general procedure A, obtained in 87% yield as white solid after silica

gel chromatography. (53.5 mg, eluent: petroleum ether/ethyl acetate = 30/1). The compound data was in agreement with the literature (*Science* **2021**, *371*, 1232–1240).

^{1}H NMR (400 MHz, $CDCl_3$) δ 7.95 (s, 1H), 7.54 (d, *J* = 7.9 Hz, 2H), 7.36 (t, *J* = 7.9 Hz, 2H), 7.19 (t, *J* = 7.4 Hz, 1H), 5.67 (s, 1H), 2.82 (t, *J* = 17.5 Hz, 2H), 2.23 – 1.94 (m, 3H), 1.87 – 1.67 (m, 2H), 1.44 (dd, *J* = 13.1, 6.5 Hz, 1H), 1.35 – 1.08 (m, 2H), 0.85 (t, *J* = 6.5 Hz, 6H).

^{13}C NMR (101 MHz, $CDCl_3$) δ 162.2 (t, *J* = 28.6 Hz), 136.0, 129.2, 129.1, 128.1, 125.6, 120.3, 117.7 (t, *J* = 249.5 Hz), 41.7 (t, *J* = 23.2 Hz), 39.5, 32.1, 30.1, 29.2, 26.4, 19.9, 19.6.

^{19}F NMR (376 MHz, $CDCl_3$) δ -103.9 (2F, td, *J* = 17.4, 2.5 Hz).

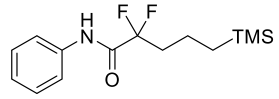

2,2-difluoro-*N*-phenyl-5-(trimethylsilyl)pentanamide (22): Following the general procedure A, obtained in 85% yield as colorless oil after silica gel chromatography. (48.3 mg, eluent: petroleum ether/ethyl acetate = 30/1).

^{1}H NMR (400 MHz, $CDCl_3$) δ 8.21 (s, 1H), 7.73 (d, *J* = 7.8 Hz, 2H), 7.52 (t, *J* = 7.9 Hz, 2H), 7.34 (t, *J* = 7.4 Hz, 1H), 2.46 – 2.21 (m, 2H), 1.87 – 1.57 (m, 2H), 0.84 – 0.59 (m, 2H), 0.15 (s, 9H).

^{13}C NMR (101 MHz, $CDCl_3$) δ 164.3 (t, *J* = 28.8 Hz), 138.1, 131.2, 127.6, 122.3, 120.2 (t, *J* = 253.4 Hz), 39.5 (t, *J* = 22.7 Hz), 18.5, 18.3 (t, *J* = 4.5 Hz), 0.3.

^{19}F NMR (376 MHz, $CDCl_3$) δ -105.5 (2F, td, *J* = 17.5, 3.0 Hz).

HRMS (ESI) *m/z*: $[M+H]^+$ Calcd. for $C_{14}H_{22}F_2NOSi$, 286.1433; found, 286.1432.

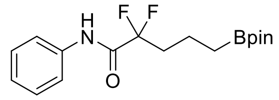

2,2-difluoro-*N*-phenyl-5-(4,4,5,5-tetramethyl-1,3,2-dioxaborolan-2-yl)pentanamide (23): Following the general procedure A, obtained in 73% yield as colorless oil after silica gel chromatography. (49.5 mg, eluent: petroleum ether/ethyl acetate = 20/1).

^{1}H NMR (400 MHz, $CDCl_3$) δ 8.02 (s, 1H), 7.58 (d, *J* = 7.8 Hz, 2H), 7.37 (t, *J* = 7.2 Hz, 2H), 7.19 (t, *J* = 7.4 Hz, 1H), 2.37 – 2.10 (m, 2H), 1.64 (dt, *J* = 15.7, 7.8 Hz, 2H), 1.24 (s, 12H), 0.86 (t, *J* = 7.7 Hz, 2H).

^{13}C NMR (101 MHz, $CDCl_3$) δ 162.2 (t, *J* = 28.6 Hz), 136.1, 129.2, 125.5, 120.2, 118.3 (t, *J* = 253.3 Hz), 83.2, 36.0 (t, *J* = 22.9 Hz), 24.8, 16.3 (t, *J* = 4.6 Hz), 10.8.

^{19}F NMR (376 MHz, $CDCl_3$) δ -105.3 (2F, td, *J* = 17.4, 3.0 Hz).

HRMS (ESI) *m/z*: $[M+Na]^+$ Calcd. for $C_{17}H_{24}BF_2NO_3Na$, 362.1710; found, 362.1705.

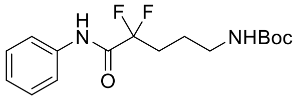

***tert*-butyl (4,4-difluoro-5-oxo-5-(phenylamino)pentyl)carbamate (24):** Following the general procedure A, obtained in 83% yield as colorless oil after silica gel chromatography. (54.5 mg, eluent: petroleum ether/ethyl acetate = 5/1).

^{1}H NMR (400 MHz, $CDCl_3$) δ 8.11 (s, 1H), 7.58 (d, *J* = 7.8 Hz, 2H), 7.36 (t, *J* = 7.4 Hz, 2H), 7.19 (t, *J* = 7.3 Hz, 1H), 4.66 (s, 1H), 3.19 (d, *J* = 4.9 Hz, 2H), 2.50 – 2.02 (m, 2H), 1.84 – 1.67 (m, 2H), 1.43 (s, 9H).

^{13}C NMR (101 MHz, $CDCl_3$) δ 161.9 (t, *J* = 28.7 Hz), 155.9, 136.0, 129.2, 125.6, 120.3, 118.1 (t, *J* = 253.7 Hz), 79.4, 39.8, 31.1 (t, *J* = 23.7 Hz), 28.4, 22.5.

^{19}F NMR (376 MHz, $CDCl_3$) δ -105.3 (2F, t, *J* = 17.2 Hz).

HRMS (ESI) *m/z*: $[M+Na]^+$ Calcd. for $C_{16}H_{22}F_2N_2O_5Na$, 351.1491; found, 351.1500.

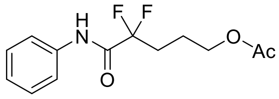

4,4-difluoro-5-oxo-5-(phenylamino)pentyl acetate (25): Following the general procedure A, obtained in 72% yield as colorless oil after silica gel chromatography. (39.1 mg, eluent: petroleum ether/ethyl acetate = 5/1).

^{1}H NMR (400 MHz, $CDCl_3$) δ 8.05 (s, 1H), 7.58 (d, *J* = 7.9 Hz, 2H), 7.37 (t, *J* = 7.9 Hz, 2H), 7.20 (t, *J* = 7.4 Hz, 1H), 4.13 (t, *J* = 6.4 Hz, 2H), 2.39 – 2.19 (m, 2H), 2.06 (s, 3H), 1.98 – 1.82 (m, 2H).

^{13}C NMR (101 MHz, $CDCl_3$) δ 171.0, 161.8 (t, *J* = 28.7 Hz), 136.0, 129.3, 125.7, 120.2, 118.0 (t, *J* = 253.9 Hz), 63.2, 30.6 (t, *J* = 23.7 Hz), 21.1 (t, *J* = 4.4 Hz), 20.9.

^{19}F NMR (376 MHz, $CDCl_3$) δ -105.7 (2F, td, *J* = 17.4, 2.6 Hz).

HRMS (ESI) *m/z*: $[M+Na]^+$ Calcd. for $C_{13}H_{15}F_2NO_3Na$, 294.0912; found, 294.0919.

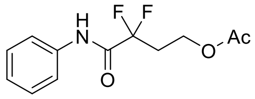

3,3-difluoro-4-oxo-4-(phenylamino)butyl acetate (26): Following the general procedure A, obtained in 69% yield as colorless oil after silica gel chromatography. (35.5 mg, eluent: petroleum ether/ethyl acetate = 5/1).

^{1}H NMR (400 MHz, $CDCl_3$) δ 8.06 (s, 1H), 7.59 (d, J = 7.6 Hz, 2H), 7.37 (t, J = 8.0 Hz, 2H), 7.20 (t, J = 7.4 Hz, 1H), 4.33 (t, J = 6.3 Hz, 2H), 2.68 – 2.49 (m, 2H), 1.97 (s, 3H).

^{13}C NMR (101 MHz, $CDCl_3$) δ 170.7, 161.5 (t, J = 28.1 Hz), 136.0, 129.3, 125.6, 120.2, 117.5 (t, *J* = 253.7 Hz), 57.5 (t, J = 5.9 Hz), 33.2 (t, J = 23.8 Hz), 20.7.

^{19}F NMR (376 MHz, $CDCl_3$) δ -105.5 (2F, td, J = 17.5, 3.0 Hz).

HRMS (ESI) *m/z*: $[M+Na]^+$ Calcd. for $C_{12}H_{13}F_2NO_3Na$, 280.0756; found, 280.0763.

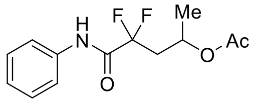

4,4-difluoro-5-oxo-5-(phenylamino)pentan-2-yl acetate (27): Following the general procedure A, obtained in 58% yield as colorless oil after silica gel chromatography. (31.4 mg, eluent: petroleum ether/ethyl acetate = 5/1).

^{1}H NMR (400 MHz, $CDCl_3$) δ 8.13 (s, 1H), 7.59 (d, *J* = 8.0 Hz, 2H), 7.36 (t, *J* = 7.9 Hz, 2H), 7.19 (t, *J* = 7.4 Hz, 1H), 5.27 (ddq, *J* = 12.6, 6.3, 3.1 Hz, 1H), 2.75 – 2.52 (m, 1H), 2.37 (dtd, *J* = 17.9, 14.7, 3.3 Hz, 1H), 1.89 (s, 3H), 1.32 (d, *J* = 6.4 Hz, 3H).

^{13}C NMR (101 MHz, $CDCl_3$) δ 170.5, 161.6 (t, *J* = 28.1 Hz), 136.1, 129.2, 125.6, 120.1, 116.7 (t, *J* = 243.9 Hz), 64.9 (t, *J* = 5.2 Hz), 39.7 (t, *J* = 23.3 Hz), 21.0, 20.8.

^{19}F NMR (376 MHz, $CDCl_3$) δ -102.9 – -105.9 (2F, m).

HRMS (ESI) *m/z*: $[M+Na]^+$ Calcd. for $C_{13}H_{15}F_2NO_3Na$, 294.0912; found, 294.0913.

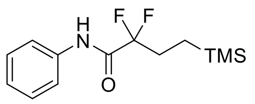

2,2-difluoro-*N*-phenyl-4-(trimethylsilyl)butanamide (28): Following the general procedure A, obtained in 90% yield as colorless oil after silica gel chromatography. (48.7 mg, eluent: petroleum ether/ethyl acetate = 30/1).

^{1}H NMR (400 MHz, $CDCl_3$) δ 8.03 (s, 1H), 7.59 (d, J = 7.9 Hz, 2H), 7.37 (t, J = 7.9 Hz, 2H), 7.19 (t, J = 7.4 Hz, 1H), 2.30 – 2.02 (m, 2H), 0.78 – 0.56 (m, 2H), 0.03 (s, 9H).

^{13}C NMR (101 MHz, $CDCl_3$) δ 164.6 (t, J = 28.9 Hz), 138.4, 131.5, 127.8, 122.5, 121.2 (t, J = 253.7 Hz), 31.0 (t, J = 24.3 Hz), 10.0 (t, J = 2.7 Hz), 0.3.

^{19}F NMR (376 MHz, $CDCl_3$) δ -107.1 (2F, td, J = 16.7, 2.9 Hz).

HRMS (ESI) *m/z*: $[M+Na]^+$ Calcd. for $C_{13}H_{19}F_2NOSiNa$, 294.1096; found, 294.1100.

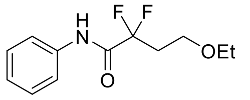

4-ethoxy-2,2-difluoro-*N*-phenylbutanamide (29): Following the general procedure A, obtained in 76% yield as colorless oil after silica gel chromatography. (36.9 mg, eluent: petroleum ether/ethyl acetate = 5/1).

^{1}H NMR (400 MHz, $CDCl_3$) δ 8.06 (s, 1H), 7.57 (d, J = 8.1 Hz, 2H), 7.36 (t, J = 7.0 Hz, 2H), 7.18 (t, J = 7.2 Hz, 1H), 3.66 (t, J = 6.3 Hz, 2H), 3.46 (q, J = 7.0 Hz, 2H), 2.61 – 2.40 (m, 2H), 1.10 (t, J = 7.0 Hz, 3H).

^{13}C NMR (101 MHz, $CDCl_3$) δ 161.9 (t, J = 28.4 Hz), 136.2, 129.2, 125.4, 120.1, 161.8 (t, J = 253.7 Hz), 66.5, 63.6 (t, J = 6.0 Hz), 34.3 (t, J = 23.5 Hz), 14.9.

^{19}F NMR (376 MHz, $CDCl_3$) δ -104.4 (2F, td, J = 16.1, 2.8 Hz).

HRMS (ESI) *m/z*: $[M+Na]^+$ Calcd. for $C_{12}H_{15}F_2NO_2Na$, 266.0963; found, 266.0970.

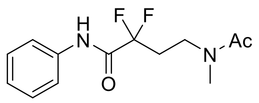

2,2-difluoro-4-(*N*-methylacetamido)-*N*-phenylbutanamide (30): Following the general procedure A, obtained in 85% yield as colorless oil after silica gel chromatography. (45.9 mg, eluent: petroleum ether/ethyl acetate = 5/1).

^{1}H NMR (400 MHz, $CDCl_3$) δ 8.96 (s, 1H), 7.66 (dd, J = 8.6, 1.0 Hz, 2H), 7.39 – 7.32 (m, 2H), 7.17 (t, J = 7.4 Hz, 1H), 3.65 (t, J = 6.5 Hz, 2H), 3.01 (s, 3H), 2.51 – 2.30 (m, 2H), 2.00 (s, 3H).

^{13}C NMR (101 MHz, $CDCl_3$) δ 171.5, 161.8 (t, J = 28.5 Hz), 136.5, 129.1, 125.3, 120.4, 117.3 (t, J = 253.7 Hz), 41.2 (t, J = 5.6 Hz), 36.0, 31.4 (t, J = 23.7 Hz), 21.8.

^{19}F NMR (376 MHz, $CDCl_3$) δ -104.7 (2F, td, J = 16.0, 1.9 Hz).

HRMS (ESI) *m/z*: $[M+Na]^+$ Calcd. for $C_{13}H_{16}F_2N_2O_2Na$, 293.1072; found, 293.1075.

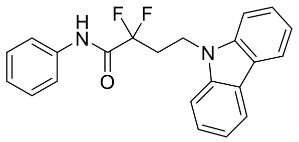

4-(9H-carbazol-9-yl)-2,2-difluoro-*N*-phenylbutanamide (31): Following the general procedure A, obtained in 73% yield as white solid after silica gel chromatography. (53.1 mg, eluent: petroleum ether/ethyl acetate = 10/1).

Mp = 155 – 157 °C

^{1}H NMR (400 MHz, $CDCl_3$) δ 8.07 (d, J = 7.7 Hz, 2H), 7.88 (s, 1H), 7.54 – 7.41 (m, 6H), 7.36 (t, J = 8.0 Hz, 2H), 7.26 – 7.17 (m, 3H), 4.69 – 4.60 (m, 2H), 2.91 – 2.61 (m, 2H).

^{13}C NMR (101 MHz, $CDCl_3$) δ 161.2 (t, J = 27.9 Hz), 139.8, 135.7, 129.2, 125.9, 125.8, 123.2, 120.5, 120.3, 119.4, 117.2 (t, J = 254.8 Hz), 108.4, 36.2 (t, J = 5.8 Hz), 32.6 (t, J = 23.0 Hz).

^{19}F NMR (376 MHz, $CDCl_3$) δ -104.64 (2F, td, J = 17.0, 2.8 Hz).

HRMS (ESI) *m/z*: $[M+H]^+$ Calcd. for $C_{22}H_{19}F_2N_2O$, 365.1460; found, 365.1460.

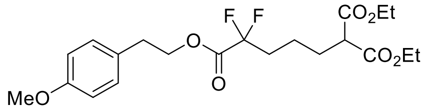

1,1-diethyl 5-(4-methoxyphenethyl) 5,5-difluoropentane-1,1,5-tricarboxylate (32): Following the general procedure B, obtained in 86% yield as colorless oil after silica gel chromatography. (73.9 mg, eluent: petroleum ether/ethyl acetate = 20/1).

^{1}H NMR (400 MHz, $CDCl_3$) δ 7.14 (d, J = 8.5 Hz, 2H), 6.85 (d, J = 8.5 Hz, 2H), 4.42 (t, J = 7.0 Hz, 2H), 4.20 (q, J = 7.0 Hz, 4H), 3.79 (s, 3H), 3.29 (t, J = 7.5 Hz, 1H), 2.95 (t, J = 7.0 Hz, 2H), 2.12 – 1.96 (m, 2H), 1.91 (dd, J = 15.8, 7.7 Hz, 2H), 1.60 – 1.37 (m, 2H), 1.27 (t, J = 7.1 Hz, 6H).

^{13}C NMR (101 MHz, $CDCl_3$) δ 169.0, 164.0 (t, *J* = 33.0 Hz), 158.5, 129.9, 128.7, 115.9 (t, *J* = 250.4 Hz), 114.0, 67.2, 61.5, 55.2, 51.6, 34.1 (t, *J* = 23.4 Hz), 33.9, 28.0, 19.4 (t, *J* = 4.4 Hz), 14.0.

^{19}F NMR (376 MHz, $CDCl_3$) δ -106.0 (2F, t, *J* = 16.7 Hz).

HRMS (ESI) *m/z*: $[M+H]^+$ Calcd. for $C_{21}H_{29}F_2O_7$, 431.1876; found, 431.1875.

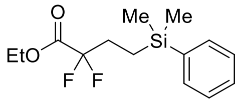

ethyl 4-(dimethyl(phenyl)silyl)-2,2-difluorobutanoate (33): Following the general procedure B, obtained in 83% yield as colorless oil after silica gel chromatography. (47.5 mg, eluent: petroleum ether).

^1H NMR (400 MHz, $CDCl_3$) δ 7.46 – 7.36 (m, 2H), 7.35 – 7.22 (m, 3H), 4.22 (q, *J* = 7.1 Hz, 2H), 2.10 – 1.74 (m, 2H), 1.25 (t, *J* = 7.1 Hz, 3H), 0.94 – 0.70 (m, 2H), 0.23 (s, 6H).

^{13}C NMR (101 MHz, $CDCl_3$) δ 164.4 (t, *J* = 33.2 Hz), 137.6, 133.5, 129.3, 127.9, 116.8 (t, *J* = 250.2 Hz), 62.7, 29.4 (t, *J* = 24.3 Hz), 13.9, 6.9 (t, *J* = 2.6 Hz), -3.4.

^{19}F NMR (376 MHz, $CDCl_3$) δ -107.4 (2F, t, *J* = 16.1 Hz).

HRMS (ESI) *m/z*: $[M+Na]^+$ Calcd. for $C_{14}H_{20}F_2O_2Si$, 309.1093; found, 309.1090.

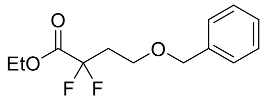

ethyl 4-(benzyloxy)-2,2-difluorobutanoate (34): Following the general procedure B, obtained in 85% yield as colorless oil after silica gel chromatography. (43.9 mg, eluent: petroleum ether/ethyl acetate = 30/1).

^1H NMR (400 MHz, $CDCl_3$) δ 7.38 – 7.26 (m, 5H), 4.46 (s, 2H), 4.17 (q, *J* = 7.1 Hz, 2H), 3.65 (t, *J* = 6.1 Hz, 2H), 2.52 – 2.33 (m, 2H), 1.24 (t, *J* = 7.2 Hz, 3H).

^{13}C NMR (101 MHz, $CDCl_3$) δ 164.0 (t, *J* = 34.1 Hz), 137.7, 128.4, 127.8, 127.7, 115.3 (t, *J* = 249.9 Hz), 73.3, 63.4 (t, *J* = 6.3 Hz), 62.7, 35.3 (t, *J* = 23.6 Hz), 13.8.

^{19}F NMR (376 MHz, $CDCl_3$) δ -105.7 (2F, t, *J* = 15.3 Hz).

HRMS (ESI) *m/z*: $[M+Na]^+$ Calcd. for $C_{13}H_{16}F_2O_3Si$, 281.0960; found, 281.0969.

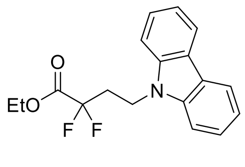

ethyl 4-(9H-carbazol-9-yl)-2,2-difluorobutanoate (35): Following the general procedure B, obtained in 89% yield as colorless oil after silica gel chromatography. (56.4 mg, eluent: petroleum ether/ethyl acetate = 30/1).

^{1}H NMR (400 MHz, $CDCl_3$) δ 8.07 (d, *J* = 7.8 Hz, 2H), 7.47 (ddd, *J* = 8.2, 7.1, 1.2 Hz, 2H), 7.38 (d, *J* = 8.2 Hz, 2H), 7.31 – 7.20 (m, 2H), 4.65 – 4.39 (m, 2H), 4.12 (q, *J* = 7.1 Hz, 2H), 2.83 – 2.43 (m, 2H), 1.20 (t, *J* = 7.2 Hz, 3H).

^{13}C NMR (101 MHz, $CDCl_3$) δ 163.4 (t, *J* = 32.3 Hz), 139.8, 126.0, 123.2, 120.6, 119.5, 115.1 (t, *J* = 250.9 Hz), 108.4, 63.2, 35.9 (t, *J* = 5.9 Hz), 33.3 (t, *J* = 23.2 Hz), 13.8.

^{19}F NMR (376 MHz, $CDCl_3$) δ -105.9 (2F, t, *J* = 16.6 Hz).

HRMS (ESI) *m/z*: $[M+H]^+$ Calcd. for $C_{18}H_{18}F_2NO_2$, 318.1300; found, 318.1313.

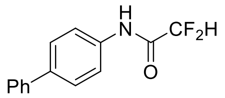

***N*-([1,1'-biphenyl]-4-yl)-2,2-difluoroacetamide (36):** Following the general procedure C, obtained in 80% yield as white solid after silica gel chromatography. (39.4 mg, eluent: petroleum ether/ethyl acetate = 10/1).

Mp = 163 – 165 ^{o}C

^{1}H NMR (400 MHz, *d*$_6$-DMSO) δ 10.85 (s, 1H), 7.83 – 7.75 (m, 2H), 7.73 – 7.62 (m, 4H), 7.52 – 7.42 (m, 2H), 7.41 – 7.31 (m, 1H), 6.42 (t, *J* = 53.7 Hz, 1H).

^{13}C NMR (101 MHz, *d*$_6$-DMSO) δ 165.8 (t, *J* = 25.9 Hz), 144.6, 141.9, 141.6, 134.2, 132.5, 132.3, 131.6, 125.8, 113.6 (t, *J* = 246.6 Hz).

^{19}F NMR (376 MHz, *d*$_6$-DMSO) δ -125.1 (2F, d, *J* = 53.6 Hz).

HRMS (ESI) *m/z*: $[M+Na]^+$ Calcd. for $C_{14}H_{11}F_2NONa$, 270.0701; found, 270.0707.

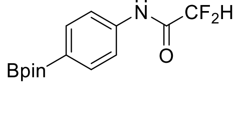

2,2-difluoro-N-(4-(4,4,5,5-tetramethyl-1,3,2-dioxaborolan-2-yl)phenyl)acetamide (37): Following the general procedure C, obtained in 75% yield as colorless oil after silica gel chromatography. (44.5 mg, eluent: petroleum ether/ethyl acetate = 10/1).

^{1}H NMR (400 MHz, $CDCl_3$) δ 8.00 (s, 1H), 7.82 (d, *J* = 8.5 Hz, 2H), 7.59 (d, *J* = 8.5 Hz, 2H), 6.01 (t, *J* = 54.4 Hz, 1H), 1.34 (s, 12H).

^{13}C NMR (101 MHz, $CDCl_3$) δ 160.3 (t, *J* = 24.9 Hz), 138.2, 135.9, 119.2, 108.5 (t, *J* = 254.3 Hz), 83.9, 24.8.

^{19}F NMR (376 MHz, $CDCl_3$) δ -125.5 (2F, d, *J* = 54.3 Hz).

HRMS (ESI) *m/z*: $[M+H]^+$ Calcd. for $C_{14}H_{19}BF_2NO_3$, 298.1421; found, 298.1415.

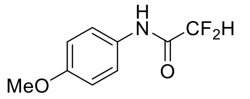

2,2-difluoro-*N*-(4-methoxyphenyl)acetamide (38): Following the general procedure C, obtained in 90% yield as white solid after silica gel chromatography. (36.2 mg, eluent: petroleum ether/ethyl acetate = 15/1). The compound data was in agreement with the literature (*Science* **2021**, *371*, 1232–1240).

^{1}H NMR (400 MHz, $CDCl_3$) δ 7.93 (s, 1H), 7.47 (d, *J* = 8.9 Hz, 2H), 6.89 (d, *J* = 9.0 Hz, 2H), 6.01 (t, *J* = 54.4 Hz, 1H), 3.80 (s, 3H).

^{13}C NMR (101 MHz, $CDCl_3$) δ 160.2 (t, *J* = 24.4 Hz), 157.4, 128.6, 122.2, 114.4, 108.6 (t, *J* = 253.8 Hz), 55.5.

^{19}F NMR (376 MHz, $CDCl_3$) δ -125.5 (2F, dd, *J* = 54.4, 2.0 Hz).

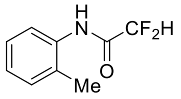

2,2-difluoro-*N*-(o-tolyl)acetamide (39): Following the general procedure C, obtained in 83% yield as white solid after silica gel chromatography. (30.7 mg, eluent: petroleum ether/ethyl acetate = 15/1).

Mp = 64 – 66 °C

^{1}H NMR (400 MHz, $CDCl_3$) δ 7.83 (d, *J* = 7.9 Hz, 1H), 7.77 (s, 1H), 7.25 – 7.20 (m, 2H), 7.16 (t, *J* = 7.4 Hz, 1H), 6.04 (t, *J* = 54.4 Hz, 1H), 2.29 (s, 3H).

^{13}C NMR (101 MHz, $CDCl_3$) δ 160.4 (t, *J* = 24.2 Hz), 133.4, 130.8, 129.5, 127.1, 126.5, 122.9, 108.8 (t, *J* = 254.0 Hz), 17.4.

^{19}F NMR (376 MHz, $CDCl_3$) δ -125.4 (2F, dd, *J* = 54.4, 2.5 Hz).

HRMS (ESI) *m/z*: $[M+H]^+$ Calcd. for $C_9H_{10}F_2NO$, 186.0725; found, 186.0721.

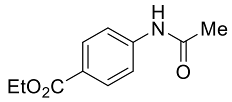

ethyl 4-acetamidobenzoate (40): Following the general procedure C, obtained in 96% yield as white solid after silica gel chromatography. (39.8 mg, eluent: petroleum ether/ethyl acetate = 1/1). The compound data was in agreement with the literature (*Angew. Chem., Int. Ed.* **2016**, *55*, 3823–3827).

^1H NMR (400 MHz, $CDCl_3$) δ 7.99 (d, *J* = 8.5 Hz, 2H), 7.94 (s, 1H), 7.61 (d, *J* = 8.3 Hz, 2H), 4.36 (q, *J* = 7.1 Hz, 2H), 2.20 (s, 3H), 1.38 (t, *J* = 7.1 Hz, 3H).

^{13}C NMR (101 MHz, $CDCl_3$) δ 168.8, 166.3, 142.2, 130.7, 125.8, 118.8, 60.9, 24.7, 14.3.

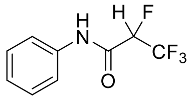

2,3,3,3-tetrafluoro-*N*-phenylpropanamide (41): Following the general procedure C, obtained in 90% yield as white solid after silica gel chromatography. (39.8 mg, eluent: petroleum ether/ethyl acetate = 15/1). The compound data was in agreement with the literature (*Science* **2021**, *371*, 1232–1240).

^1H NMR (400 MHz, $CDCl_3$) δ 7.96 (s, 1H), 7.57 (dd, *J* = 8.5, 1.0 Hz, 2H), 7.39 (dd, *J* = 10.8, 5.1 Hz, 2H), 7.22 (t, *J* = 7.4 Hz, 1H), 5.21 (dq, *J* = 46.5, 6.4 Hz, 1H).

^{13}C NMR (101 MHz, $CDCl_3$) δ 158.9 (d, *J* = 18.2 Hz), 135.7, 129.3, 125.9, 120.5 (qd, *J* = 280.4, 25.7 Hz), 120.3, 85.7 (dq, *J* = 205.4, 34.0 Hz).

^{19}F NMR (376 MHz, $CDCl_3$) δ -75.7 (3F, d, *J* = 11.0 Hz), -200.1 (1F, q, *J* = 11.0 Hz).

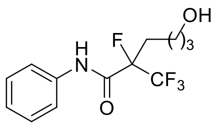

2-fluoro-6-hydroxy-*N*-phenyl-2-(trifluoromethyl)hexanamide (43): Following the general procedure C, obtained in 80% yield as white solid after silica gel chromatography. (46.9 mg, eluent: petroleum ether/ethyl acetate = 1/1).

Mp = 65– 67 °C

^{1}H NMR (400 MHz, $CDCl_3$) δ 8.16 (s, 1H), 7.57 (dd, *J* = 8.5, 0.9 Hz, 2H), 7.37 (t, *J* = 8.0 Hz, 2H), 7.20 (t, *J* = 7.4 Hz, 1H), 3.65 (td, *J* = 6.1, 1.6 Hz, 2H), 2.53 – 2.27 (m, 1H), 2.16 – 2.00 (m, 1H), 1.70 (s, 1H), 1.68 – 1.57 (m, 3H), 1.56 – 1.41 (m, 1H).

^{13}C NMR (101 MHz, $CDCl_3$) δ 162.1 (d, *J* = 19.5 Hz), 136.0, 129.2, 125.7, 121.8 (qd, *J* = 285.1, 28.5 Hz), 120.5, 95.9 (dq, *J* = 202.7, 30.3 Hz), 61.8, 31.9, 30.12 (d, *J* = 20.2 Hz), 18.5 (d, *J* = 2.5 Hz).

^{19}F NMR (376 MHz, $CDCl_3$) δ -78.3 (3F, d, *J* = 6.7 Hz), -169.8 – -183.4 (1F, m).

HRMS (ESI) *m/z*: $[M+H]^+$ Calcd. for $C_{13}H_{16}F_4NO_2$, 294.1112; found, 294.1116.

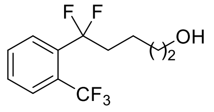

5,5-difluoro-5-(2-(trifluoromethyl)phenyl)pentan-1-ol (44): Following the general procedure D, obtained in 81% yield as colorless oil after silica gel chromatography. (43.4 mg, eluent: petroleum ether/ethyl acetate = 2/1). The compound data was in agreement with the literature (*J. Am. Chem. Soc.* **2018**, *140*, 163−166).

^{1}H NMR (400 MHz, $CDCl_3$) δ 7.78 (d, *J* = 7.9 Hz, 1H), 7.67 – 7.56 (m, 2H), 7.52 (t, *J* = 7.3 Hz, 1H), 3.63 (t, *J* = 6.1 Hz, 2H), 2.40 – 2.10 (m, 2H), 1.96 (s, 1H), 1.70 – 1.55 (m, 4H).

^{13}C NMR (101 MHz, $CDCl_3$) δ 136.1 (t, *J* = 28.0 Hz), 131.8, 129.8, 128.1 (t, *J* = 9.1 Hz), 127.5 (q, *J* = 6.5 Hz), 127.1 (q, *J* = 32.4 Hz), 123.6 (q, J = 273.5 Hz), 122.5 (t, J = 244.8 Hz), 62.5, 39.3 (t, *J* = 26.9 Hz), 32.1, 18.7 (t, *J* = 3.6 Hz).

^{19}F NMR (376 MHz, $CDCl_3$) δ -57.7 (3F, t, *J* = 16.1 Hz), -93.3 (2F, h, *J* = 16.5 Hz).

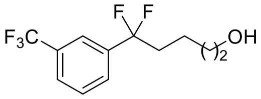

5,5-difluoro-5-(3-(trifluoromethyl)phenyl)pentan-1-ol (45): Following the general procedure D, obtained in 80% yield as colorless oil after silica gel chromatography.

(42.9 mg, eluent: petroleum ether/ethyl acetate = 2/1). The compound data was in agreement with the literature (*J. Am. Chem. Soc.* **2018**, *140*, 163−166).

^{1}H NMR (400 MHz, $CDCl_3$) δ 7.63 (s, 1H), 7.60 – 7.51 (m, 2H), 7.44 (t, *J* = 7.8 Hz, 1H), 3.49 (t, *J* = 6.1 Hz, 2H), 2.37 – 1.74 (m, 2H), 1.72 – 1.19 (m, 4H).

^{13}C NMR (101 MHz, $CDCl_3$) δ 138.4 (t, *J* = 27.5 Hz), 130.9 (q, *J* = 32.7 Hz), 129.1, 128.4 (t, *J* = 5.6 Hz), 126.7 – 126.4 (m), 123.8 (q, *J* = 272.3 Hz), 122.3 (t, *J* = 242.8 Hz), 122.1 – 121.7 (m), 62.1, 38.7 (t, *J* = 27.2 Hz), 31.9, 18.8 (t, *J* = 4.1 Hz).

^{19}F NMR (376 MHz, $CDCl_3$) δ -62.8 (3F, s), -96.2 (2F, t, *J* = 16.4 Hz).

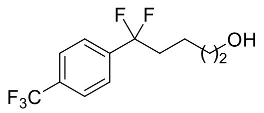

5,5-difluoro-5-(4-(trifluoromethyl)phenyl)pentan-1-ol (46): Following the general procedure D, obtained in 78% yield as colorless oil after silica gel chromatography. (41.8 mg, eluent: petroleum ether/ethyl acetate = 2/1). The compound data was in agreement with the literature (*J. Am. Chem. Soc.* **2018**, *140*, 163−166).

^{1}H NMR (400 MHz, $CDCl_3$) δ 7.68 (d, *J* = 8.1 Hz, 2H), 7.59 (d, *J* = 8.1 Hz, 2H), 3.60 (t, *J* = 6.1 Hz, 2H), 2.30 – 2.00 (m, 2H), 1.80 – 1.38 (m, 4H).

^{13}C NMR (101 MHz, $CDCl_3$) δ 140.9 (t, *J* = 27.4 Hz), 131.8 (q, *J* = 32.7 Hz), 125.7 – 125.4 (m, 2xC), 123.8 (q, *J* = 272.3 Hz), 122.4 (t, *J* = 242.9 Hz), 62.2, 38.7 (t, *J* = 27.1 Hz), 31.9, 18.9 (t, *J* = 4.1 Hz).

^{19}F NMR (376 MHz, $CDCl_3$) δ -62.9 (3F, s), -96.5 (2F, t, *J* = 16.3 Hz).

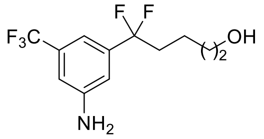

5-(3-amino-5-(trifluoromethyl)phenyl)-5,5-difluoropentan-1-ol (47): Following the general procedure D, obtained in 88% yield as yellow oil after silica gel chromatography. (49.8 mg, eluent: petroleum ether/ethyl acetate = 2/1).

^{1}H NMR (400 MHz, $CDCl_3$) δ 7.05 (s, 1H), 6.92 (s, 1H), 6.90 (s, 1H), 3.62 (t, *J* = 6.2 Hz, 2H), 3.12 (s, 2H), 2.20 – 2.02 (m, 2H), 1.74 – 1.40 (m, 4H).

^{13}C NMR (101 MHz, $CDCl_3$) δ 146.9, 139.5 (t, J = 27.1 Hz), 132.0 (q, J = 32.4 Hz), 123.8 (q, J = 272.5 Hz), 122.4 (t, J = 243.0 Hz), 114.4 (t, J = 5.9 Hz), 112.6 – 112.3 (m), 111.8 – 111.3 (m), 62.4, 38.6 (t, J = 27.3 Hz), 32.1, 18.9 (t, J = 4.1 Hz).

^{19}F NMR (376 MHz, $CDCl_3$) δ -62.9 (3F, s), -96.1 (2F, t, J = 16.3 Hz).

HRMS (ESI) *m/z*: $[M+Na]^+$ Calcd. for $C_{12}H_{14}F_5NONa$, 306.0888; found, 306.0894.

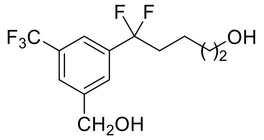

5,5-difluoro-5-(3-(hydroxymethyl)-5-(trifluoromethyl)phenyl)pentan-1-ol (48): Following the general procedure D, obtained in 85% yield as colorless oil after silica gel chromatography. (50.6 mg, eluent: petroleum ether/ethyl acetate = 2/1).

^{1}H NMR (400 MHz, $CDCl_3$) δ 7.67 (s, 1H), 7.64 (d, J = 7.0 Hz, 2H), 4.77 (s, 2H), 3.60 (t, J = 5.9 Hz, 2H), 2.26 (s, 1H), 2.21 – 2.07 (m, 2H), 1.62 – 1.40 (m, 4H).

^{13}C NMR (101 MHz, $CDCl_3$) δ 142.6, 138.6 (t, J = 27.5 Hz), 131.3 (q, J = 32.7 Hz), 126.6 (t, J = 5.6 Hz), 124.9 – 124.5 (m), 123.7 (q, J = 272.5 Hz), 122.4 (t, J = 243.0 Hz), 121.3 – 120.7 (m), 63.9, 62.3, 38.6 (t, J = 27.1 Hz), 31.9, 18.9 (t, J = 4.1 Hz).

^{19}F NMR (376 MHz, $CDCl_3$) δ -62.7 (3F, s), -95.7 (2F, t, J = 16.3 Hz).

HRMS (ESI) *m/z*: $[M+Na]^+$ Calcd. for $C_{13}H_{15}F_5O_2Na$, 321.0884; found, 321.0893.

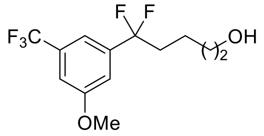

5,5-difluoro-5-(3-methoxy-5-(trifluoromethyl)phenyl)pentan-1-ol (49): Following the general procedure D, obtained in 73% yield as colorless oil after silica gel chromatography. (43.5 mg, eluent: petroleum ether/ethyl acetate = 5/1).

^{1}H NMR (400 MHz, $CDCl_3$) δ 7.30 (s, 1H), 7.17 (s, 2H), 3.88 (s, 3H), 3.64 (t, J = 6.1 Hz, 2H), 2.20 – 2.02 (m, 2H), 1.77 – 1.11 (m, 4H).

^{13}C NMR (101 MHz, $CDCl_3$) δ 159.9, 139.9 (t, J = 27.5 Hz), 132.3 (q, J = 32.7 Hz), 123.5 (q, J = 272.6 Hz), 122.1 (t, J = 243.3 Hz), 114.4 (t, J = 5.8 Hz), 114.3 – 113.5 (m), 112.1– 111.7 (m), 62.4, 55.7, 38.7 (t, J = 27.2 Hz), 32.1, 18.9 (t, J = 4.1 Hz).

^{19}F NMR (376 MHz, $CDCl_3$) δ -62.8 (3F, s), -96.1 (2F, t, J = 16.4 Hz).

HRMS (ESI) *m/z*: $[M+Na]^+$ Calcd. for $C_{13}H_{15}F_5O_2Na$, 321.0884; found, 321.0880.

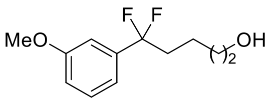

5,5-difluoro-5-(3-methoxyphenyl)pentan-1-ol (50): Following the general procedure D, obtained in 50% yield as colorless oil after silica gel chromatography. (23.0 mg, eluent: petroleum ether/ethyl acetate = 5/1).

^1H NMR (400 MHz, $CDCl_3$) δ 7.33 (t, *J* = 7.9 Hz, 1H), 7.04 (d, *J* = 7.6 Hz, 1H), 6.99 (s, 1H), 6.95 (d, *J* = 8.2 Hz, 1H), 3.83 (s, 3H), 3.63 (t, *J* = 6.2 Hz, 2H), 2.45 – 1.98 (m, 2H), 1.65 – 1.56 (m, 2H), 1.55 – 1.47 (m, 2H).

^{13}C NMR (101 MHz, $CDCl_3$) δ 159.6, 138.8 (t, *J* = 26.6 Hz), 129.6, 122.8 (t, *J* = 242.5 Hz), 117.2 (t, *J* = 6.2 Hz), 115.1, 110.7 (t, *J* = 6.6 Hz), 62.5, 55.4, 38.8 (t, *J* = 27.6 Hz), 32.2, 19.0 (t, *J* = 4.2 Hz).

^{19}F NMR (376 MHz, $CDCl_3$) δ -95.4 (2F, t, *J* = 16.1 Hz).

HRMS (ESI) *m/z*: $[M+Na]^+$ Calcd. for $C_{12}H_{16}F_2O_2Na$, 253.1011; found, 253.1009.

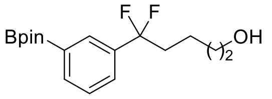

5,5-difluoro-5-(3-(4,4,5,5-tetramethyl-1,3,2-dioxaborolan-2-yl)phenyl)pentan-1-ol (51): Following the general procedure D, obtained in 78% yield as colorless oil after silica gel chromatography. (50.9 mg, eluent: petroleum ether/ethyl acetate = 2/1).

^1H NMR (400 MHz, $CDCl_3$) δ 7.88 (s, 1H), 7.84 (d, *J* = 7.3 Hz, 1H), 7.55 (d, *J* = 7.7 Hz, 1H), 7.41 (t, *J* = 7.5 Hz, 1H), 3.60 (t, *J* = 6.1 Hz, 2H), 2.32 – 2.04 (m, 2H), 1.69 – 1.41 (m, 4H), 1.34 (s, 12H).

^{13}C NMR (101 MHz, $CDCl_3$) δ 136.7 (t, *J* = 26.5 Hz), 136.0, 131.1 (t, *J* = 6.0 Hz), 127.8, 127.7 (t, *J* = 6.0 Hz), 123.1 (t, *J* = 242.3 Hz), 84.1, 62.4, 38.9 (t, *J* = 27.5 Hz), 32.2, 24.9, 18.9 (t, *J* = 4.1 Hz).

^{19}F NMR (376 MHz, $CDCl_3$) δ -95.6 (2F, t, *J* = 16.3 Hz).

HRMS (ESI) *m/z*: $[M+Na]^+$ Calcd. for $C_{17}H_{25}BF_2O_3Na$, 349.1757; found, 349.1787.

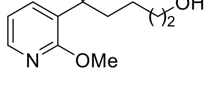

5,5-difluoro-5-(2-methoxypyridin-3-yl)pentan-1-ol (52): Following the general procedure D, obtained in 90% yield as colorless oil after silica gel chromatography. (41.6 mg, eluent: petroleum ether/ethyl acetate = 2/1).

^{1}H NMR (400 MHz, $CDCl_3$) δ 8.22 (d, J = 4.5 Hz, 1H), 7.78 (d, J = 7.4 Hz, 1H), 6.94 (dd, J = 7.4, 5.0 Hz, 1H), 4.00 (s, 3H), 3.63 (t, J = 6.4 Hz, 2H), 2.51 – 2.11 (m, 2H), 1.66 – 1.55 (m, 2H), 1.51 – 1.37 (m, 2H).

^{13}C NMR (101 MHz, $CDCl_3$) δ 160.5 (t, J = 4.4 Hz), 148.3, 135.8 (t, J = 8.3 Hz), 121.8 (t, J = 227.8 Hz), 119.4 (t, J = 27.4 Hz), 116.4, 62.5, 53.7, 36.1 (t, J = 26.1 Hz), 32.2, 19.0 (t, J = 4.2 Hz).

^{19}F NMR (376 MHz, $CDCl_3$) δ -96.3 (2F, t, J = 16.9 Hz).

HRMS (ESI) *m/z*: $[M+H]^+$ Calcd. for $C_{11}H_{16}F_2NO_2$, 232.1144; found, 232.1147.

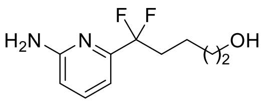

5-(6-aminopyridin-2-yl)-5,5-difluoropentan-1-ol (53): Following the general procedure D, obtained in 57% yield as colorless oil after silica gel chromatography. (24.6 mg, eluent: petroleum ether/ethyl acetate = 2/1).

^{1}H NMR (400 MHz, $CDCl_3$) δ 7.50 (t, J = 7.8 Hz, 1H), 6.93 (d, J = 7.4 Hz, 1H), 6.53 (d, J = 8.3 Hz, 1H), 4.71 (s, 2H), 3.64 (t, J = 6.2 Hz, 2H), 2.26 (ddd, J = 24.3, 16.4, 7.9 Hz, 2H), 1.60 (dt, J = 12.9, 6.6 Hz, 2H), 1.56 – 1.45 (m, 2H).

^{13}C NMR (101 MHz, $CDCl_3$) δ 158.2, 152.9 (t, J = 28.9 Hz), 138.6, 121.6 (t, J = 241.7 Hz), 109.9, 109.8 (t, J = 5.3 Hz), 62.3, 35.9 (t, J = 25.7 Hz), 32.0, 18.6 (t, J = 4.3 Hz).

^{19}F NMR (376 MHz, $CDCl_3$) δ -99.7 (2F, t, J = 16.6 Hz).

HRMS (ESI) *m/z*: $[M+H]^+$ Calcd. for $C_{10}H_{15}F_2N_2O$, 217.1147; found, 217.1146.

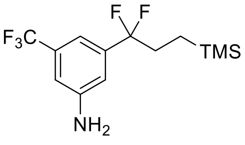

3-(1,1-difluoro-3-(trimethylsilyl)propyl)-5-(trifluoromethyl)aniline (54): Following the general procedure D, obtained in 92% yield as yellow oil after silica gel chromatography. (57.3 mg, eluent: petroleum ether/ethyl acetate = 20/1).

^{1}H NMR (400 MHz, $CDCl_3$) δ 7.21 (s, 1H), 7.07 (s, 1H), 7.05 (s, 1H), 3.96 (s, 2H), 2.31 – 1.94 (m, 2H), 0.87 – 0.57 (m, 2H), 0.15 (s, 9H).

^{13}C NMR (101 MHz, $CDCl_3$) δ 149.2, 141.9 (t, *J* = 27.4 Hz), 134.3 (q, *J* = 32.3 Hz), 126.1 (q, *J* = 272.4 Hz), 125.2 (t, *J* = 243.1 Hz), 116.9 (t, *J* = 5.9 Hz), 114.7 – 114.5 (m), 114.4 – 113.6 (m), 36.0 (t, *J* = 28.5 Hz), 10.9 (t, *J* = 2.6 Hz), 0.2.

^{19}F NMR (376 MHz, $CDCl_3$) δ -62.9 (3F, s), -97.9 (2F, t, *J* = 15.9 Hz).

HRMS (ESI) *m/z*: $[M+H]^+$ Calcd. for $C_{13}H_{19}F_5NSi$, 312.1201; found, 312.1210.

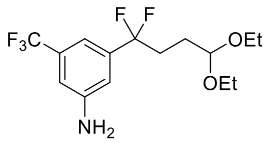

3-(4,4-diethoxy-1,1-difluorobutyl)-5-(trifluoromethyl)aniline (55): Following the general procedure D, obtained in 76% yield as yellow oil after silica gel chromatography. (51.8 mg, eluent: petroleum ether/ethyl acetate = 9/1).

^{1}H NMR (400 MHz, $CDCl_3$) δ 7.06 (s, 1H), 6.92 (s, 1H), 6.90 (s, 1H), 4.49 (t, *J* = 5.6 Hz, 1H), 3.62 (dq, *J* = 9.4, 7.1 Hz, 2H), 3.47 (dq, *J* = 9.4, 7.1 Hz, 2H), 2.33 – 1.98 (m, 2H), 1.91 – 1.71 (m, 2H), 1.19 (t, *J* = 7.1 Hz, 6H).

^{13}C NMR (101 MHz, $CDCl_3$) δ 147.0, 139.4 (t, *J* = 27.1 Hz), 132.1 (q, *J* = 32.4 Hz), 123.8 (q, *J* = 272.4 Hz), 122.3 (t, *J* = 242.8 Hz), 114.3 (t, *J* = 6.0 Hz), 112.5 – 112.2 (m), 111.7 – 111.2 (m), 101.8, 61.5, 34.0 (t, *J* = 27.7 Hz), 26.8 (t, *J* = 3.7 Hz), 15.3.

^{19}F NMR (376 MHz, $CDCl_3$) δ -63.0 (3F, s), -96.4 (2F, t, *J* = 16.6 Hz).

HRMS (ESI) *m/z*: $[M+Na]^+$ Calcd. for $C_{15}H_{20}F_5NO_2Na$, 364.1306; found, 364.1313.

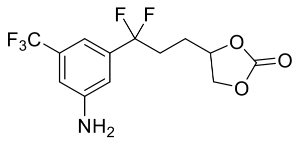

4-(3-(3-amino-5-(trifluoromethyl)phenyl)-3,3-difluoropropyl)-1,3-dioxolan-2-one (56): Following the general procedure D, obtained in 72% yield as yellow oil after silica gel chromatography. (46.8 mg, eluent: petroleum ether/ethyl acetate = 2/1).

^{1}H NMR (400 MHz, $CDCl_3$) δ 7.05 (s, 1H), 6.95 (s, 1H), 6.90 (s, 1H), 4.90 – 4.70 (m, 1H), 4.57 (t, *J* = 8.2 Hz, 1H), 4.25 – 3.97 (m, 1H), 2.48 – 2.11 (m, 2H), 1.96 (dd, *J* = 14.9, 7.3 Hz, 2H).

^{13}C NMR (101 MHz, $CDCl_3$) δ 154.6, 147.3, 138.7 (t, *J* = 26.8 Hz), 132.3 (q, *J* = 32.6 Hz), 123.7 (q, *J* = 272.2 Hz), 121.5 (t, *J* = 243.5 Hz), 114.0 (t, *J* = 5.9 Hz), 112.9 – 112.7 (m), 111.9 – 109.5 (m), 75.9, 69.2, 34.4 (t, *J* = 28.2 Hz), 27.3 (t, *J* = 3.6 Hz).

^{19}F NMR (376 MHz, $CDCl_3$) δ -63.1 (3F, s), -96.7 (1F, ddd, *J* = 245.5, 21.0, 11.7 Hz), -97.8 (1F, ddd, *J* = 245.5, 20.5, 12.6 Hz).

HRMS (ESI) *m/z*: $[M+H]^+$ Calcd. for $C_{13}H_{13}F_5NO_3$, 326.0810; found, 326.0815.

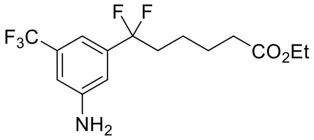

ethyl 6-(3-amino-5-(trifluoromethyl)phenyl)-6,6-difluorohexanoate (57): Following the general procedure D, obtained in 84% yield as yellow oil after silica gel chromatography. (57.0 mg, eluent: petroleum ether/ethyl acetate = 9/1).

^{1}H NMR (400 MHz, $CDCl_3$) δ 7.04 (s, 1H), 6.92 (s, 1H), 6.90 (s, 1H), 4.11 (q, *J* = 7.1 Hz, 2H), 3.63 (s, 2H), 2.30 (t, *J* = 7.4 Hz, 2H), 2.21 – 1.98 (m, 2H), 1.66 (dt, *J* = 15.2, 7.5 Hz, 2H), 1.53 – 1.40 (m, 2H), 1.24 (t, *J* = 7.1 Hz, 3H).

^{13}C NMR (101 MHz, $CDCl_3$) δ 173.4, 146.9, 139.5 (t, *J* = 27.2 Hz), 132.1 (q, *J* = 32.4 Hz), 123.8 (q, *J* = 272.5 Hz), 122.2 (t, *J* = 243.0 Hz), 114.4 (t, *J* = 5.9 Hz), 112.8 – 112.2 (m), 111.9 – 111.2 (m), 60.4, 38.6 (t, *J* = 27.4 Hz), 34.0, 24.5, 21.9 (t, *J* = 4.0 Hz), 14.2.

^{19}F NMR (376 MHz, $CDCl_3$) δ -63.0 (3F, s), -96.2 (2F, t, *J* = 16.3 Hz).

HRMS (ESI) *m/z*: $[M+Na]^+$ Calcd. for $C_{15}H_{18}F_5NO_2Na$, 362.1150; found, 362.1150.

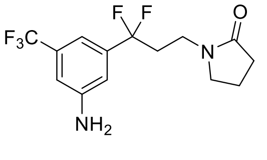

1-(3-(3-amino-5-(trifluoromethyl)phenyl)-3,3-difluoropropyl)pyrrolidin-2-one (58): Following the general procedure D, obtained in 88% yield as yellow oil after silica gel chromatography. (56.7 mg, eluent: petroleum ether/ethyl acetate = 2/1).

^{1}H NMR (400 MHz, $CDCl_3$) δ 7.05 (s, 1H), 6.93 (s, 2H), 3.53 – 3.44 (m, 2H), 3.34 (t, *J* = 7.0 Hz, 2H), 2.46 – 2.34 (m, 2H), 2.34 – 2.28 (m, 2H), 1.95 (dt, *J* = 15.4, 7.5 Hz, 2H).

^{13}C NMR (101 MHz, $CDCl_3$) δ 175.2, 147.1, 138.7 (t, *J* = 26.7 Hz), 132.1 (q, *J* = 32.5 Hz), 123.7 (q, *J* = 272.6 Hz), 121.3 (t, *J* = 243.5 Hz), 114.5 (t, *J* = 6.1 Hz), 112.8 – 112.6 (m), 111.4 – 110.9 (m), 47.3, 36.8 (t, *J* = 5.0 Hz), 36.1 (t, *J* = 27.4 Hz), 30.9, 17.9.

^{19}F NMR (376 MHz, $CDCl_3$) δ -63.0 (3F, s), -96.1 (2F, t, *J* = 16.3 Hz).

HRMS (ESI) *m/z*: $[M+H]^+$ Calcd. for $C_{14}H_{16}F_5N_2O$, 323.1177; found, 323.1183.

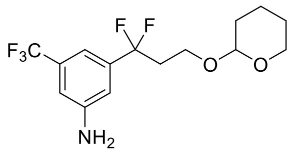

3-(1,1-difluoro-3-((tetrahydro-2H-pyran-2-yl)oxy)propyl)-5-(trifluoromethyl)aniline (59): Following the general procedure D, obtained in 82% yield as yellow oil after silica gel chromatography. (55.6 mg, eluent: petroleum ether/ethyl acetate = 9/1).

^{1}H NMR (400 MHz, $CDCl_3$) δ 7.09 (s, 1H), 6.92 (s, 2H), 4.52 (s, 1H), 3.88 (dt, *J* = 10.2, 6.9 Hz, 1H), 3.77 (ddd, *J* = 11.1, 7.9, 3.0 Hz, 1H), 3.59 – 3.37 (m, 2H), 2.56 – 2.35 (m, 2H), 1.77 – 1.39 (m, 6H).

^{13}C NMR (101 MHz, $CDCl_3$) δ 147.0, 139.2 (t, *J* = 26.8 Hz), 132.0 (q, *J* = 32.4 Hz), 123.8 (q, *J* = 272.5 Hz), 121.5 (t, *J* = 243.1 Hz), 114.3 (t, *J* = 6.3 Hz), 112.5 – 112.3 (m), 112.1 – 111.3 (m), 98.9, 62.2, 61.3 (t, *J* = 5.2 Hz), 39.0 (t, *J* = 27.1 Hz), 30.5, 25.3, 19.3.

^{19}F NMR (376 MHz, $CDCl_3$) δ -63.0 (3F, s), -94.5 (2F, dt, *J* = 39.8, 16.1 Hz).

HRMS (ESI) *m/z*: $[M+H]^+$ Calcd. for $C_{15}H_{19}F_5NO_2$, 340.1330; found, 340.1325.

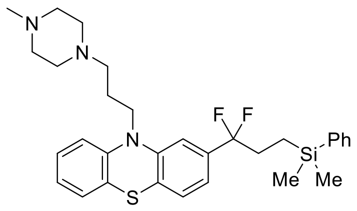

2-(3-(dimethyl(phenyl)silyl)-1,1-difluoropropyl)-10-(3-(4-methylpiperazin-1-yl)propyl)-10*H*-phenothiazine (60): Following the general procedure D, obtained in 70% yield as colorless oil after silica gel chromatography. (77.2 mg, eluent: dichloromethane /methanol = 10/1).

^{1}H NMR (400 MHz, $CDCl_3$) δ 7.50 – 7.41 (m, 2H), 7.33 (dd, *J* = 5.6, 1.4 Hz, 3H), 7.19 – 7.07 (m, 3H), 6.91 (t, *J* = 8.2 Hz, 4H), 3.91 (t, *J* = 6.8 Hz, 2H), 2.47 – 2.42 (m, 10H), 2.25 (s, 3H), 2.11 – 1.99 (m, 2H), 1.96 – 1.81 (m, 2H), 0.92 – 0.71 (m, 2H), 0.26 (s, 6H).

^{13}C NMR (101 MHz, $CDCl_3$) δ 145.4, 144.8, 138.0, 136.5 (t, *J* = 26.9 Hz), 133.5, 129.2, 127.9, 127.5, 127.4, 127.2, 127.0, 124.6, 123.5 (t, *J* = 243.1 Hz), 122.8, 119.2 (t, *J* = 6.0 Hz), 115.8, 112.2 (t, *J* = 6.4 Hz), 55.5, 55.1, 53.2, 46.0, 45.3, 33.8 (t, *J* = 29.2 Hz), 24.3, 8.1 (t, *J* = 2.0 Hz), -3.3.

^{19}F NMR (376 MHz, $CDCl_3$) δ -96.5 (2F, t, *J* = 15.6 Hz).

HRMS (ESI) *m/z*: $[M+H]^+$ Calcd. for $C_{31}H_{40}F_2N_3SSi$, 552.2675; found, 552.2668.

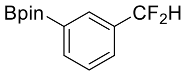

2-(3-(difluoromethyl)phenyl)-4,4,5,5-tetramethyl-1,3,2-dioxaborolane (61): Following the general procedure D, obtained in 70% yield as colorless oil after silica gel chromatography. (35.5 mg, eluent: petroleum ether/ethyl acetate = 15/1).

^{1}H NMR (400 MHz, $CDCl_3$) δ 8.02 – 7.86 (m, 2H), 7.61 (d, *J* = 7.7 Hz, 1H), 7.46 (t, *J* = 7.6 Hz, 1H), 6.64 (t, *J* = 56.4 Hz, 1H), 1.35 (s, 12H).

^{13}C NMR (101 MHz, $CDCl_3$) δ 137.1 (t, *J* = 1.8 Hz), 133.7 (t, *J* = 22.3 Hz), 131.9 (t, *J* = 6.1 Hz), 128.2 (t, *J* = 6.0 Hz), 128.1, 114.9 (t, *J* = 238.6 Hz), 84.2, 24.9.

^{19}F NMR (376 MHz, $CDCl_3$) δ -110.3 (2F, d, *J* = 56.4 Hz).

HRMS (ESI) *m/z*: $[M+Na]^+$ Calcd. for $C_{13}H_{17}BF_2O_2Na$, 277.1182; found, 277.1184.

2-3 Experimental Studies on Mechanism

2-3-1 Measurement of quantum yield

According to the procedure of Glorius[9] and Melchiorre[10], the photon flux of the LEDs (λ_{max} = 427 nm) was determined by standard ferrioxalate actinometry. A 0.15 M solution of ferrioxalate was prepared by dissolving potassium ferrioxalate hydrate (0.737 g) in 10 mL H_2SO_4 (0.05 M). A buffered solution of 1,10-phenanthroline was prepared by dissolving sodium acetate (5.63 g) and phenanthroline (25 mg) in 25 mL H_2SO_4 (0.5 M) at the same time. Both solutions were stored in the dark. To determine the photon flux of the LED, the ferrioxalate solution (1.0 mL) was placed in a cuvette and irradiated for 900 seconds at λ_{max} = 427 nm. After irradiation, the phenanthroline solution (0.175 mL) was added to the cuvette and the mixture was stirred in the dark for 1 hour to allow the ferrous ions to completely coordinate to the phenanthroline. The absorbance of the solution was measured at 510 nm. A non-irradiated sample was also prepared and the absorbance at 510 nm was measured. Using then the Beer's Law, the number of moles of Fe^{2+} produced by light irradiation is obtained by:

$$Fe^{2+} = \frac{v_1 v_3 \Delta A(510\ nm)}{10^3 v_2 l \varepsilon(510\ nm)}$$

where v_1 is the irradiated volume (1 mL), v_2 is the aliquot of the irradiated solution taken for the determination of the ferrous ions (1 mL), v_3 is the final volume after complexation with phenanthroline (10 mL), l is the optical path-length of the irradiation cell (1 cm), ΔA(510 nm) the optical difference in absorbance between the irradiated solution and the one stored in the dark, ε(510 nm) is that of the complex $Fe(phen)_3^{2+}$(11100 L $mol^{-1}cm^{-1}$).

The photon flux (F) is obtained by using the following equation:

$$\phi(\lambda) = \frac{mol\left(Fe^{2+}\right)}{F\left(1-10^{-A(\lambda)}\right)}$$

Wher Φ (λ) = The quantum yield for Fe^{2+} formation at 420 nm is 1.1. A(λ) = ferrioxalate actinometer absorbance at 420 nm, which was measured placing 1 mL of

the solution in a cuvette of pathlength 1 cm by UV/Vis spectrophotometry. We obtained an absorbance value of 3.5. The photon flux (**F**) is **3.1 x 10^{-9} einsteins/s.**

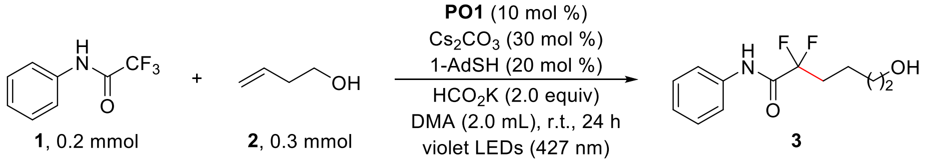

To obtain the quantum yield (Φ). The number of moles of the product **3** were determined by ^{1}H-NMR analysis using diphenylmethane as internal standard. As such, a photocatalytic reaction was performed under the set of optimized reaction conditions under visible light irradiation of 427 nm blue LEDs. After 900 s of light irradiation, 2.0 x 10^{-6} moles of **3** were obtained. The quantum yield of this reaction was calculated using the following equation:

$$\phi(427\ nm) = \frac{moles\ of\ product}{F\left(1\text{-}10^{-A(427\ nm)}\right)t}$$

Where A(427 nm) = is the absorbance at 427 nm of the photocatalytic reaction which was measured placing 1 mL of the solution in a cuvette of path length 1 cm by UV/Vis spectrophotometry.

t = is the reaction time.

The quantum yield (Φ) of the reaction is 4.4.

2-3-2 UV-vis absorption spectroscopic measurements

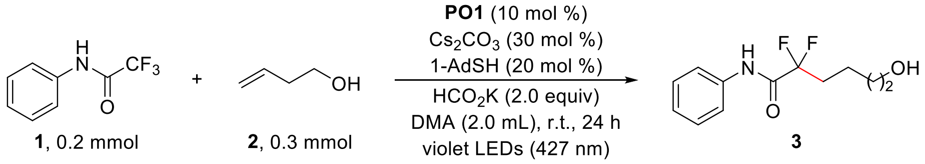

Stock solutions of **1**, **2**, **PO1**, AdSH, HCO_2K were prepared with the same concentration used in the reaction. Cs_2CO_3 was used in 150 mol% to ensure generation of **PO** anion under measurement condition. The solutions were prepared in the presence of air using DMA as solvent.

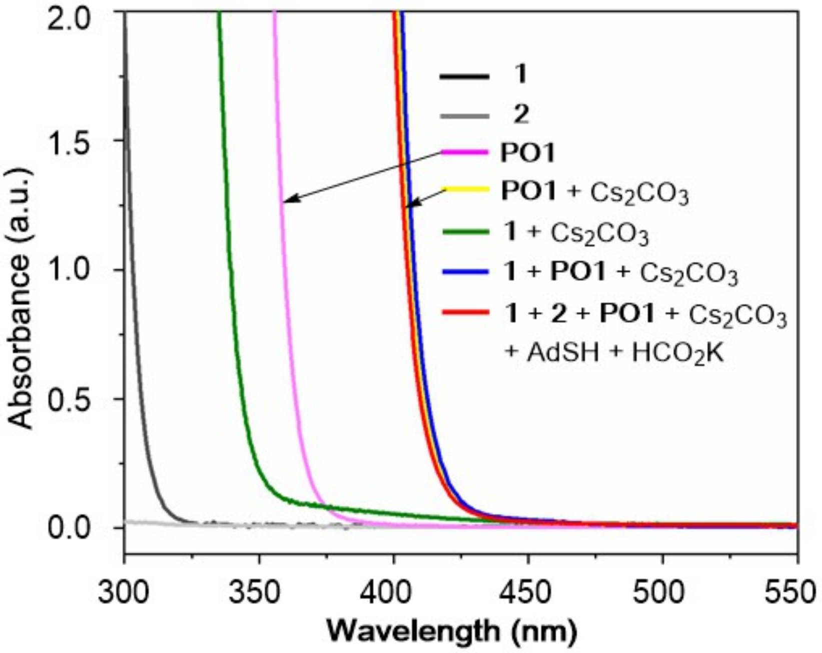

Supplementary Figure 2. UV/vis absorption spectra of catalyst, substrate, and reaction mixture.

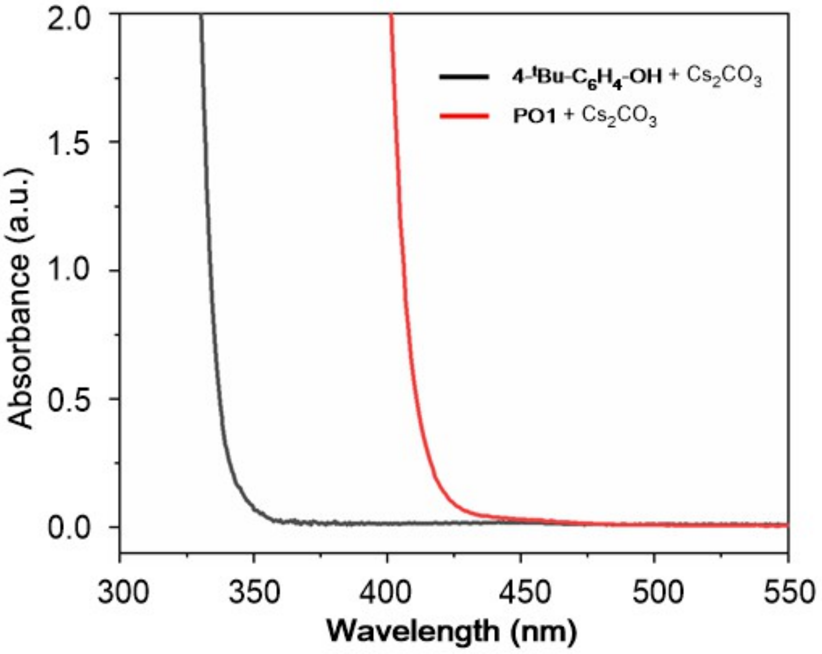

Supplementary Figure 3. UV/vis absorption spectra of 4-tert-Butylphenol anion and **PO1** anion.

2-3-3 Stern-Volmer Quenching Study

To evaluate the role of **PO1** anion in this process, we conducted Stern-Volmer fluorescence quenching experiments.[11] The samples were prepared mixing the **PO1** anion (5×10^{-4} M, freshly prepared in situ by the deprotonation of **PO1** with Cs_2CO_3) with the required amount of **1** in a total volume of 1 mL of dry DMA in a 10 × 10 mm light path quartz fluorescence cuvette under an argon atmosphere. The excitation

wavelength was fixed at 384 nm, the emission light was acquired from 400 nm to 700 nm. Quenching was not observed in similar experiments using **2** and 1-adamantanethiol.

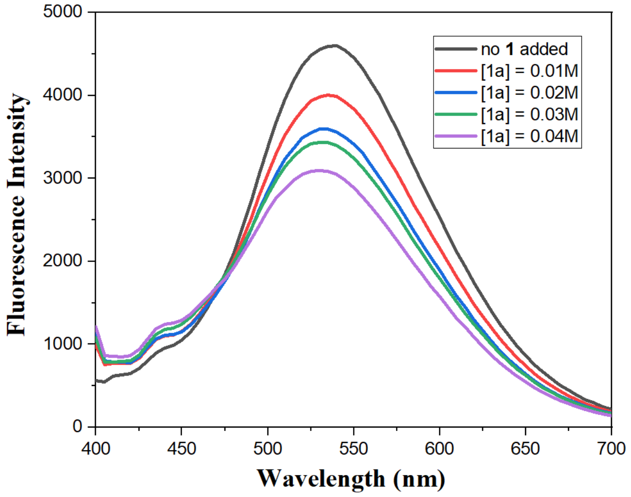

Supplementary Figure 4. Quenching of the **PO1** anion emission (5×10^{-4} M in DMA) in the presence of increasing amounts of **1**.

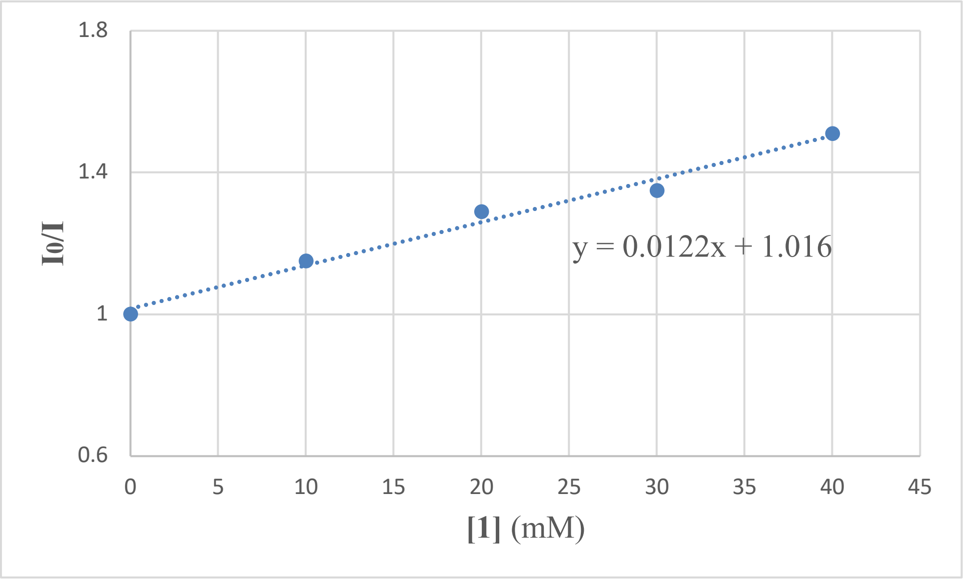

Supplementary Figure 5. Stern-Volmer quenching plot of substrate I.

Other reaction components:

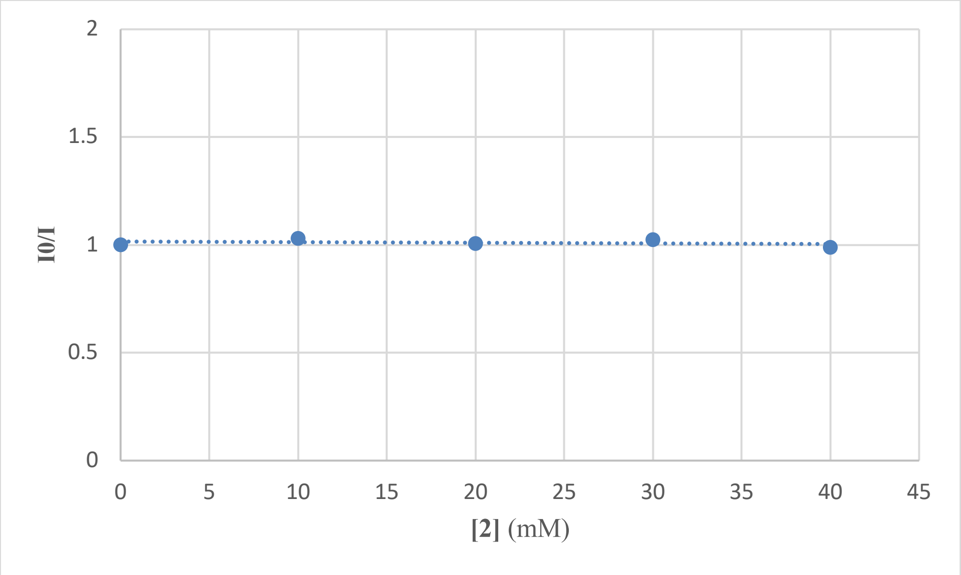

Supplementary Figure 6. Stern-Volmer quenching plot of **2**.

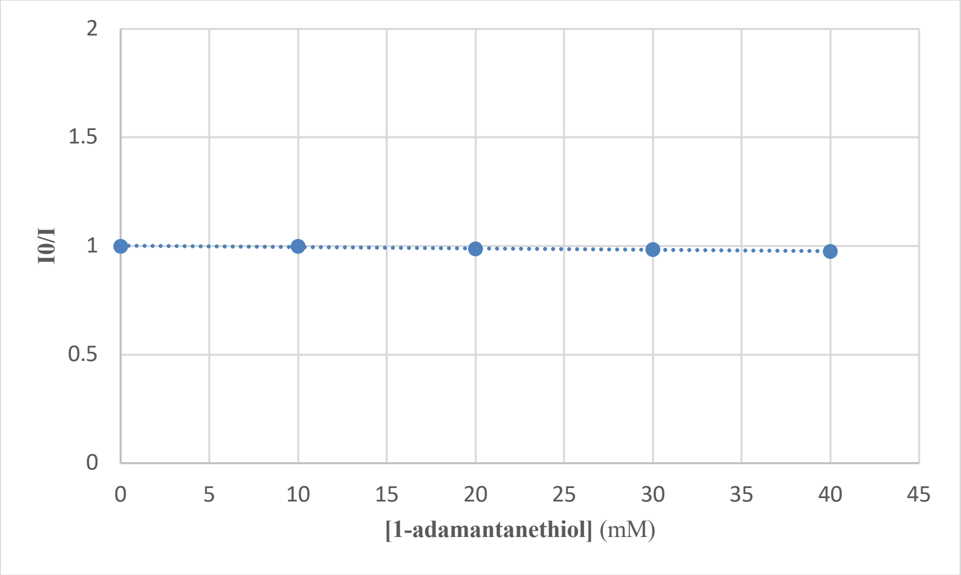

Supplementary Figure 7. Stern-Volmer quenching plot of 1-adamantanethiol.

2-3-4 Electrochemical Measurements

2-3-4-1 Cyclic Voltammogram of Catalyst

Tetrabutylammonium hexafluorophosphate (1161 mg, 3.0 mmol) was added to a 0.01 M solution of the **PO1** anion catalyst (generated in situ by the deprotonation of the **PO1** catalyst with 1.3 equiv Cs_2CO_3) in 30 mL of dry DMSO and the solution was vigorously bubbled with Ar for 5 minutes prior to the measurement. The oxidation/reduction potential was measured using platinum disc working electrode, a

platinum wire counter electrode, and a saturated calomel electrode (SCE) at 0.1 V/s scan rate.

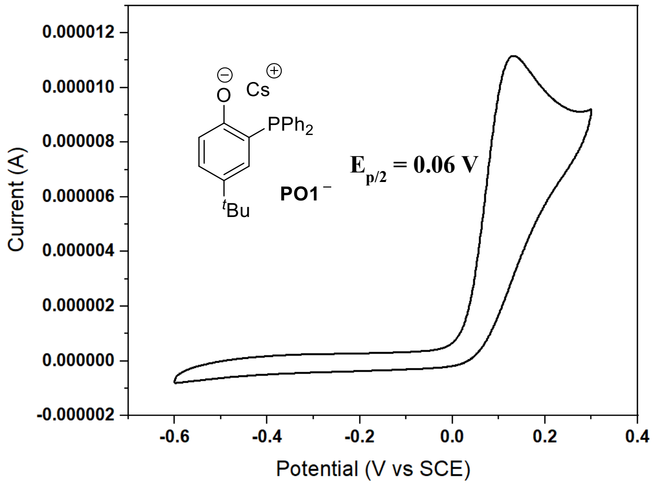

Supplementary Figure 8. The cyclic voltammogram of the **PO1** anions vs SCE in DMSO at 0.1V/s.

With this data in hand we calculated the redox potential of the excited **PO1** anion employing the following equation:[12]

$$E_{p/2}(\mathbf{PO1}^{\cdot}/\ \mathbf{PO1}^{-*}) = E_{p/2}(\mathbf{PO1}^{\cdot}/\ \mathbf{PO1}^{-}) - E_{0\text{-}0}(\mathbf{PO1}^{-*}/\ \mathbf{PO1}^{-})$$

$E_{p/2}$(**PO1**$^{\cdot}$/ **PO1**$^{-}$) = 0.06 V vs. SCE, In the absence of vibrational structures, $E_{0\text{-}0}$ can be roughly estimated from the absorption spectrum.[13] This corresponds to 420 nm, which translates into an $E_{0\text{-}0}$(**PO1**$^{-*}$/ **PO1**$^{-}$) of 2.95 eV for the **PO1** anion.

$E_{p/2}$(**PO1**$^{\cdot}$/ **PO1**$^{-*}$) = $E_{p/2}$(**PO1**$^{\cdot}$/ **PO1**$^{-}$) – $E_{0\text{-}0}$(**PO1**$^{-*}$/ **PO1**$^{-}$) = 0.06 – 2.95 = – 2.89 V vs. SCE

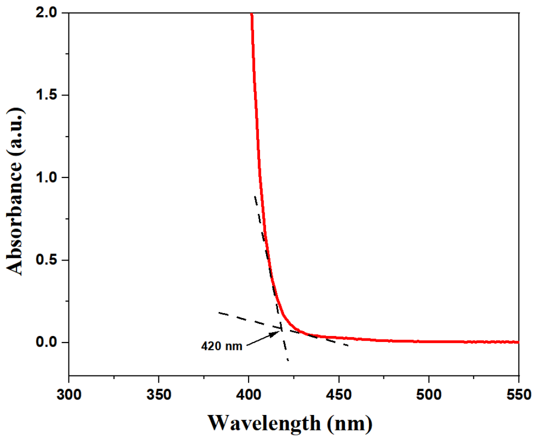

2-3-4-2 Cyclic Voltammogram of Substrate

Electrochemical potentials were obtained with a standard set of conditions to main internal consistency. Samples were prepared with 0.3 mmol of substrate in 30 mL of 0.1 M tetrabutylammonium hexafluorophosphate in dry, degassed acetonitrile. Measurements employed a glassy carbon working electrode, platinum wire counter electrode, saturated calomel reference electrode, and a scan rate of 0.1 V/s. Reductions were measured by scanning potentials in the negative direction; the glassy carbon electrode was polished between each scan.

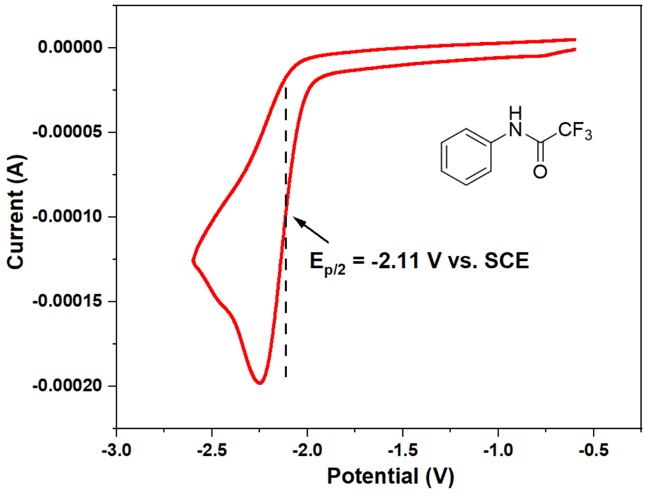

Supplementary Figure 9. Cyclic voltammogram of 2,2,2-trifluoro-*N*-phenylacetamide.

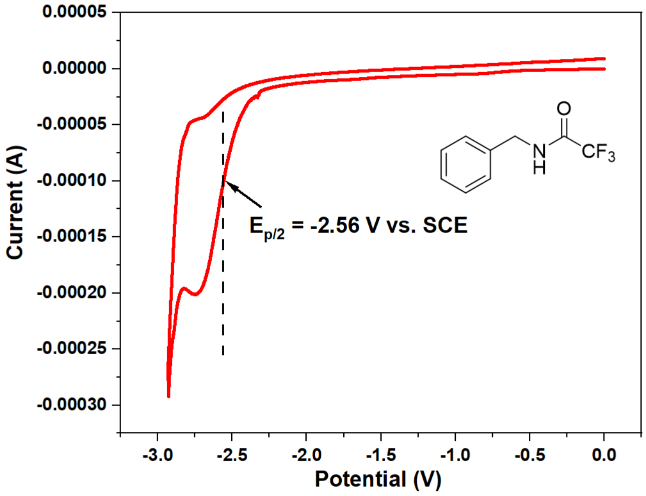

Supplementary Figure 10. Cyclic voltammogram of *N*-benzyl-2,2,2-trifluoroacetamide.

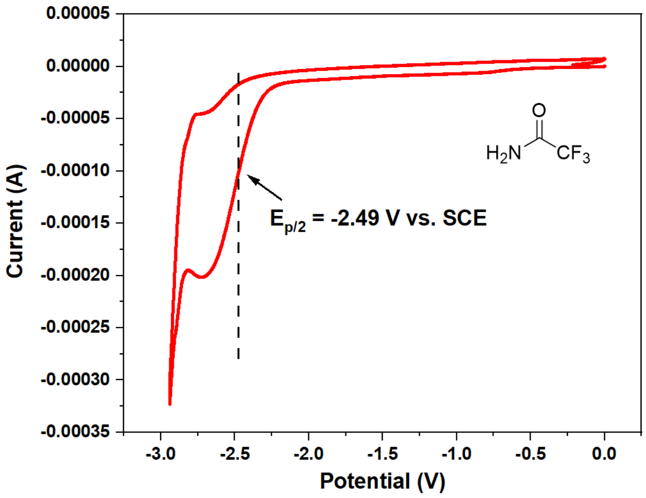

Supplementary Figure 11. Cyclic voltammogram of 2,2,2-trifluoroacetamide.

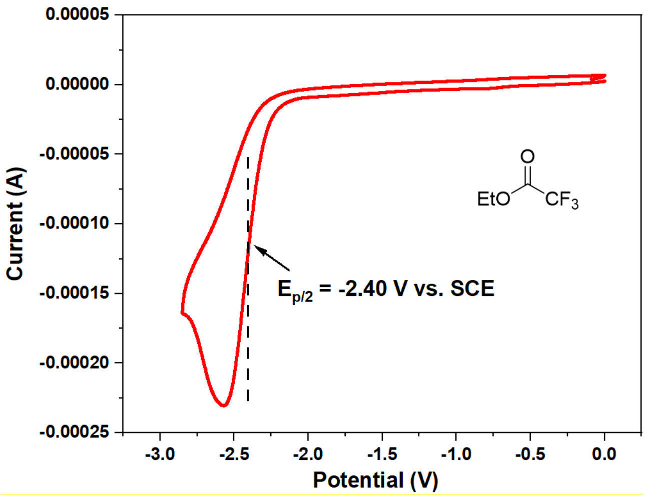

Supplementary Figure 12. Cyclic voltammogram of ethyl 2,2,2-trifluoroacetate.

2-4 ^{1}H-NMR, ^{13}C-NMR, and ^{19}F-NMR Spectra

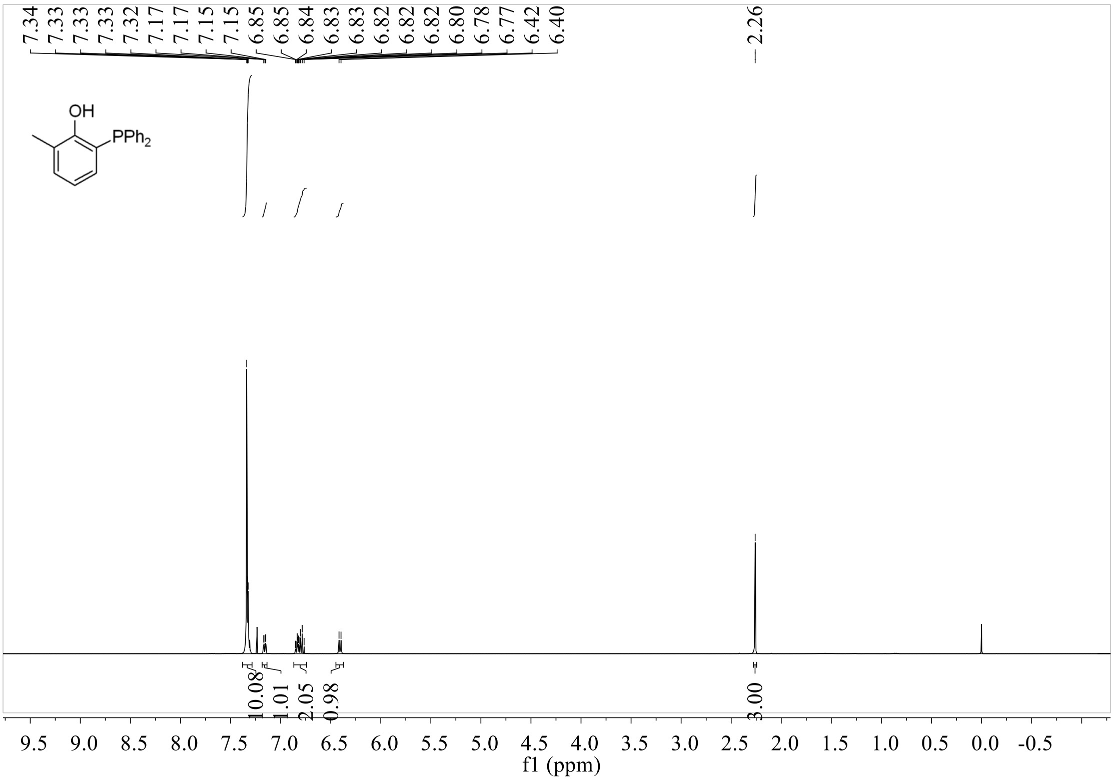

Supplementary Figure 13. ^{1}H NMR (400 MHz, $CDCl_3$) spectrum of **PO2**

^{13}C NMR (101 MHz, $CDCl_3$) spectrum of **2-(diphenylphosphaneyl)-6-methylphenol (PO2)**

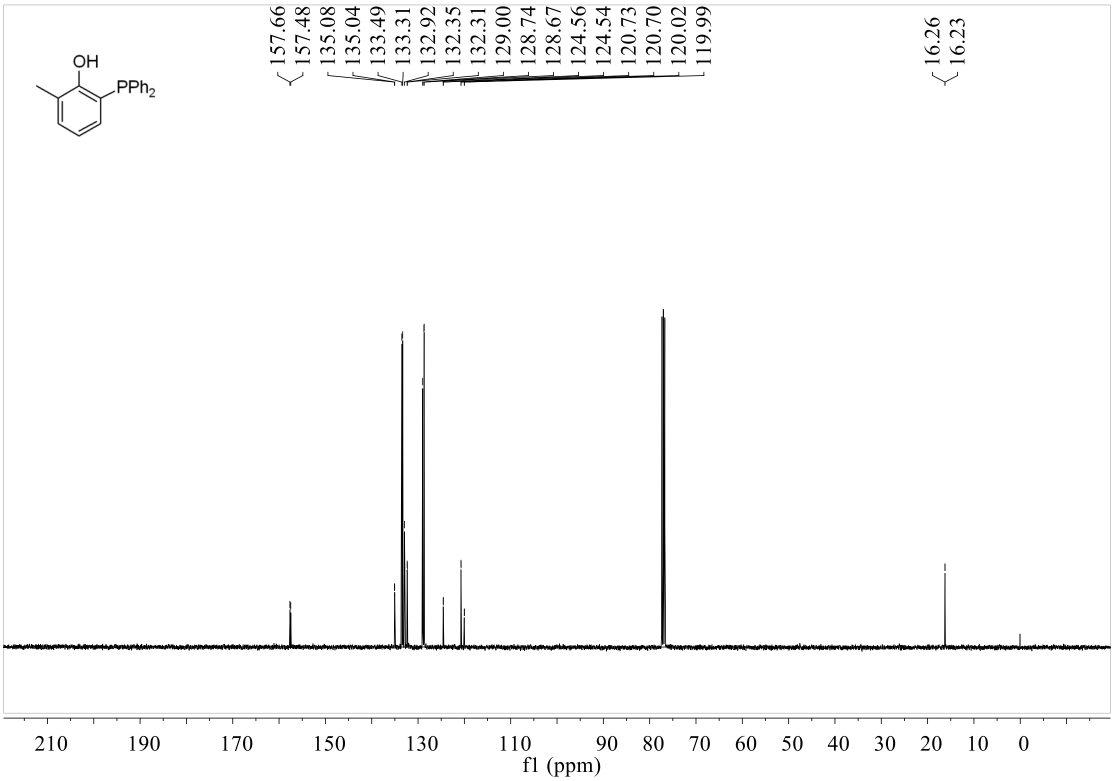

Supplementary Figure 14. ^{13}C NMR (101 MHz, $CDCl_3$) spectrum of **PO2**

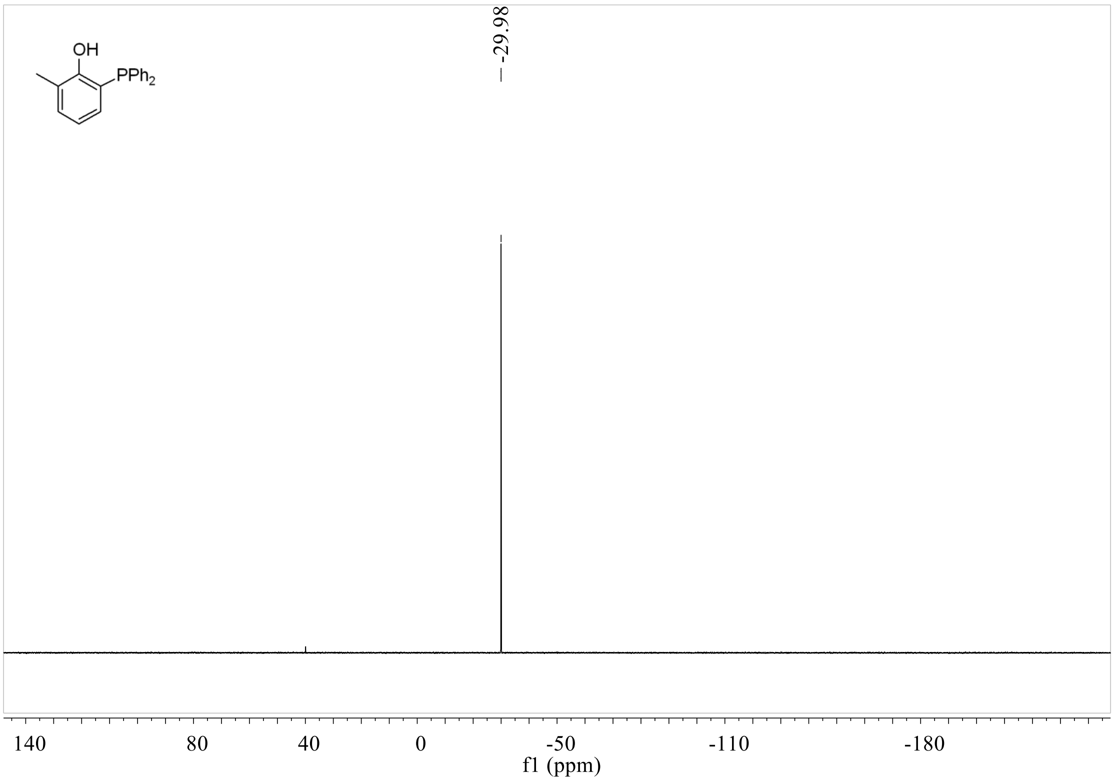

Supplementary Figure 15. ^{31}P NMR (162 MHz, $CDCl_3$) spectrum of **PO2**

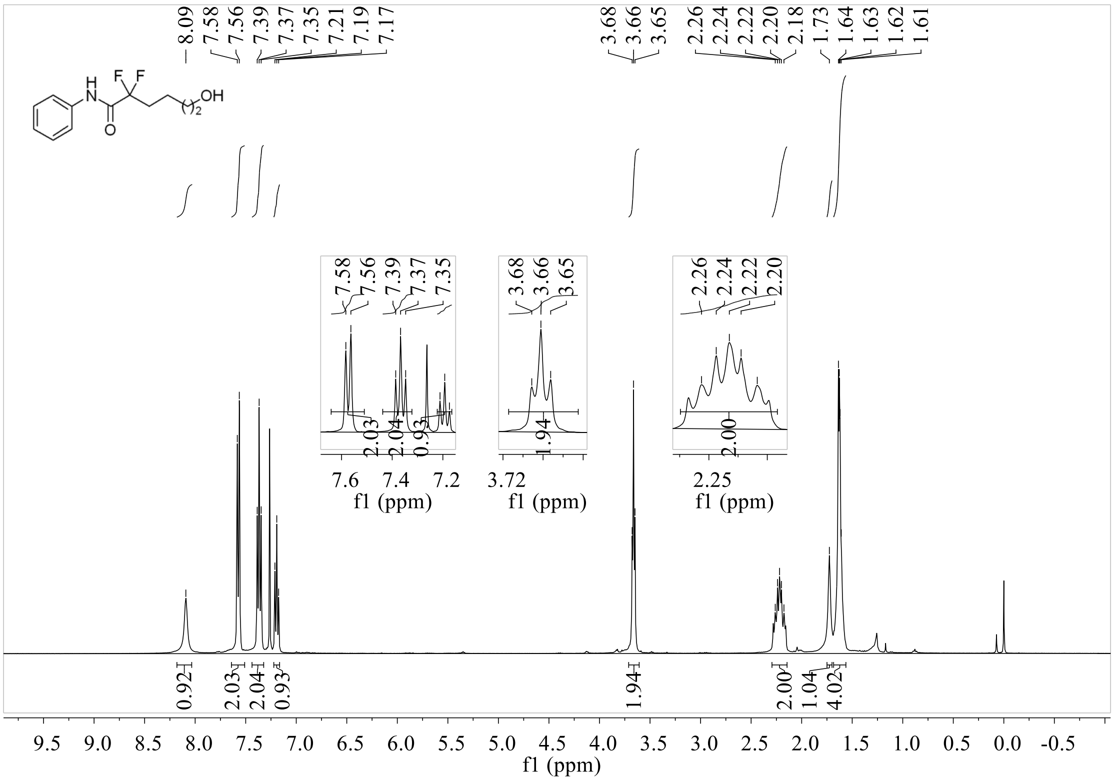

Supplementary Figure 16. ^{1}H NMR (400 MHz, $CDCl_3$) spectrum of **3**

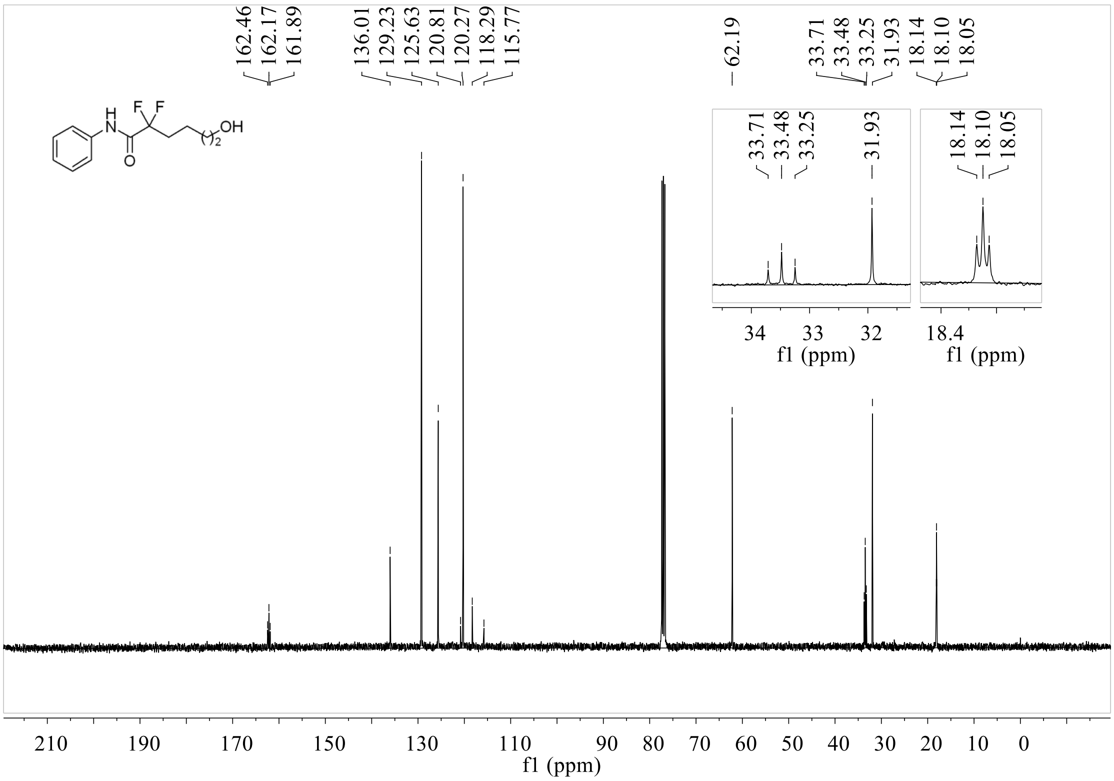

Supplementary Figure 17. ^{13}C NMR (101 MHz, $CDCl_3$) spectrum of **3**

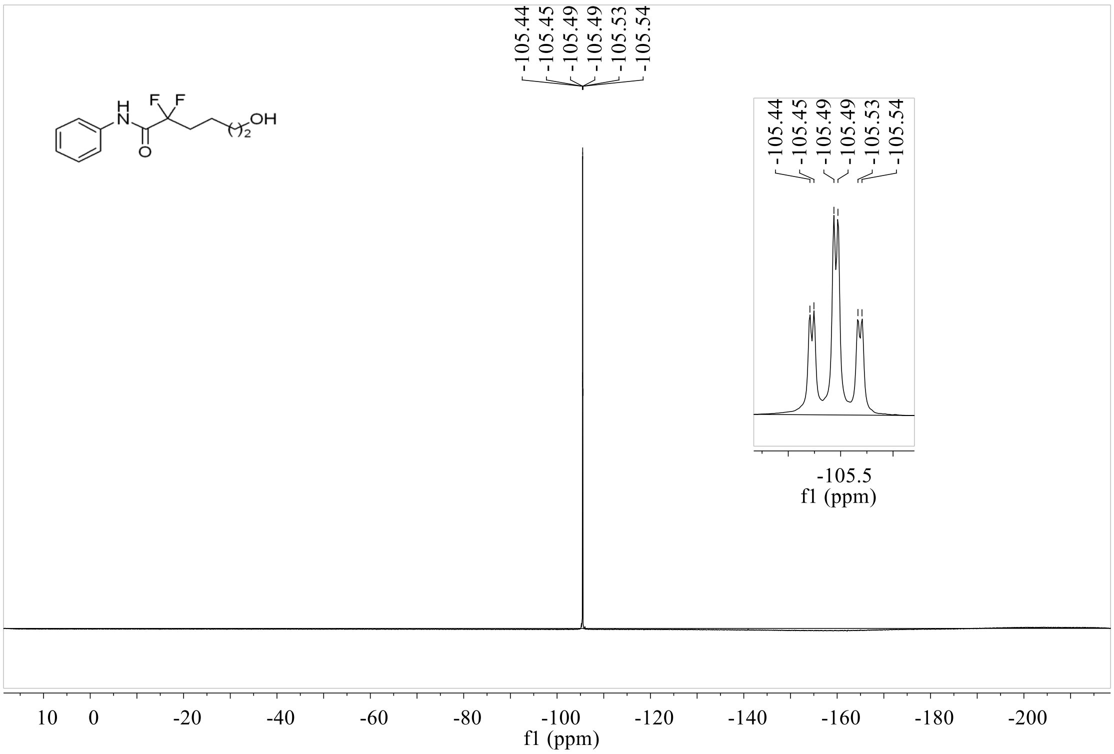

Supplementary Figure 18. ^{19}F NMR (376 MHz, $CDCl_3$) spectrum of **3**

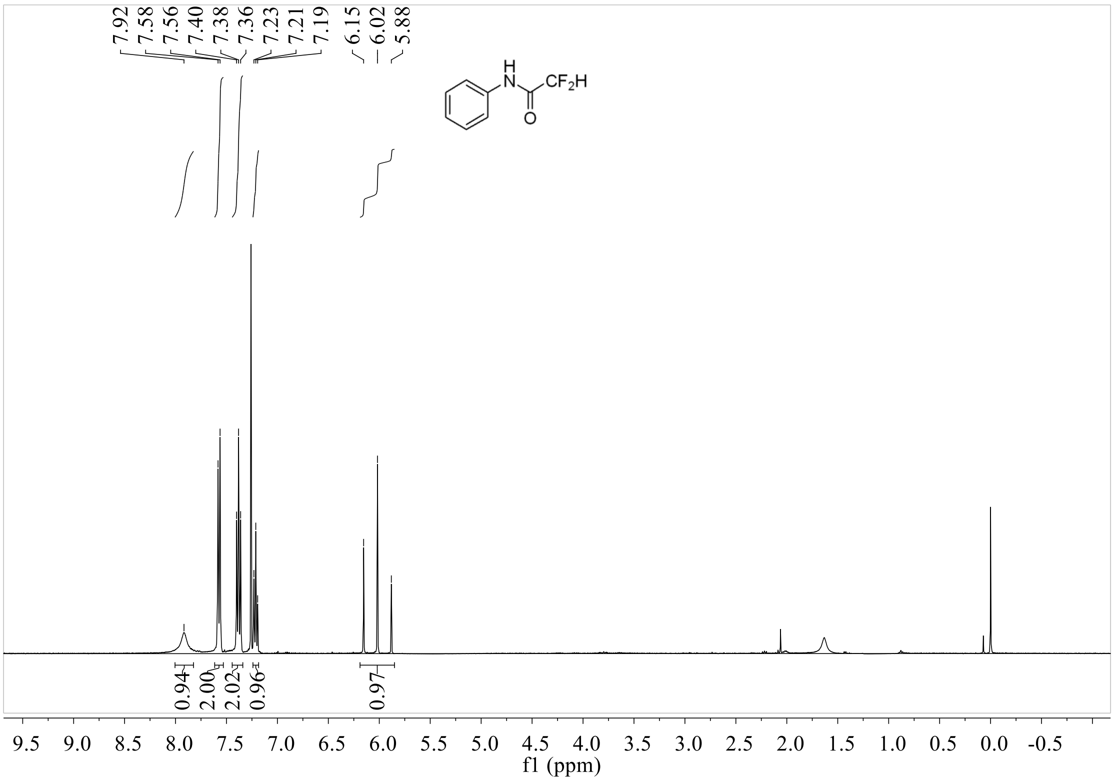

Supplementary Figure 19. ^{1}H NMR (400 MHz, $CDCl_3$) spectrum of **4**

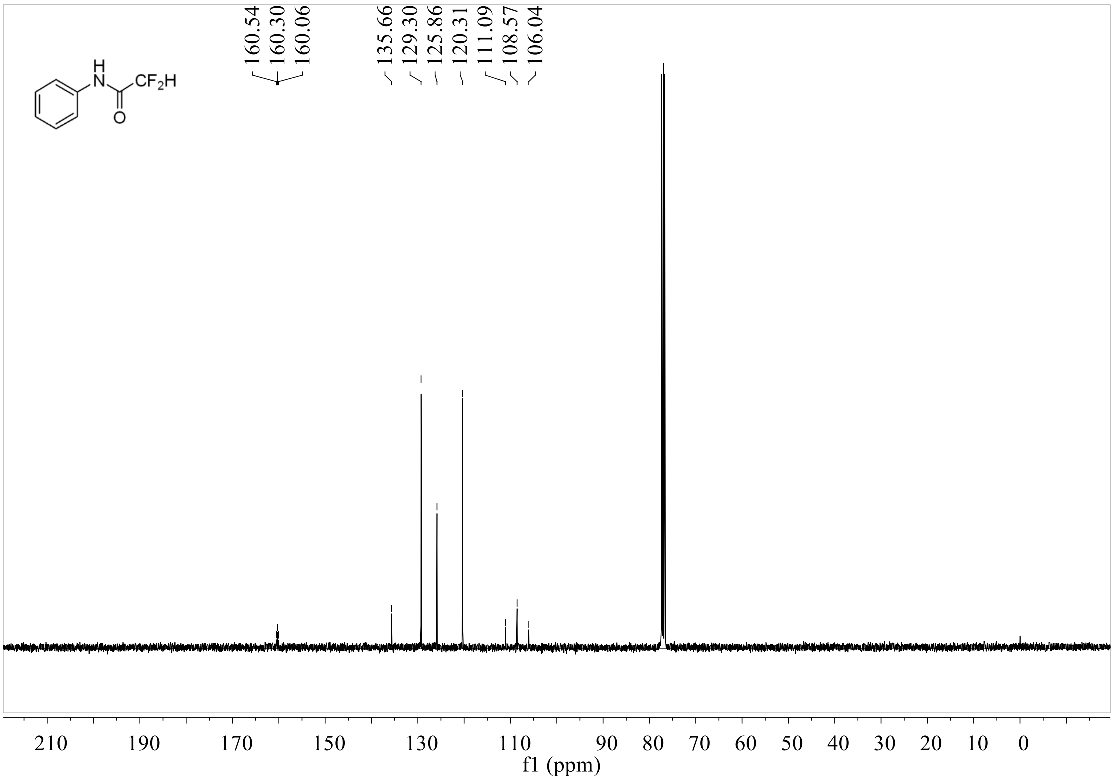

Supplementary Figure 20. ^{13}C NMR (101 MHz, $CDCl_3$) spectrum of **4**

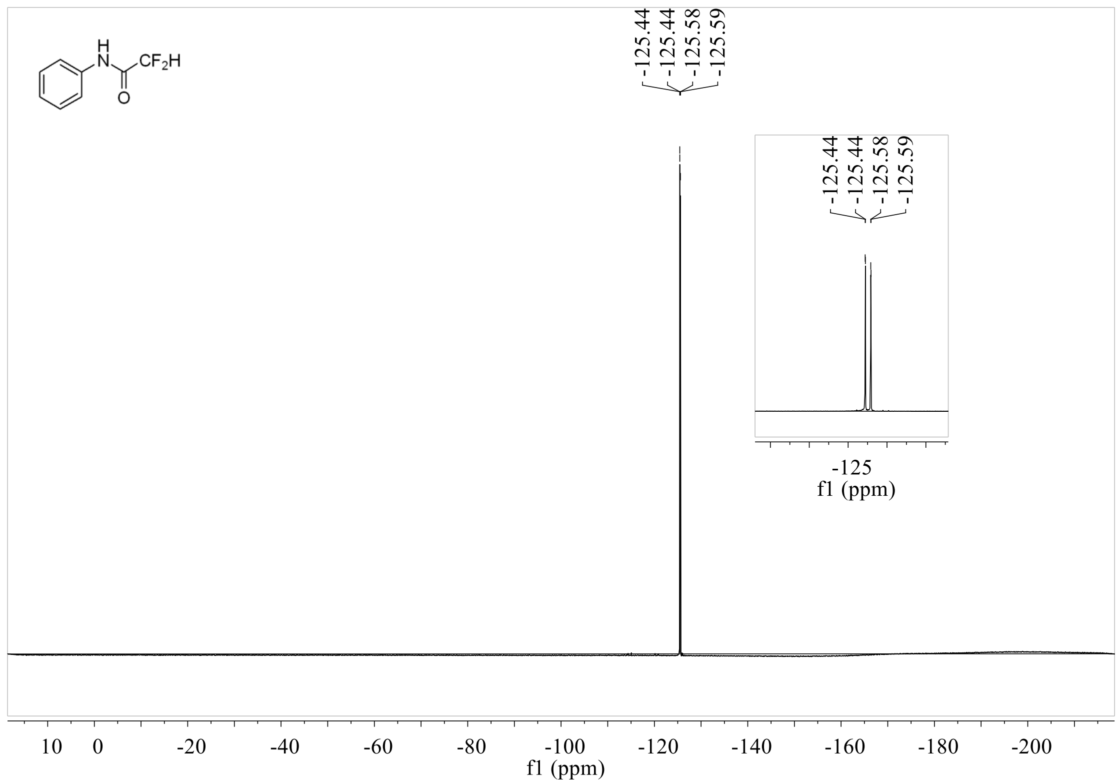

Supplementary Figure 21. ^{19}F NMR (101 MHz, $CDCl_3$) spectrum of **4**

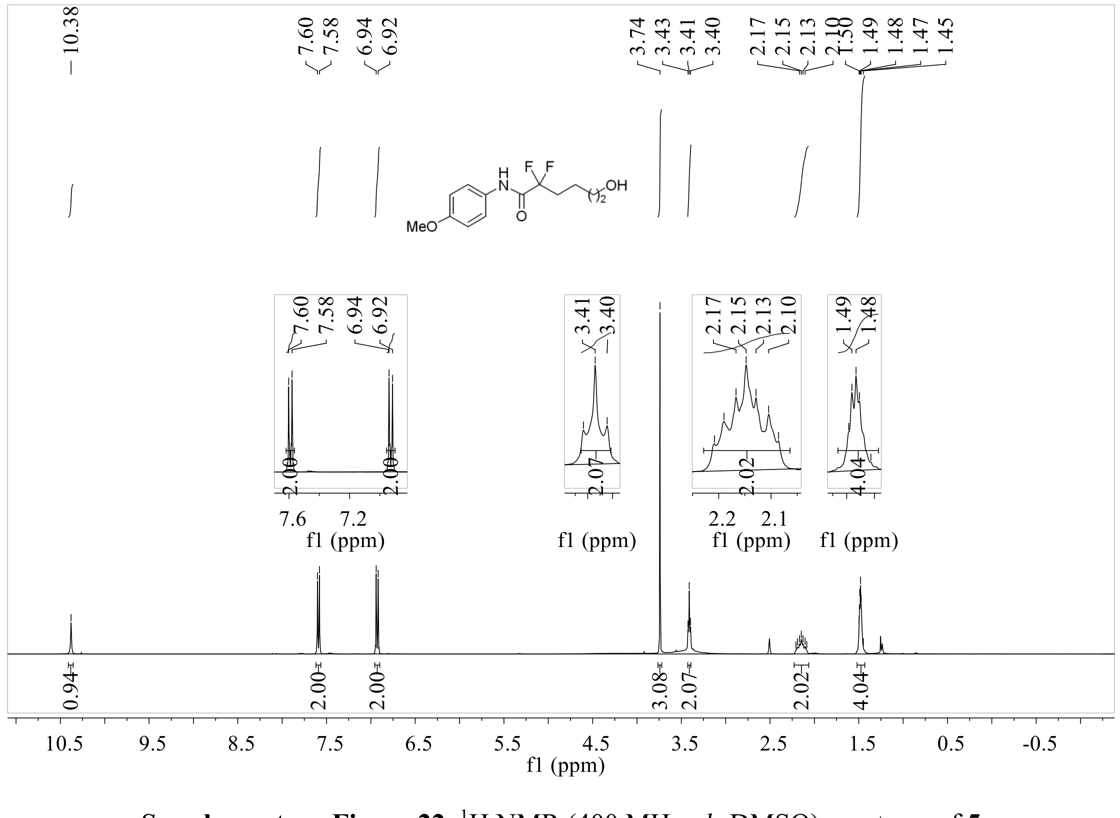

Supplementary Figure 22. ^{1}H NMR (400 MHz, d_6-DMSO) spectrum of **5**

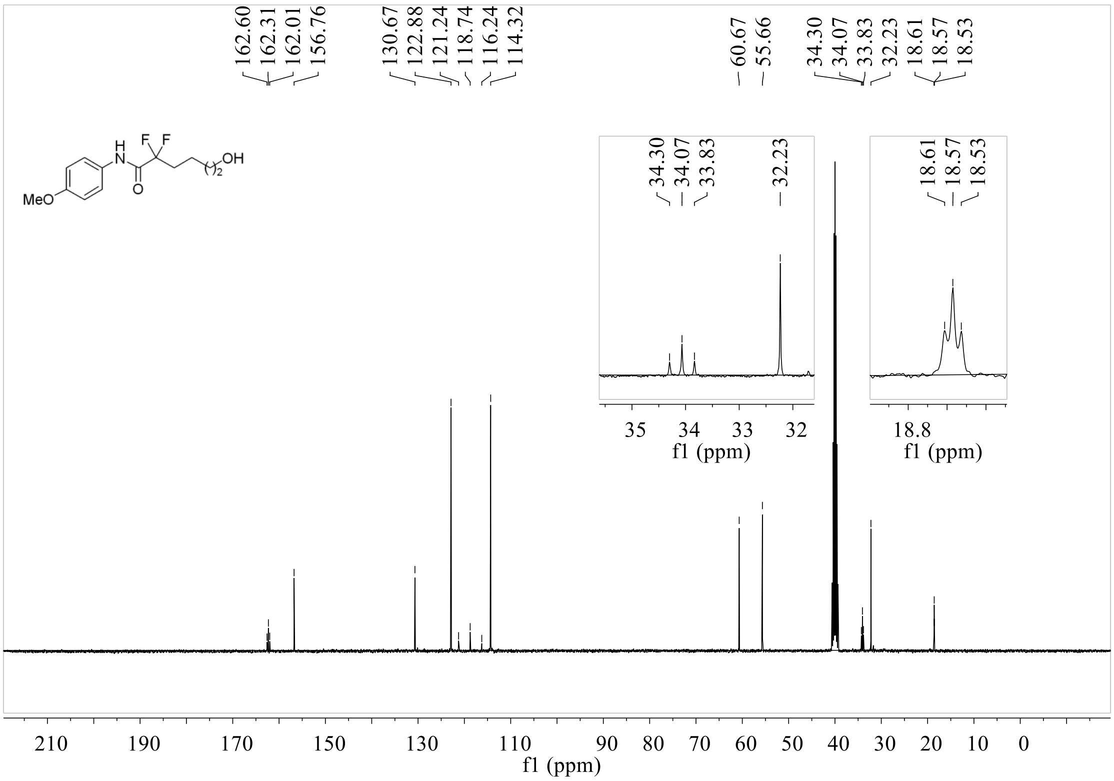

Supplementary Figure 23. ^{13}C NMR (101 MHz, d_6-DMSO) spectrum of **5**

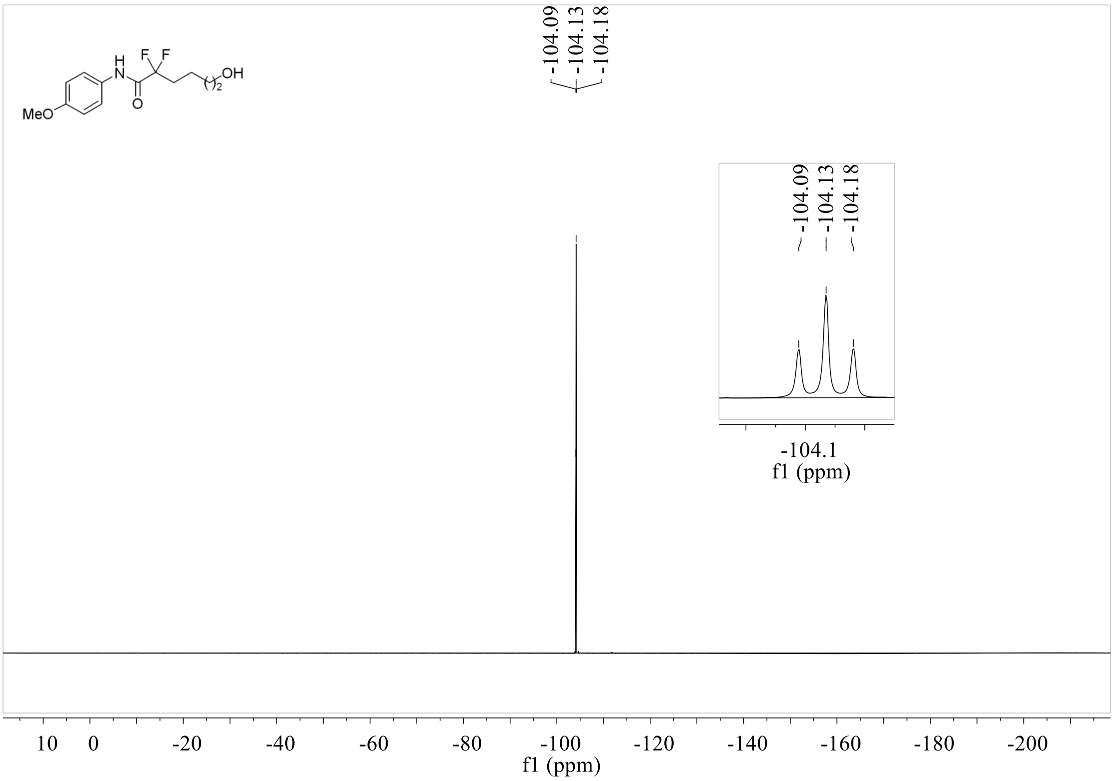

Supplementary Figure 24. ^{19}F NMR (376 MHz, d_6-DMSO) spectrum of **5**

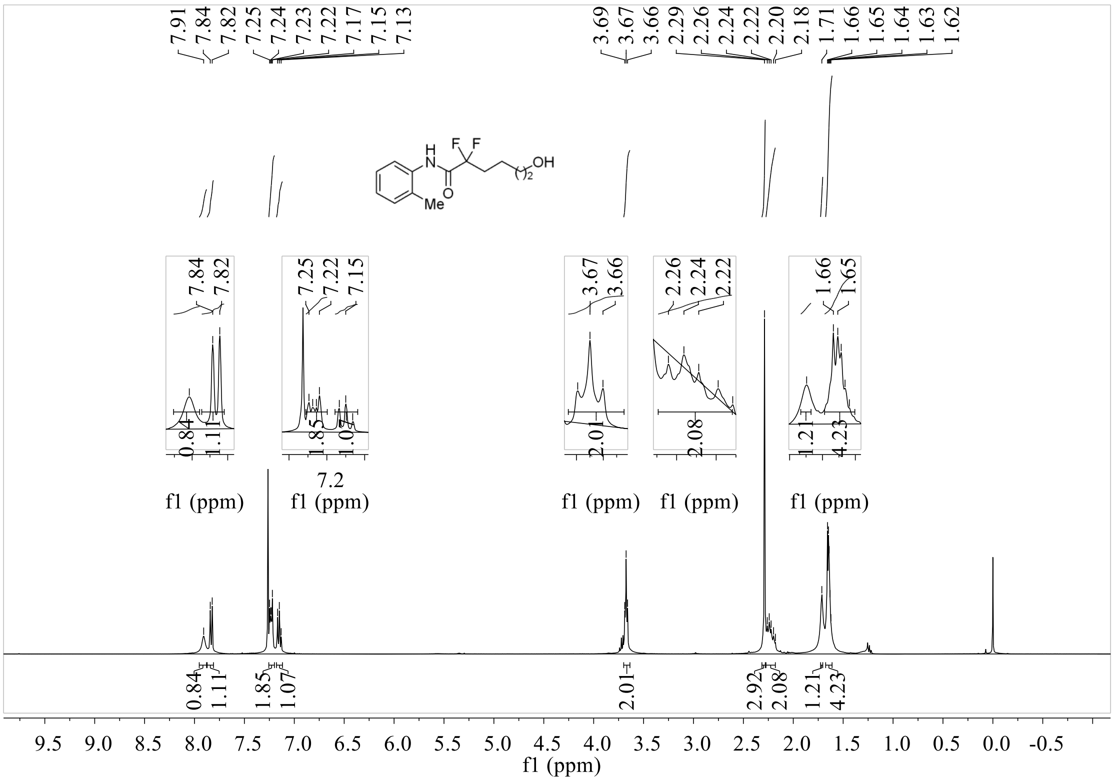

Supplementary Figure 25. ^{1}H NMR (400 MHz, $CDCl_3$) spectrum of **6**

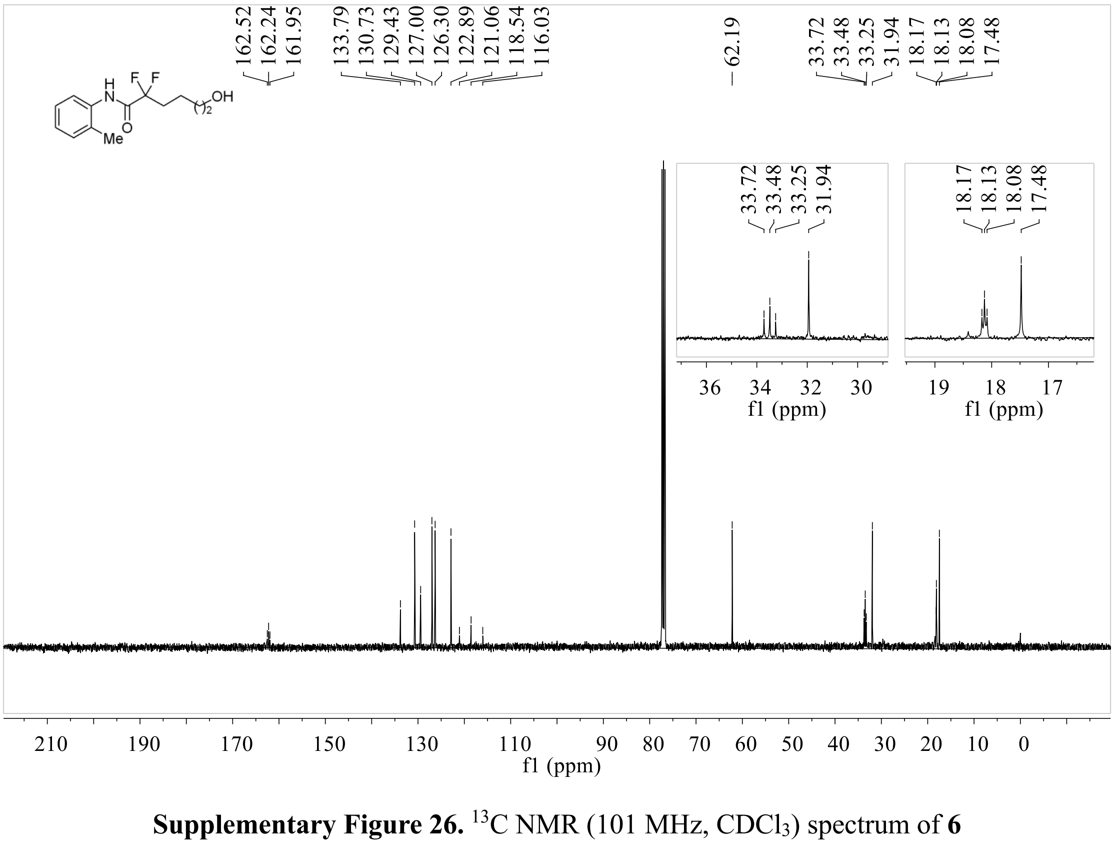

Supplementary Figure 26. ^{13}C NMR (101 MHz, $CDCl_3$) spectrum of **6**

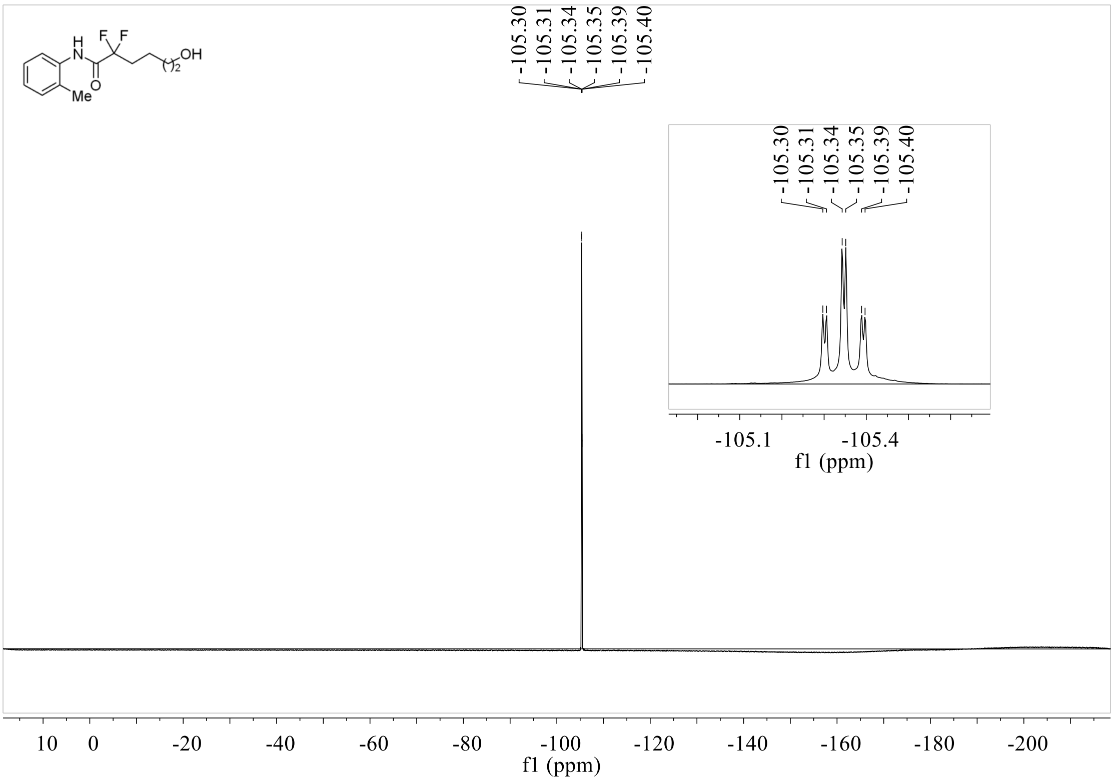

Supplementary Figure 27. ^{19}F NMR (376 MHz, $CDCl_3$) spectrum of **6**

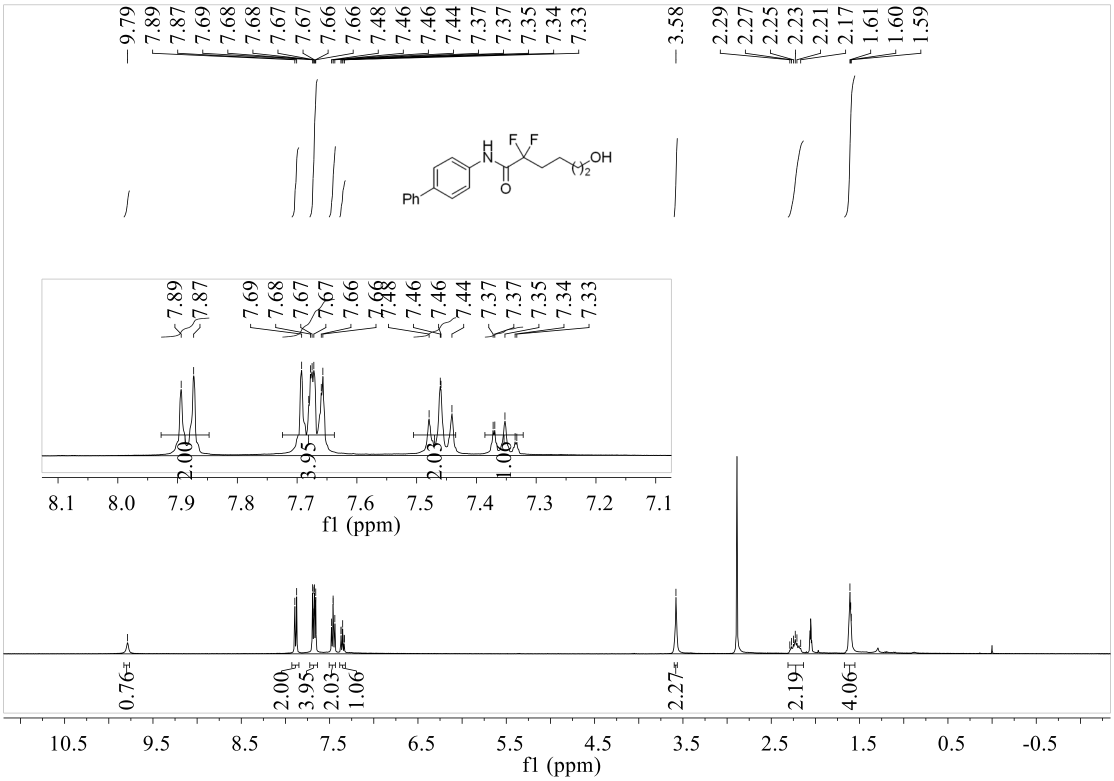

Supplementary Figure 28. ^{1}H NMR (400 MHz, Acetone-d_6) spectrum of **7**

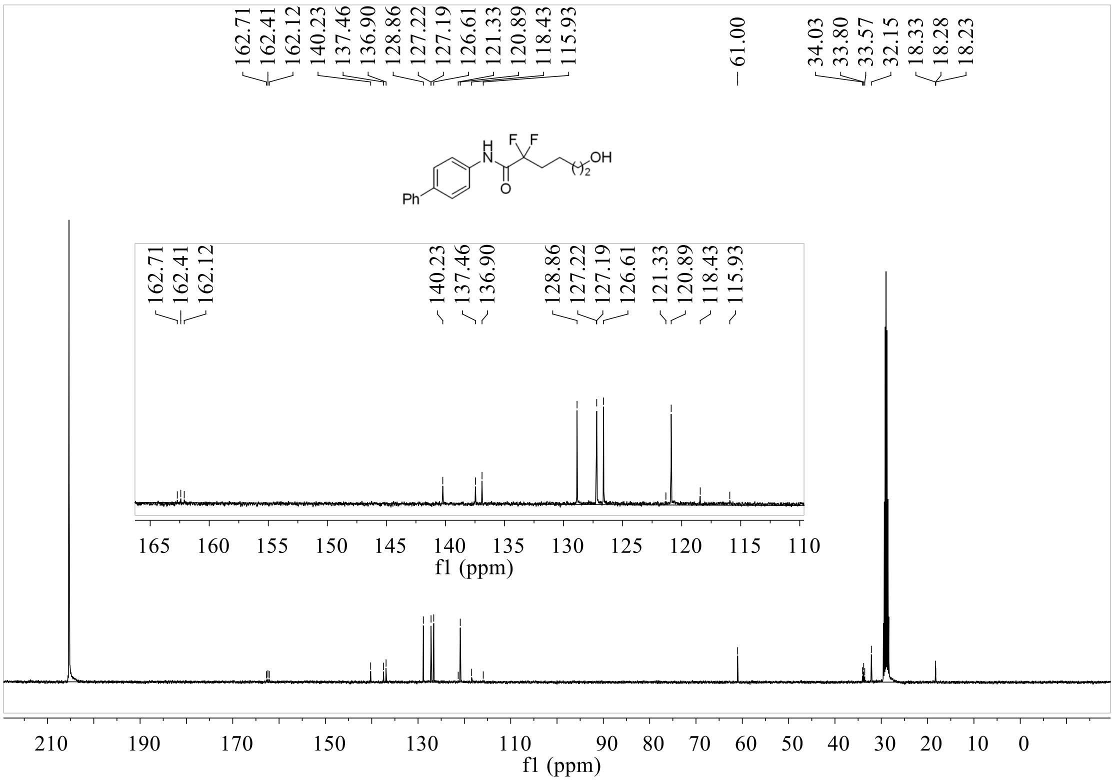

Supplementary Figure 29. ^{13}C NMR (101 MHz, Acetone-d_6) spectrum of **7**

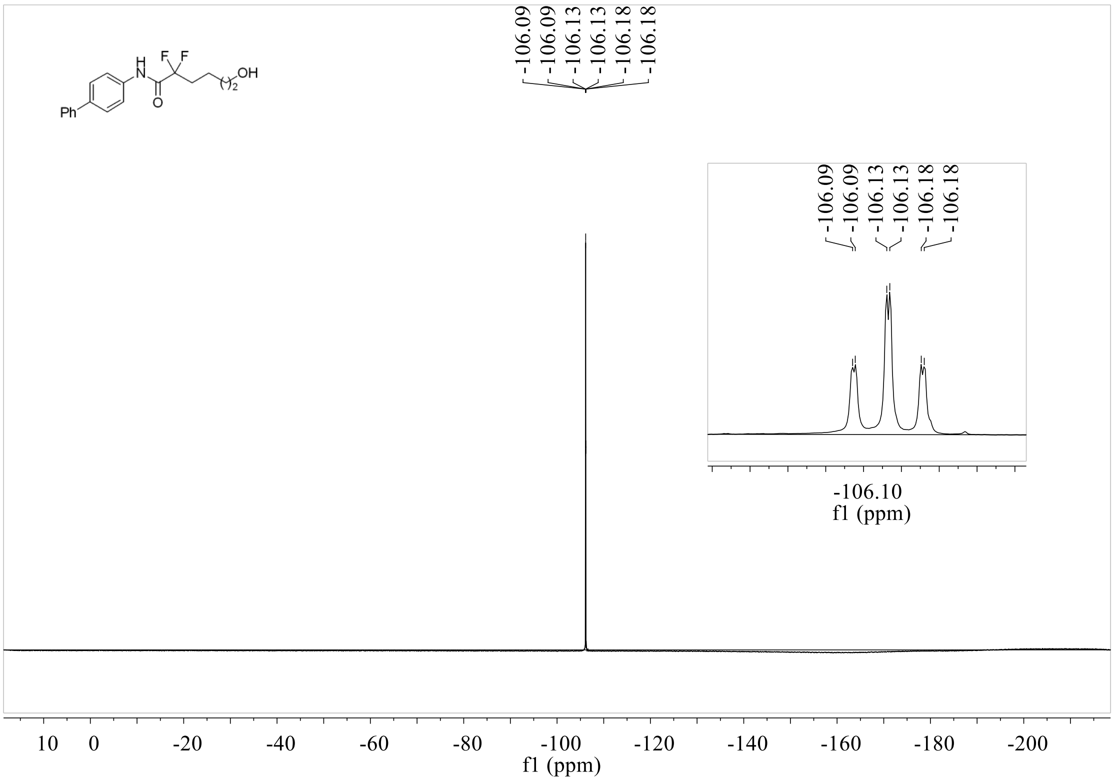

Supplementary Figure 30. ^{19}F NMR (376 MHz, Acetone-d_6) spectrum of **7**

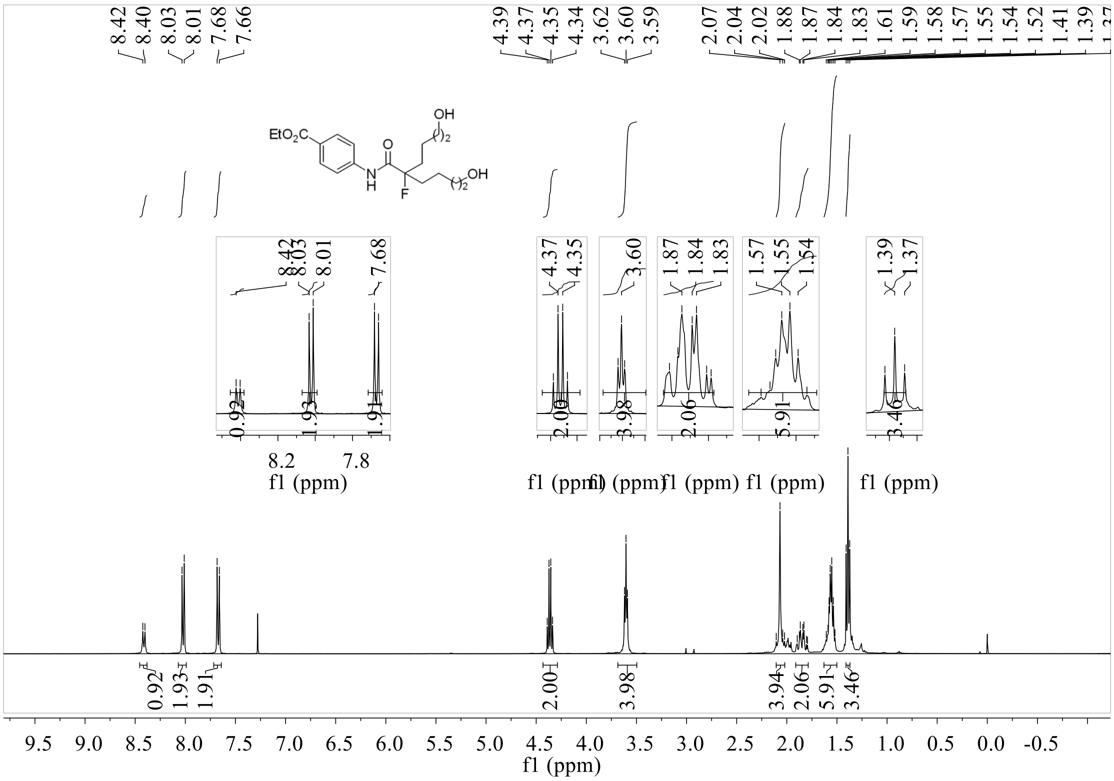

Supplementary Figure 31. ^{1}H NMR (400 MHz, $CDCl_3$) spectrum of **8**

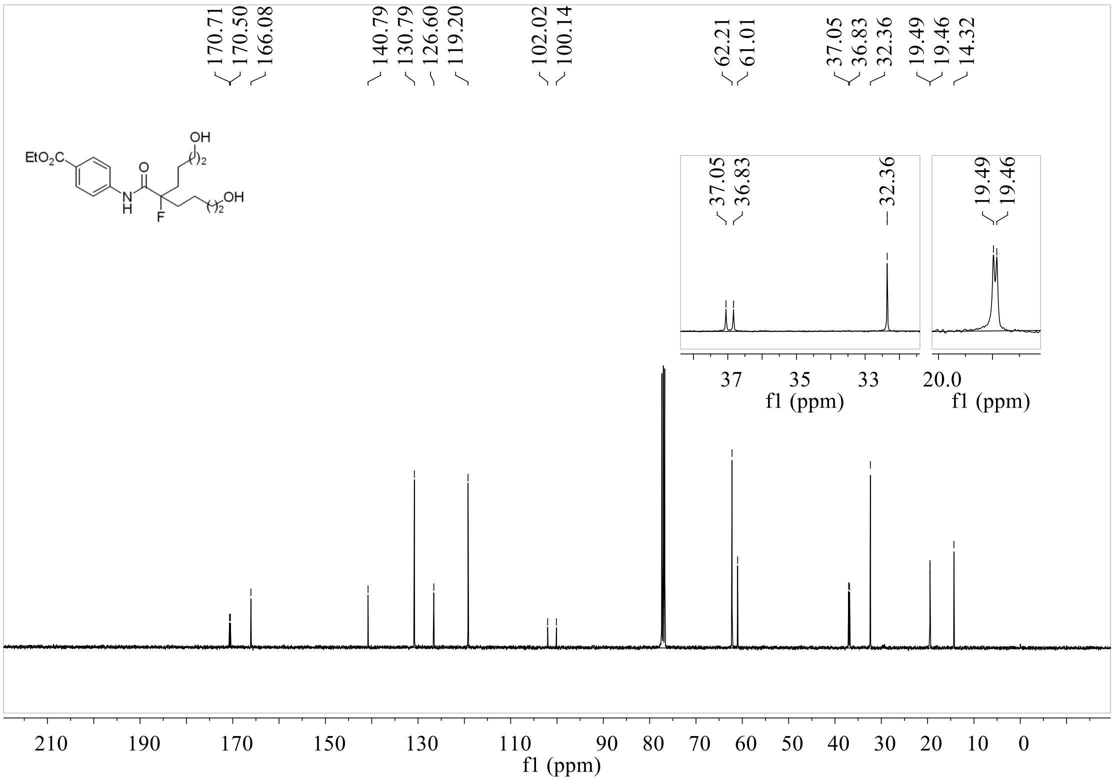

Supplementary Figure 32. ^{13}C NMR (101 MHz, $CDCl_3$) spectrum of **8**

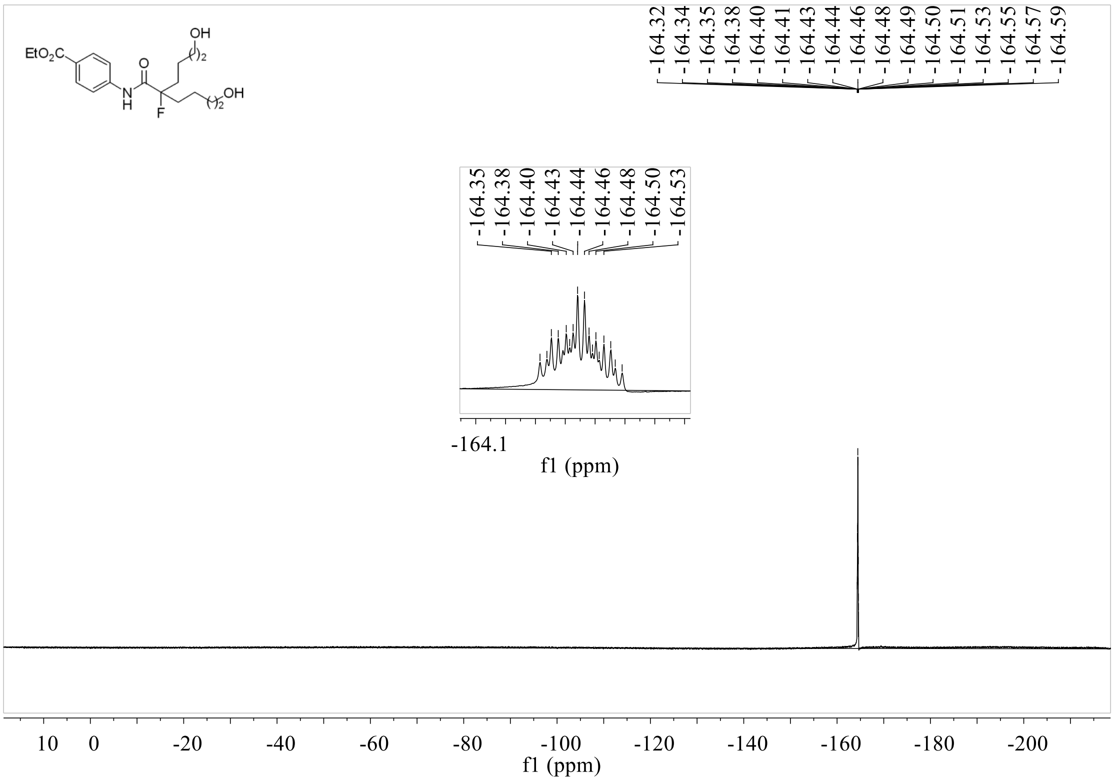

Supplementary Figure 33. ^{19}F NMR (376 MHz, $CDCl_3$) spectrum of **8**

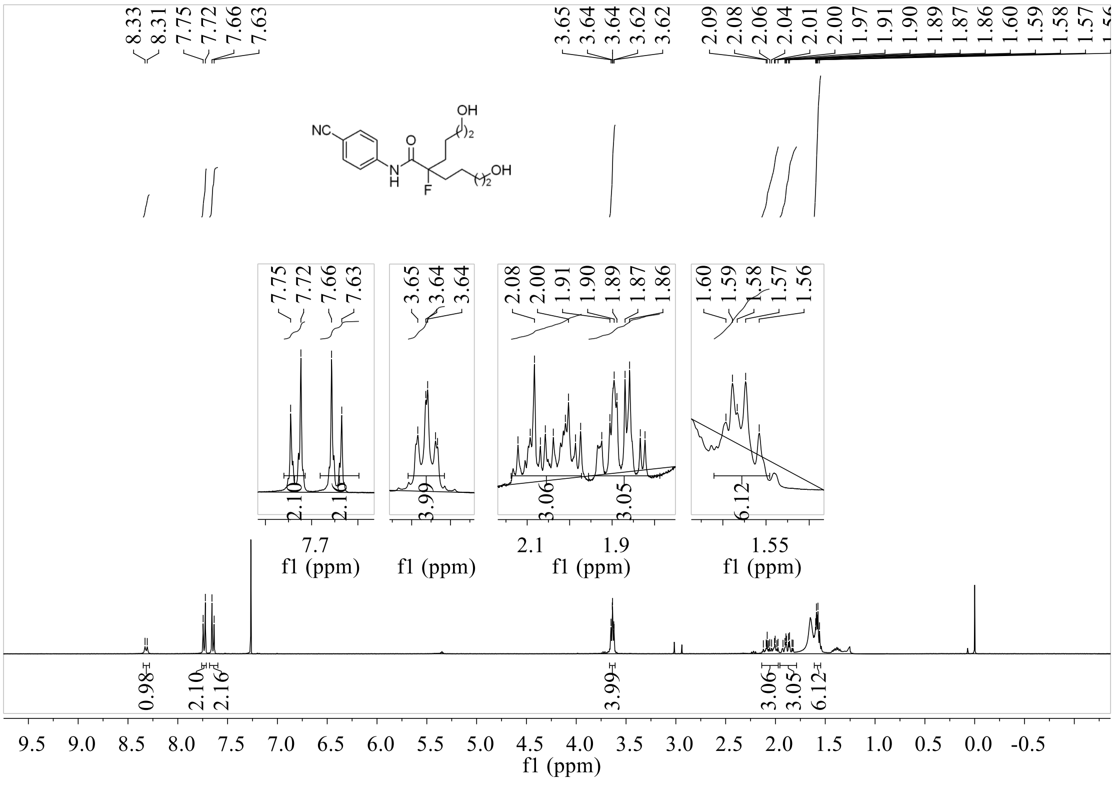

Supplementary Figure 34. ^{1}H NMR (400 MHz, $CDCl_3$) spectrum of **9**

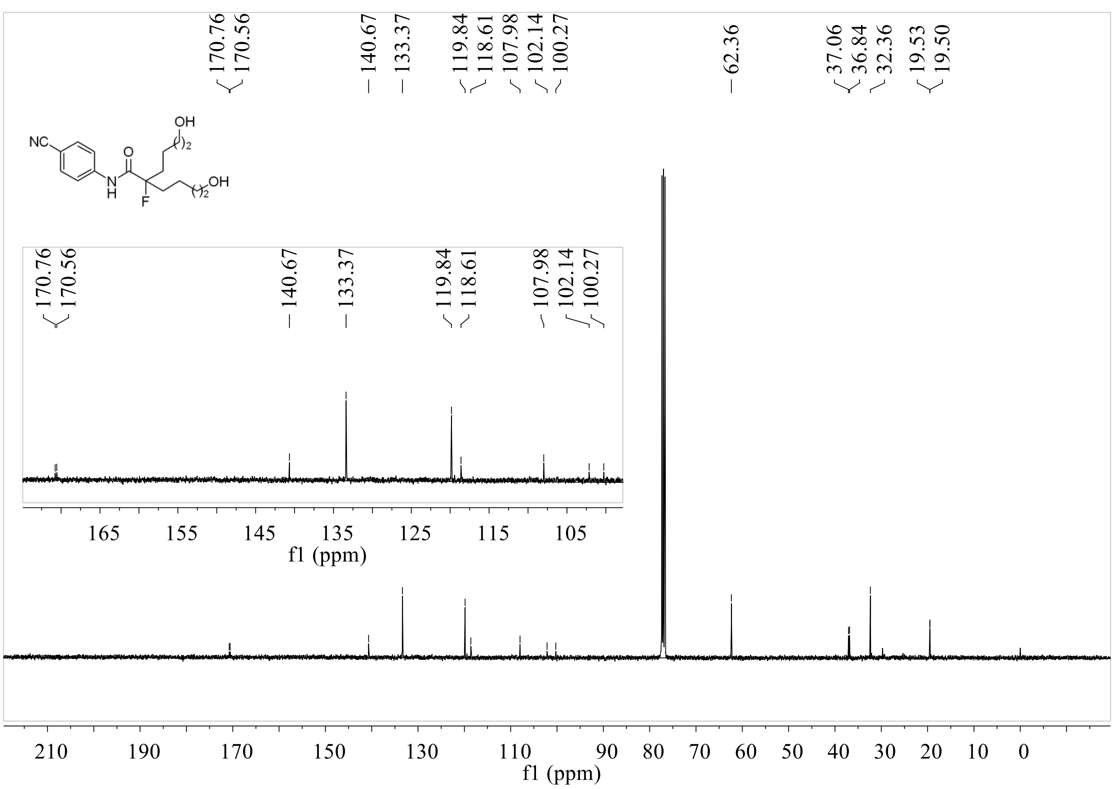

Supplementary Figure 35. ^{13}C NMR (101 MHz, $CDCl_3$) spectrum of **9**

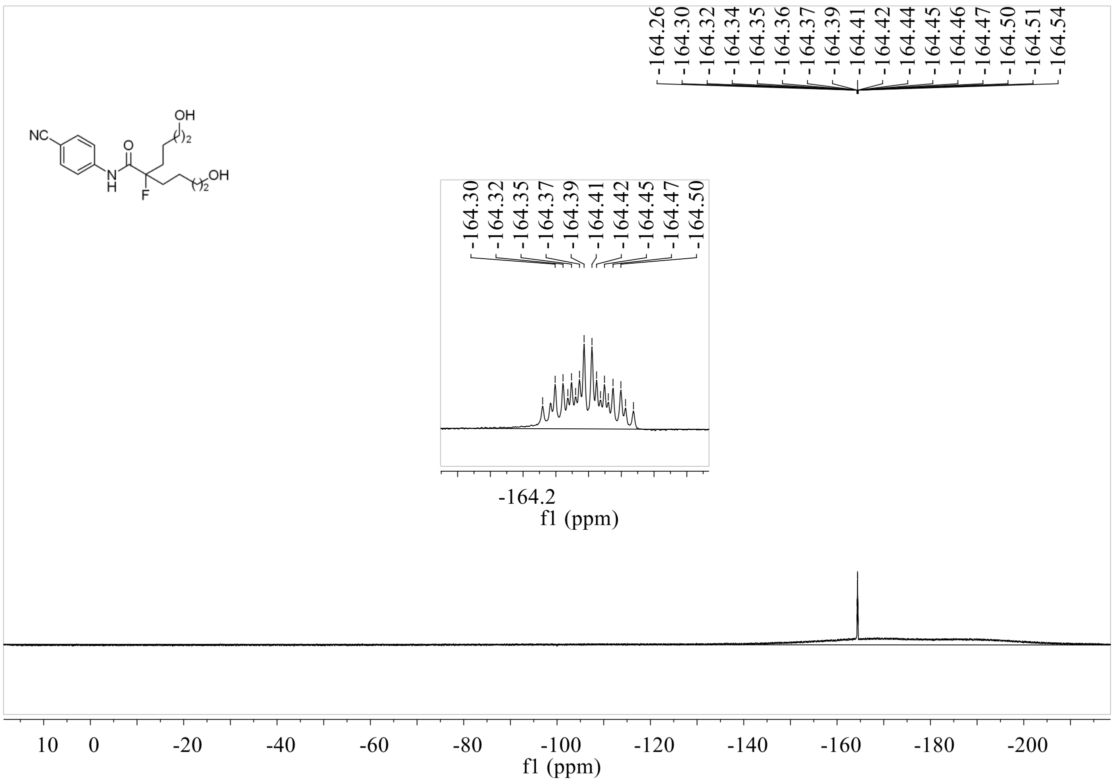

Supplementary Figure 36. ^{19}F NMR (376 MHz, $CDCl_3$) spectrum of **9**

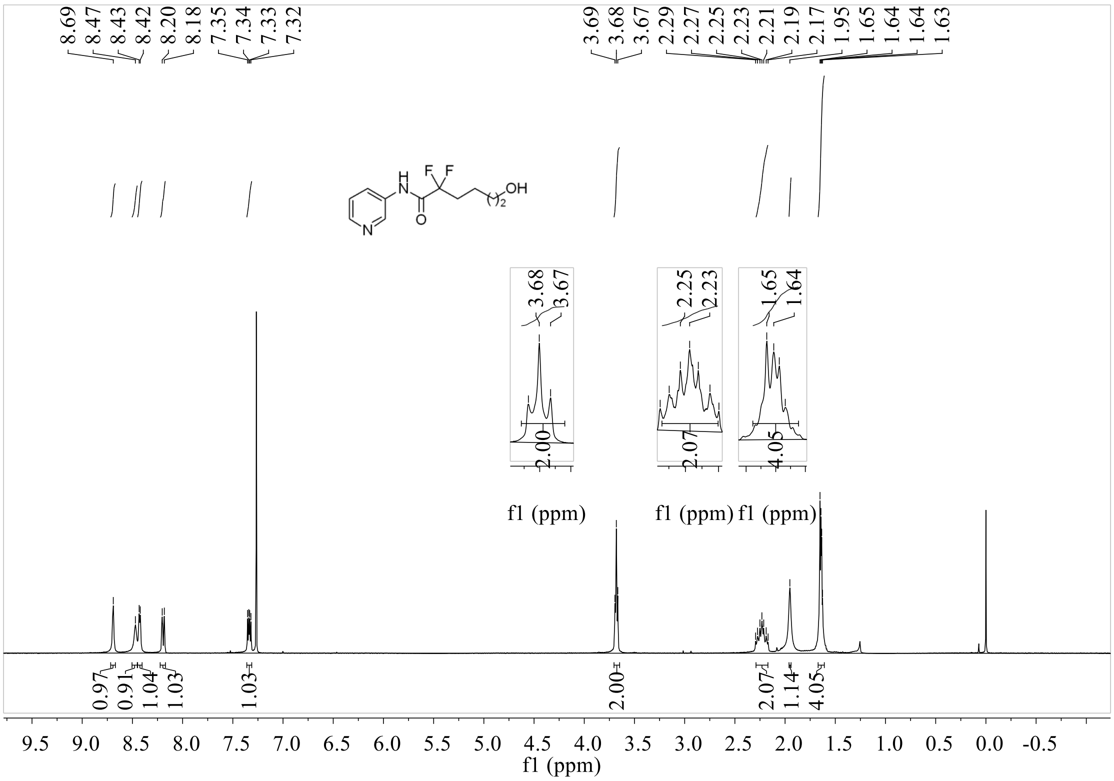

Supplementary Figure 37. ^{1}H NMR (400 MHz, $CDCl_3$) spectrum of **10**

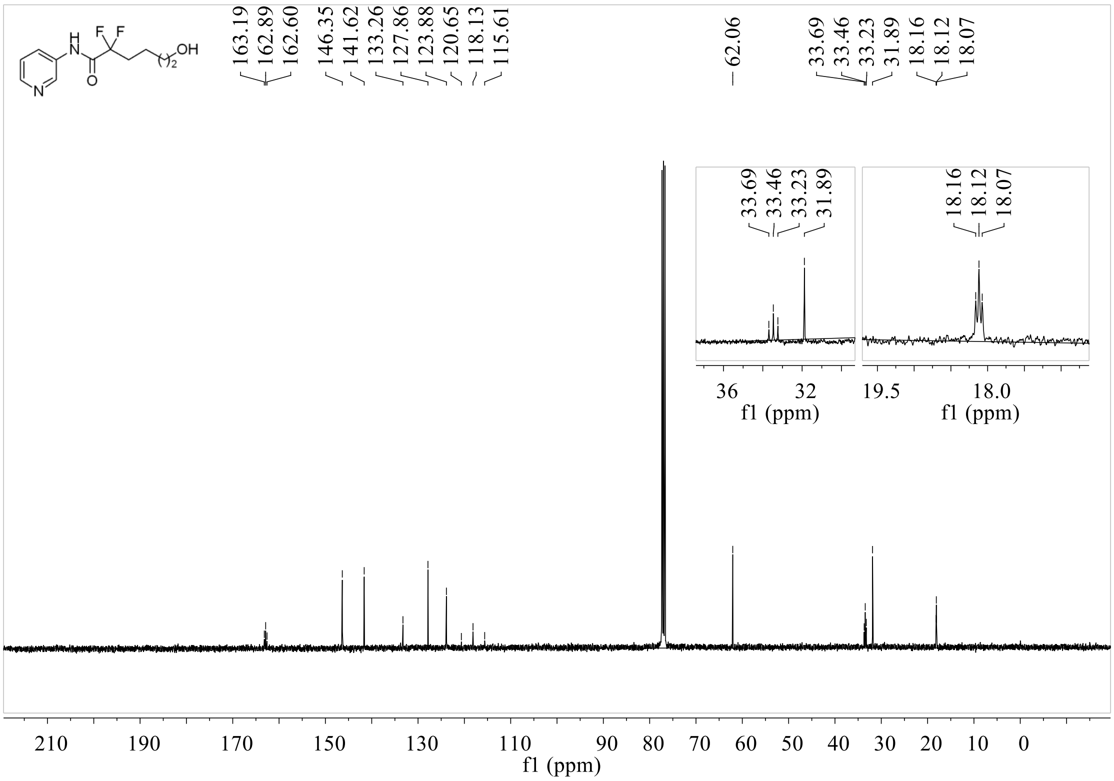

Supplementary Figure 38. ^{13}C NMR (101 MHz, $CDCl_3$) spectrum of **10**

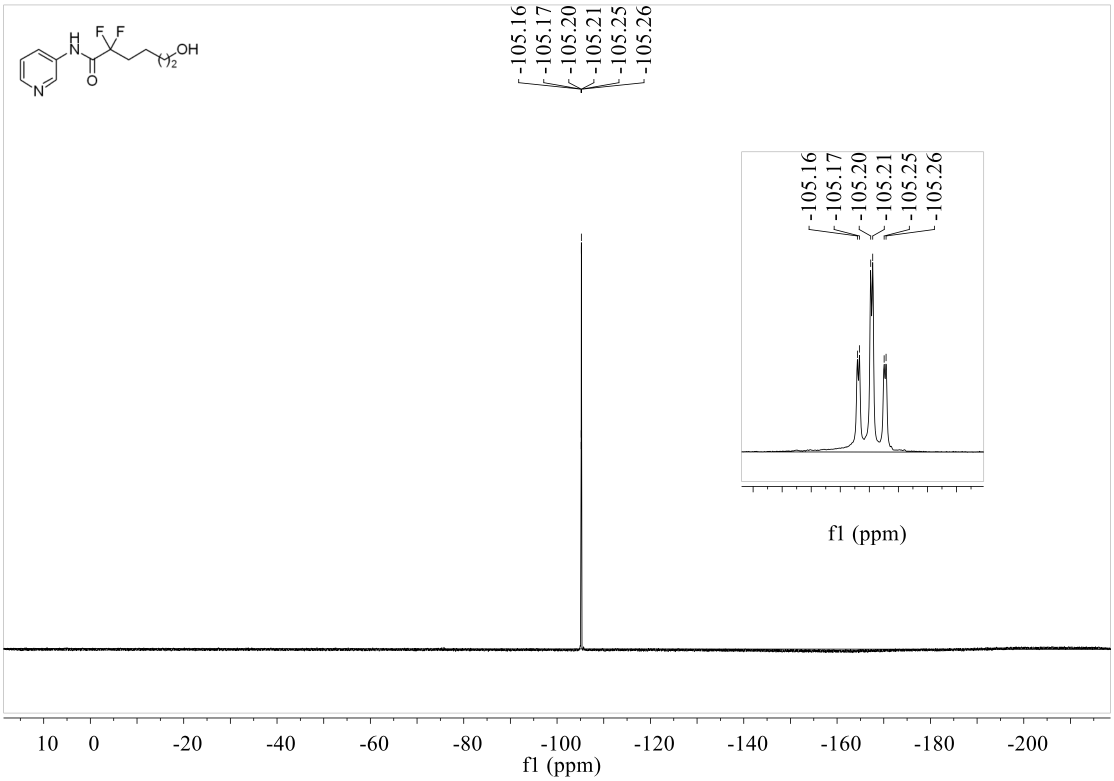

Supplementary Figure 39. ^{19}F NMR (376 MHz, $CDCl_3$) spectrum of **10**

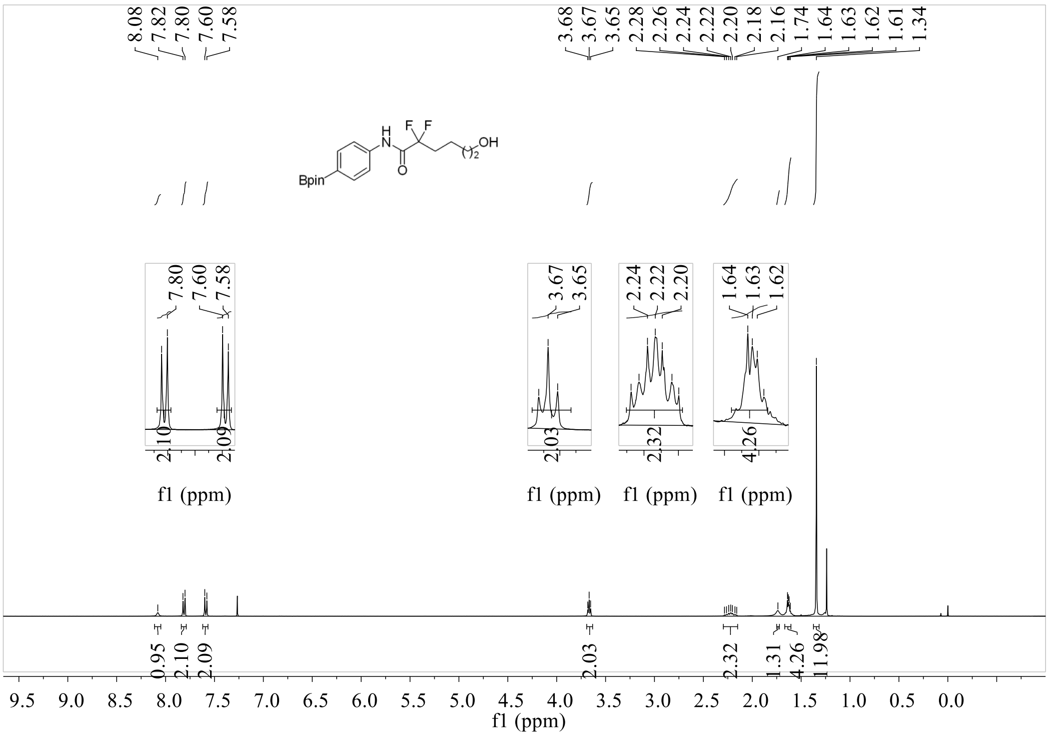

Supplementary Figure 40. ^{1}H NMR (400 MHz, $CDCl_3$) spectrum of **11**

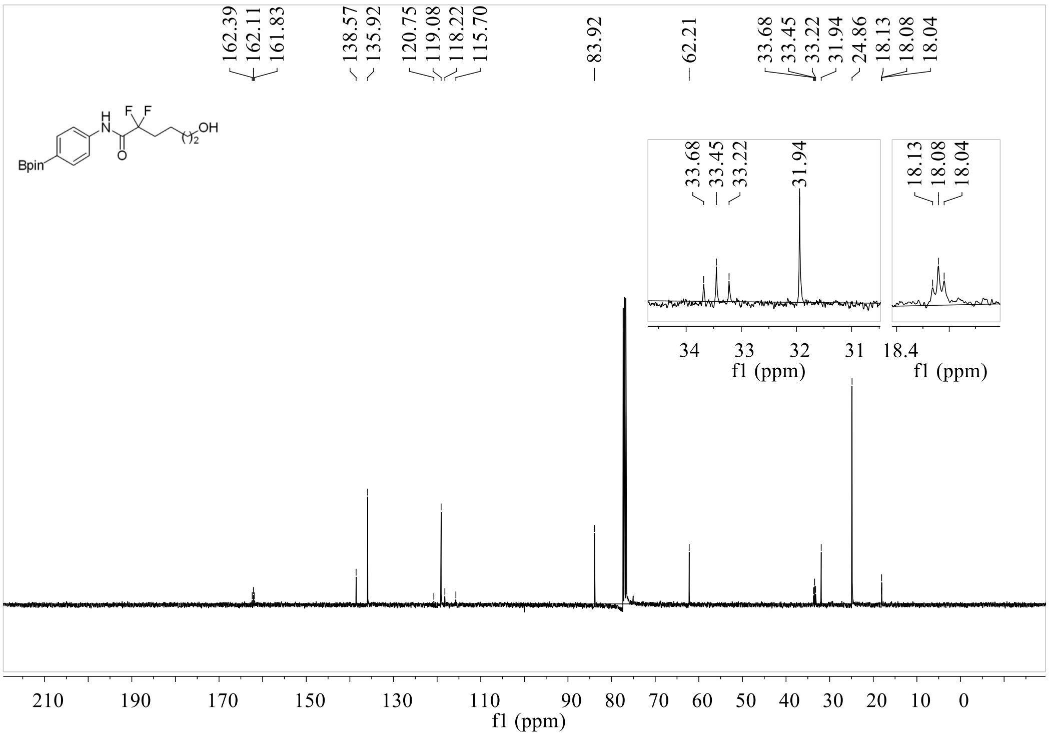

Supplementary Figure 41. ^{13}C NMR (101 MHz, $CDCl_3$) spectrum of **11**

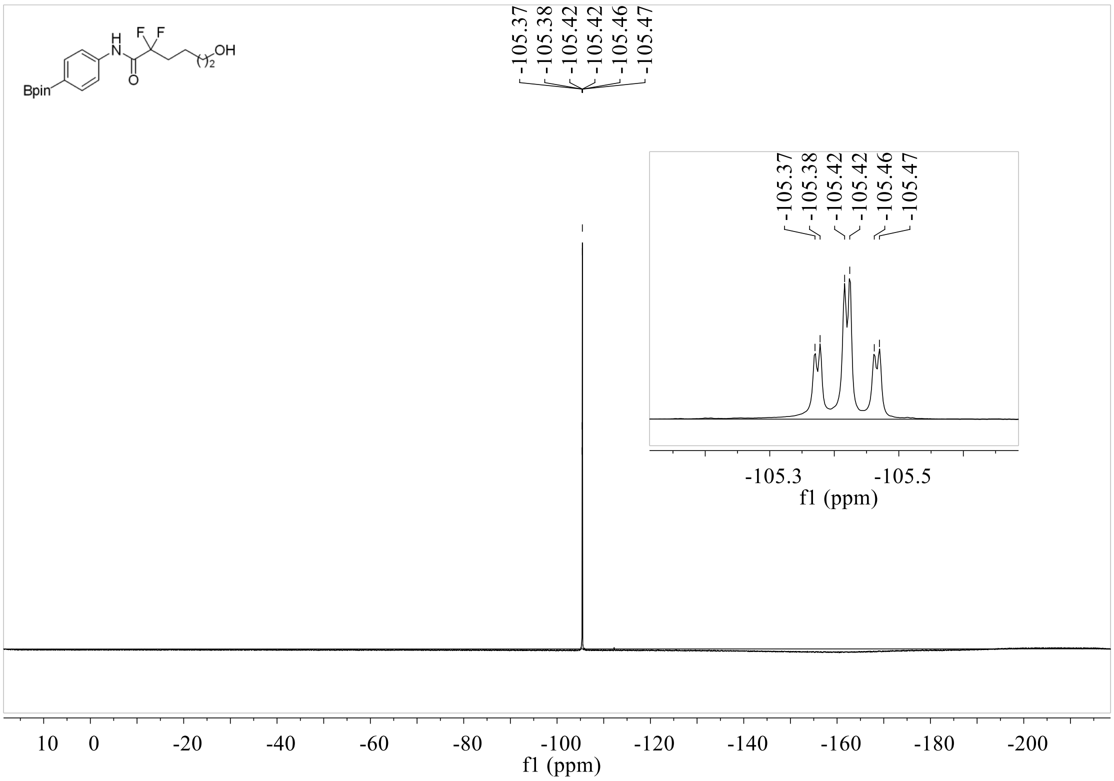

Supplementary Figure 42. ^{19}F NMR (376 MHz, $CDCl_3$) spectrum of **11**

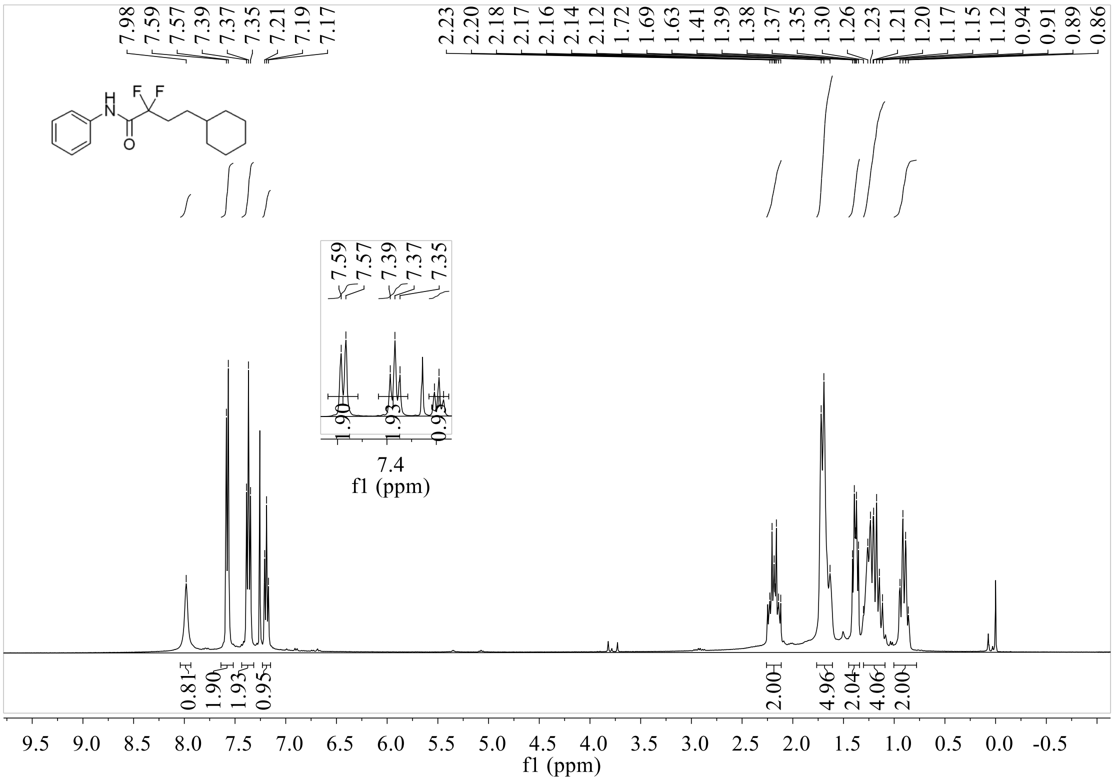

Supplementary Figure 43. ^{1}H NMR (400 MHz, $CDCl_3$) spectrum of **15**

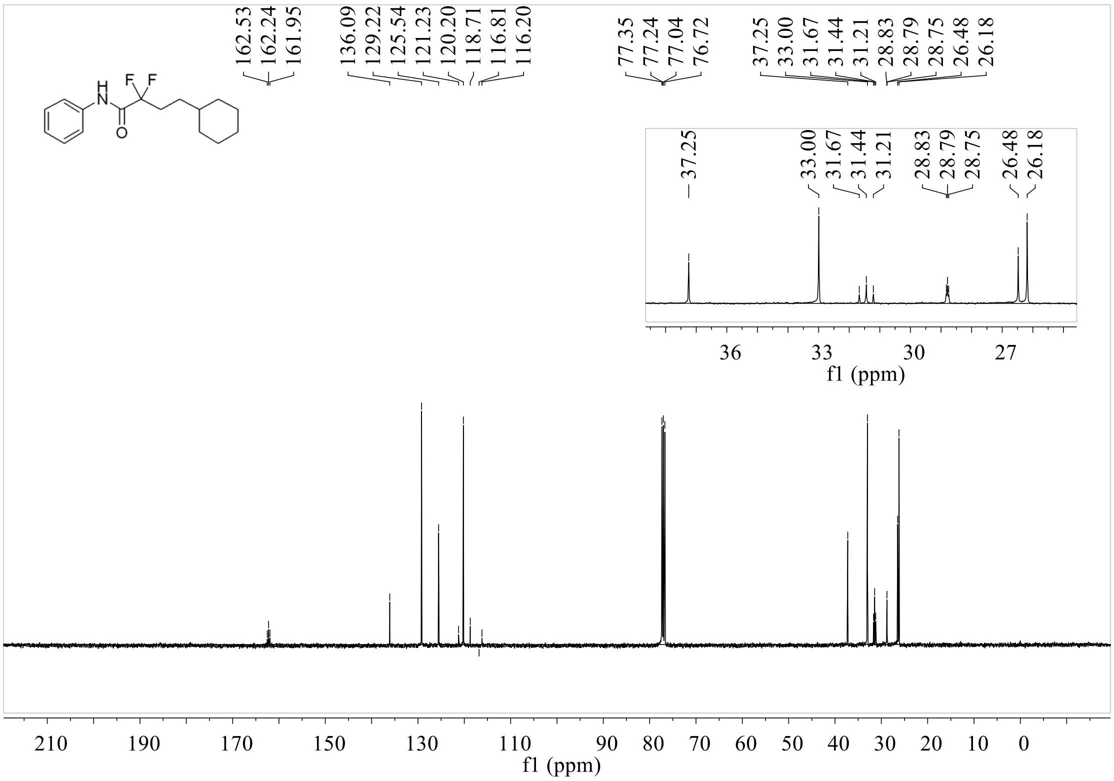

Supplementary Figure 44. ^{13}C NMR (101 MHz, $CDCl_3$) spectrum of **15**

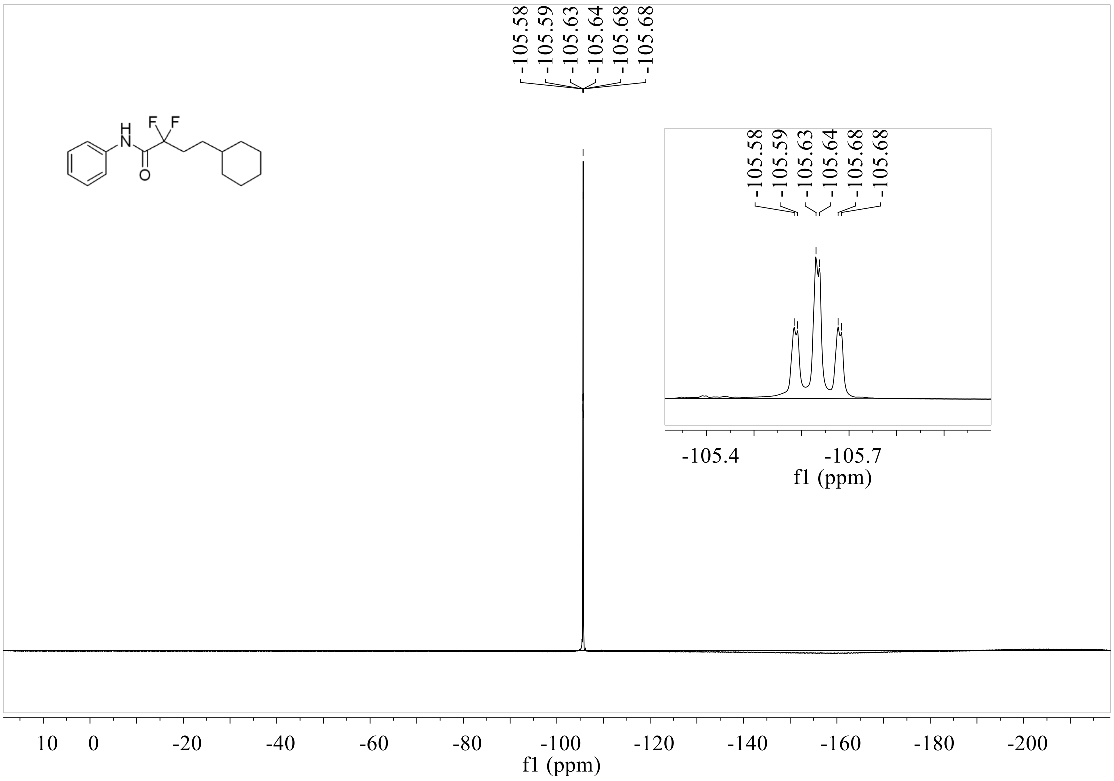

Supplementary Figure 45. ^{19}F NMR (376 MHz, $CDCl_3$) spectrum of **15**

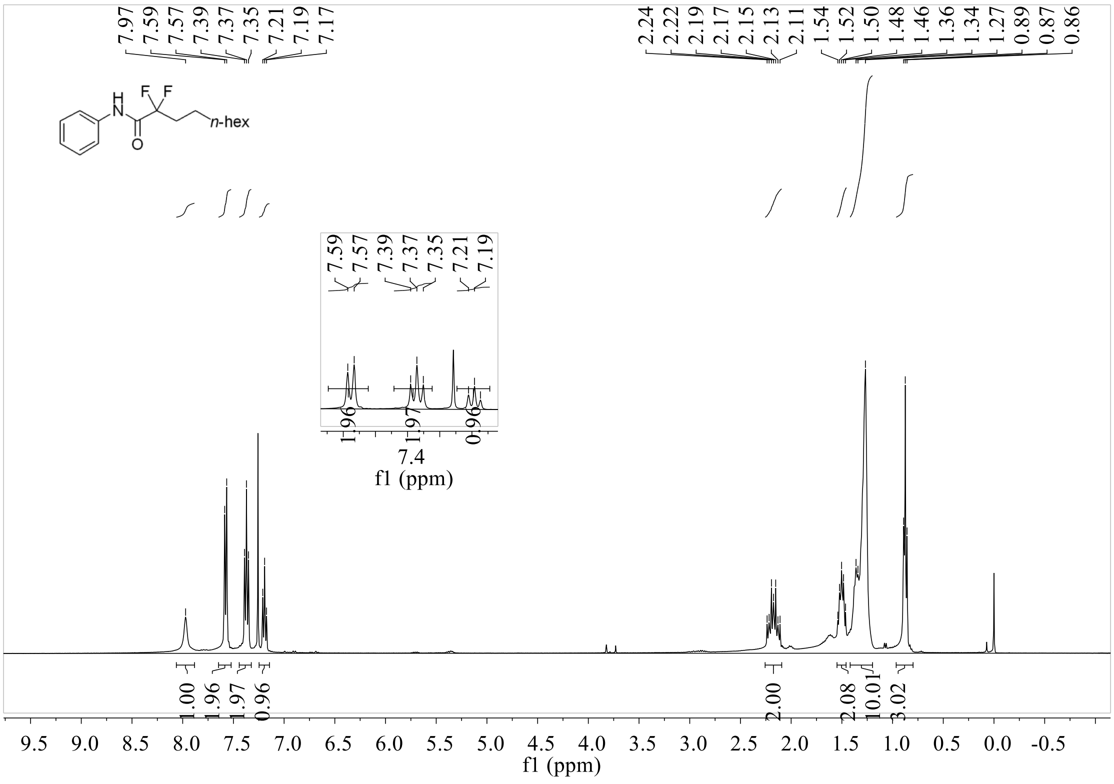

Supplementary Figure 46. ^{1}H NMR (400 MHz, $CDCl_3$) spectrum of **16**

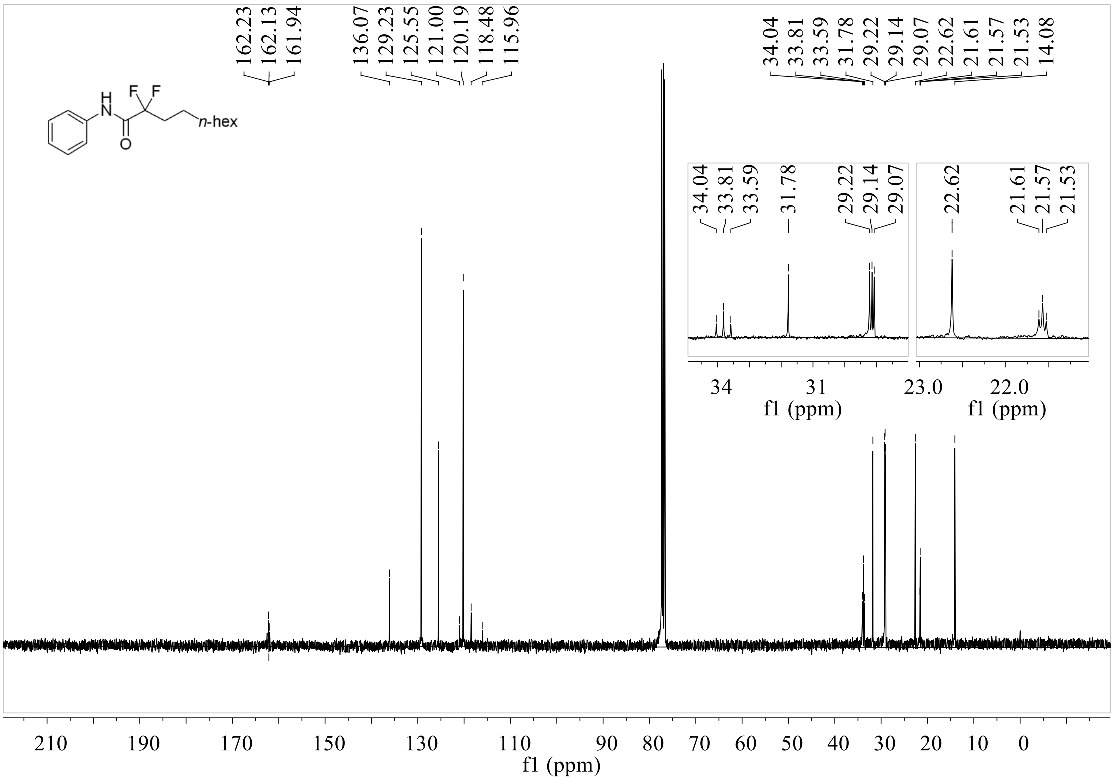

Supplementary Figure 47. ^{13}C NMR (101 MHz, $CDCl_3$) spectrum of **16**

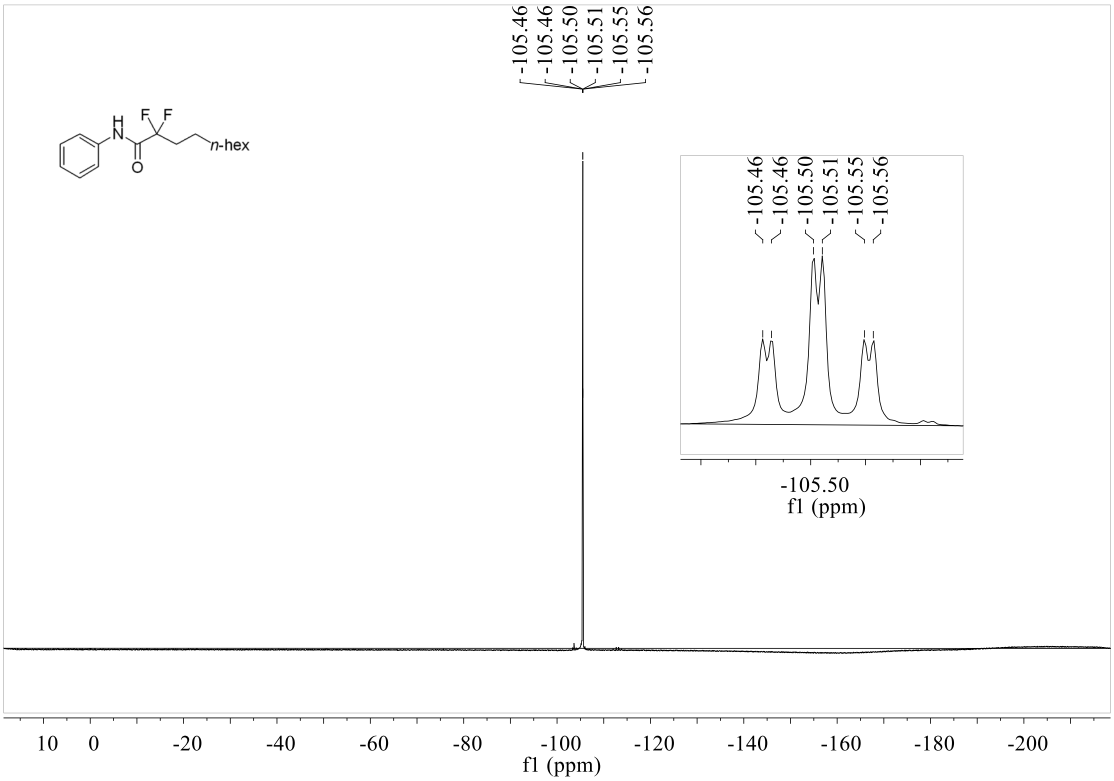

Supplementary Figure 48. ^{19}F NMR (376 MHz, $CDCl_3$) spectrum of **16**

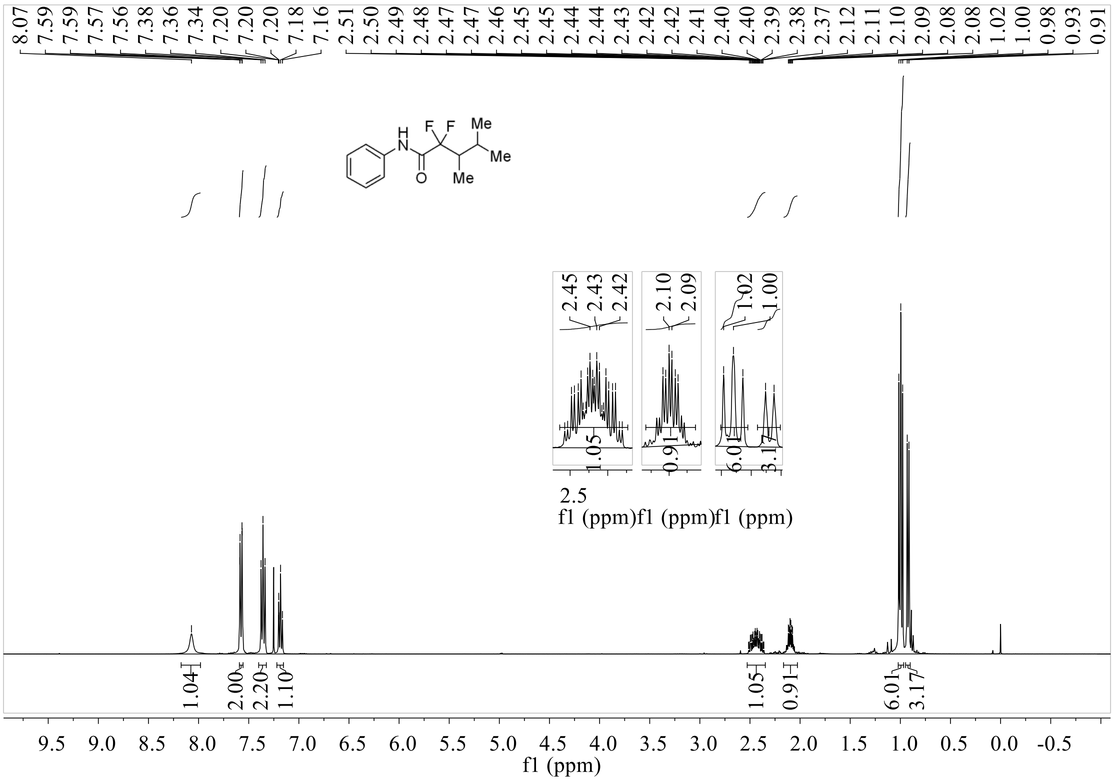

Supplementary Figure 49. ^{1}H NMR (400 MHz, $CDCl_3$) spectrum of **17**

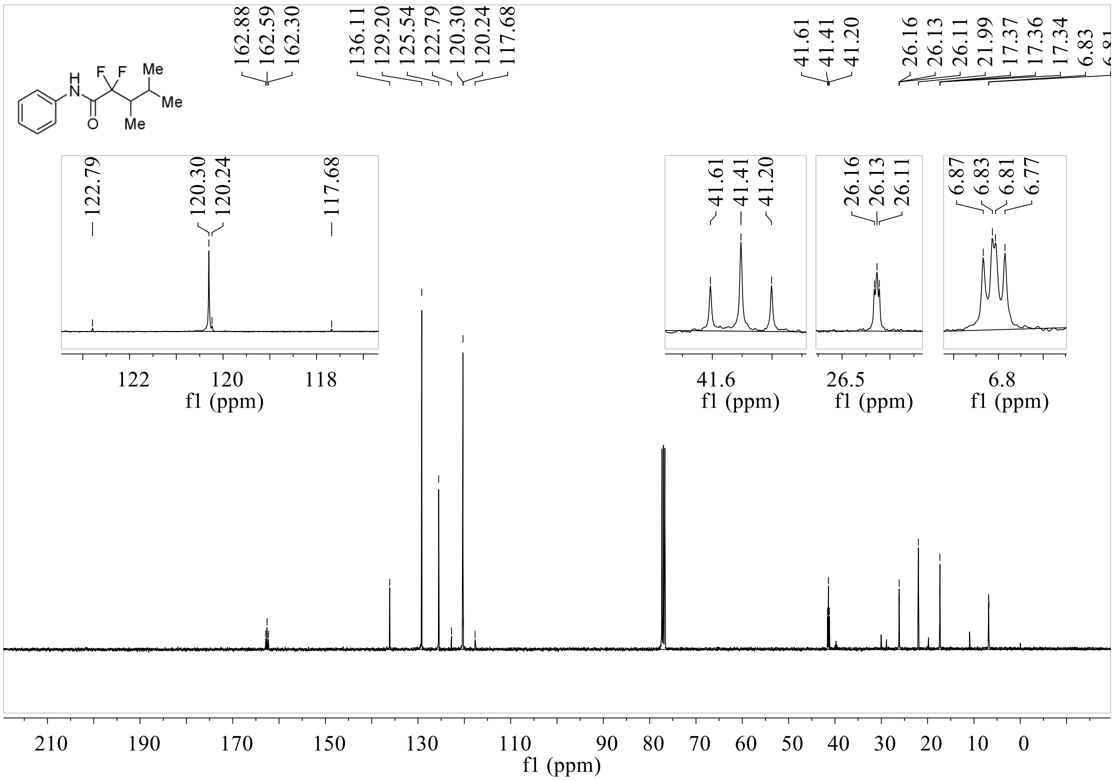

Supplementary Figure 50. ^{13}C NMR (101 MHz, $CDCl_3$) spectrum of **17**

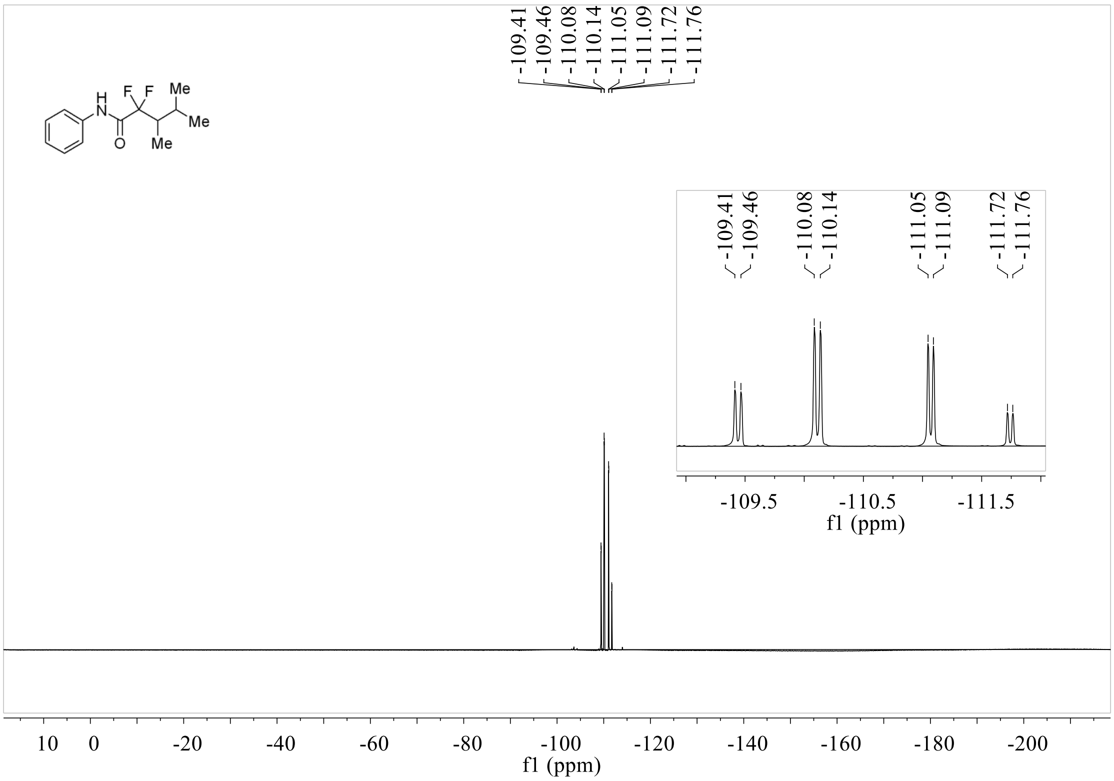

Supplementary Figure 51. ^{19}F NMR (376 MHz, $CDCl_3$) spectrum of **17**

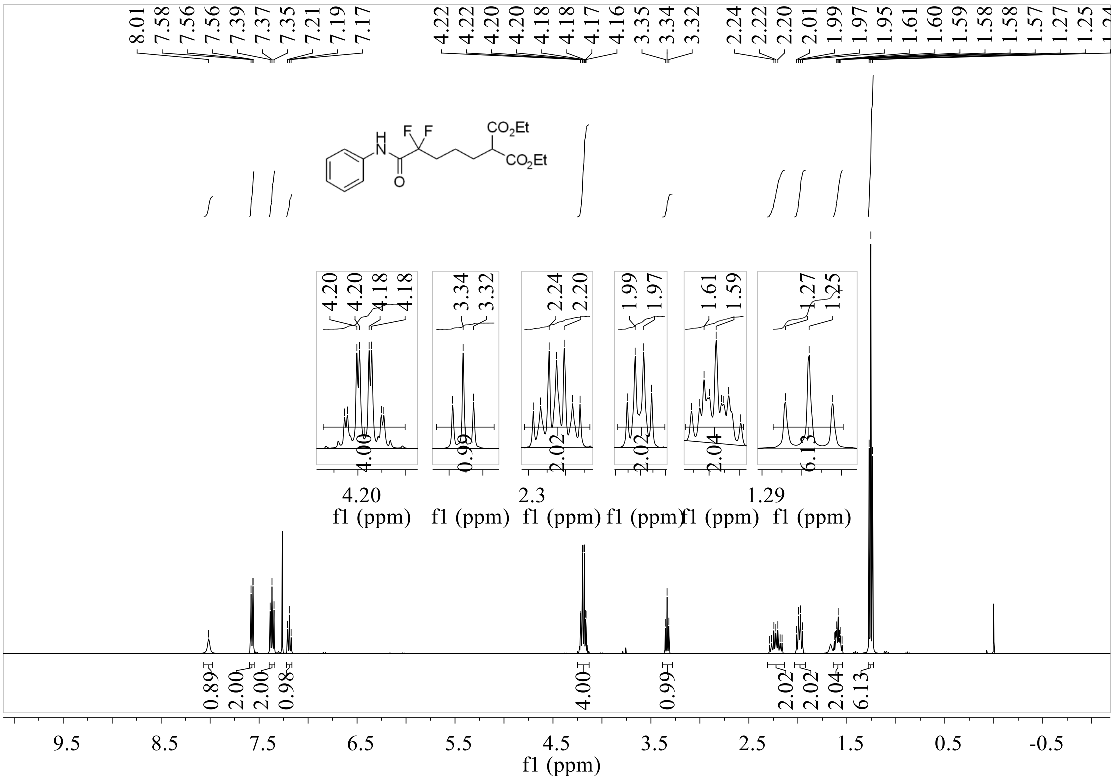

Supplementary Figure 52. ^{1}H NMR (400 MHz, $CDCl_3$) spectrum of **18**

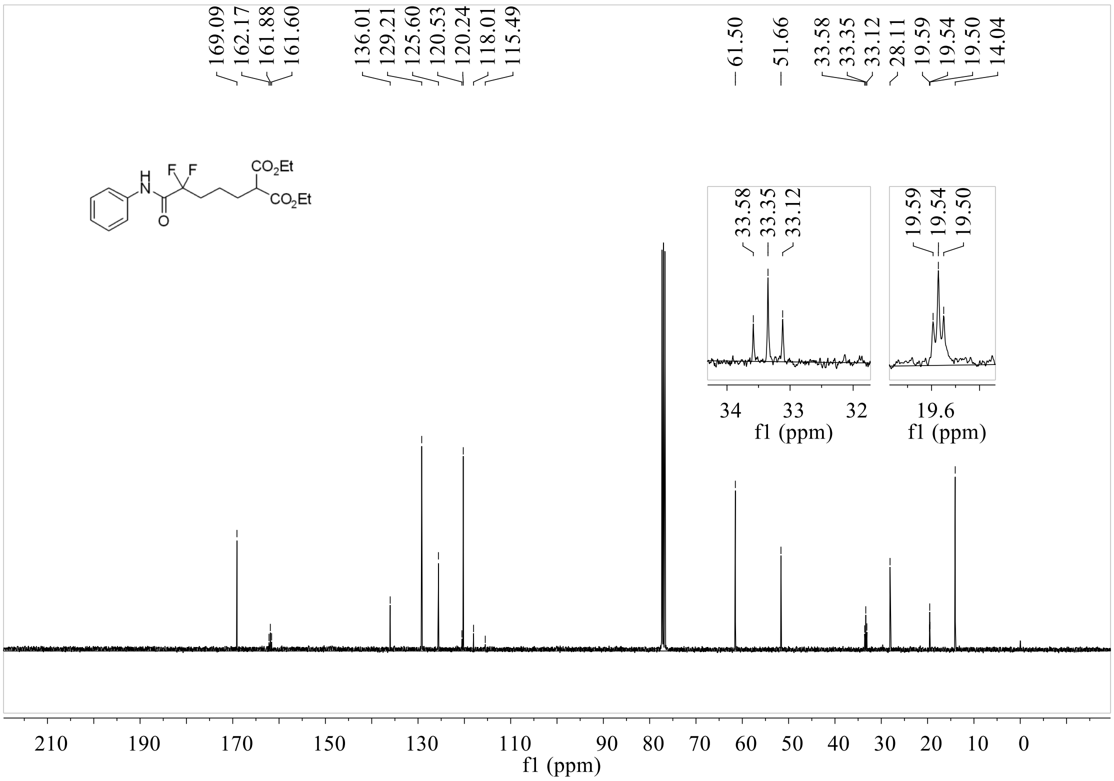

Supplementary Figure 53. ^{13}C NMR (101 MHz, $CDCl_3$) spectrum of **18**

^{19}F NMR (376 MHz, $CDCl_3$) spectrum of **diethyl 2-(4,4-difluoro-5-oxo-5-(phenylamino)pentyl)malonate (18)**

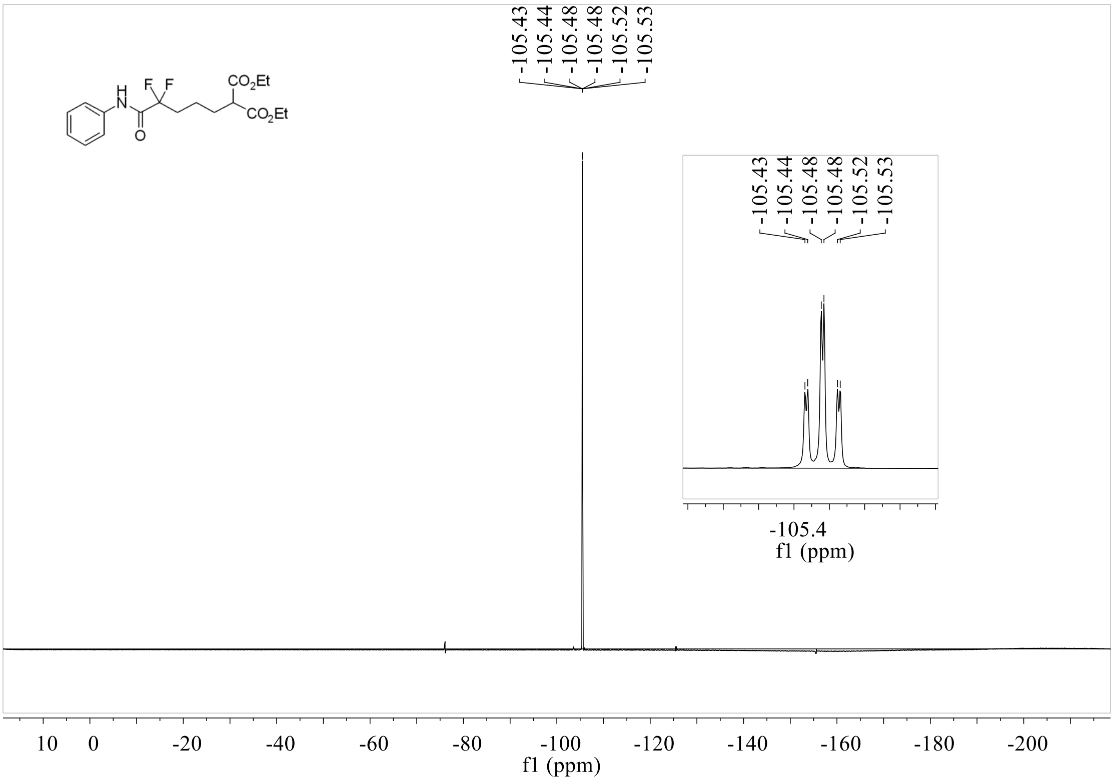

Supplementary Figure 54. ^{19}F NMR (376 MHz, $CDCl_3$) spectrum of **18**

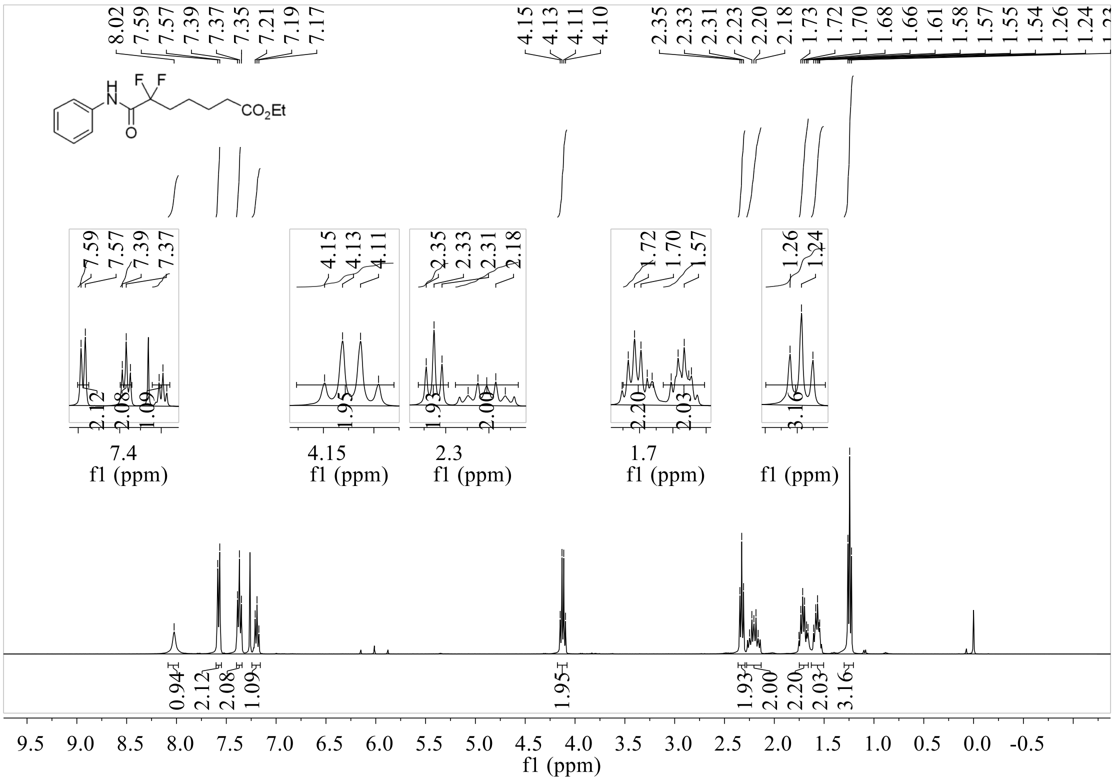

Supplementary Figure 55. ^{1}H NMR (400 MHz, $CDCl_3$) spectrum of **19**

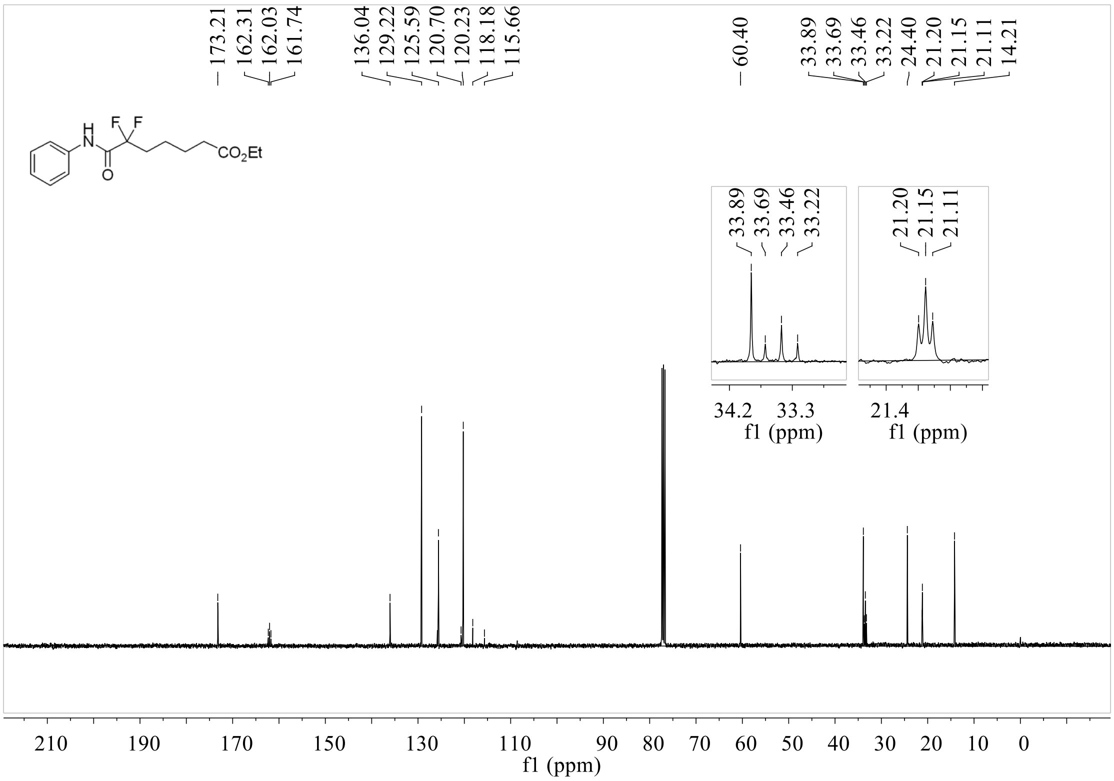

Supplementary Figure 56. ^{13}C NMR (101 MHz, $CDCl_3$) spectrum of **19**

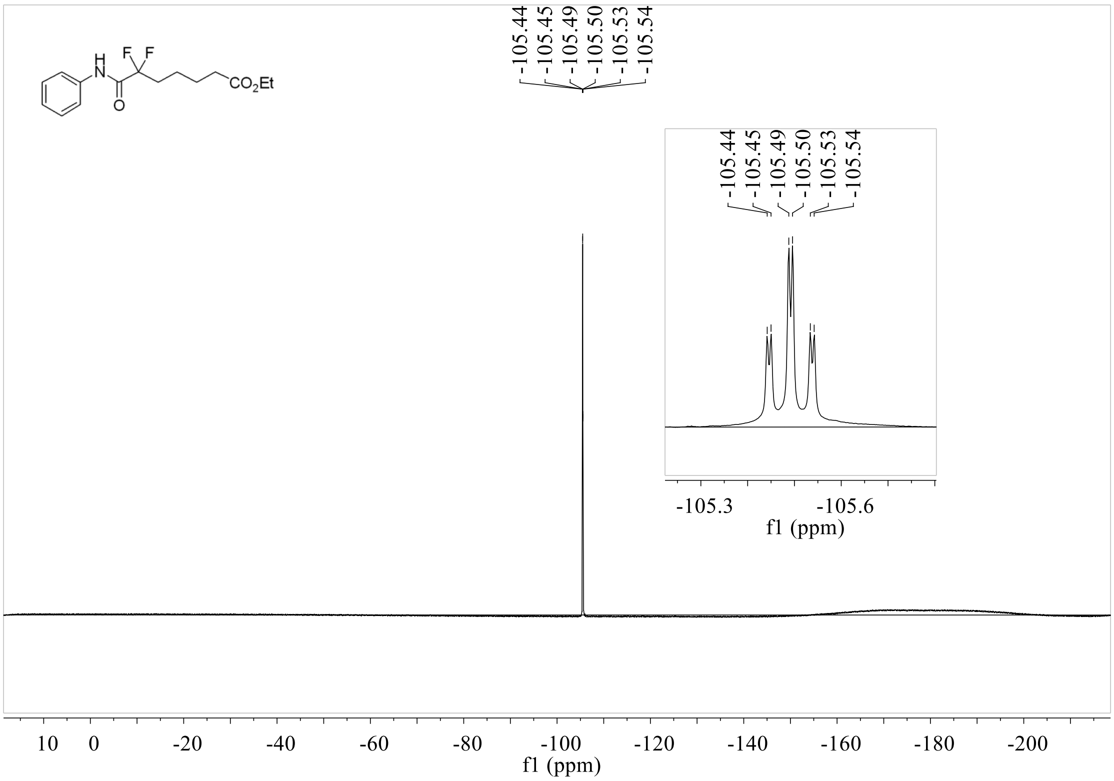

Supplementary Figure 57. ^{19}F NMR (376 MHz, $CDCl_3$) spectrum of **19**

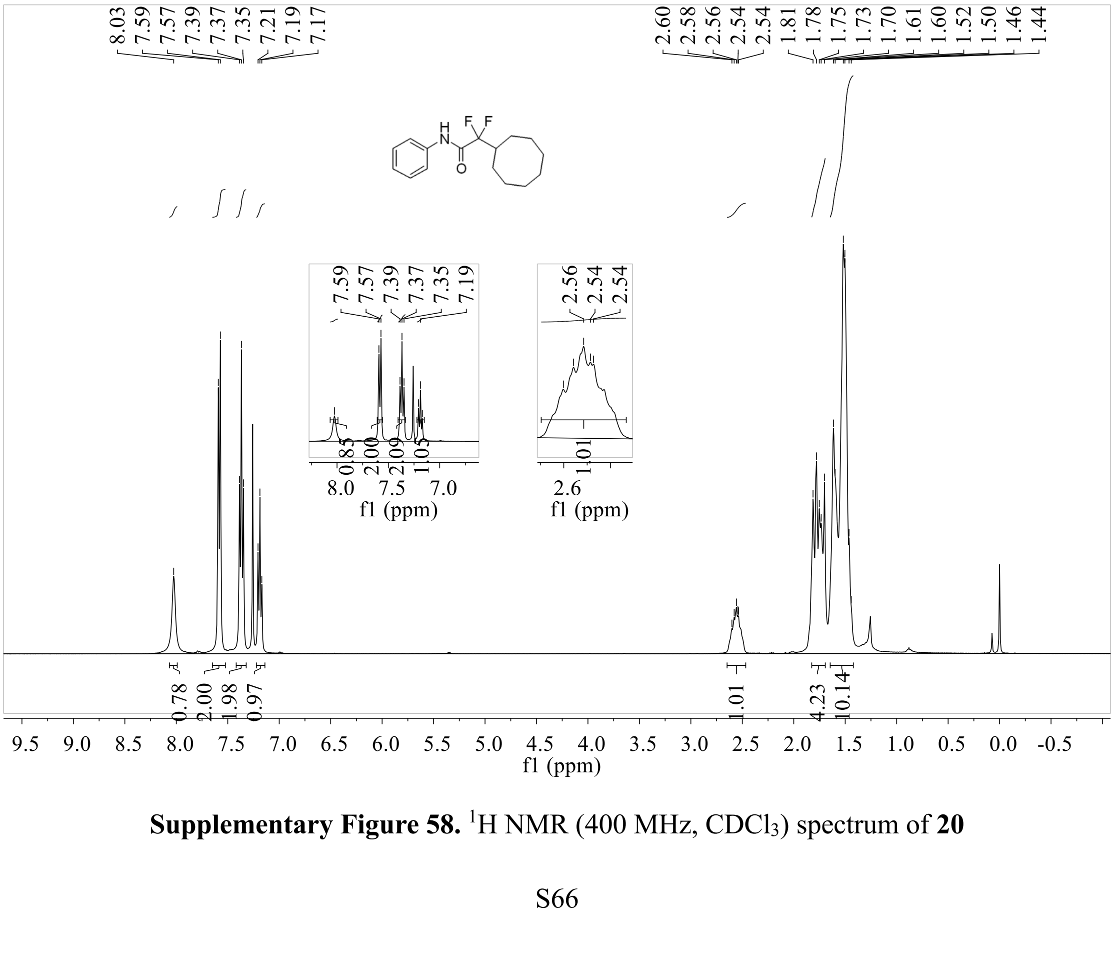

Supplementary Figure 58. ^{1}H NMR (400 MHz, $CDCl_3$) spectrum of **20**

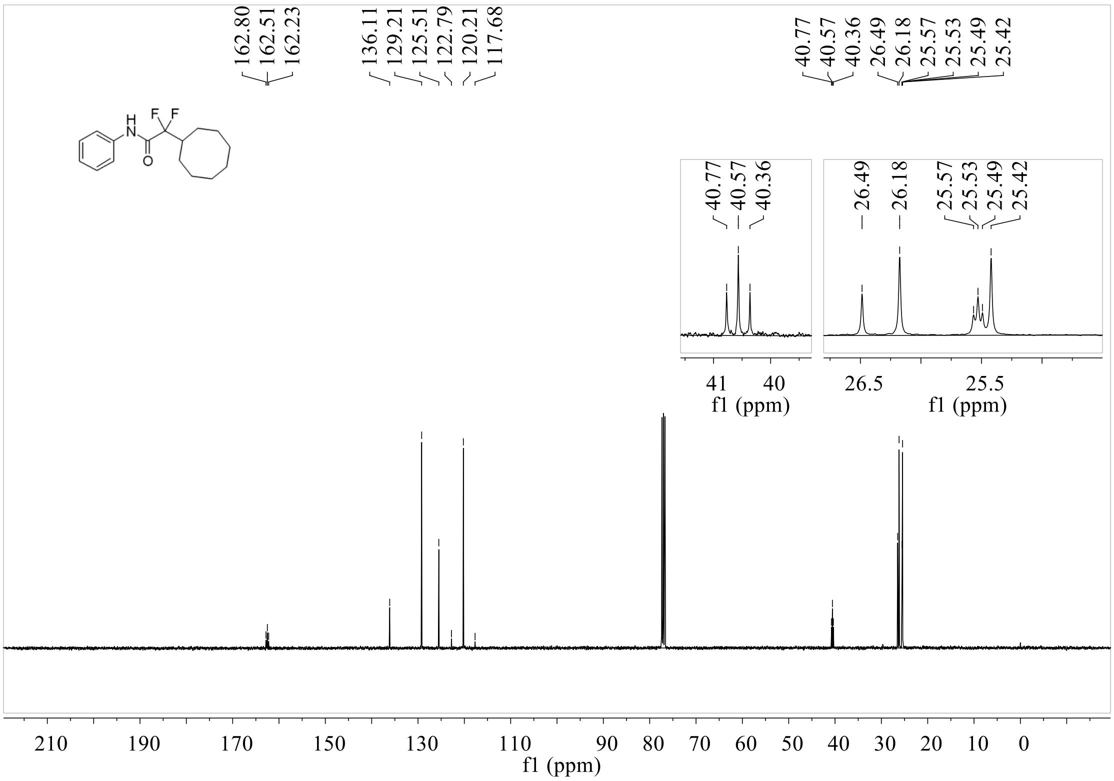

Supplementary Figure 59. ^{13}C NMR (101 MHz, $CDCl_3$) spectrum of **20**

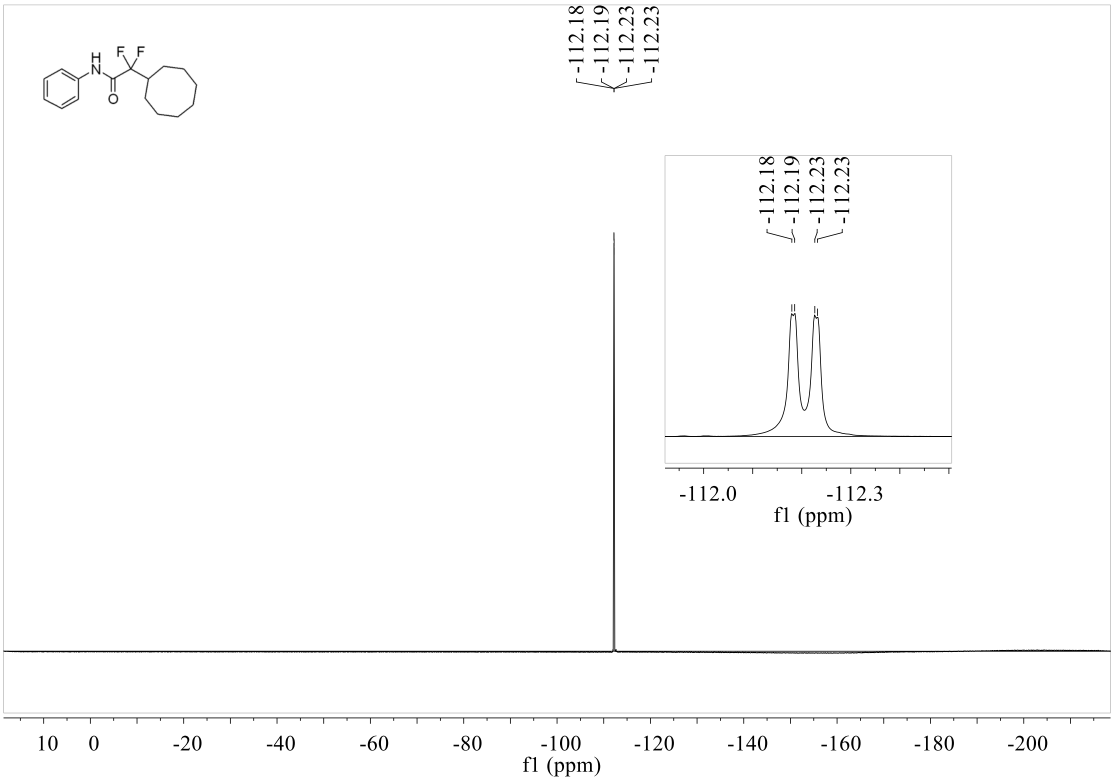

Supplementary Figure 60. ^{19}F NMR (376 MHz, $CDCl_3$) spectrum of **20**

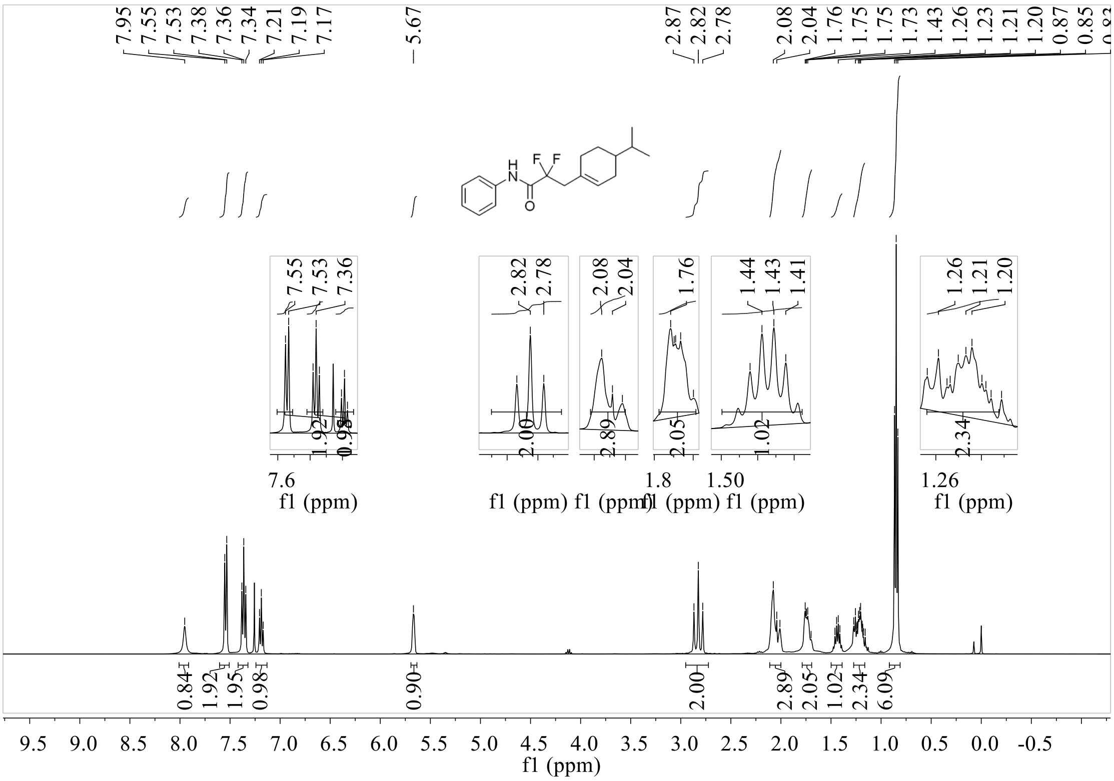

Supplementary Figure 61. ^{1}H NMR (400 MHz, $CDCl_3$) spectrum of **21**

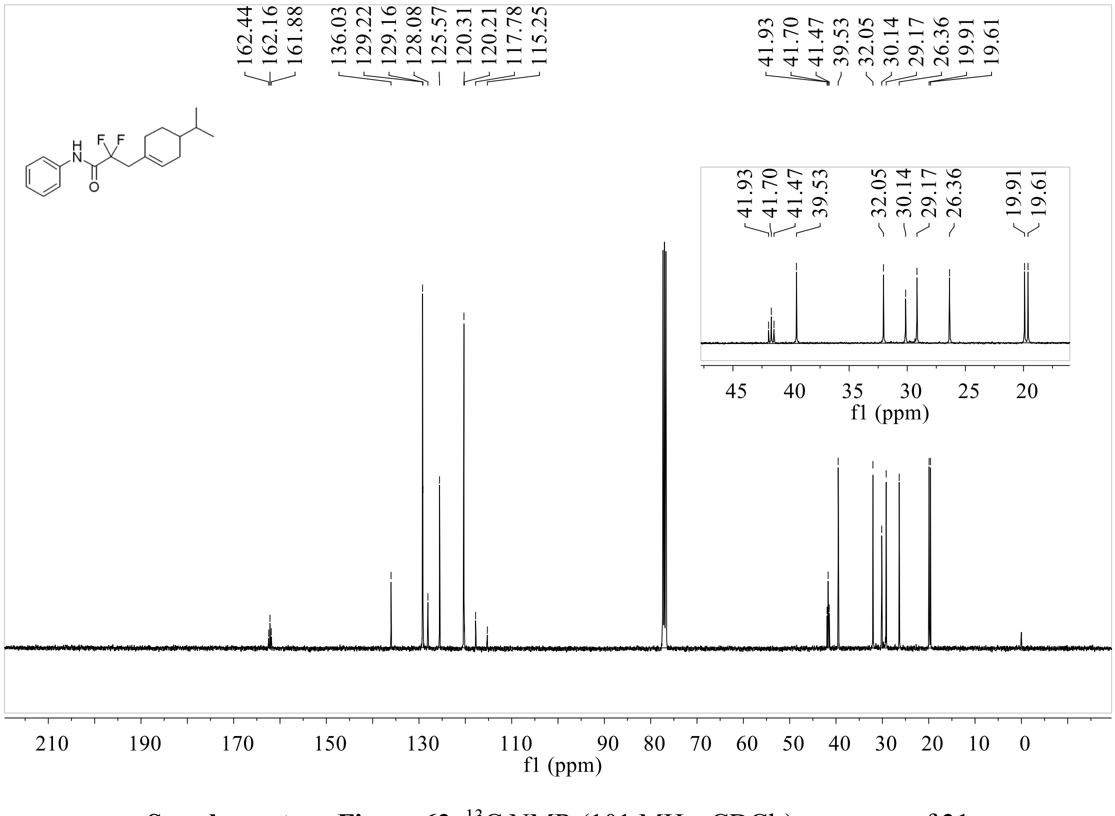

Supplementary Figure 62. ^{13}C NMR (101 MHz, $CDCl_3$) spectrum of **21**

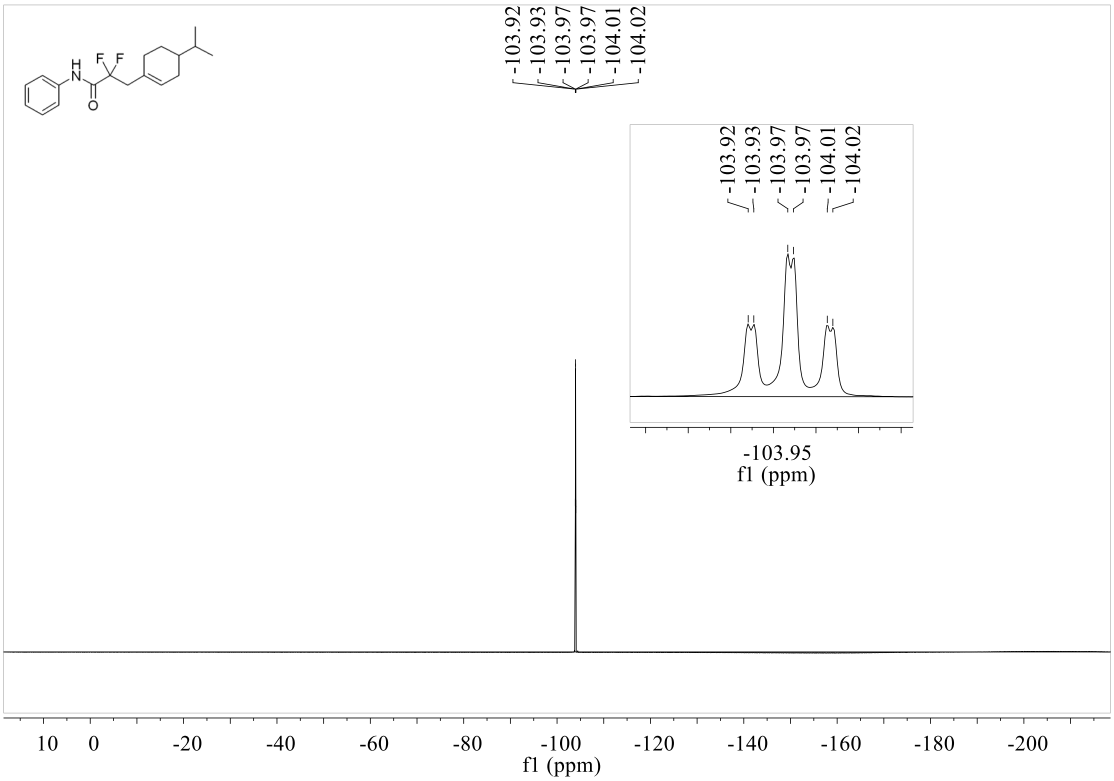

Supplementary Figure 63. ^{19}F NMR (376 MHz, $CDCl_3$) spectrum of **21**

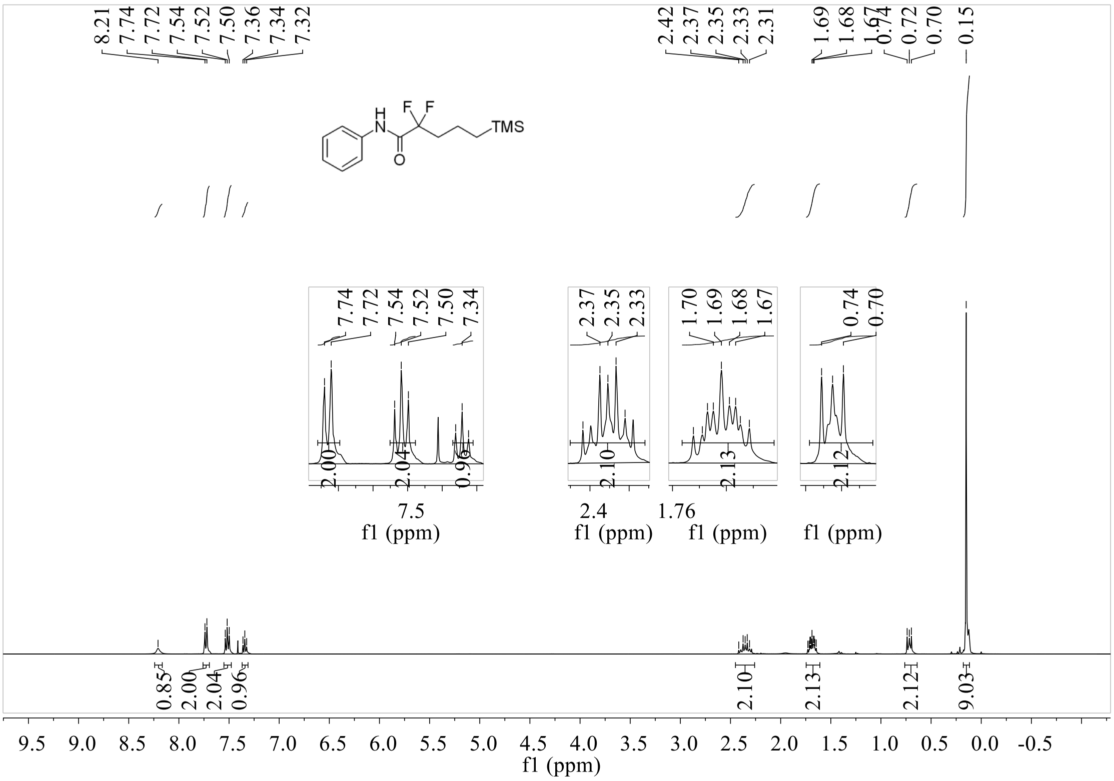

Supplementary Figure 64. ^{1}H NMR (400 MHz, $CDCl_3$) spectrum of **22**

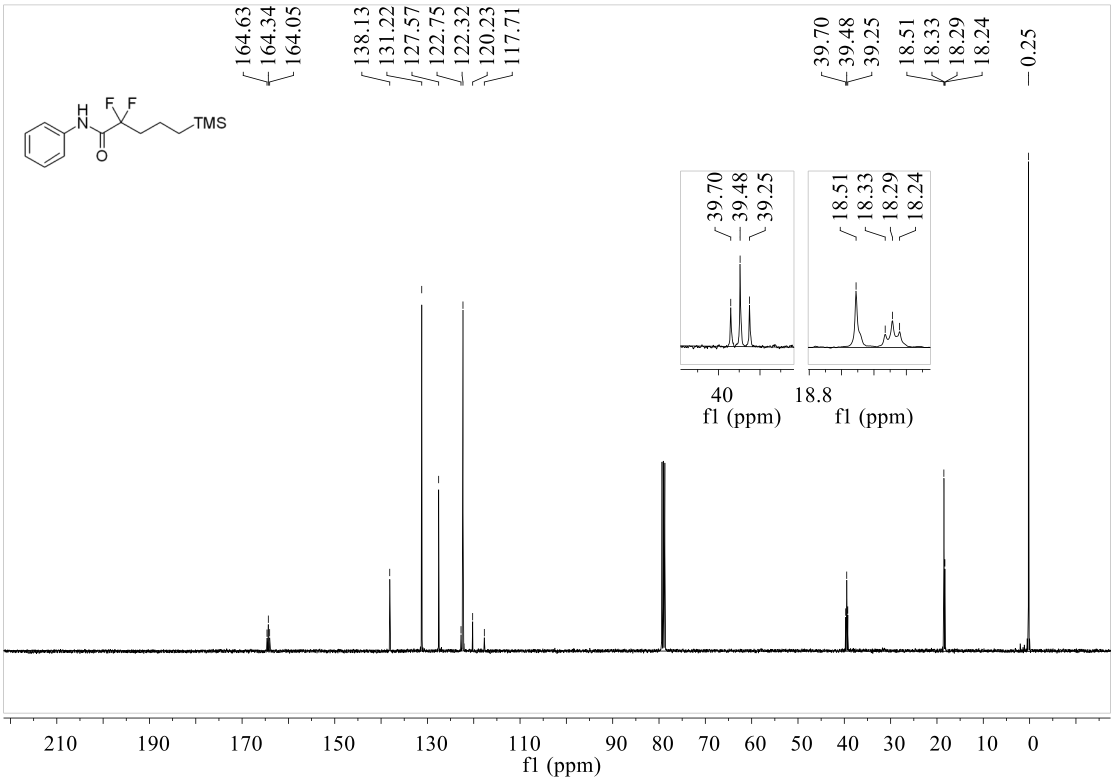

Supplementary Figure 65. ^{13}C NMR (101 MHz, $CDCl_3$) spectrum of **22**

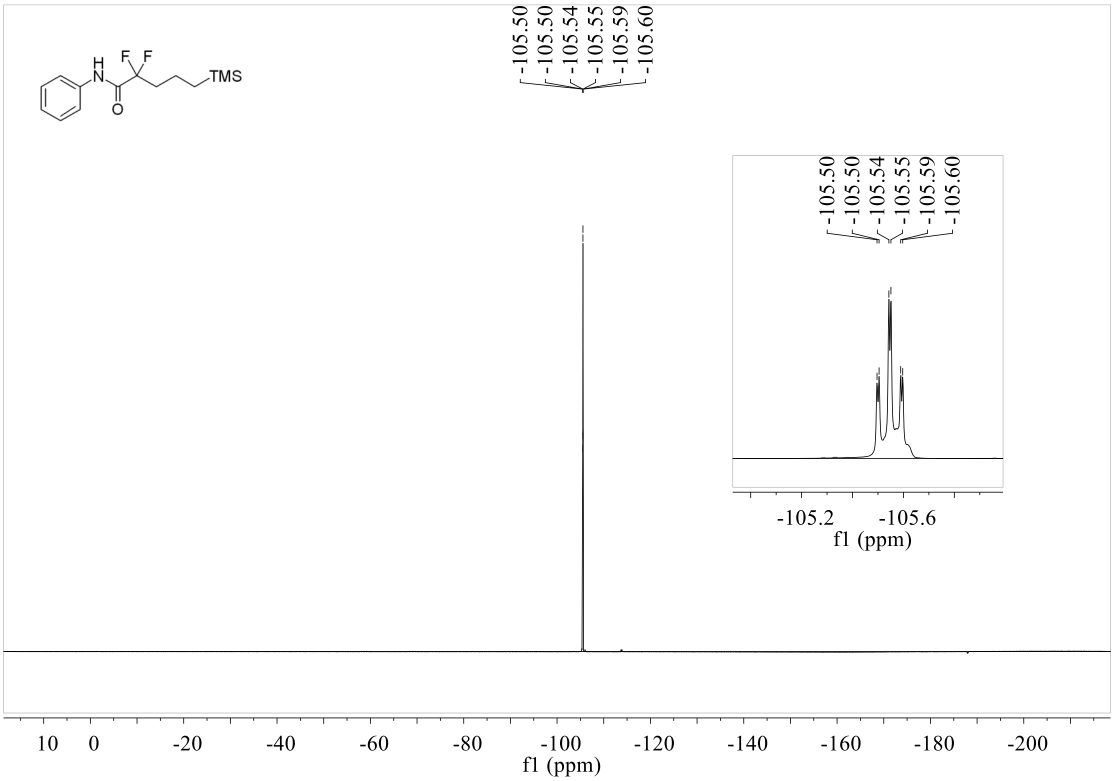

Supplementary Figure 66. ^{19}F NMR (376 MHz, $CDCl_3$) spectrum of **22**

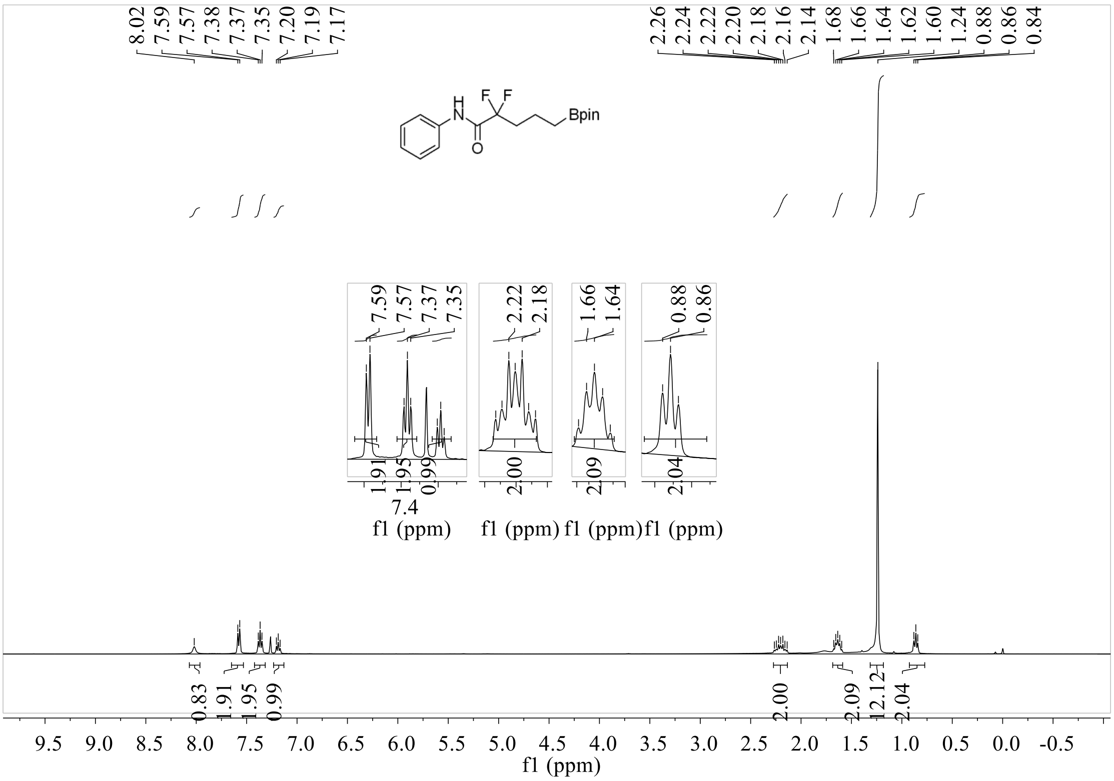

Supplementary Figure 67. ^{1}H NMR (400 MHz, $CDCl_3$) spectrum of **23**

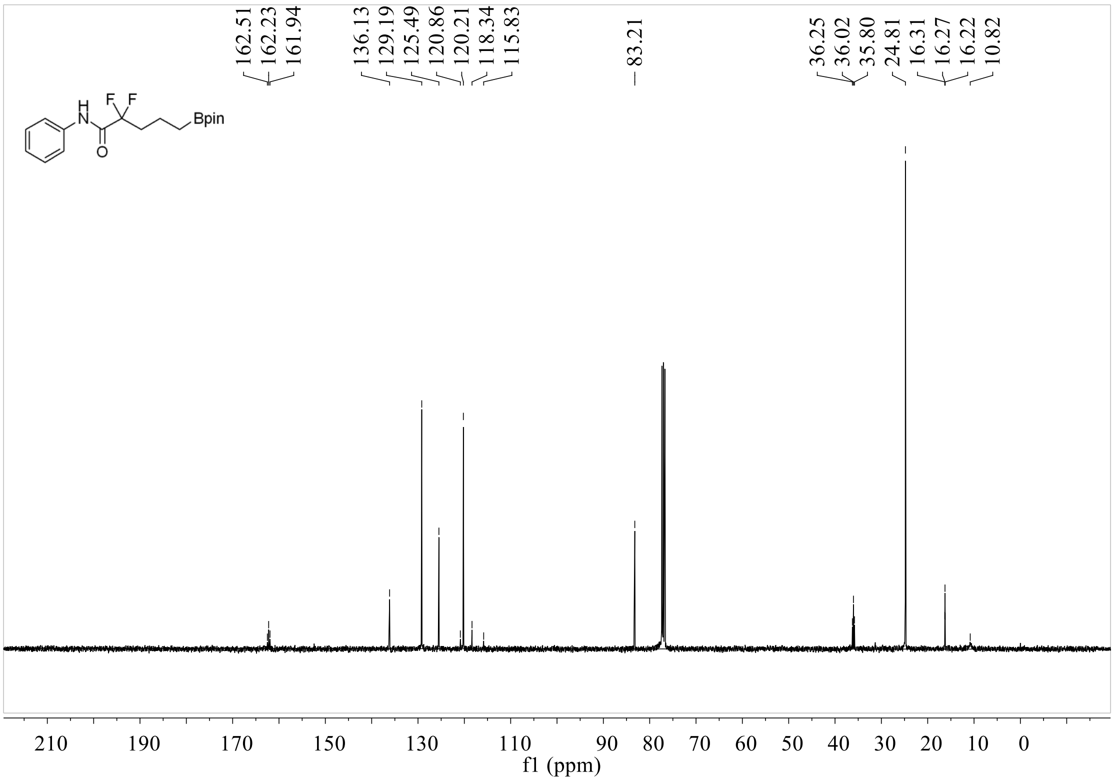

Supplementary Figure 68. ^{13}C NMR (101 MHz, $CDCl_3$) spectrum of **23**

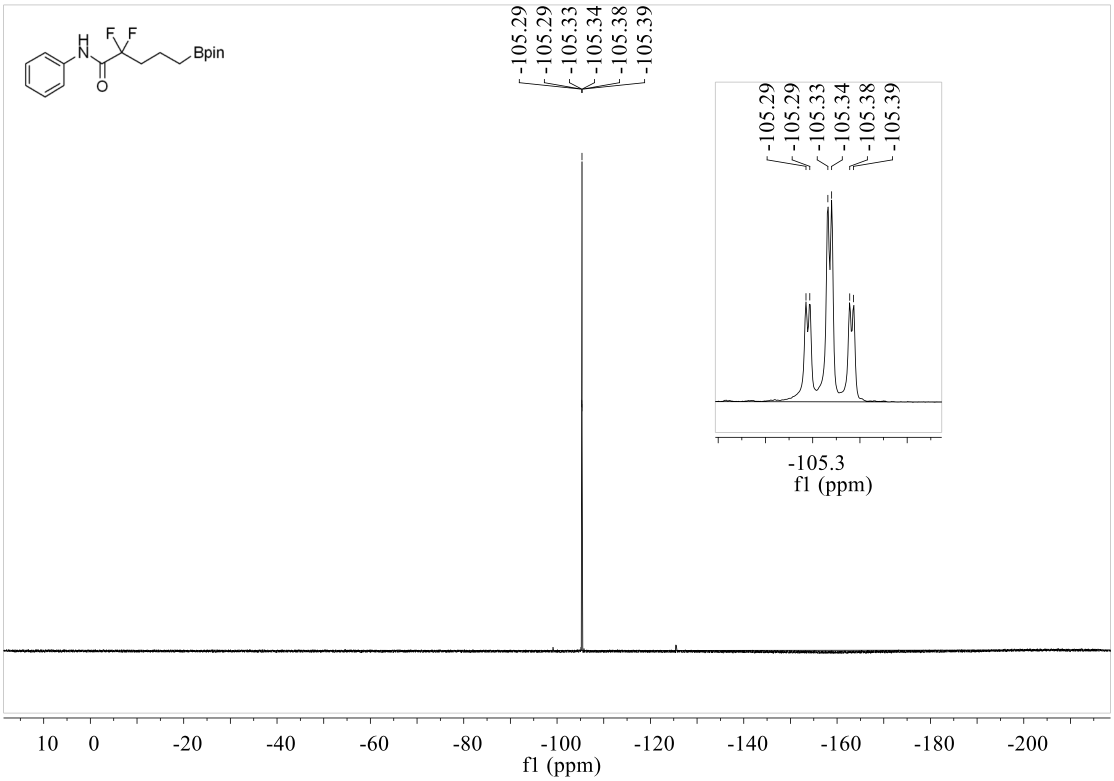

Supplementary Figure 69. ^{19}F NMR (376 MHz, $CDCl_3$) spectrum of **23**

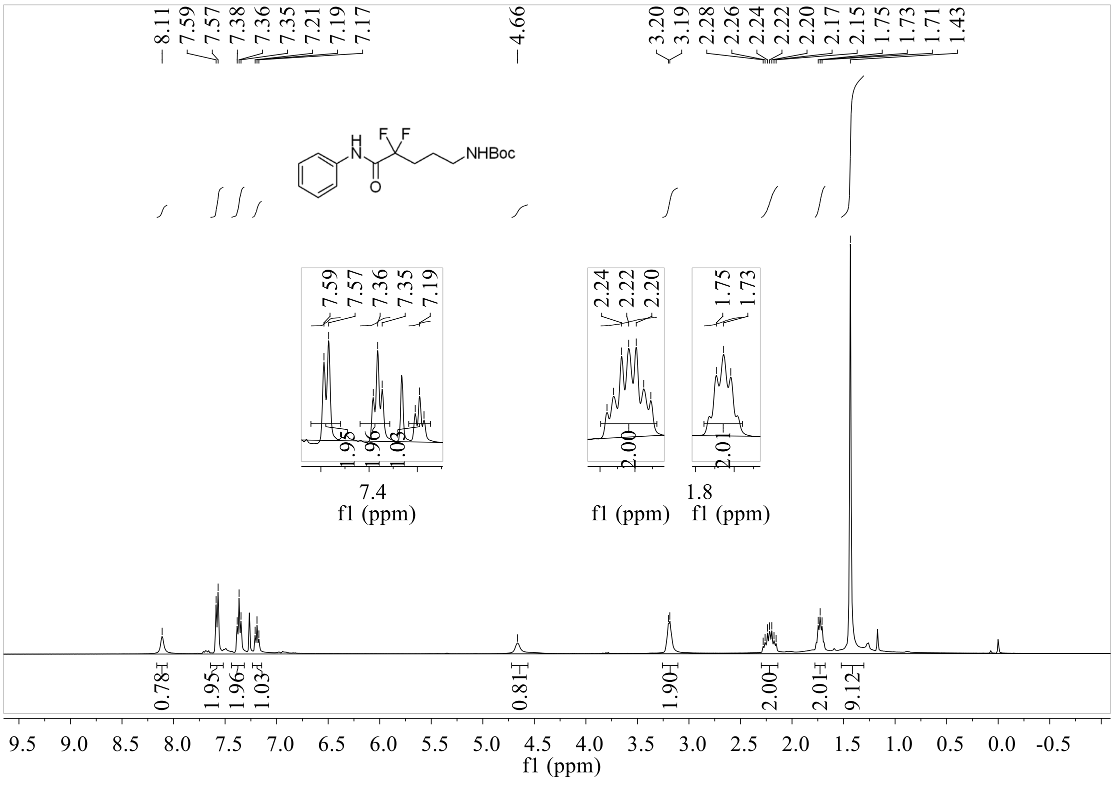

Supplementary Figure 70. ^{1}H NMR (400 MHz, $CDCl_3$) spectrum of **24**

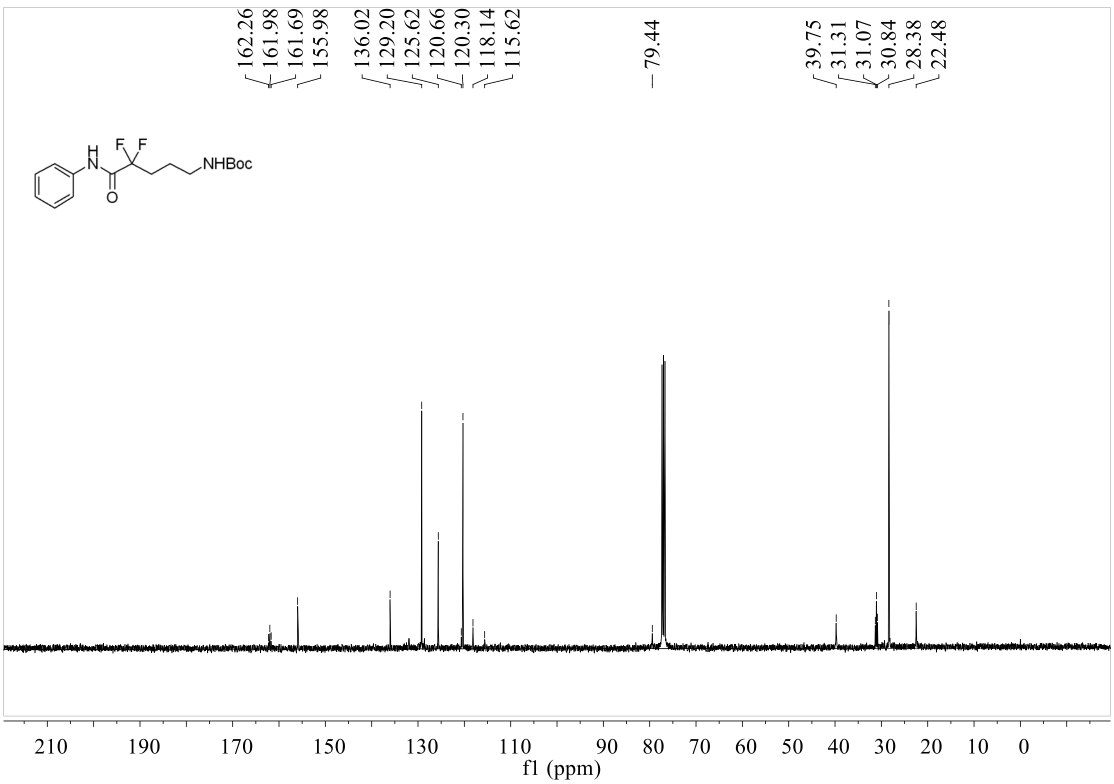

Supplementary Figure 71. ^{13}C NMR (101 MHz, $CDCl_3$) spectrum of **24**

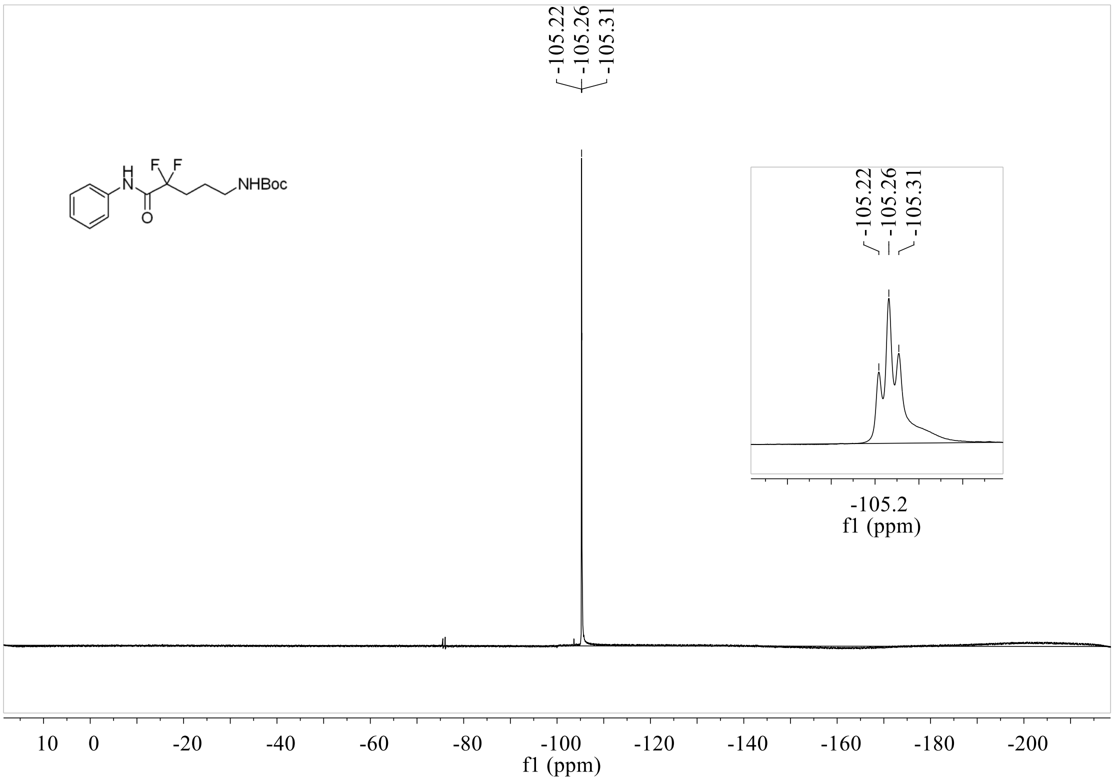

Supplementary Figure 72. ^{19}F NMR (376 MHz, $CDCl_3$) spectrum of **24**

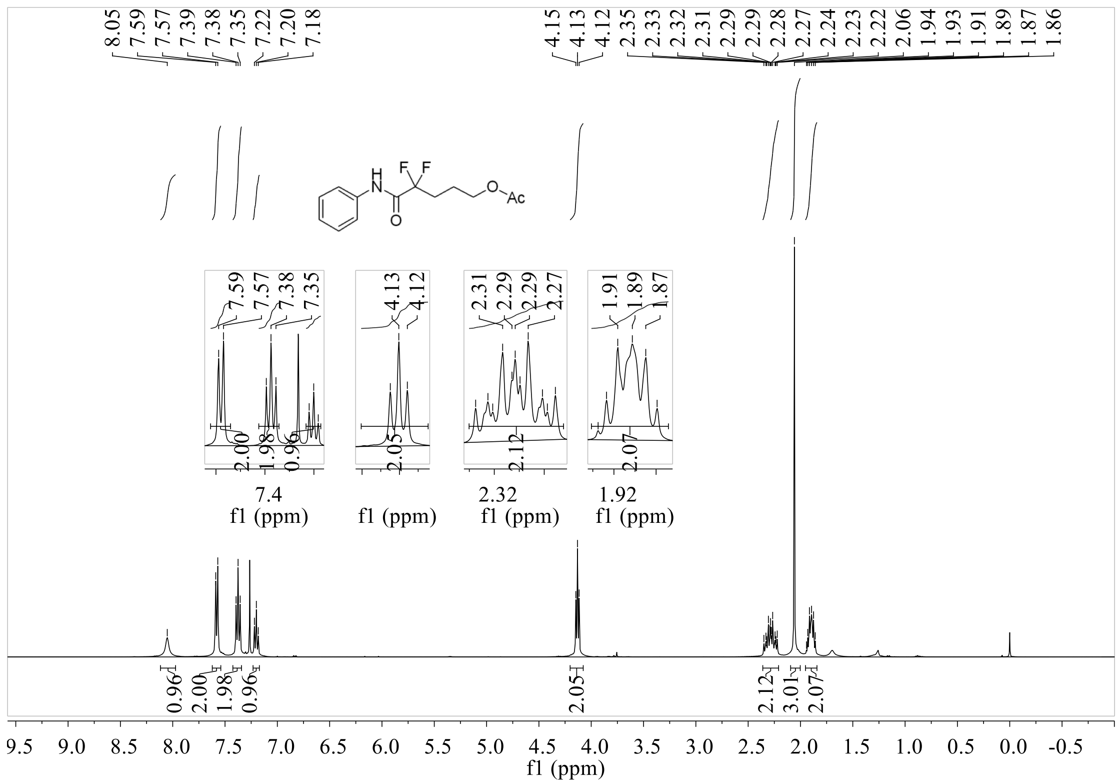

Supplementary Figure 73. ^{1}H NMR (400 MHz, $CDCl_3$) spectrum of **25**

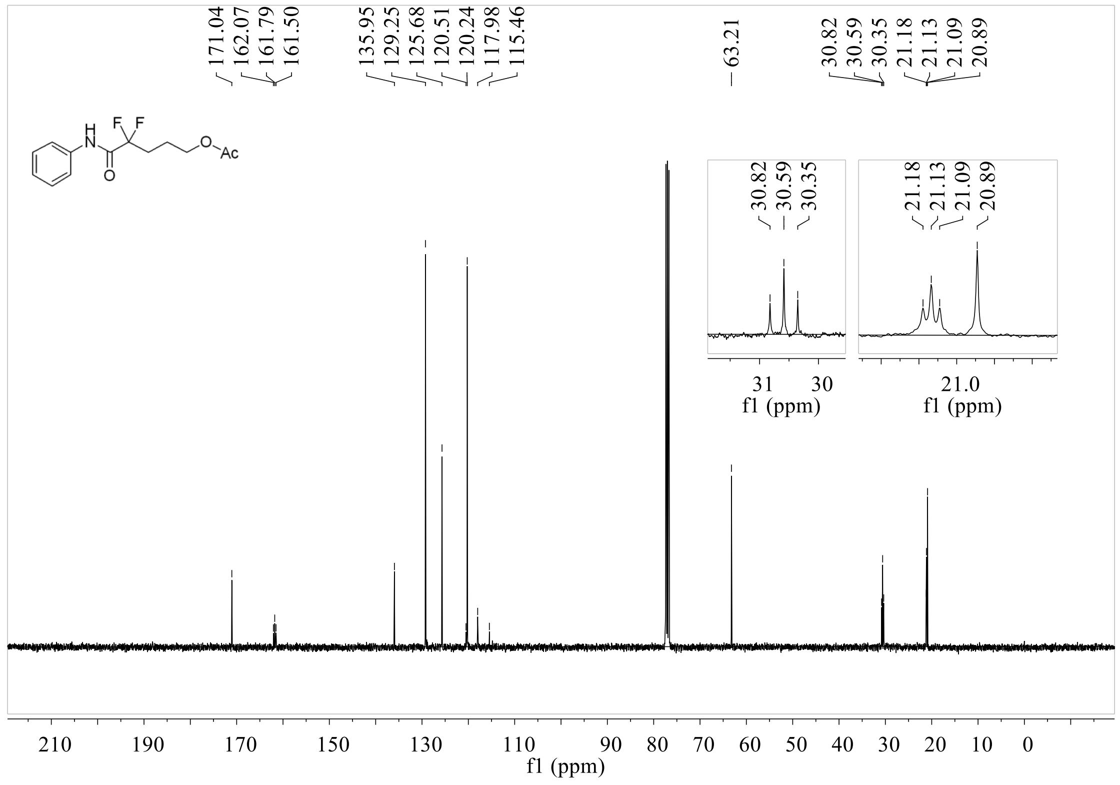

Supplementary Figure 74. ^{13}C NMR (101 MHz, $CDCl_3$) spectrum of **25**

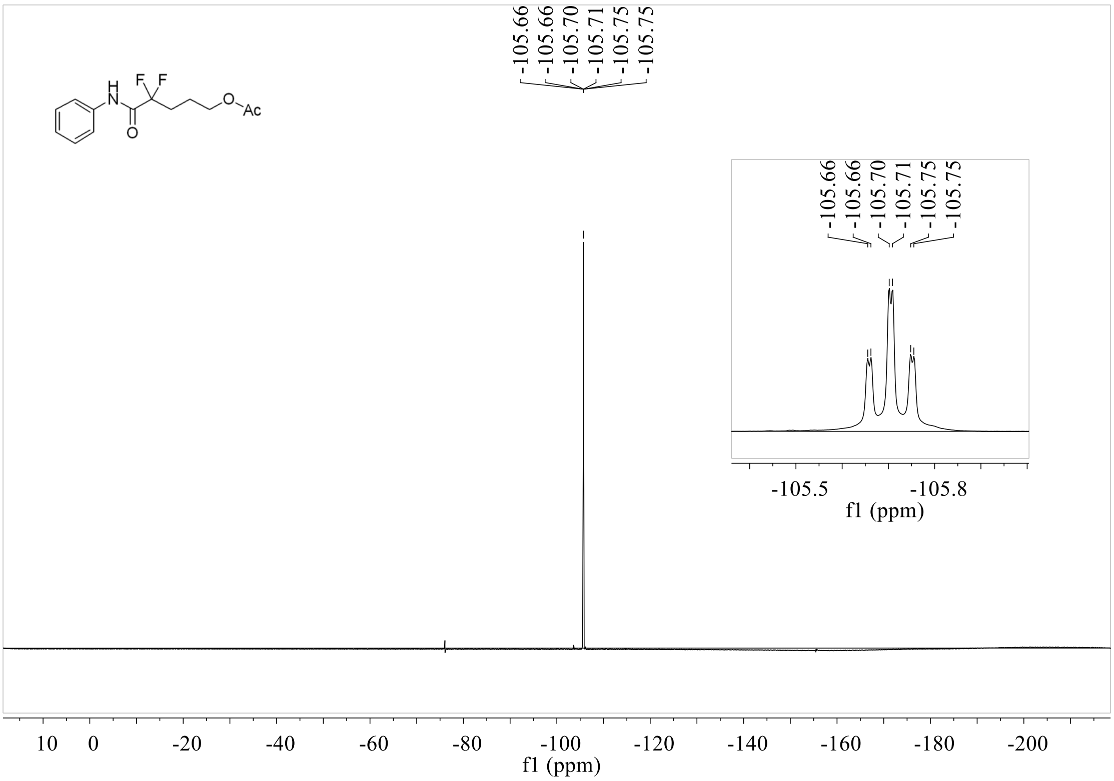

Supplementary Figure 75. ^{19}F NMR (376 MHz, $CDCl_3$) spectrum of **25**

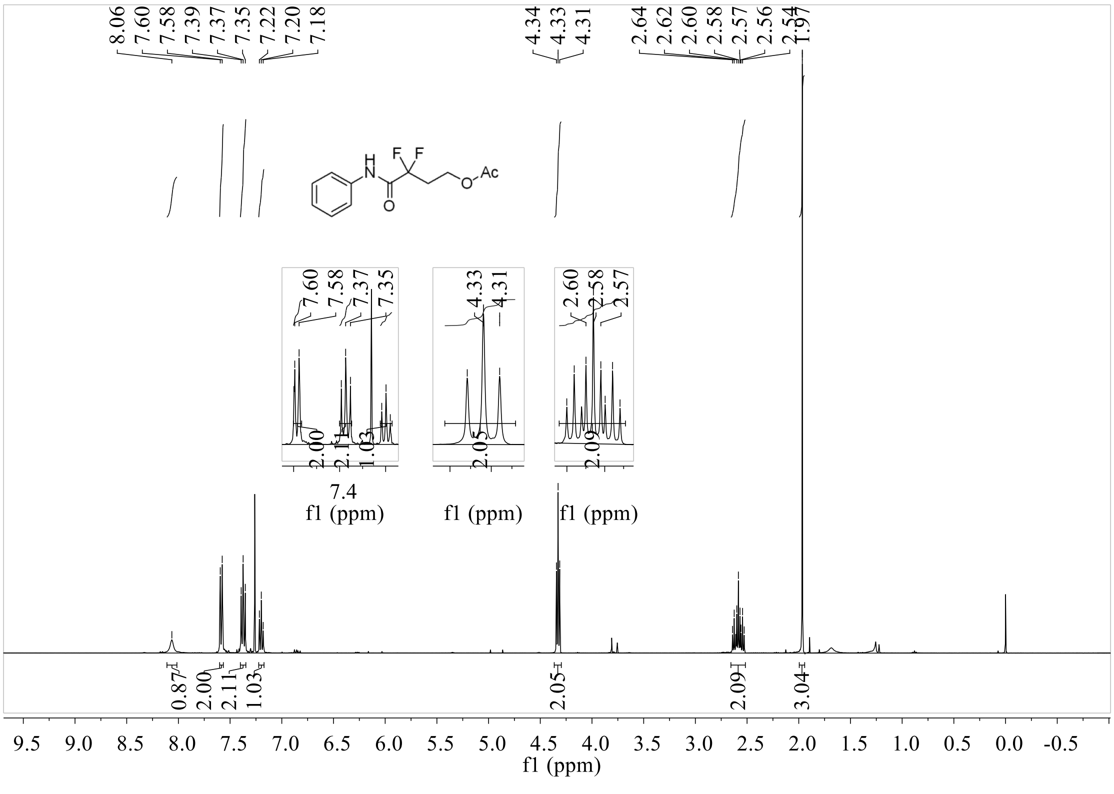

Supplementary Figure 76. ^{1}H NMR (400 MHz, $CDCl_3$) spectrum of **26**

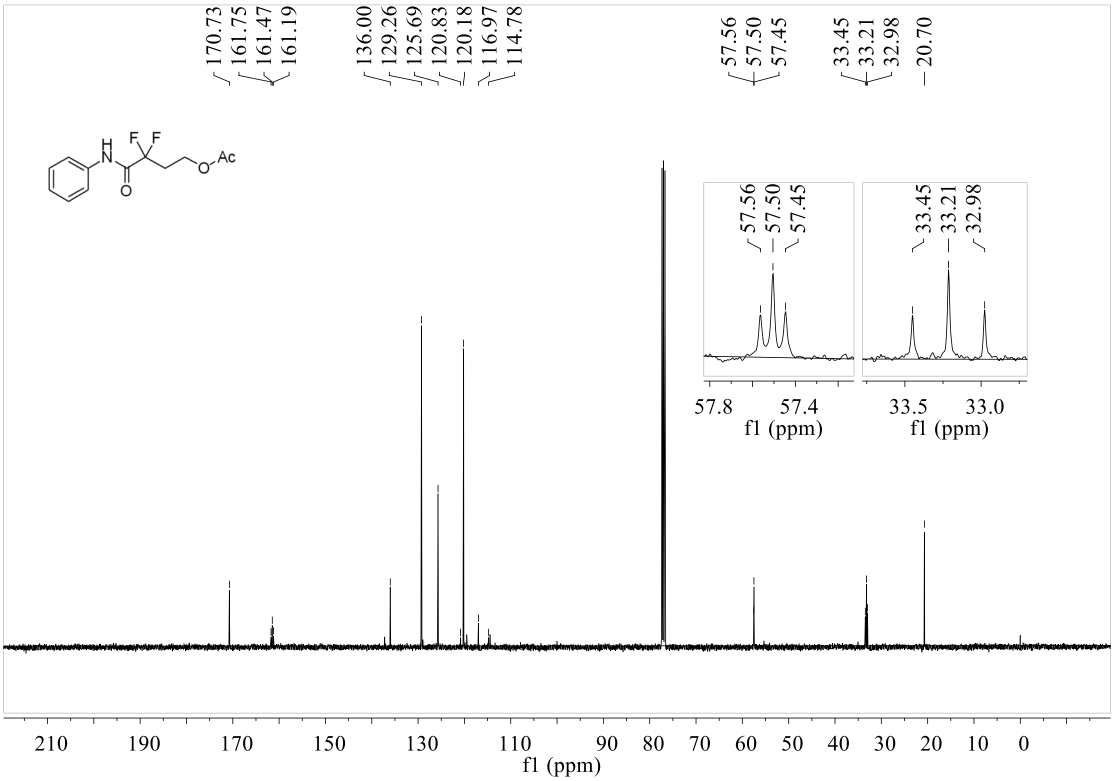

Supplementary Figure 77. ^{13}C NMR (101 MHz, $CDCl_3$) spectrum of **26**

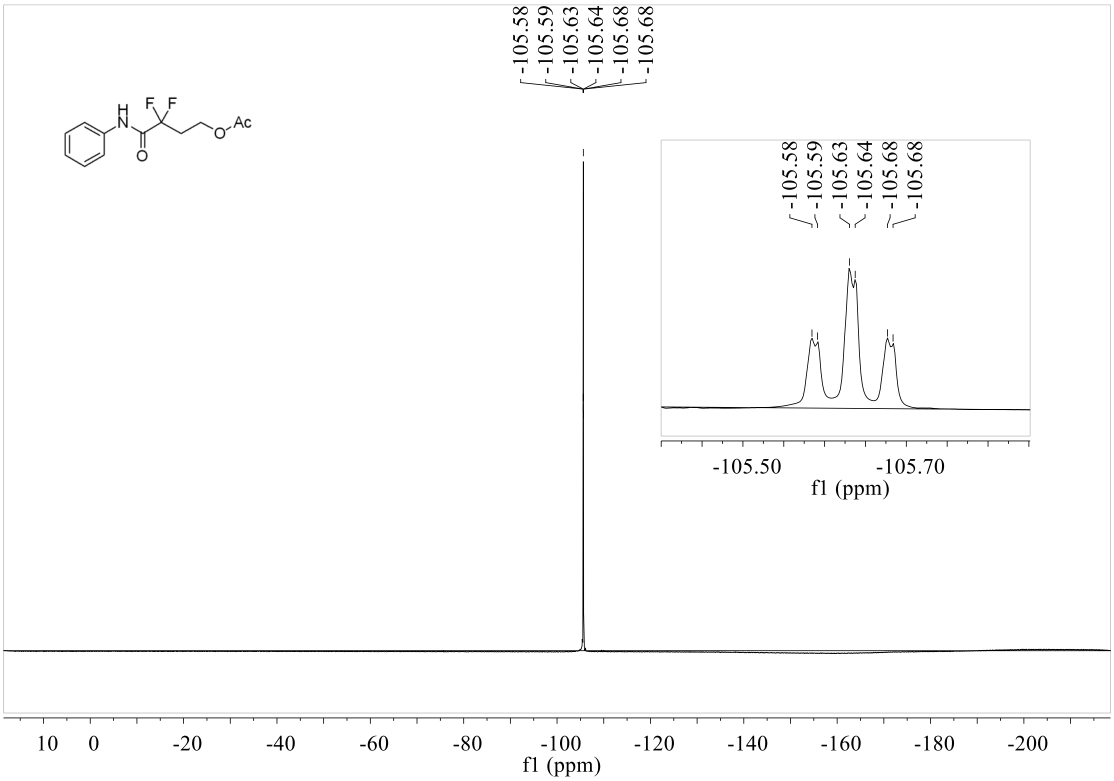

Supplementary Figure 78. ^{19}F NMR (376 MHz, $CDCl_3$) spectrum of **26**

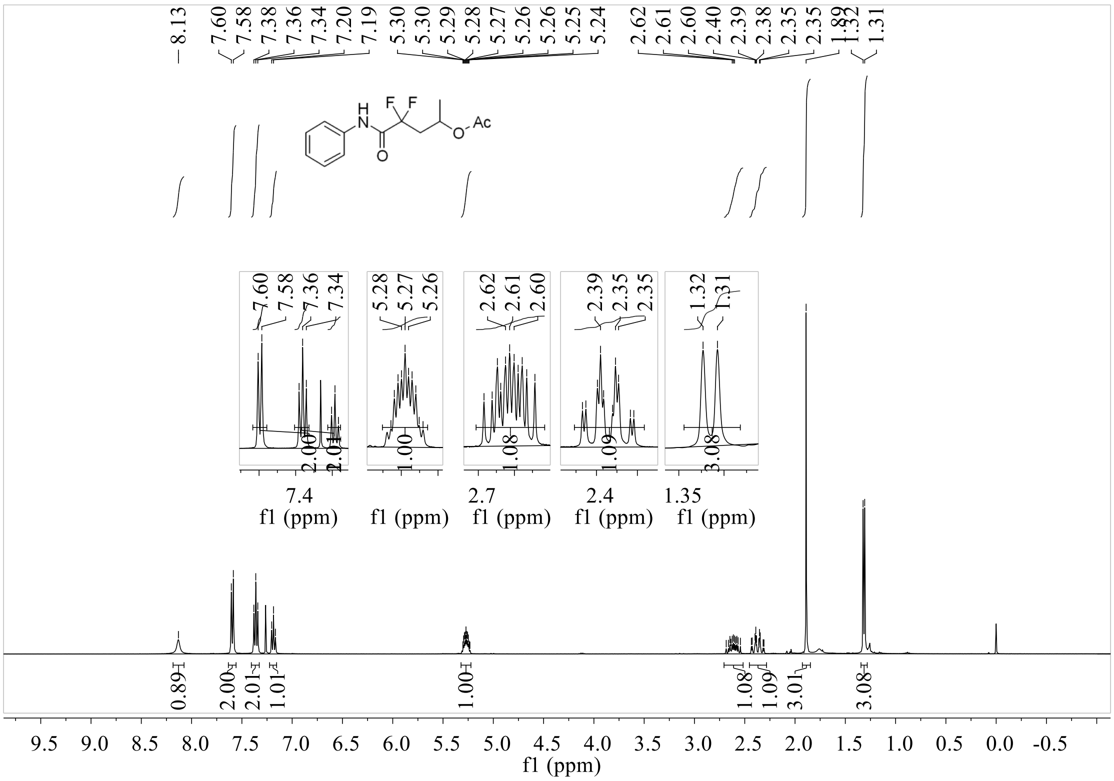

Supplementary Figure 79. ^{1}H NMR (400 MHz, $CDCl_3$) spectrum of **27**

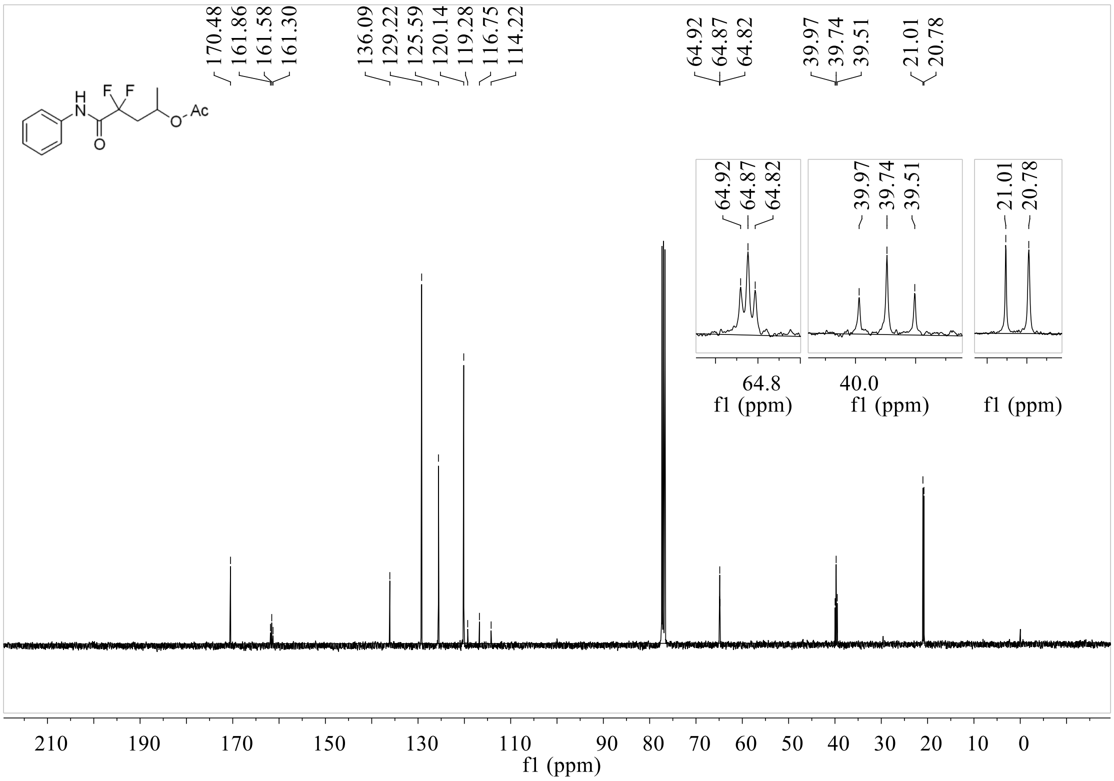

Supplementary Figure 80. ^{13}C NMR (101 MHz, $CDCl_3$) spectrum of **27**

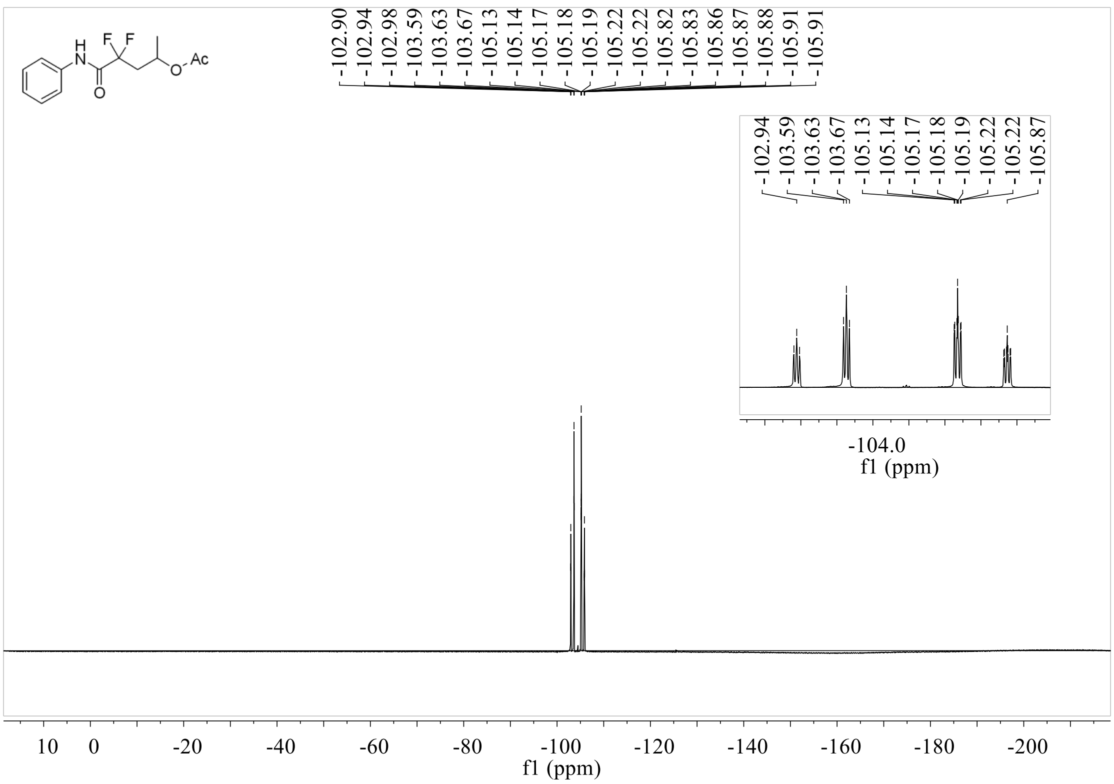

Supplementary Figure 81. ^{19}F NMR (376 MHz, $CDCl_3$) spectrum of **27**

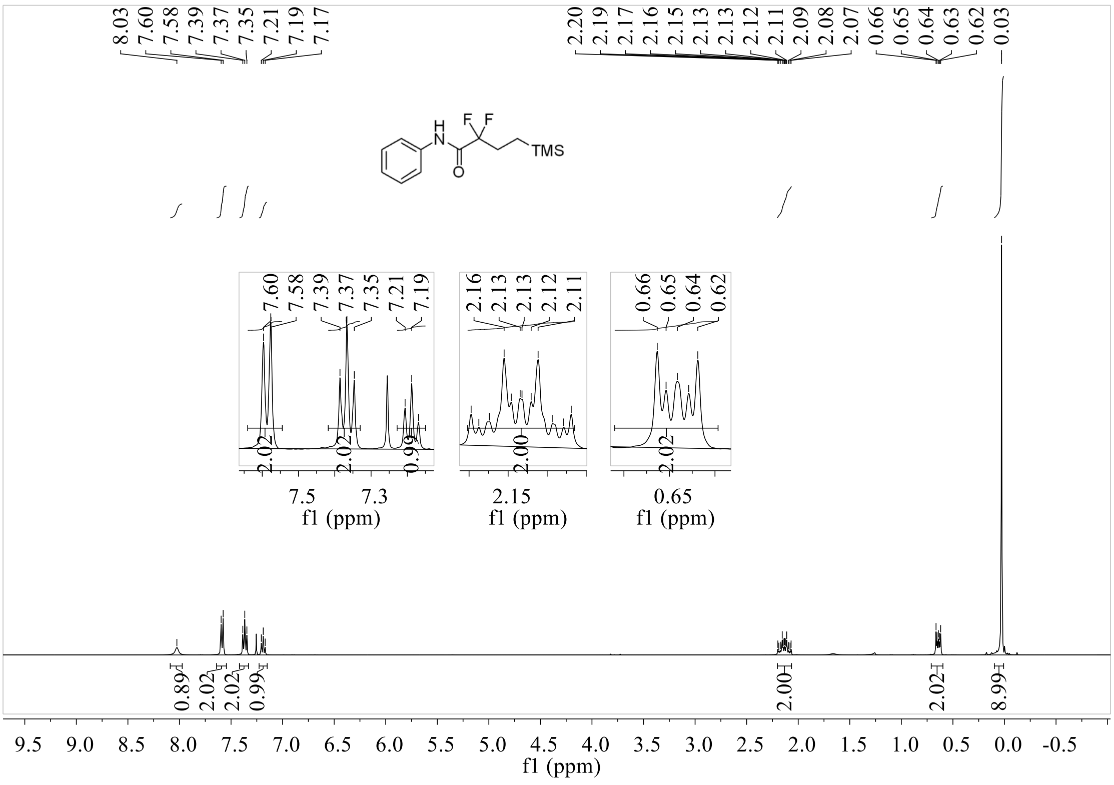

Supplementary Figure 82. ^{1}H NMR (400 MHz, $CDCl_3$) spectrum of **28**

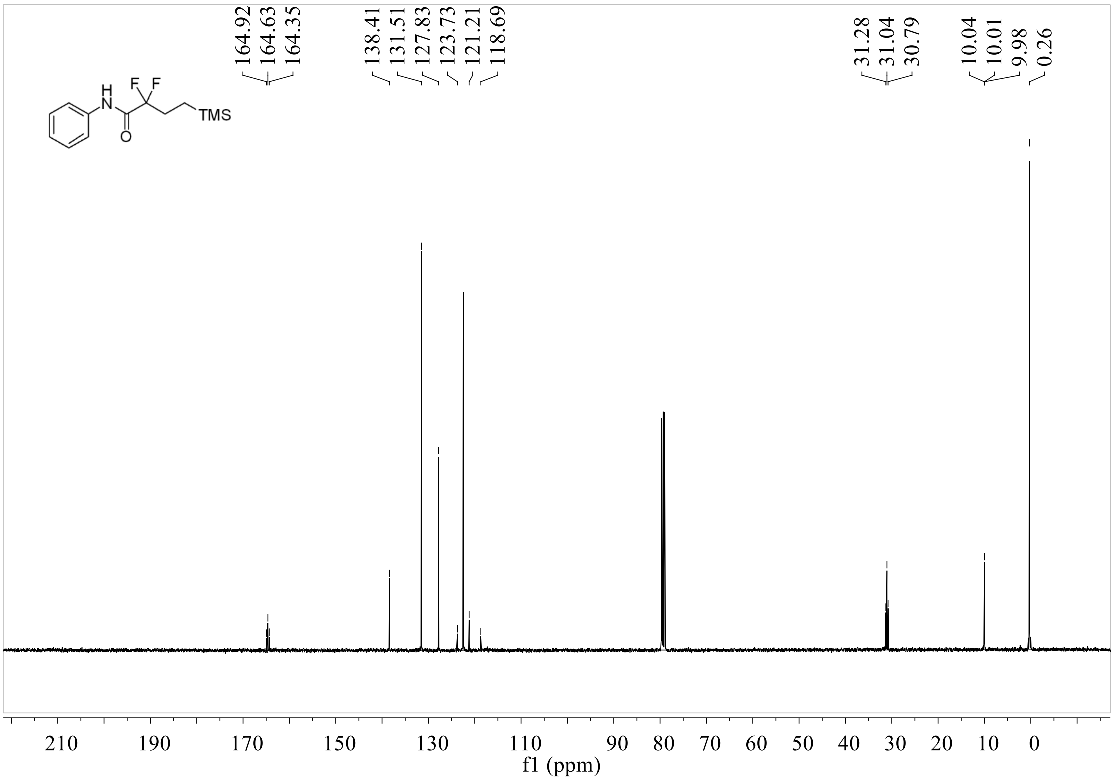

Supplementary Figure 83. ^{13}C NMR (101 MHz, $CDCl_3$) spectrum of **28**

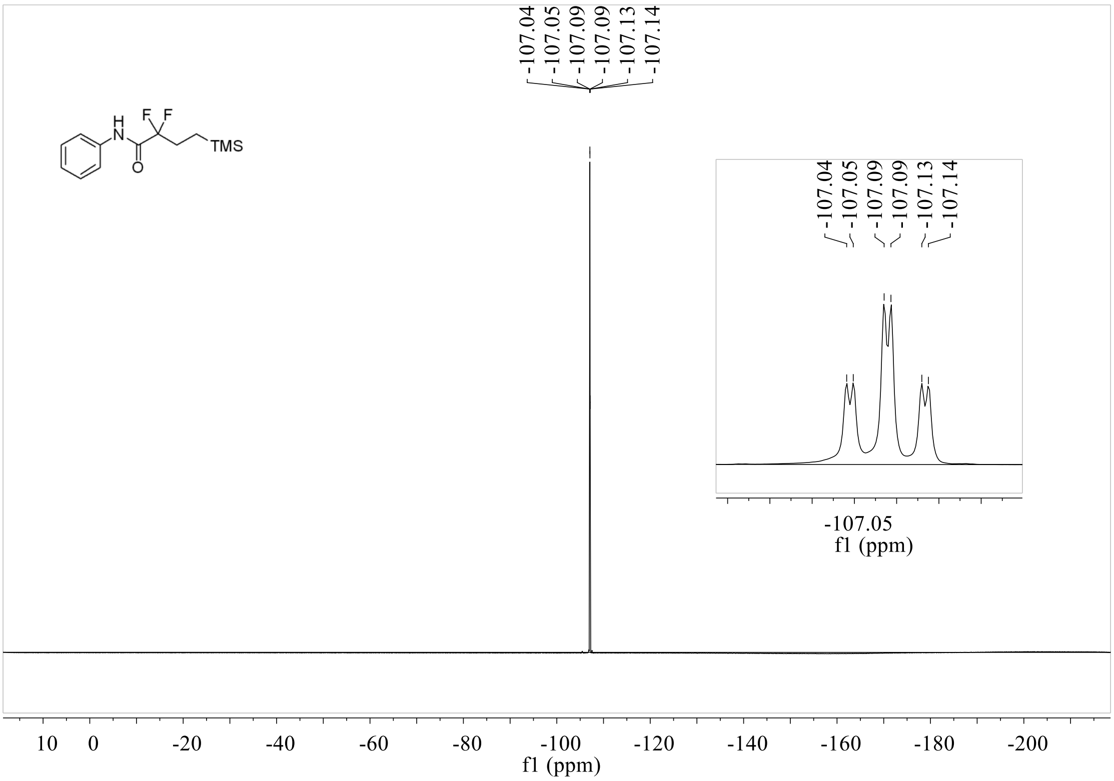

Supplementary Figure 84. ^{19}F NMR (376 MHz, $CDCl_3$) spectrum of **28**

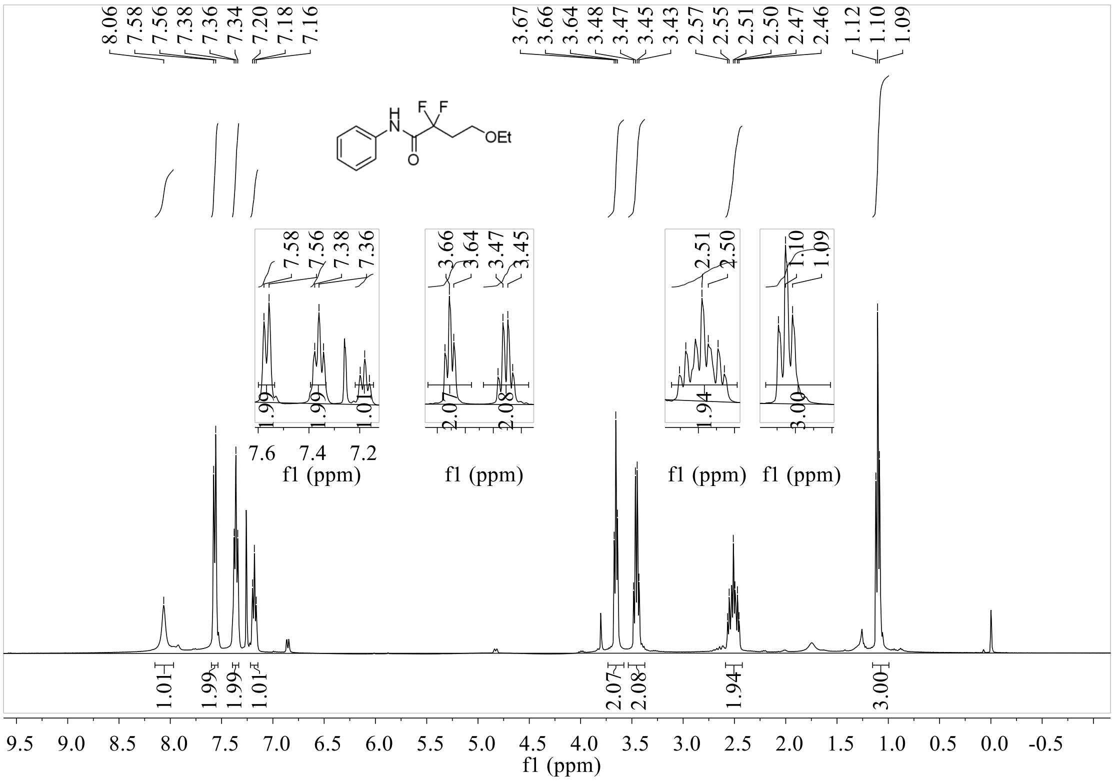

Supplementary Figure 85. ^{1}H NMR (400 MHz, $CDCl_3$) spectrum of **29**

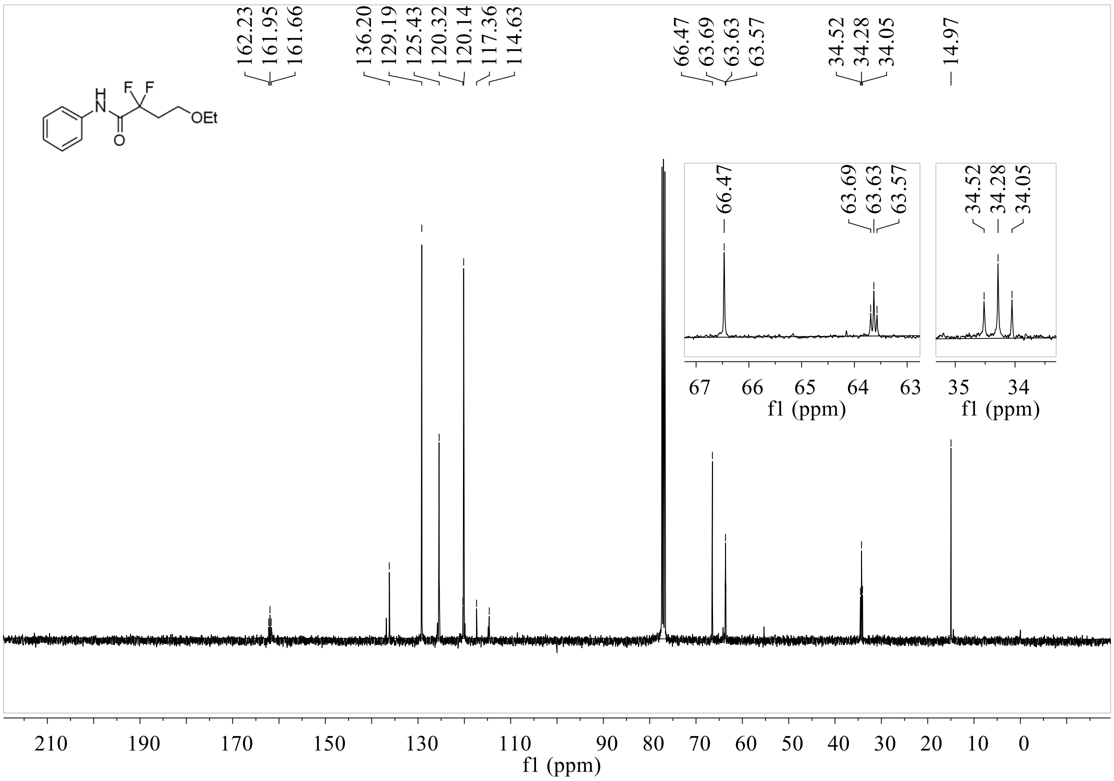

Supplementary Figure 86. ^{13}C NMR (101 MHz, $CDCl_3$) spectrum of **29**

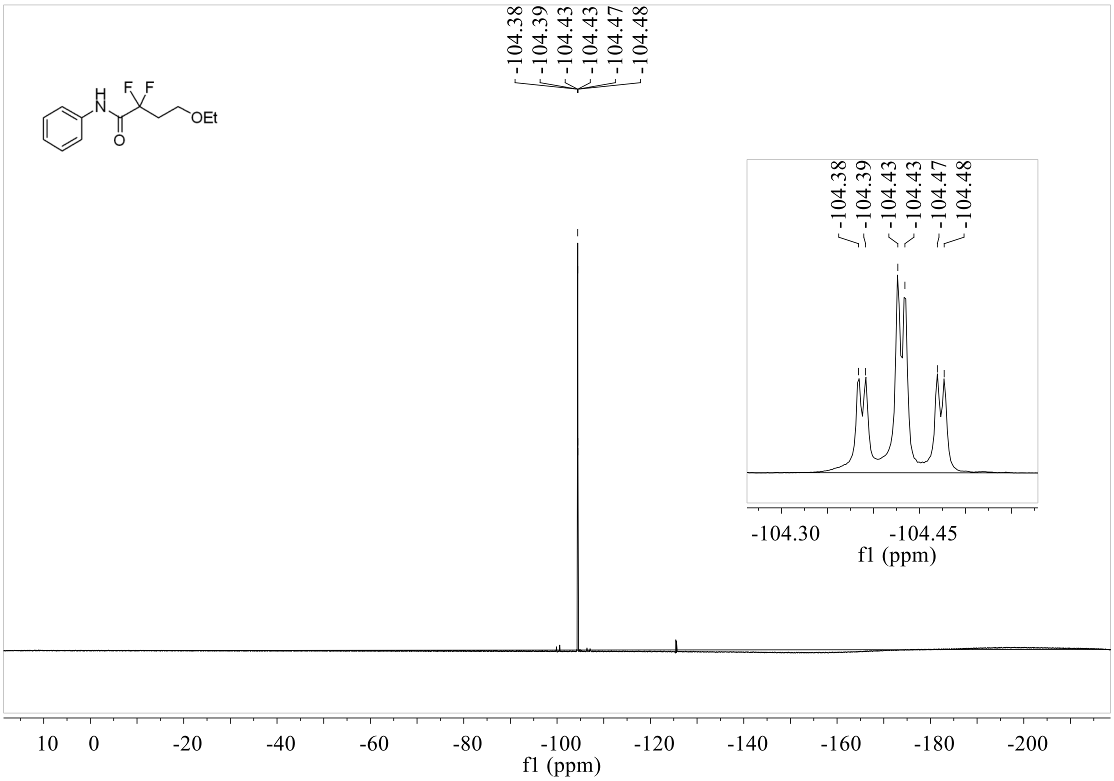

Supplementary Figure 87. ^{19}F NMR (376 MHz, $CDCl_3$) spectrum of **29**

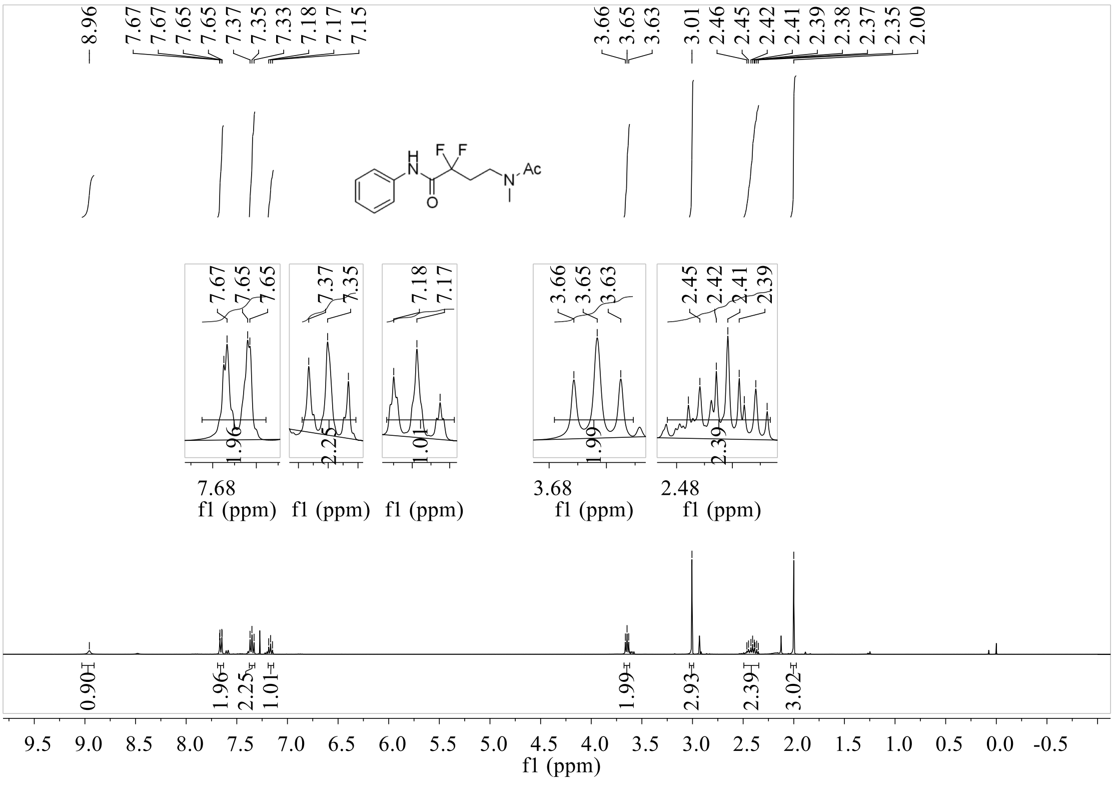

Supplementary Figure 88. ^{1}H NMR (400 MHz, $CDCl_3$) spectrum of **30**

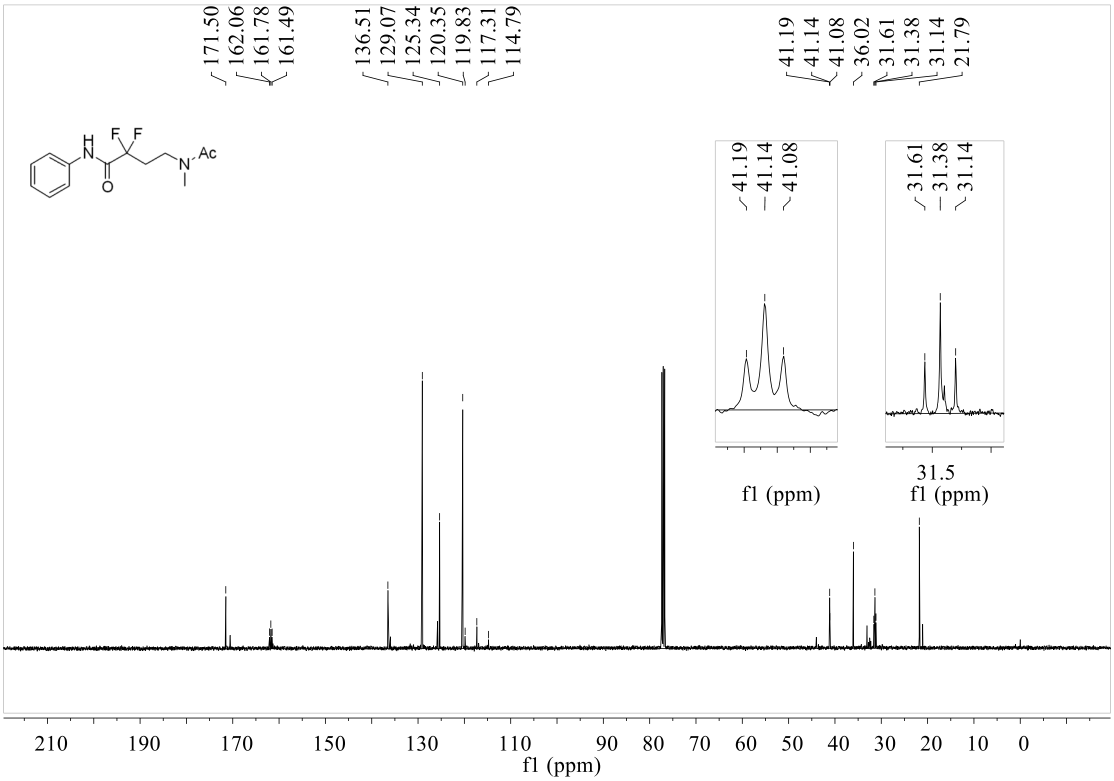

Supplementary Figure 89. ^{13}C NMR (101 MHz, $CDCl_3$) spectrum of **30**

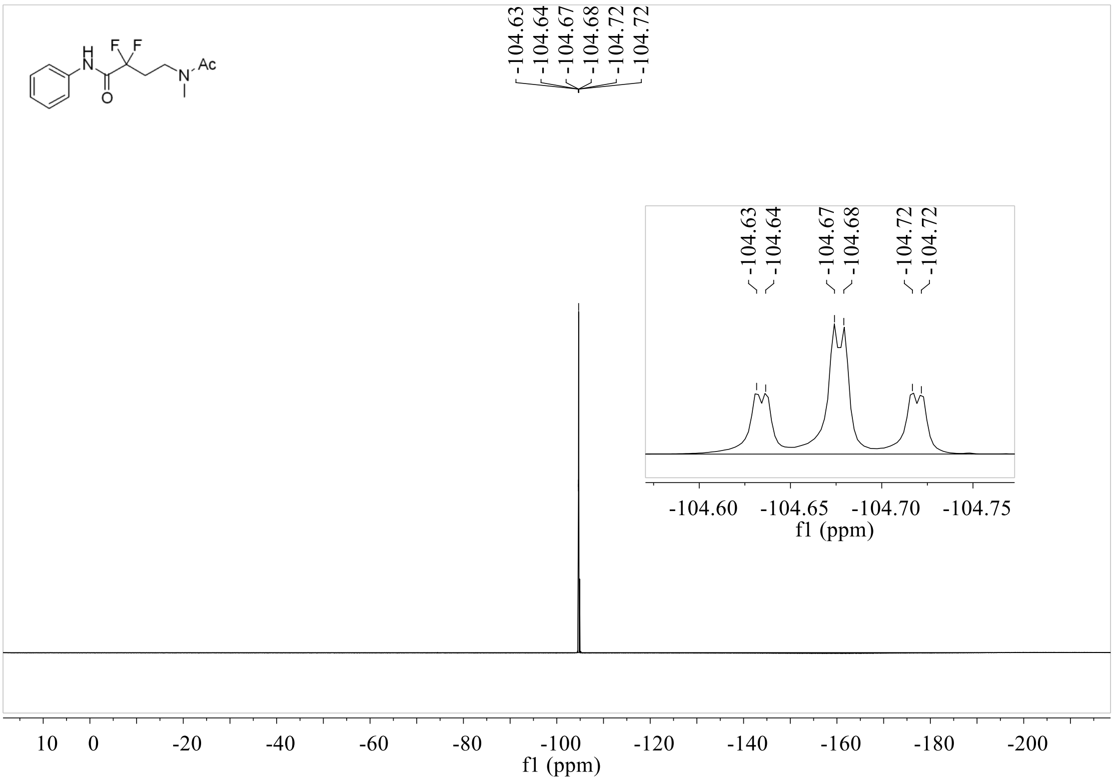

Supplementary Figure 90. ^{19}F NMR (376 MHz, $CDCl_3$) spectrum of **30**

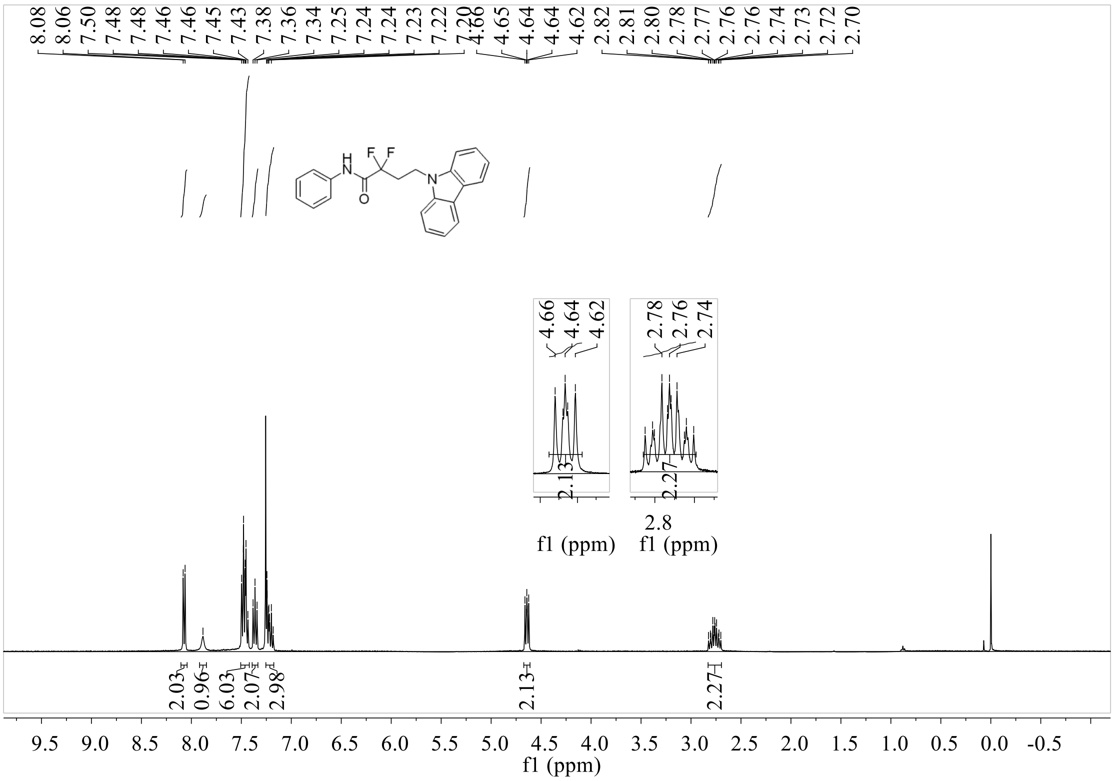

Supplementary Figure 91. ^{1}H NMR (400 MHz, $CDCl_3$) spectrum of **31**

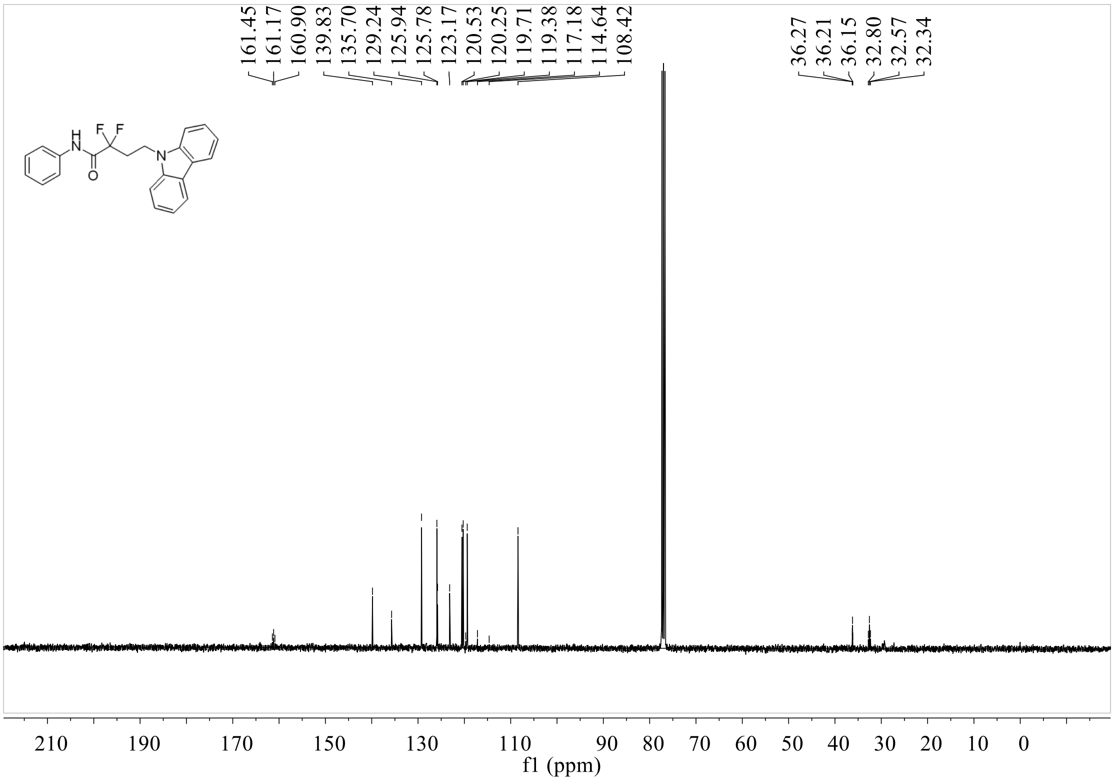

Supplementary Figure 92. ^{13}C NMR (101 MHz, $CDCl_3$) spectrum of **31**

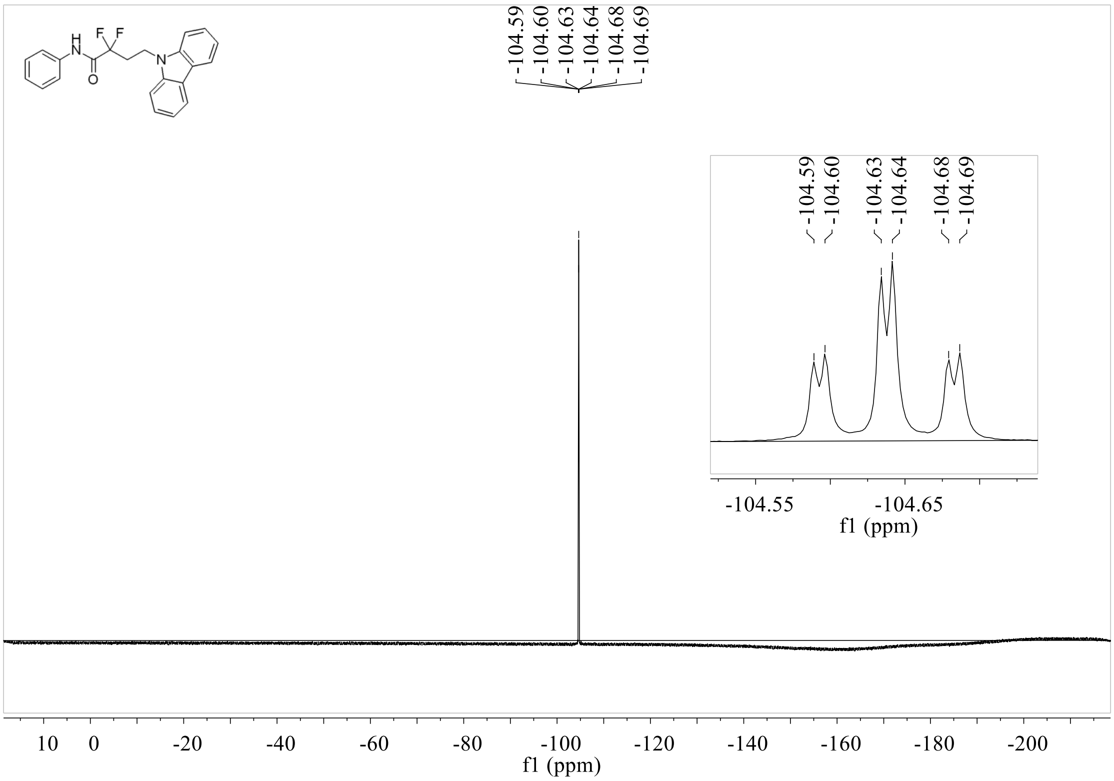

Supplementary Figure 93. ^{19}F NMR (376 MHz, $CDCl_3$) spectrum of **31**

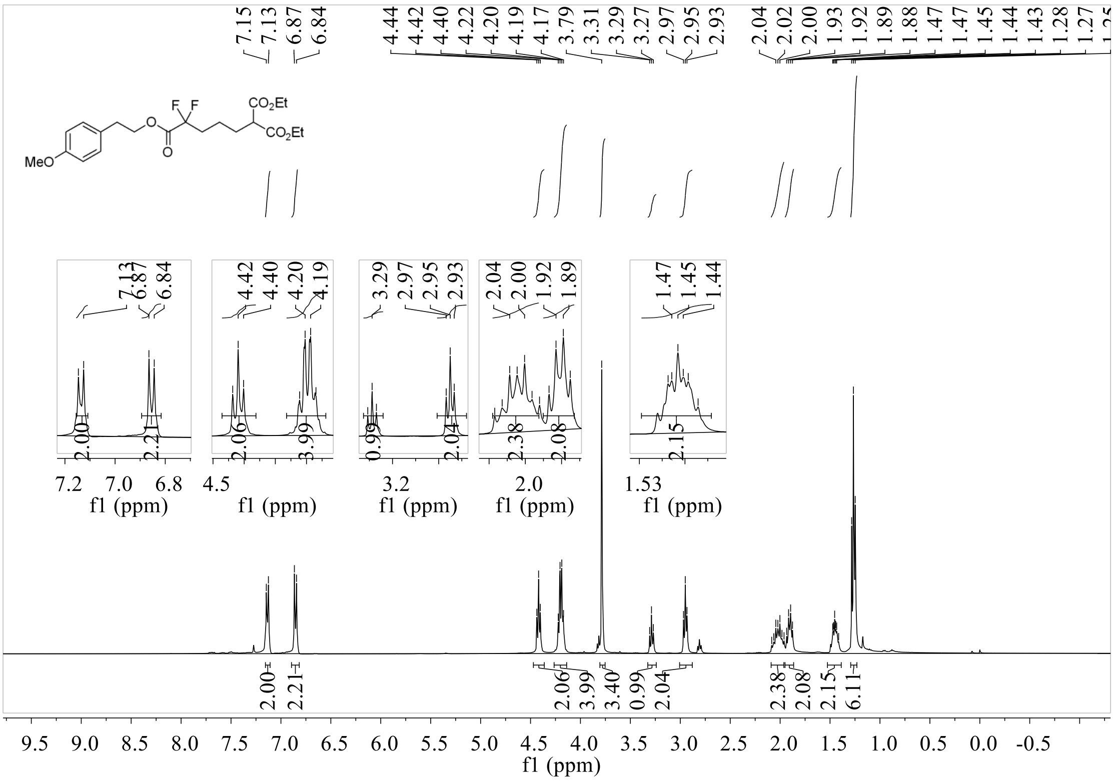

Supplementary Figure 94. ^{1}H NMR (400 MHz, $CDCl_3$) spectrum of **32**

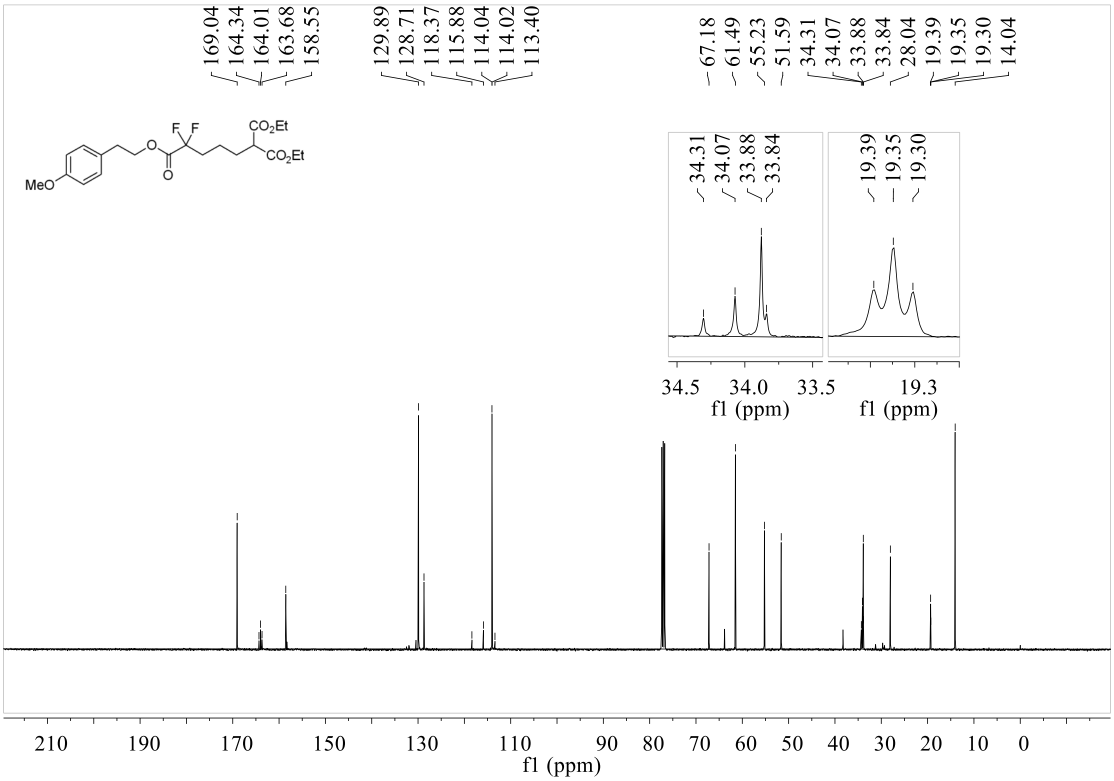

Supplementary Figure 95. ^{13}C NMR (101 MHz, $CDCl_3$) spectrum of **32**

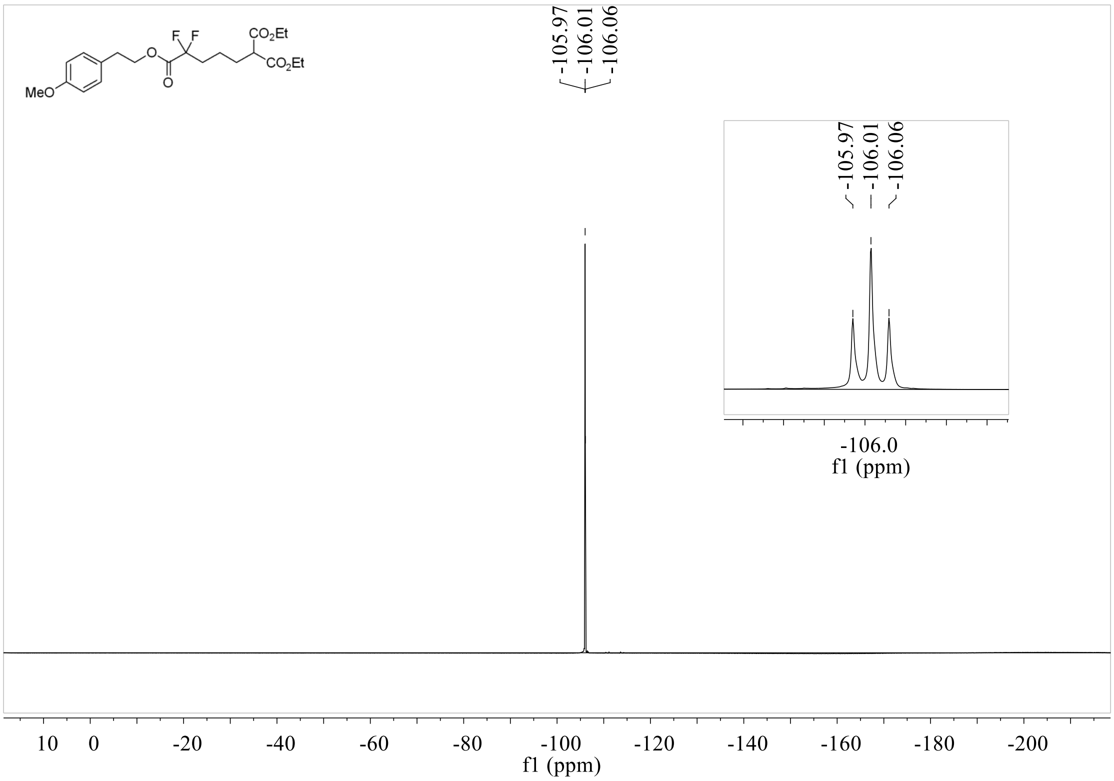

Supplementary Figure 96. ^{19}F NMR (376 MHz, $CDCl_3$) spectrum of **32**

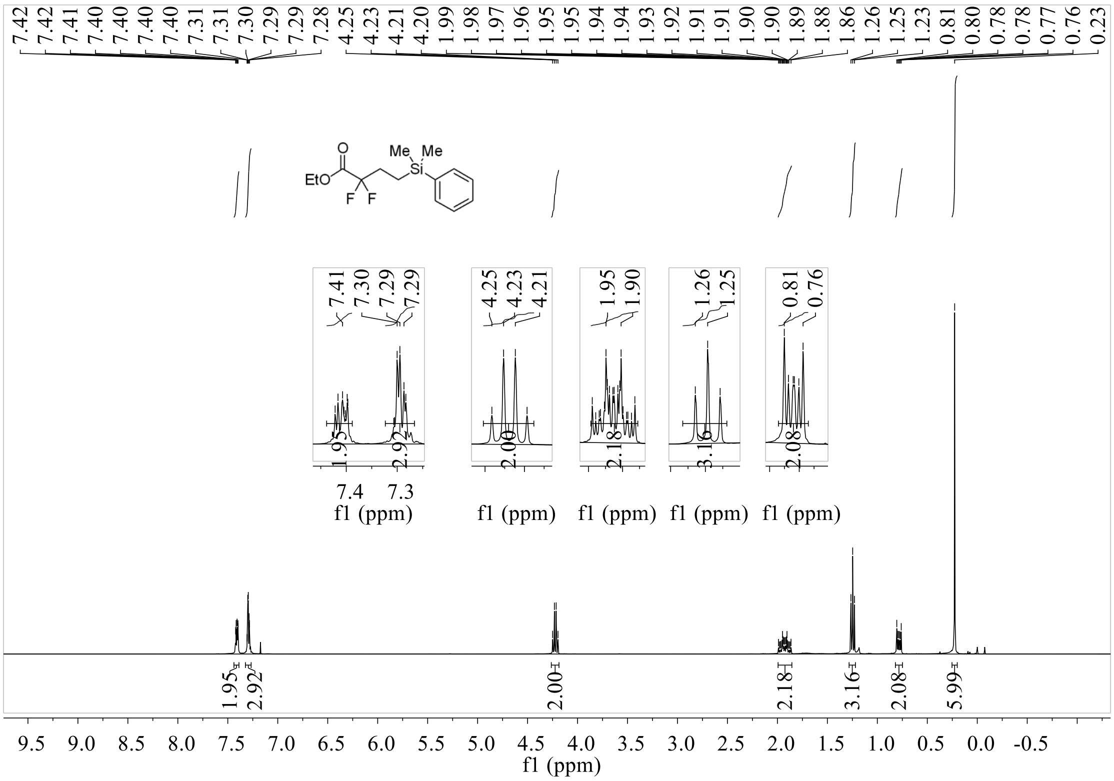

Supplementary Figure 97. ^{1}H NMR (400 MHz, $CDCl_3$) spectrum of **33**

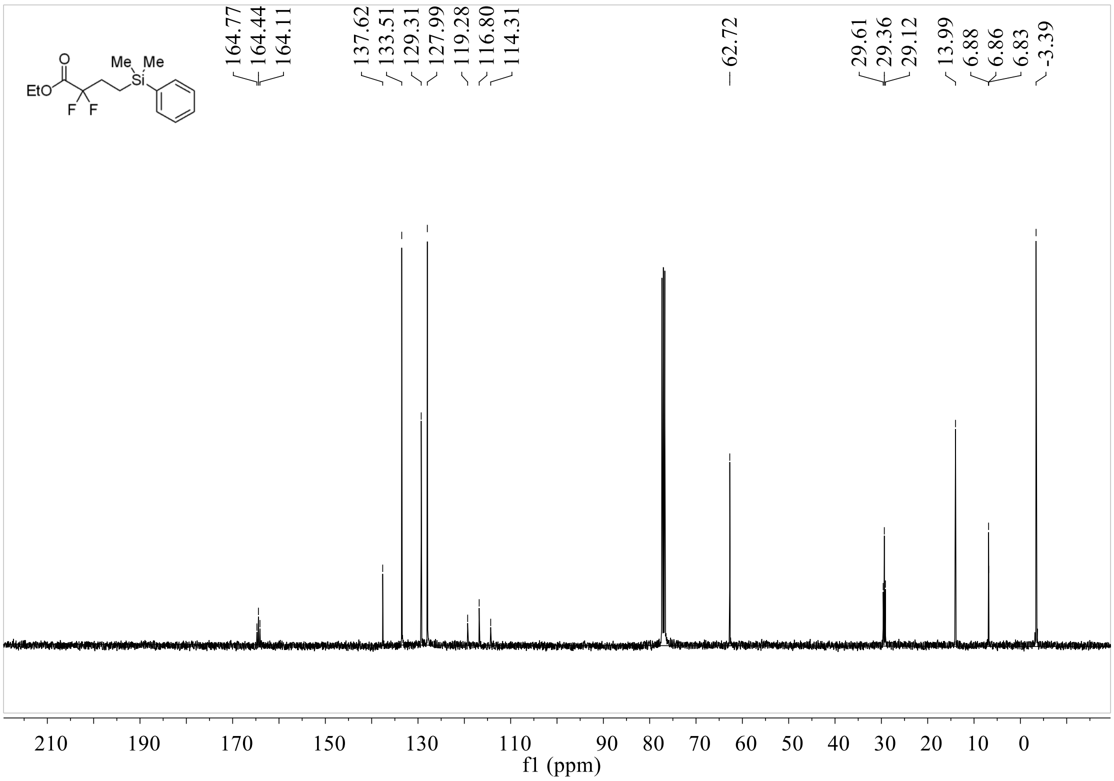

Supplementary Figure 98. ^{13}C NMR (101 MHz, $CDCl_3$) spectrum of **33**

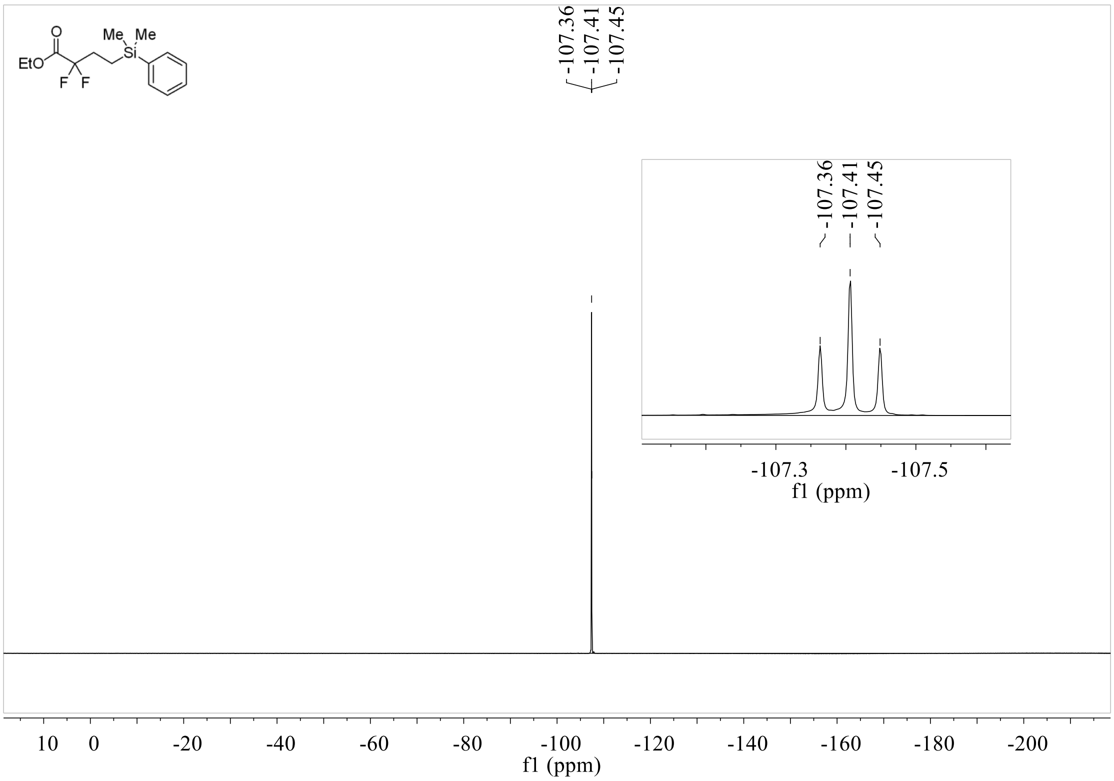

Supplementary Figure 99. ^{19}F NMR (376 MHz, $CDCl_3$) spectrum of **33**

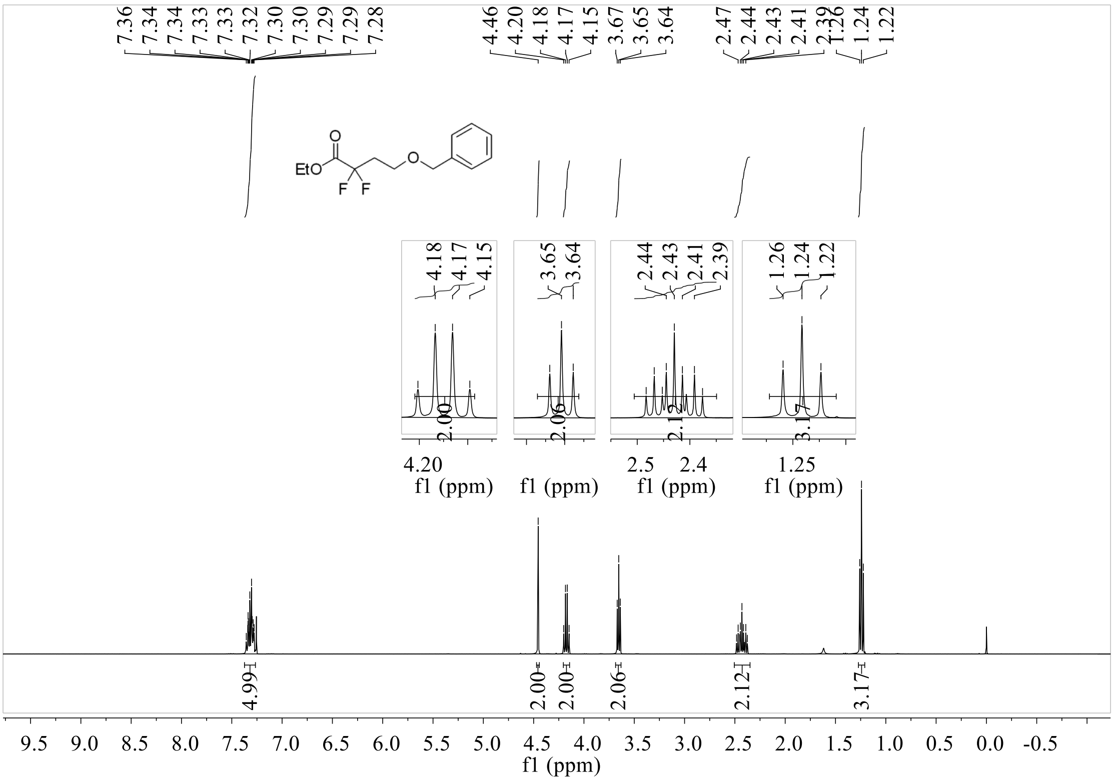

Supplementary Figure 100. ^{1}H NMR (400 MHz, $CDCl_3$) spectrum of **34**

^{13}C NMR (101 MHz, $CDCl_3$) spectrum of **ethyl 4-(benzyloxy)-2,2-difluorobutanoate (34)**

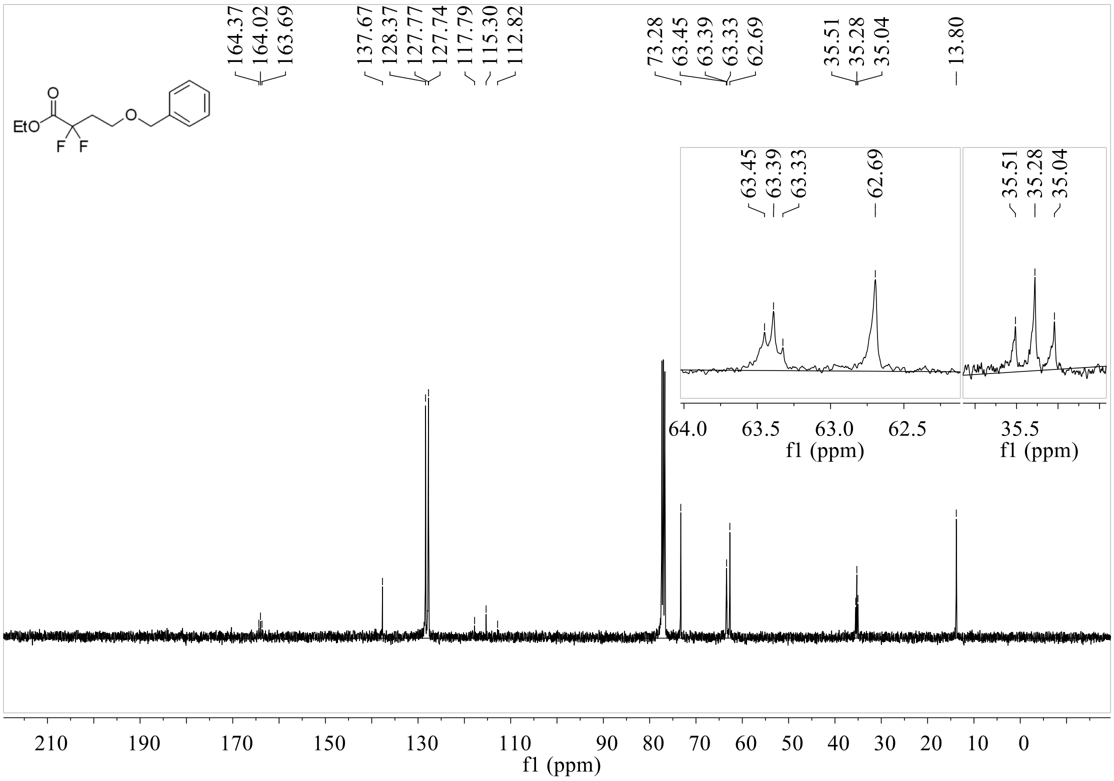

Supplementary Figure 101. ^{13}C NMR (101 MHz, $CDCl_3$) spectrum of **34**

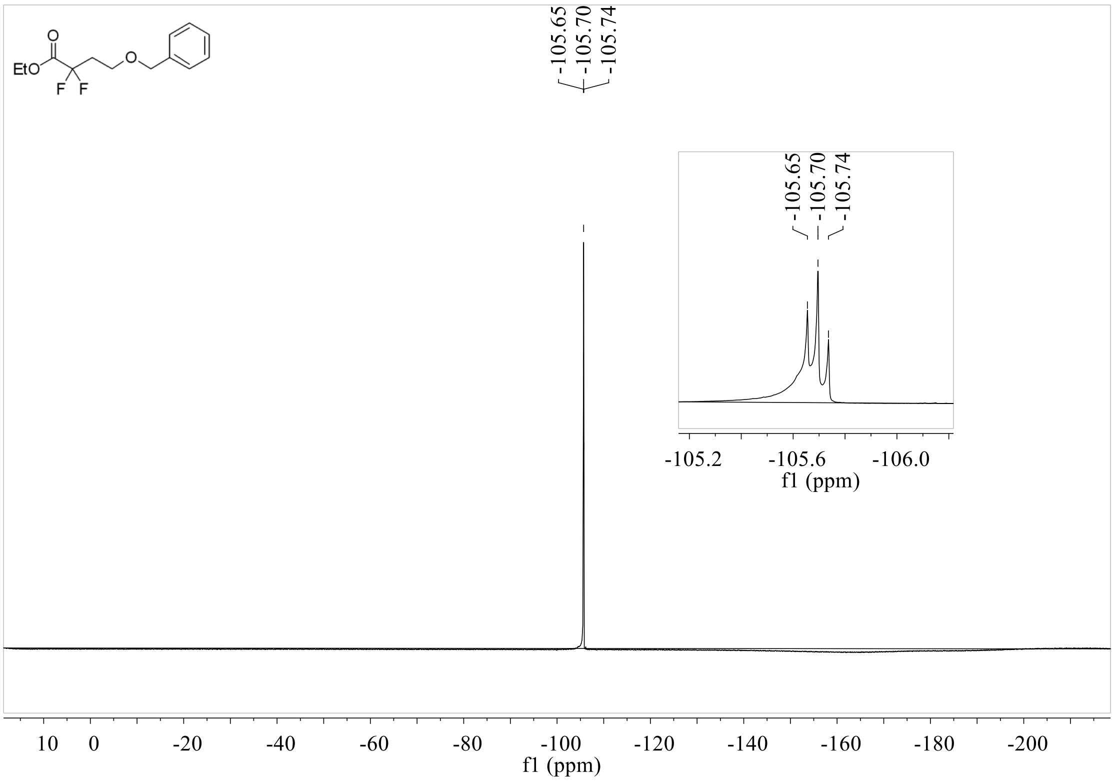

Supplementary Figure 102. ^{19}F NMR (376 MHz, $CDCl_3$) spectrum of **34**

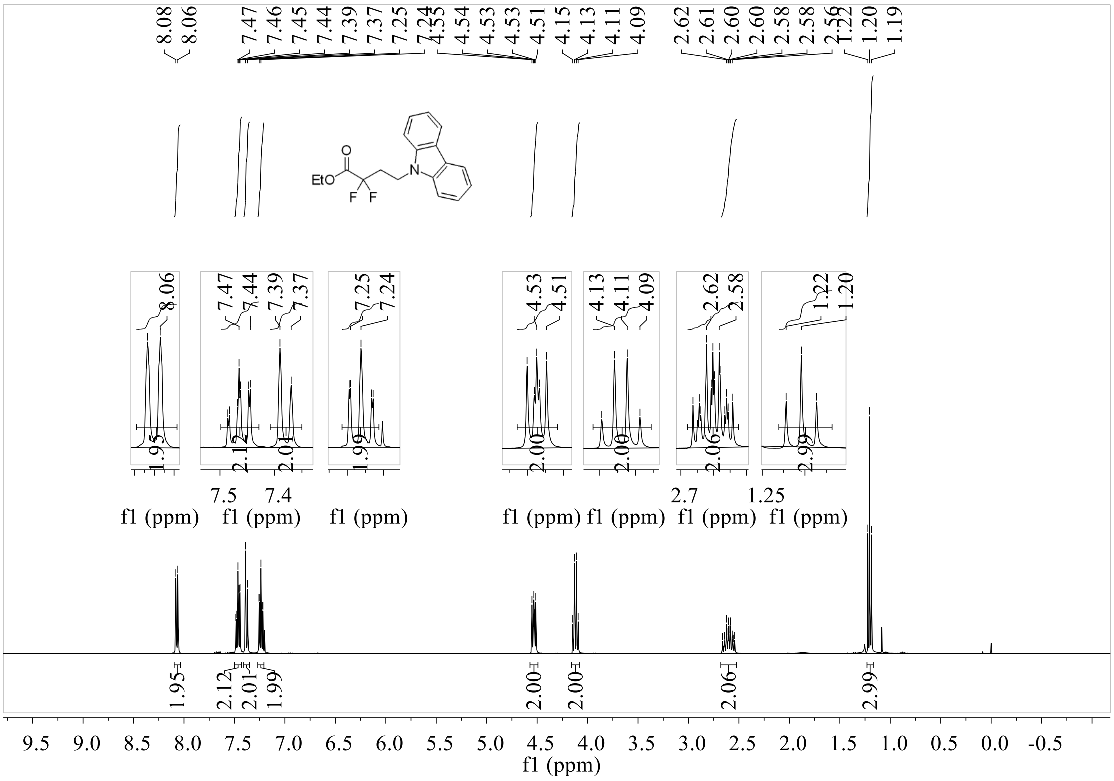

Supplementary Figure 103. ^{1}H NMR (400 MHz, $CDCl_3$) spectrum of **35**

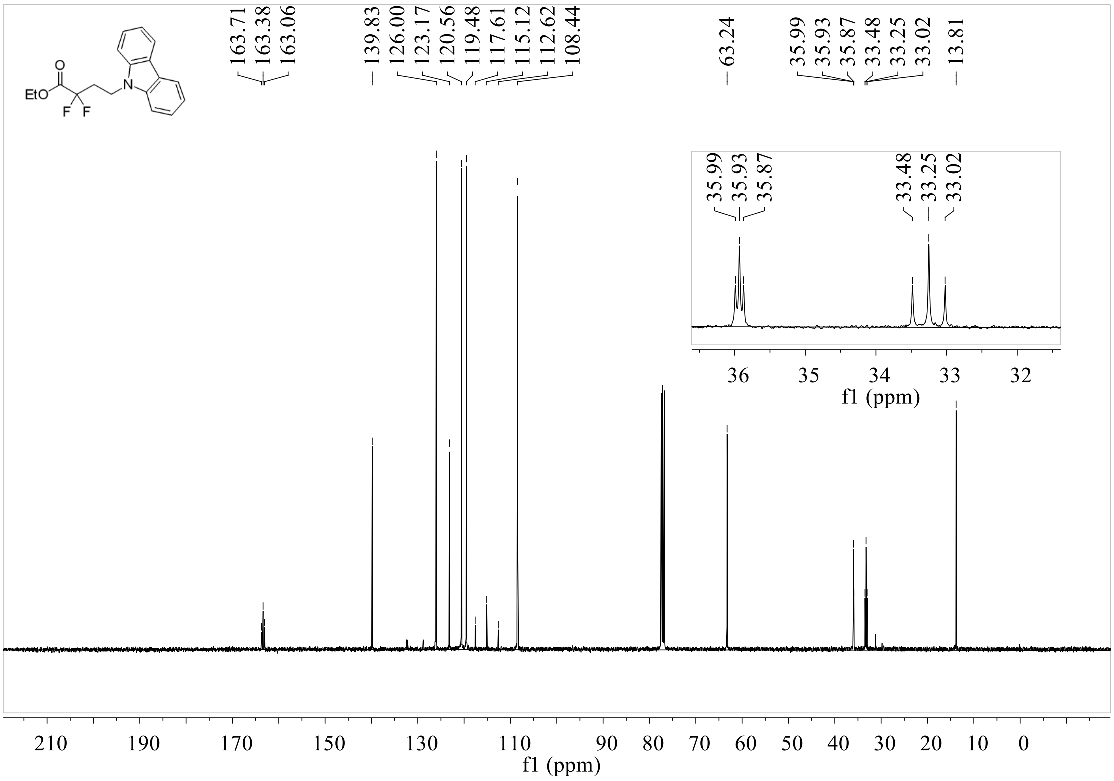

Supplementary Figure 104. ^{13}C NMR (101 MHz, $CDCl_3$) spectrum of **35**

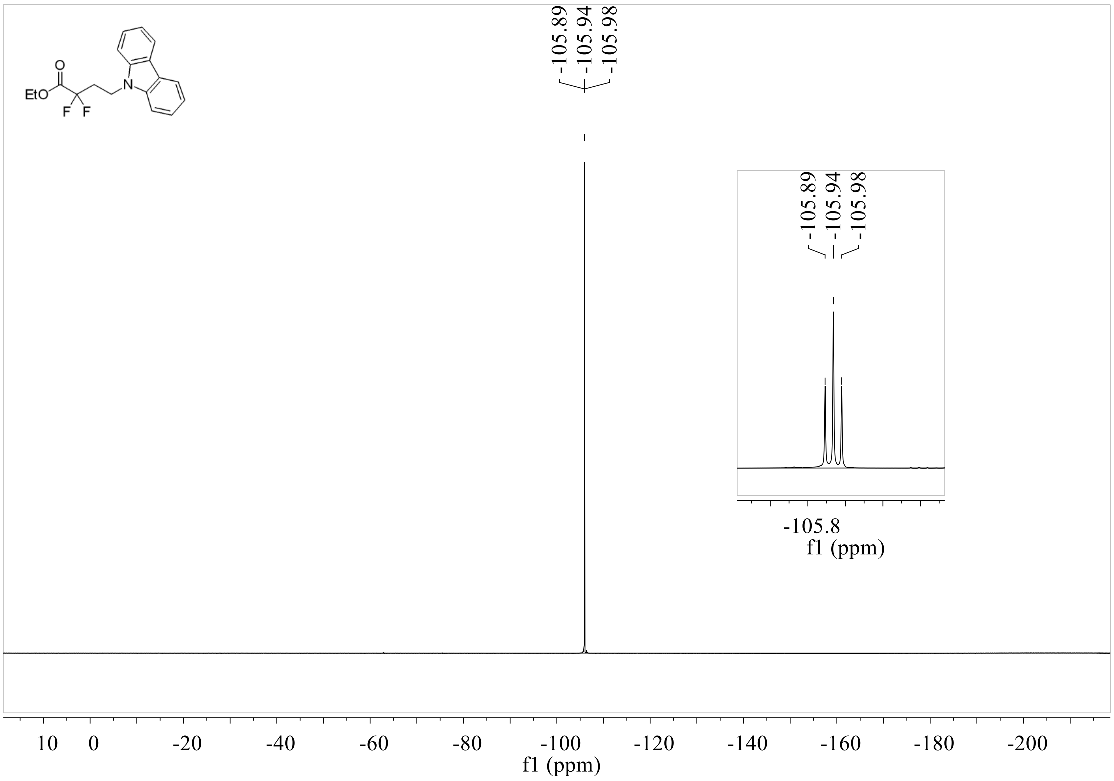

Supplementary Figure 105. ^{19}F NMR (376 MHz, $CDCl_3$) spectrum of **35**

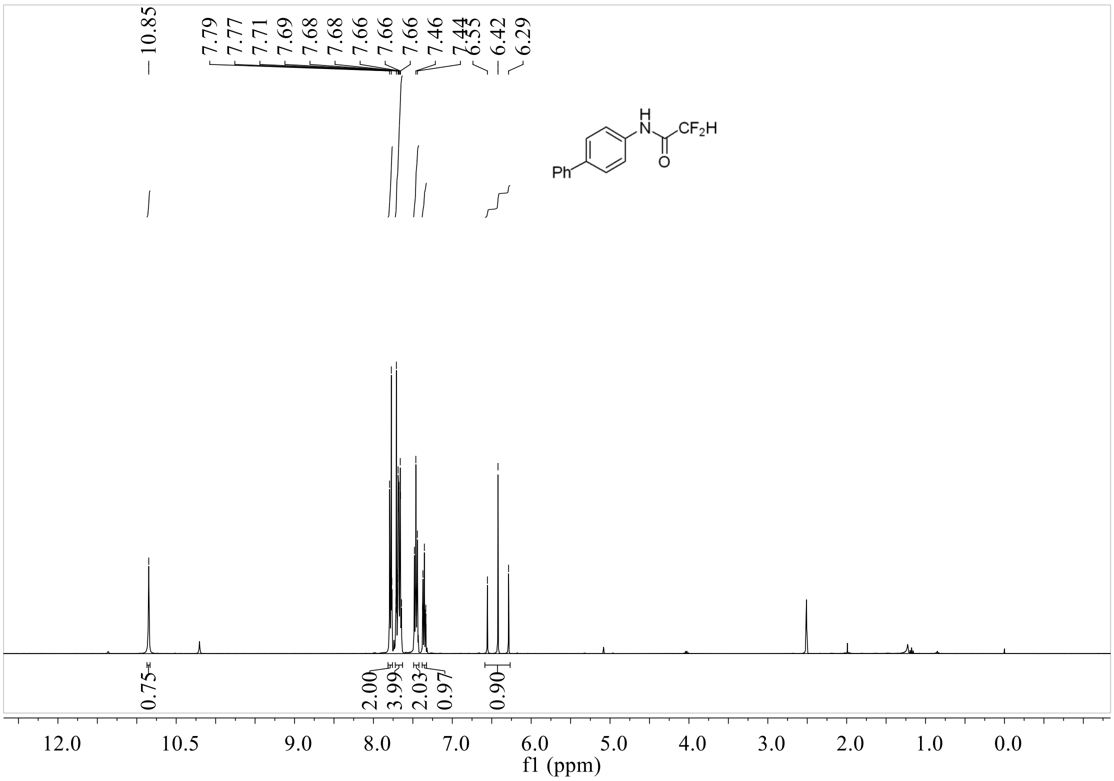

Supplementary Figure 106. ^{1}H NMR (400 MHz, d_6-DMSO) spectrum of **36**

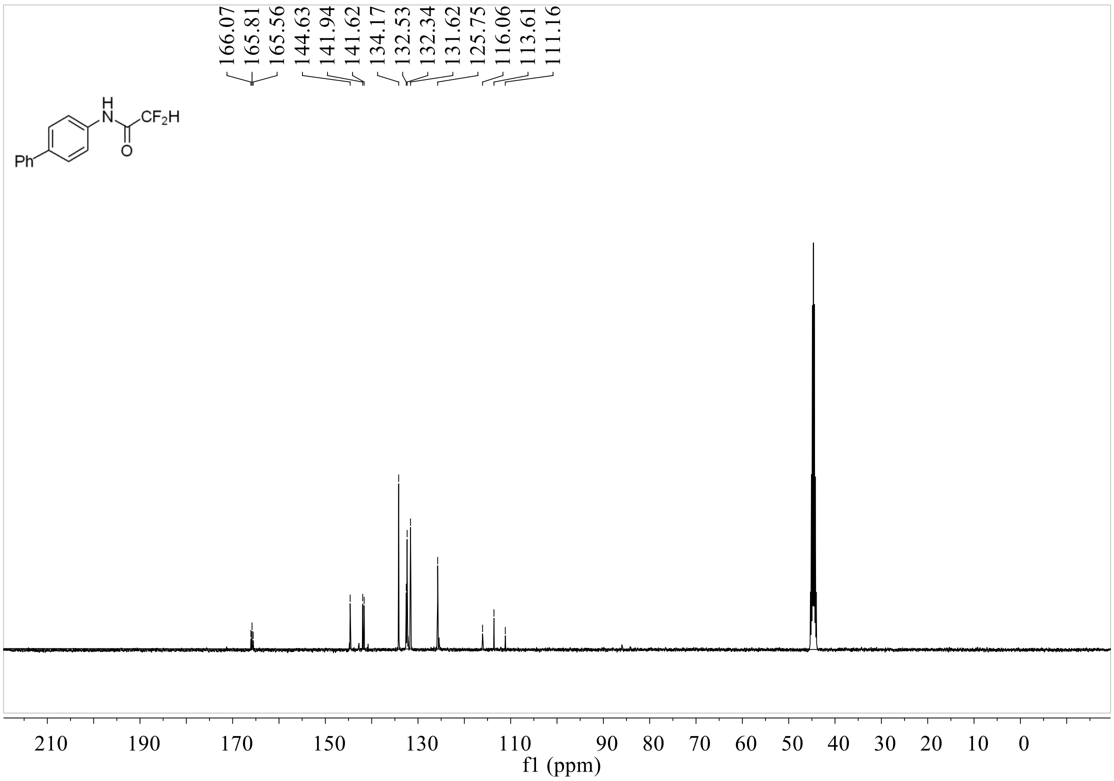

Supplementary Figure 107. ^{13}C NMR (101 MHz, d_6-DMSO) spectrum of **36**

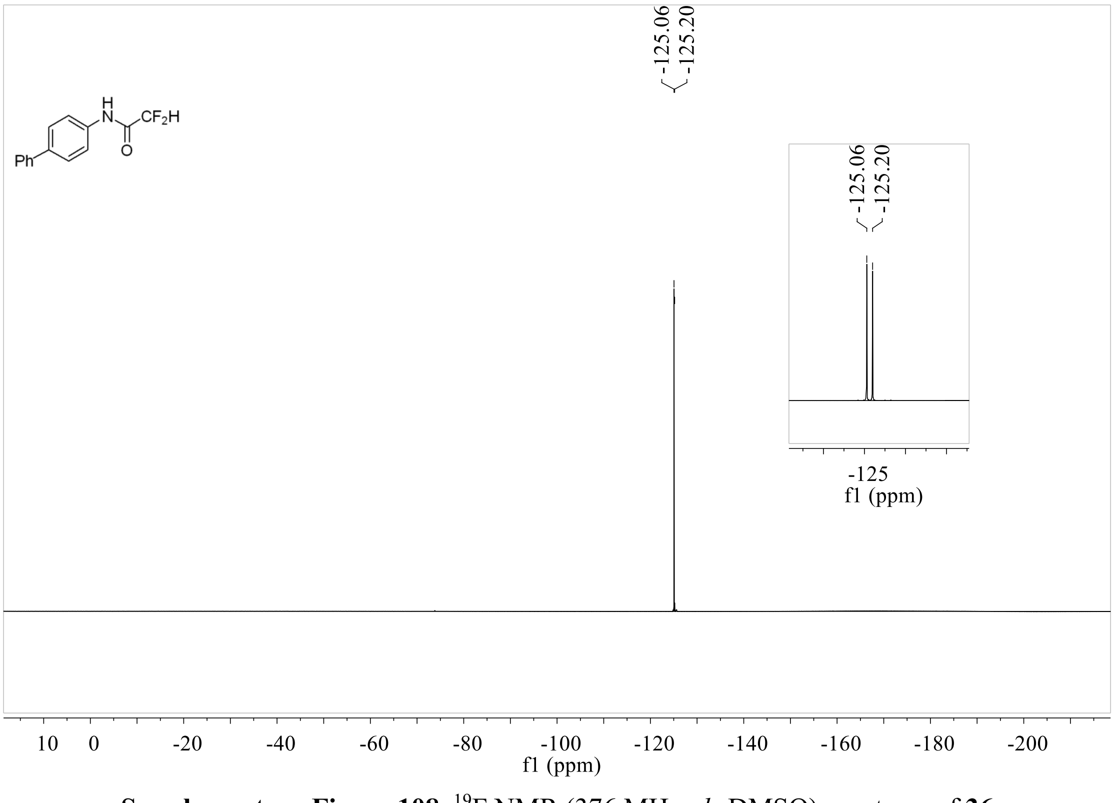

Supplementary Figure 108. ^{19}F NMR (376 MHz, d_6-DMSO) spectrum of **36**

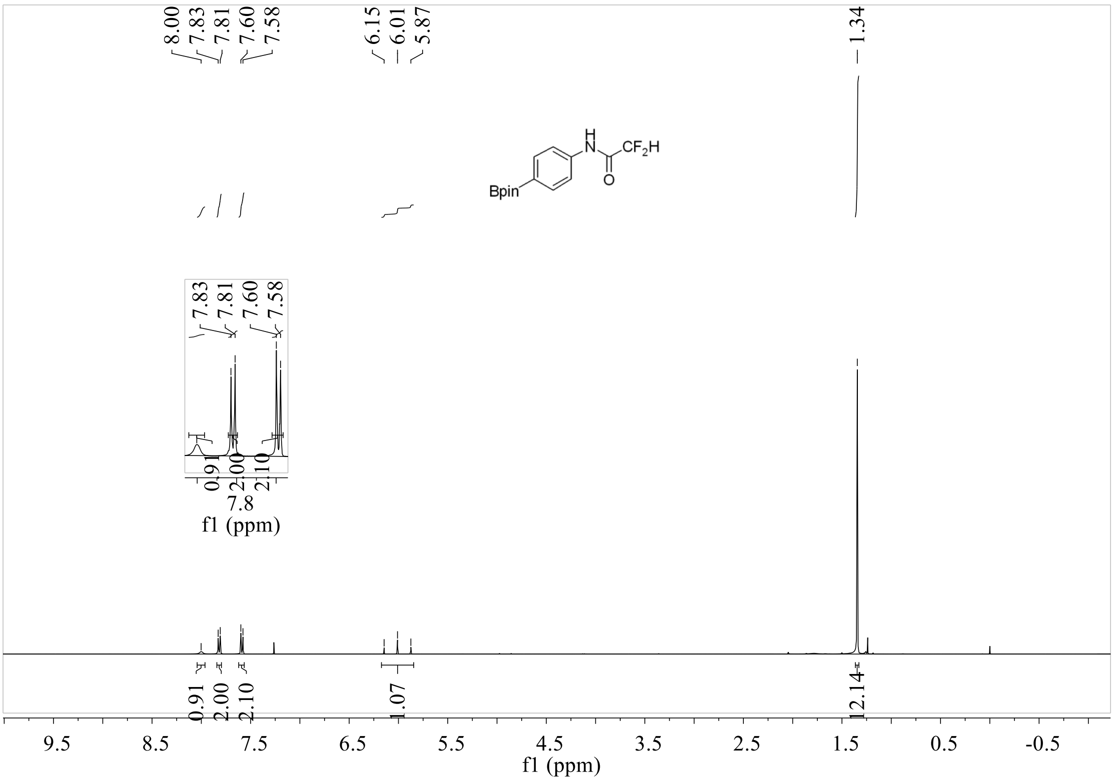

Supplementary Figure 109. ^{1}H NMR (400 MHz, $CDCl_3$) spectrum of **37**

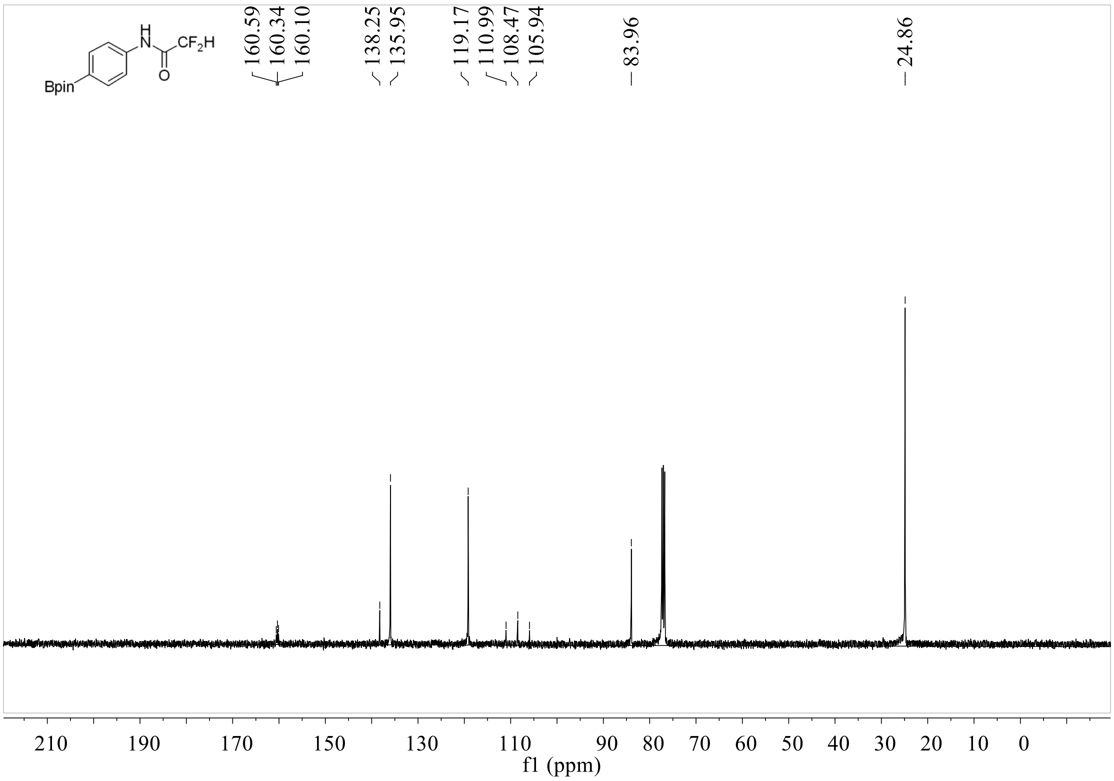

Supplementary Figure 110. ^{13}C NMR (101 MHz, $CDCl_3$) spectrum of **37**

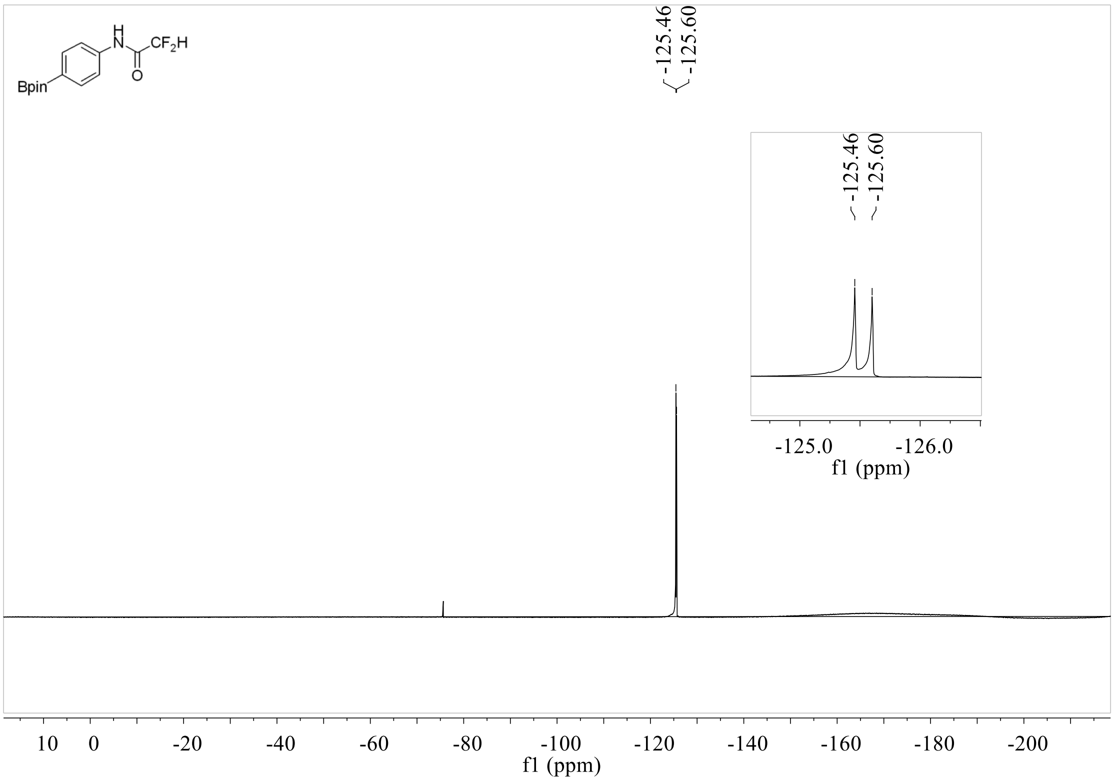

Supplementary Figure 111. ^{19}F NMR (376 MHz, $CDCl_3$) spectrum of **37**

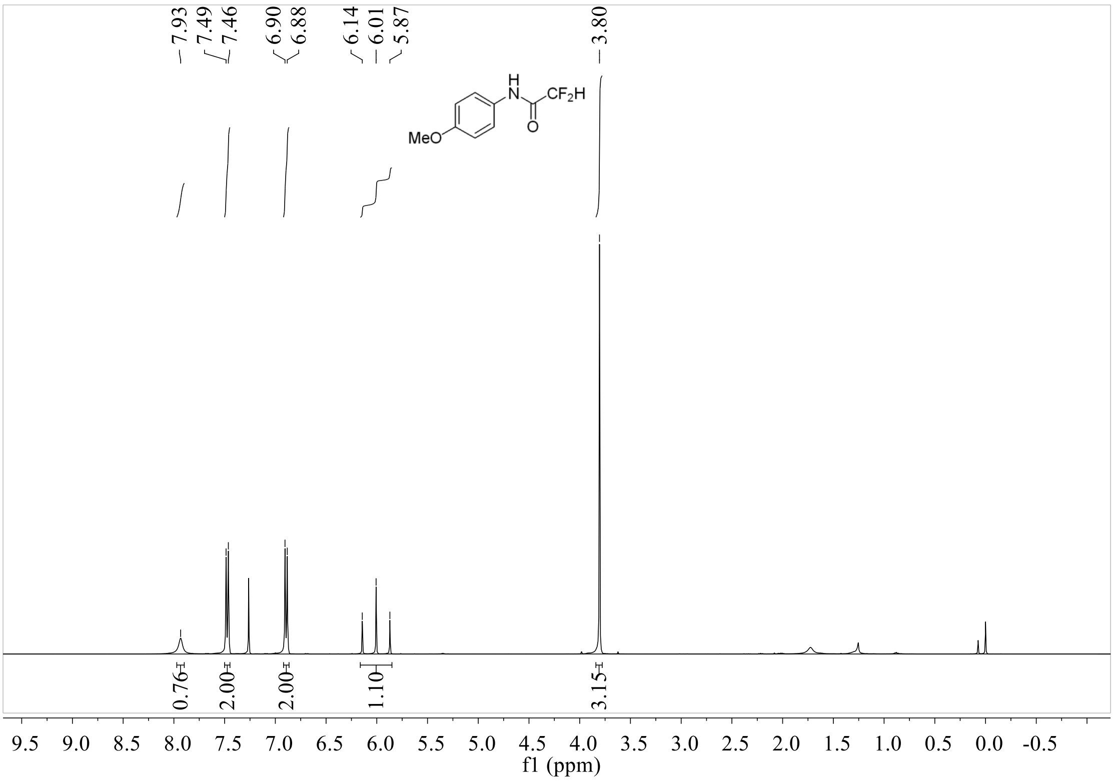

Supplementary Figure 112. ^{1}H NMR (400 MHz, $CDCl_3$) spectrum of **38**

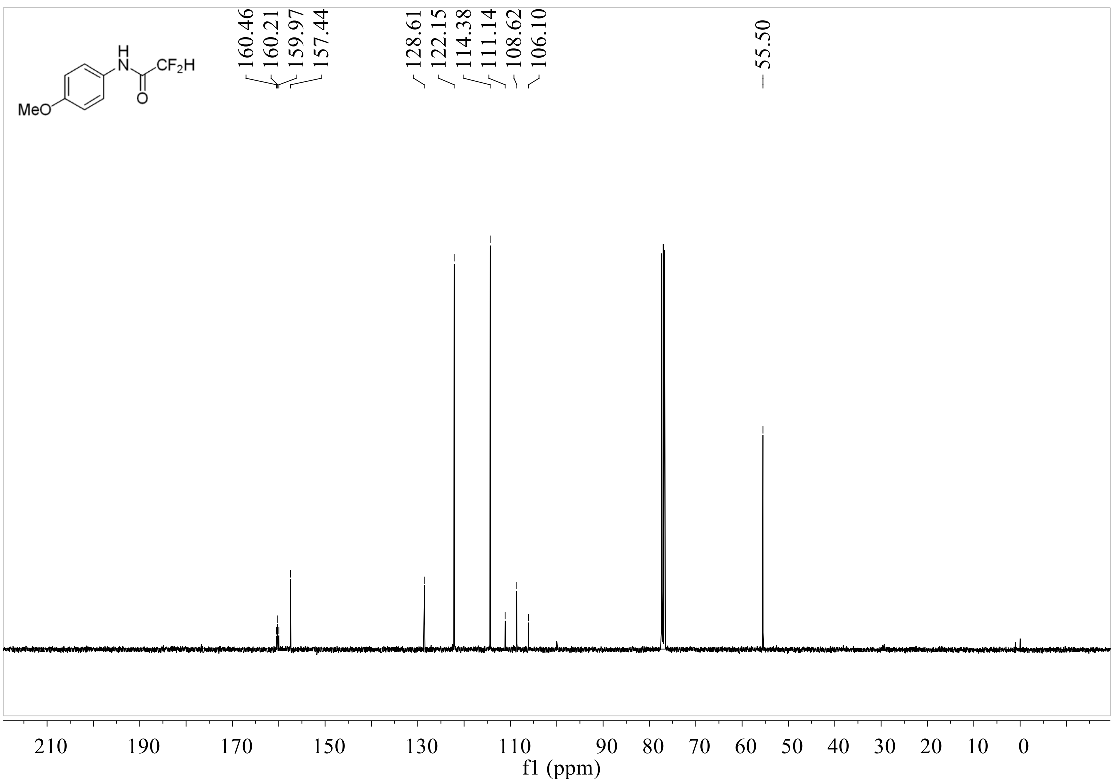

Supplementary Figure 113. ^{13}C NMR (101 MHz, $CDCl_3$) spectrum of **38**

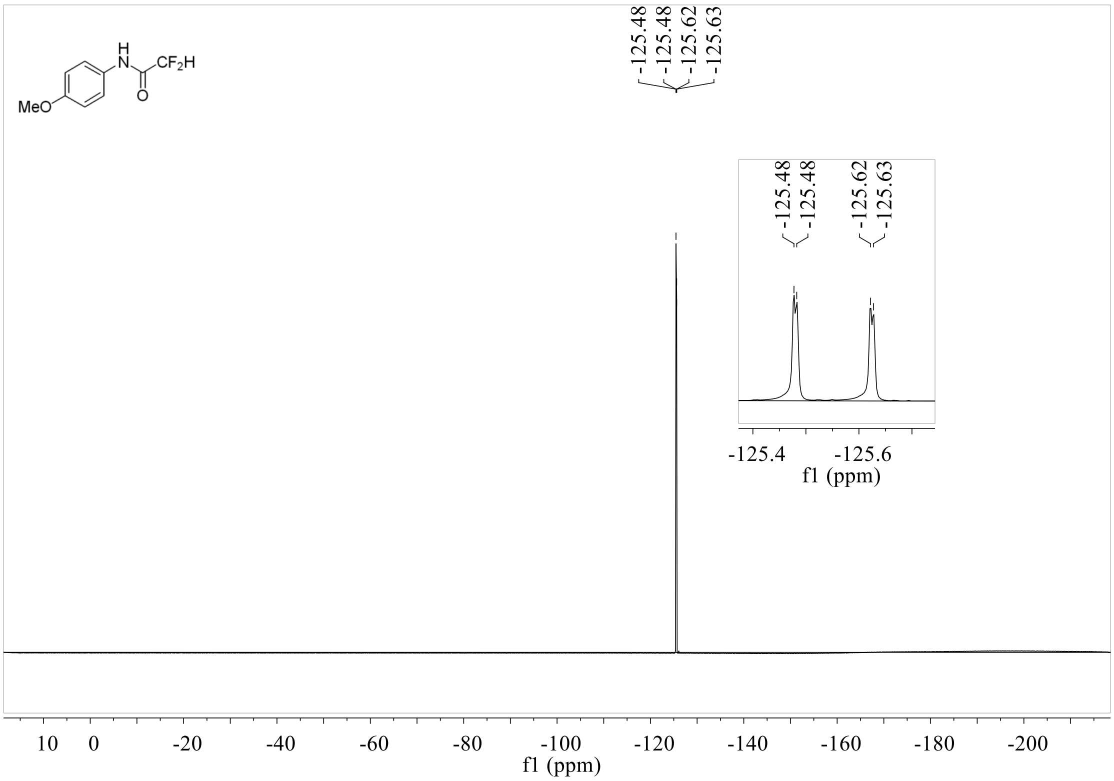

Supplementary Figure 114. ^{19}F NMR (376 MHz, $CDCl_3$) spectrum of **38**

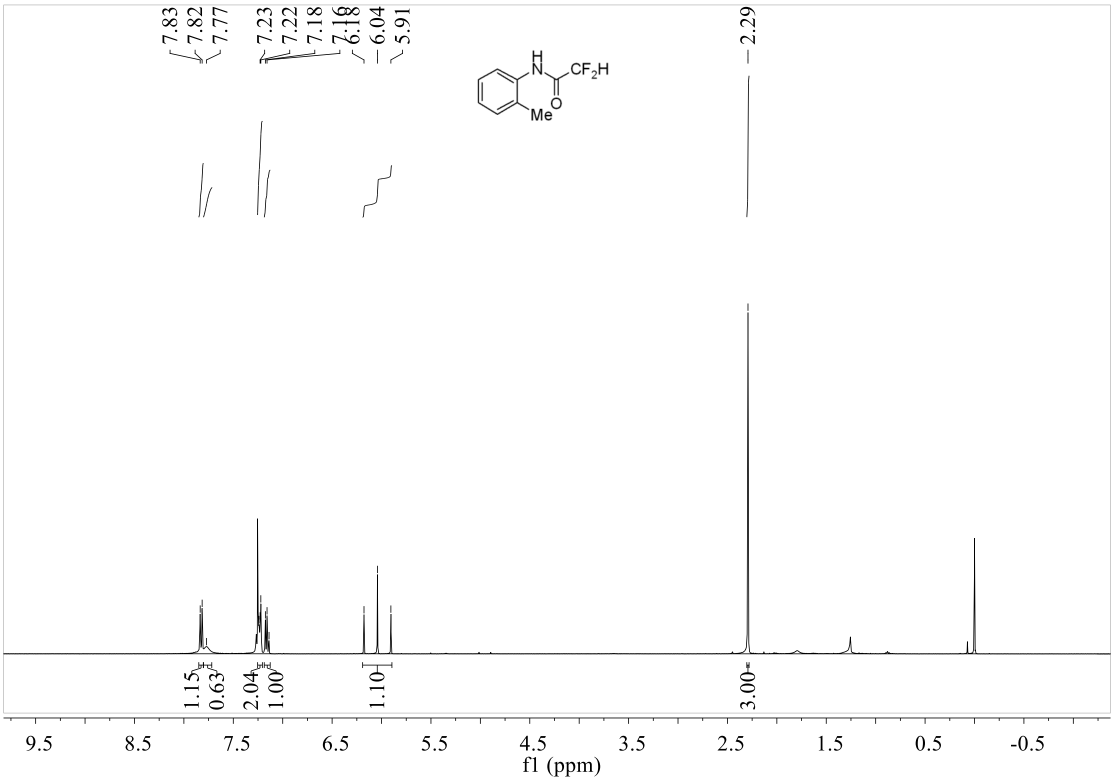

Supplementary Figure 115. ^{1}H NMR (400 MHz, $CDCl_3$) spectrum of **39**

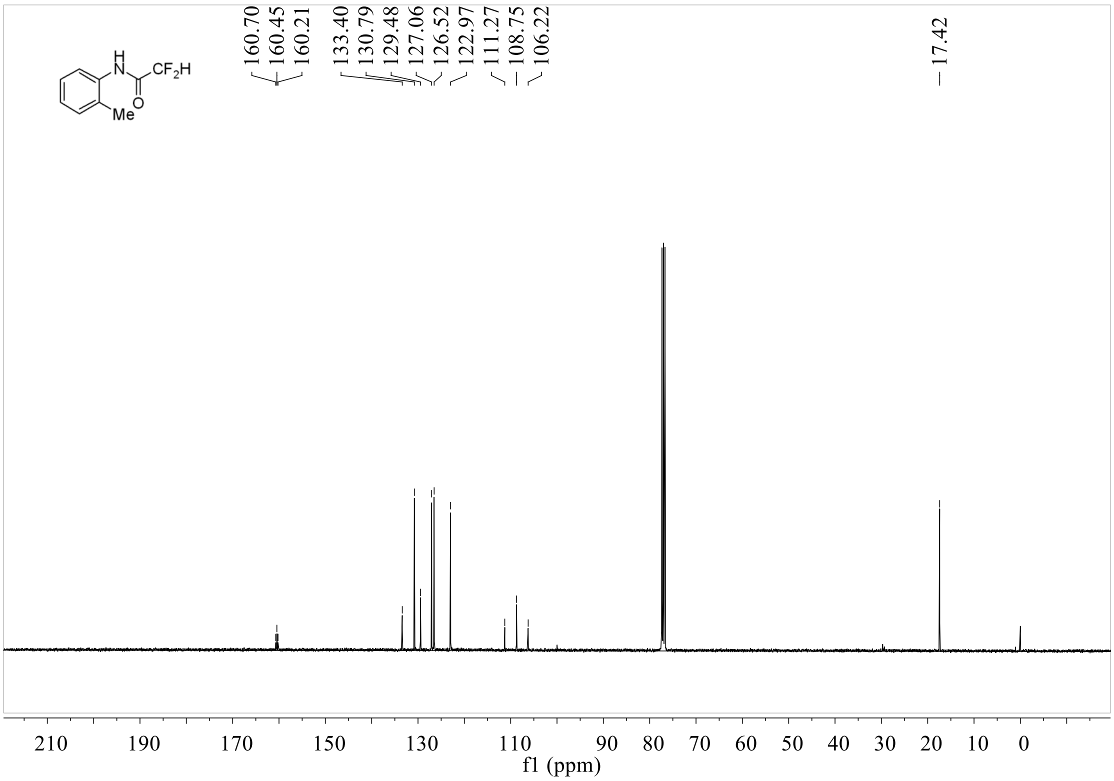

Supplementary Figure 116. ^{13}C NMR (101 MHz, $CDCl_3$) spectrum of **39**

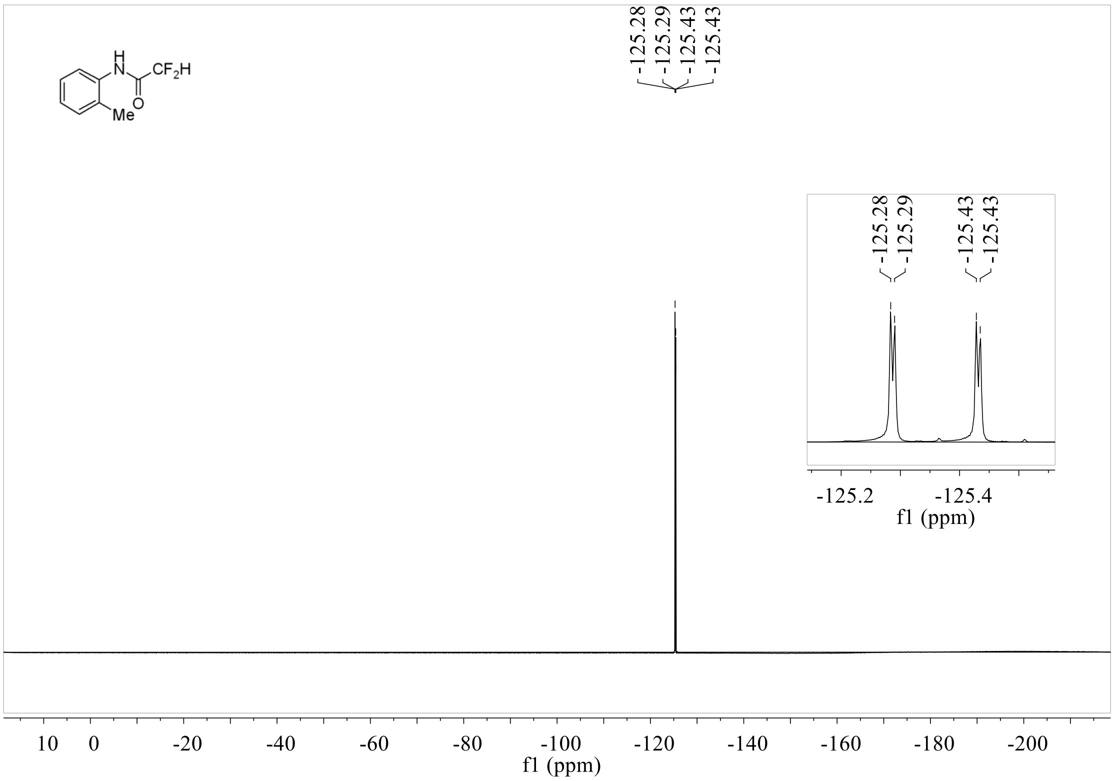

Supplementary Figure 117. ^{19}F NMR (376 MHz, $CDCl_3$) spectrum of **39**

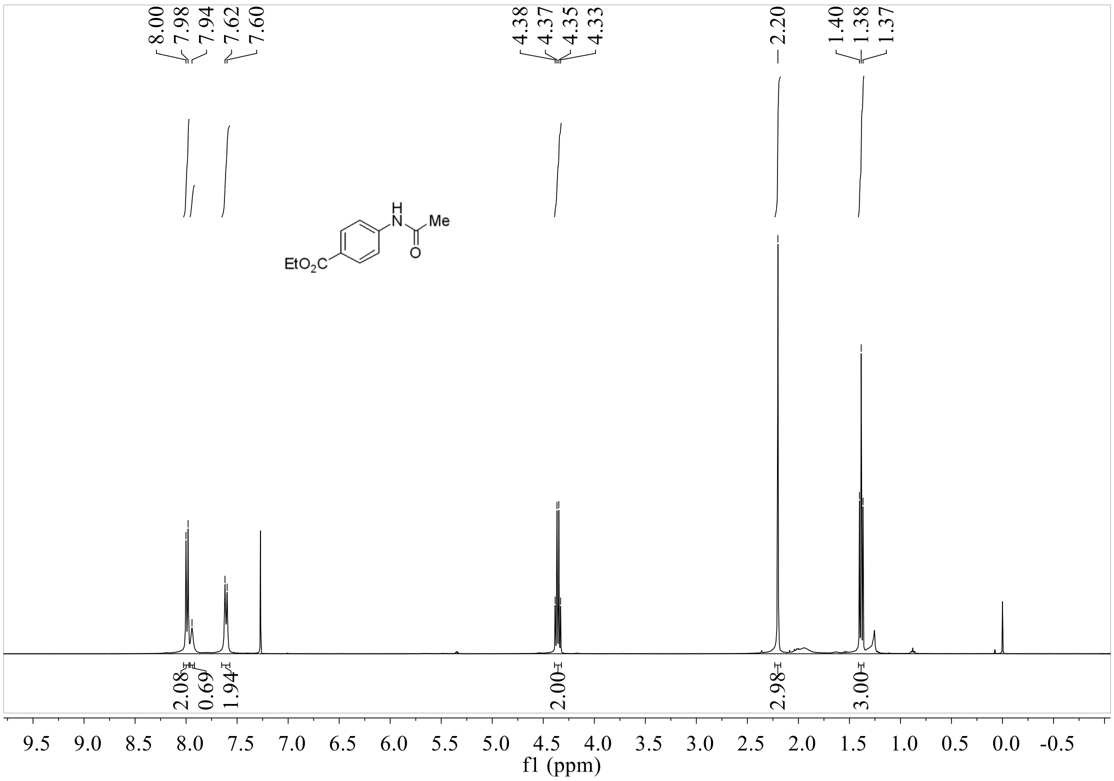

Supplementary Figure 118. ^{1}H NMR (400 MHz, $CDCl_3$) spectrum of **40**

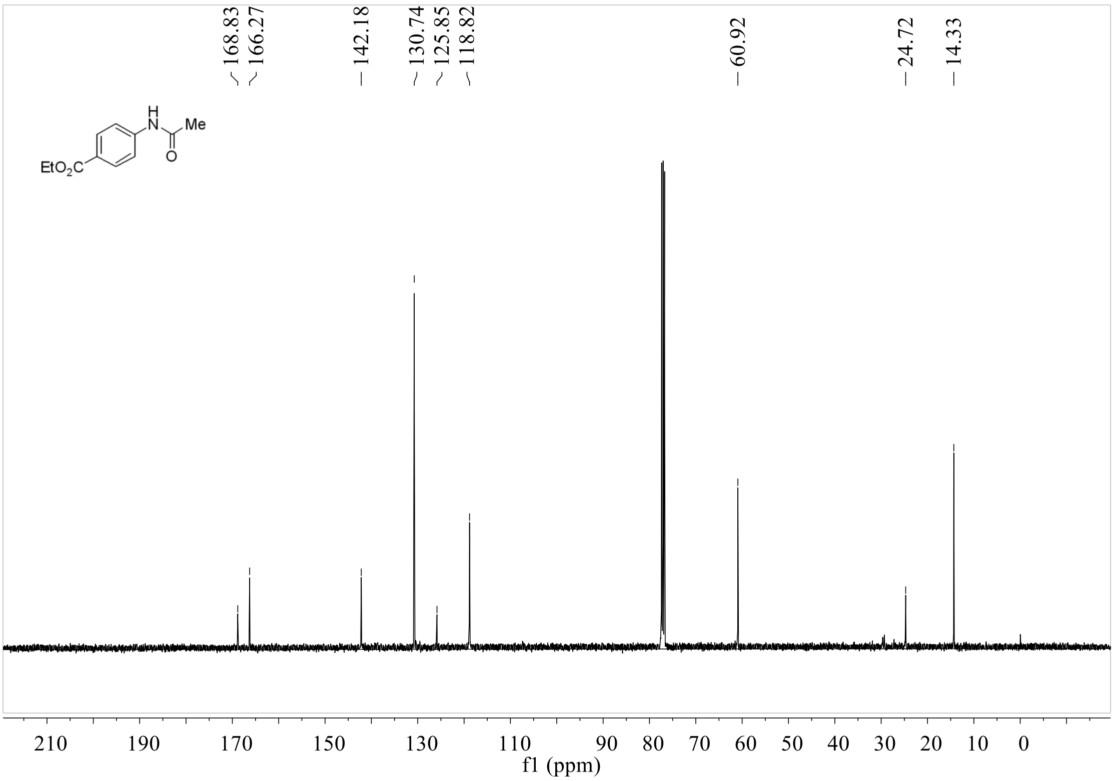

Supplementary Figure 119. ^{13}C NMR (101 MHz, $CDCl_3$) spectrum of **40**

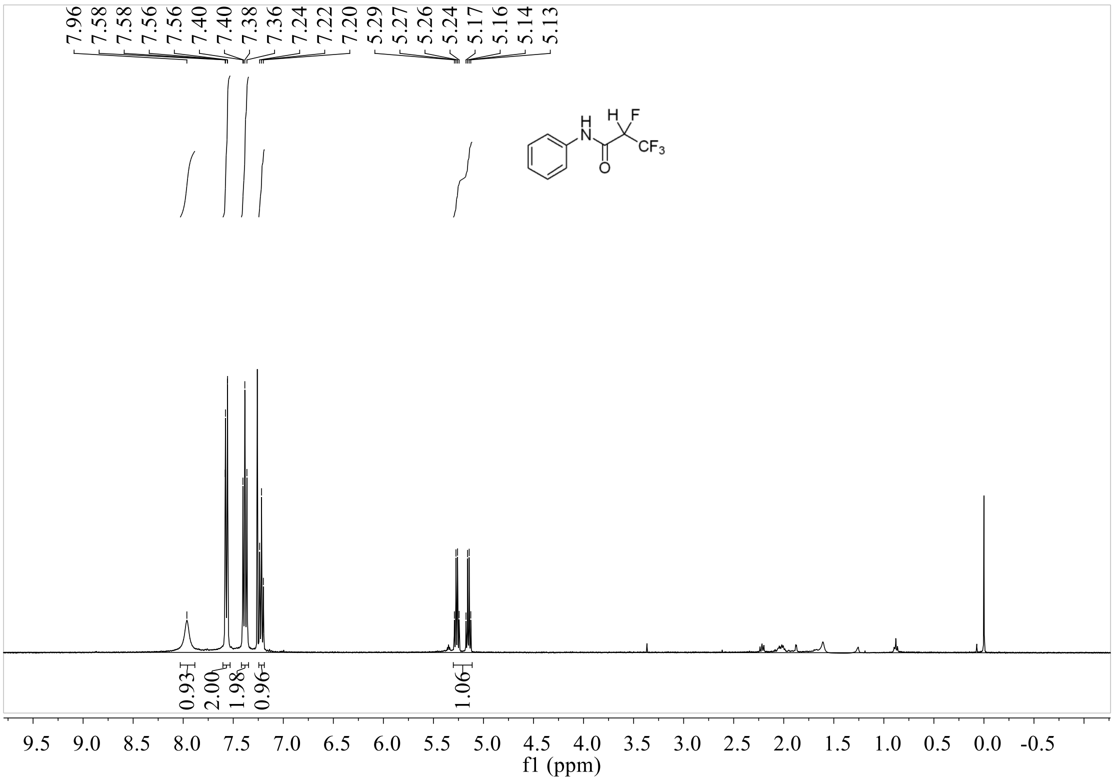

Supplementary Figure 120. ^{1}H NMR (400 MHz, $CDCl_3$) spectrum of **41**

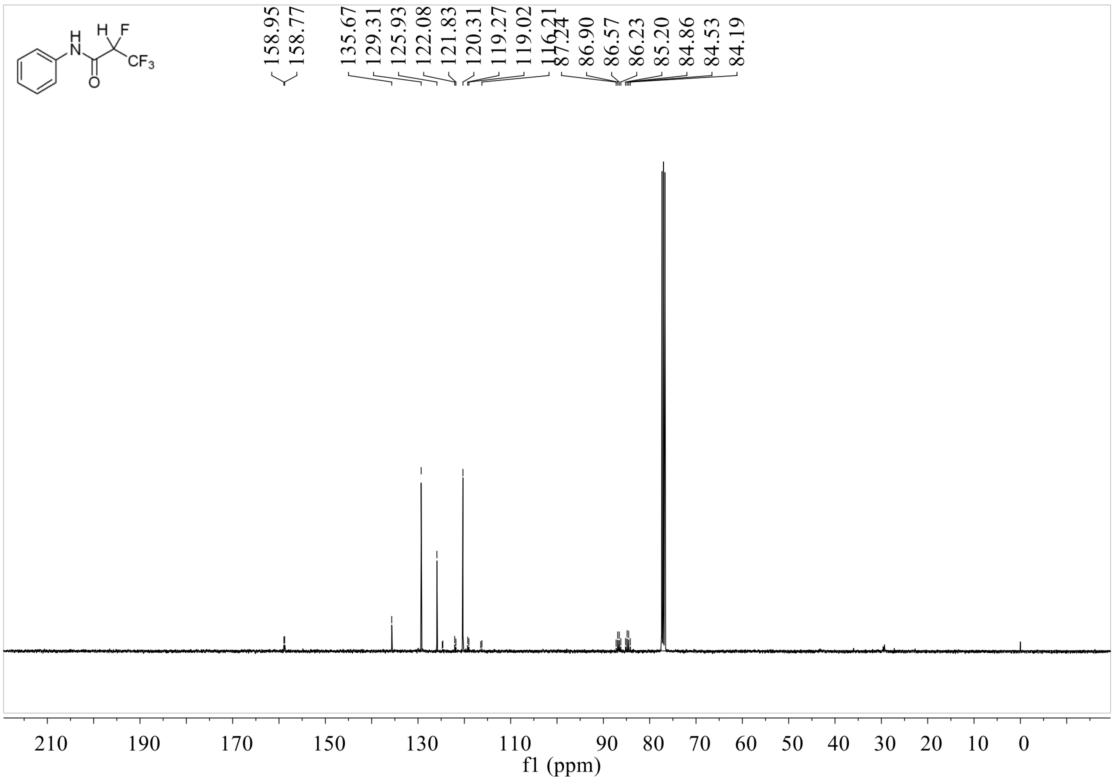

Supplementary Figure 121. ^{13}C NMR (101 MHz, $CDCl_3$) spectrum of **41**

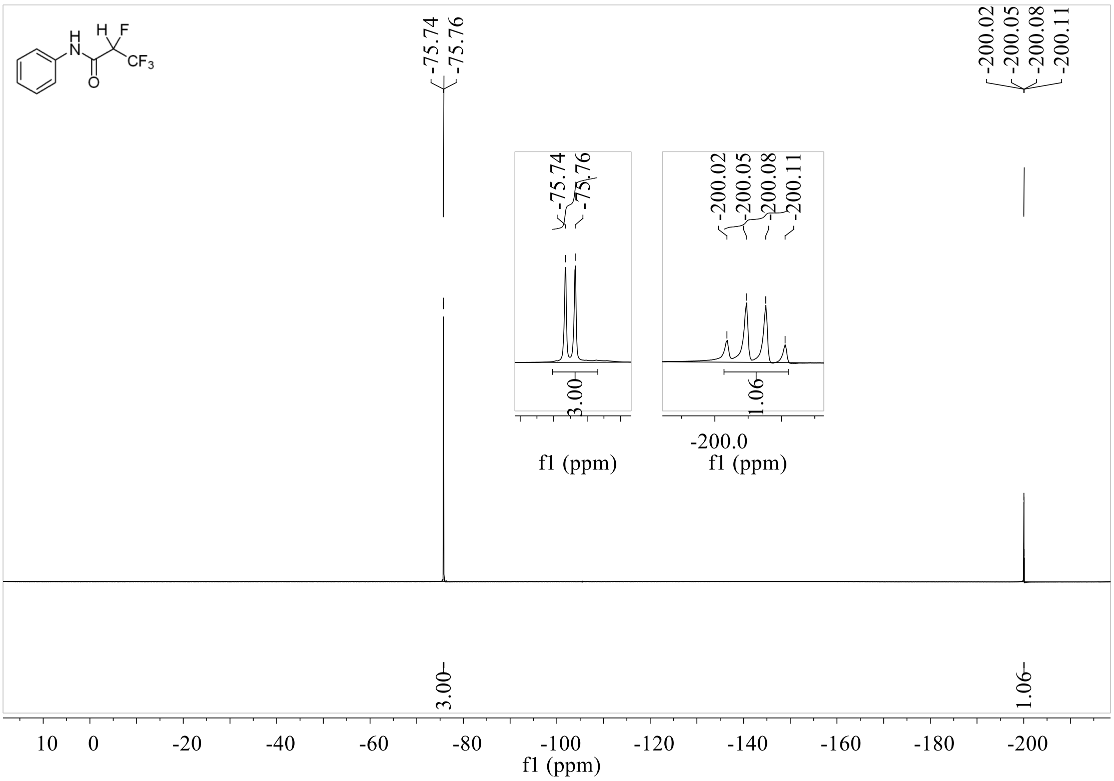

Supplementary Figure 122. ^{19}F NMR (376 MHz, $CDCl_3$) spectrum of **41**

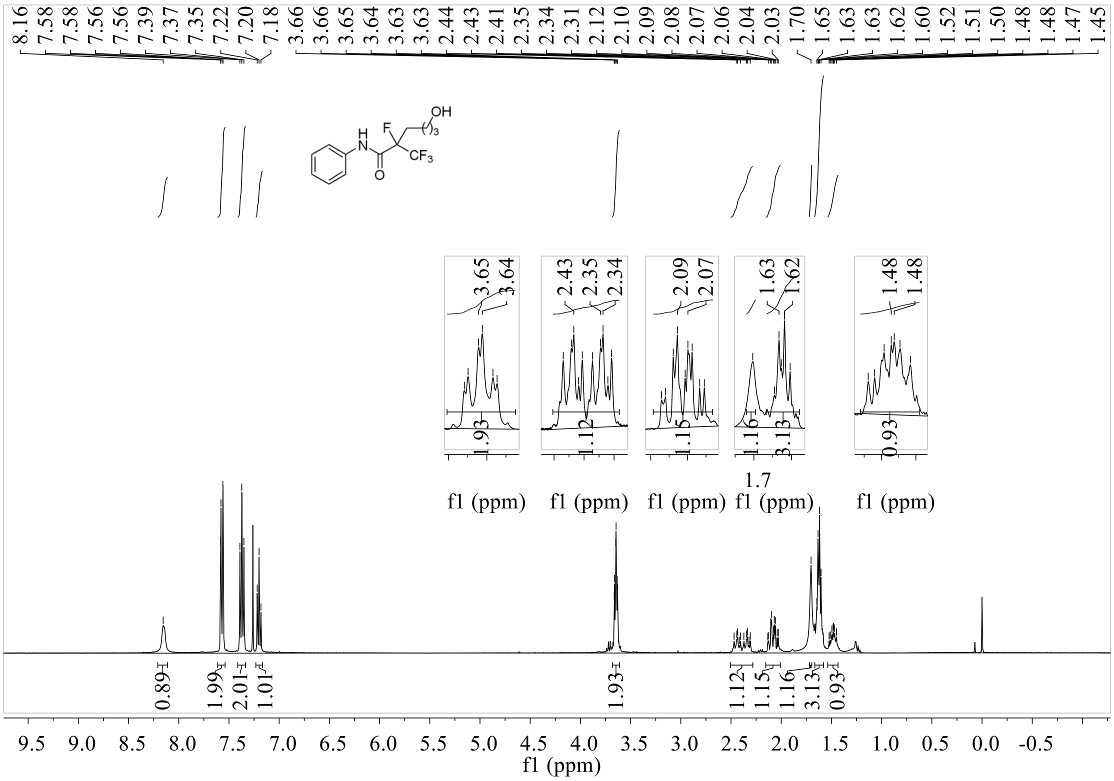

Supplementary Figure 123. ^{1}H NMR (400 MHz, $CDCl_3$) spectrum of **43**

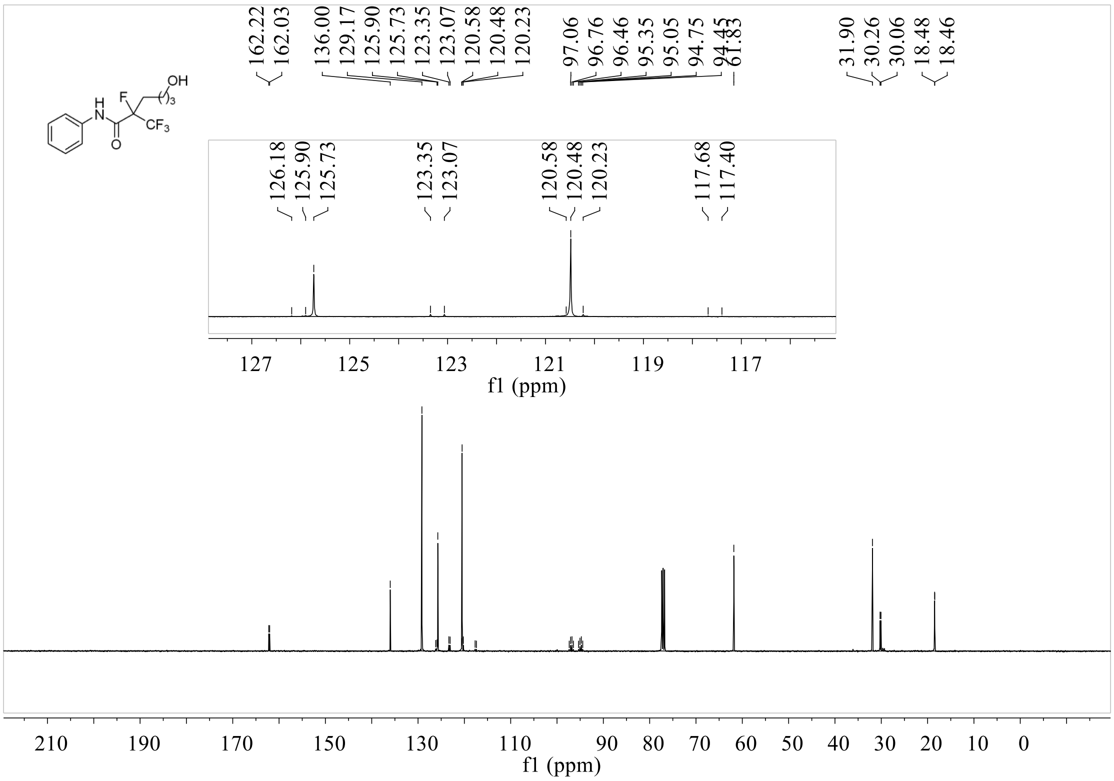

Supplementary Figure 124. ^{13}C NMR (101 MHz, $CDCl_3$) spectrum of **43**

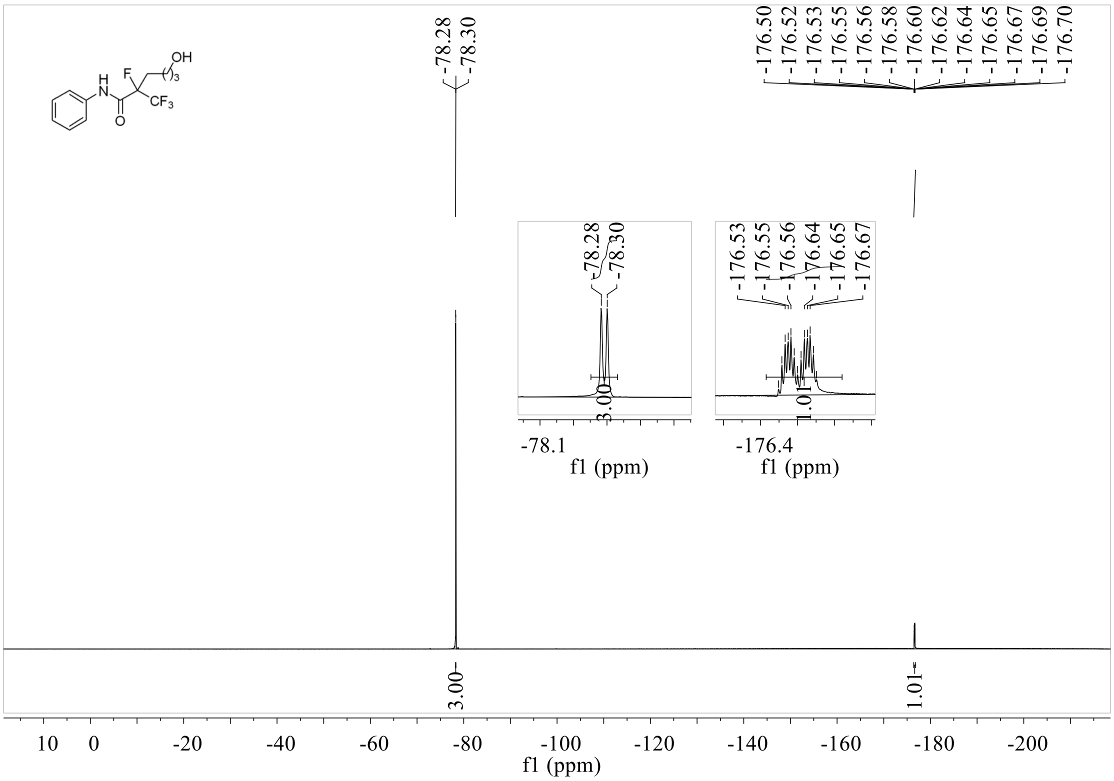

Supplementary Figure 125. ^{19}F NMR (376 MHz, $CDCl_3$) spectrum of **43**

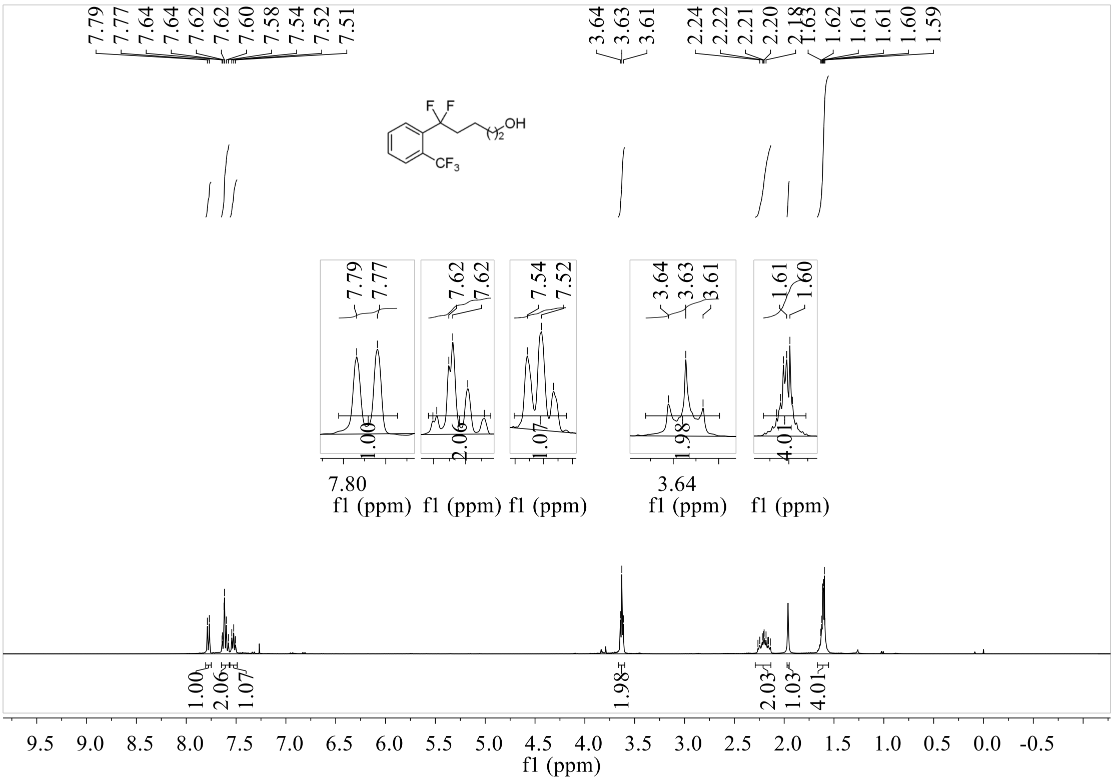

Supplementary Figure 126. ^{1}H NMR (400 MHz, $CDCl_3$) spectrum of **44**

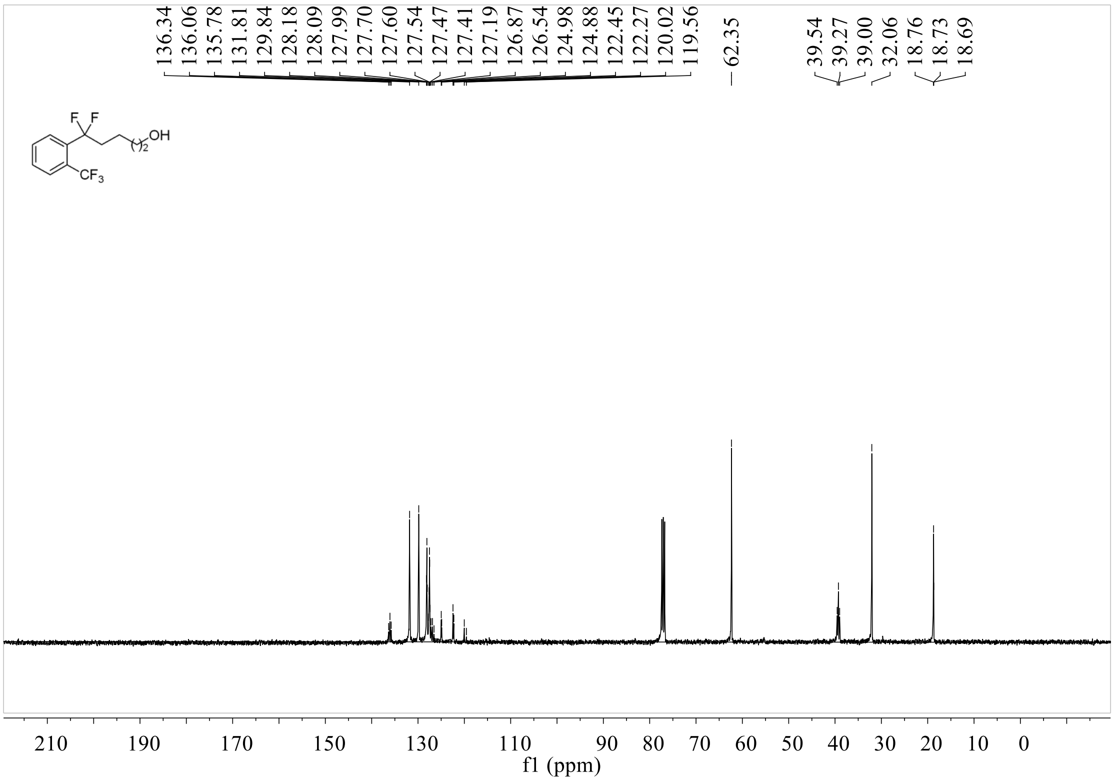

Supplementary Figure 127. ^{13}C NMR (101 MHz, $CDCl_3$) spectrum of **44**

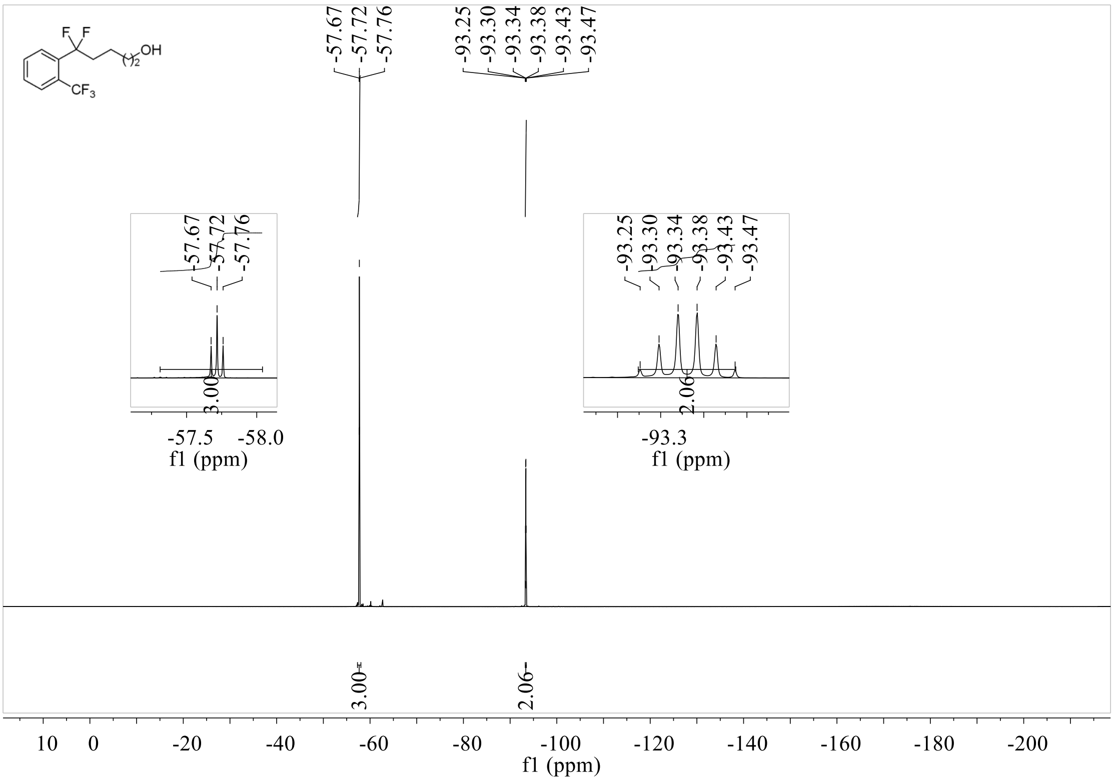

Supplementary Figure 128. ^{19}F NMR (376 MHz, $CDCl_3$) spectrum of **44**

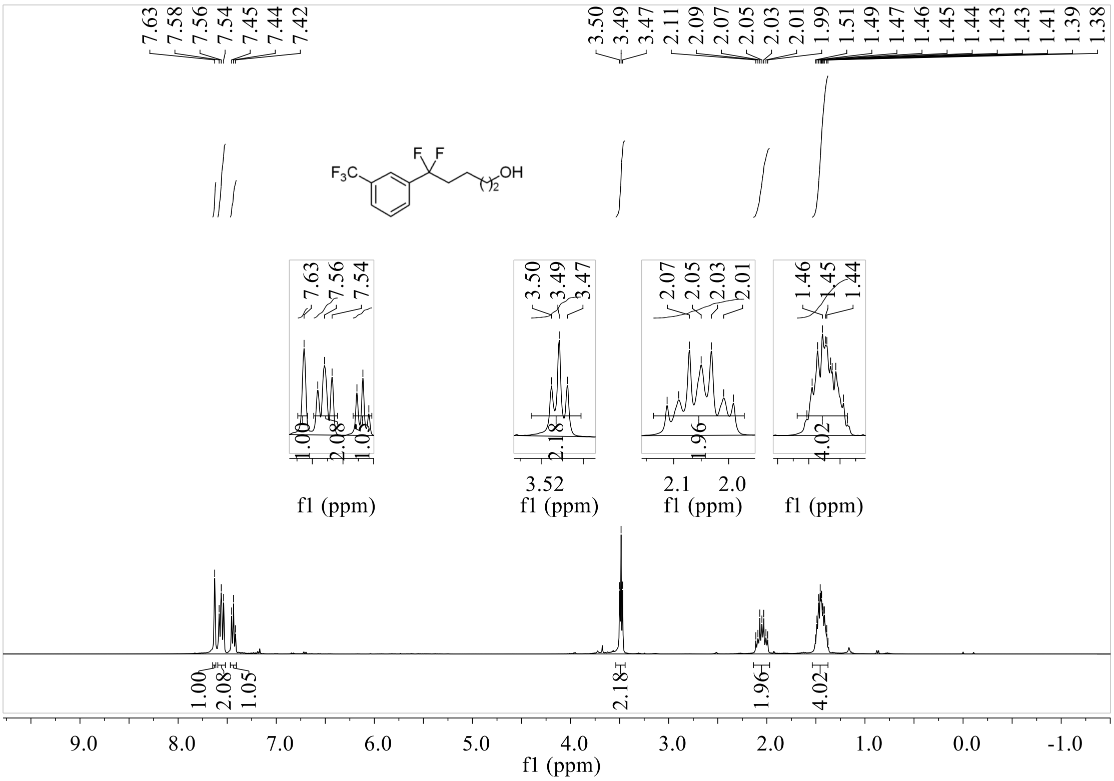

Supplementary Figure 129. ^{1}H NMR (400 MHz, $CDCl_3$) spectrum of **45**

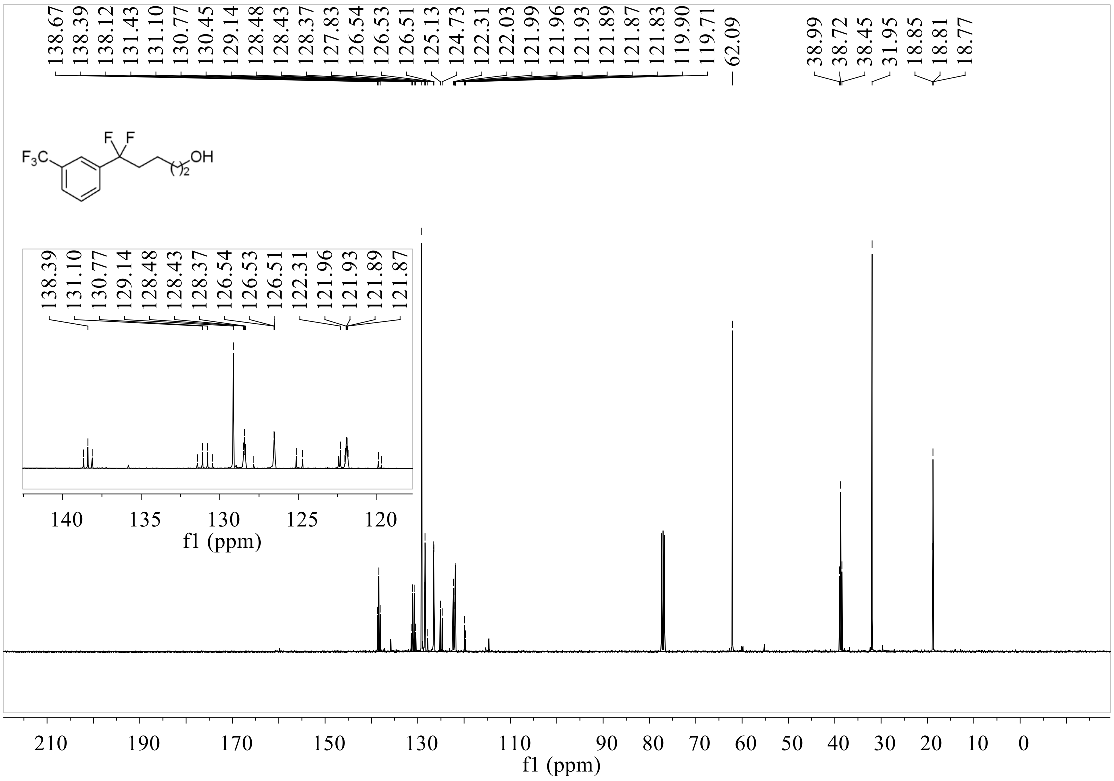

Supplementary Figure 130. ^{13}C NMR (101 MHz, $CDCl_3$) spectrum of **45**

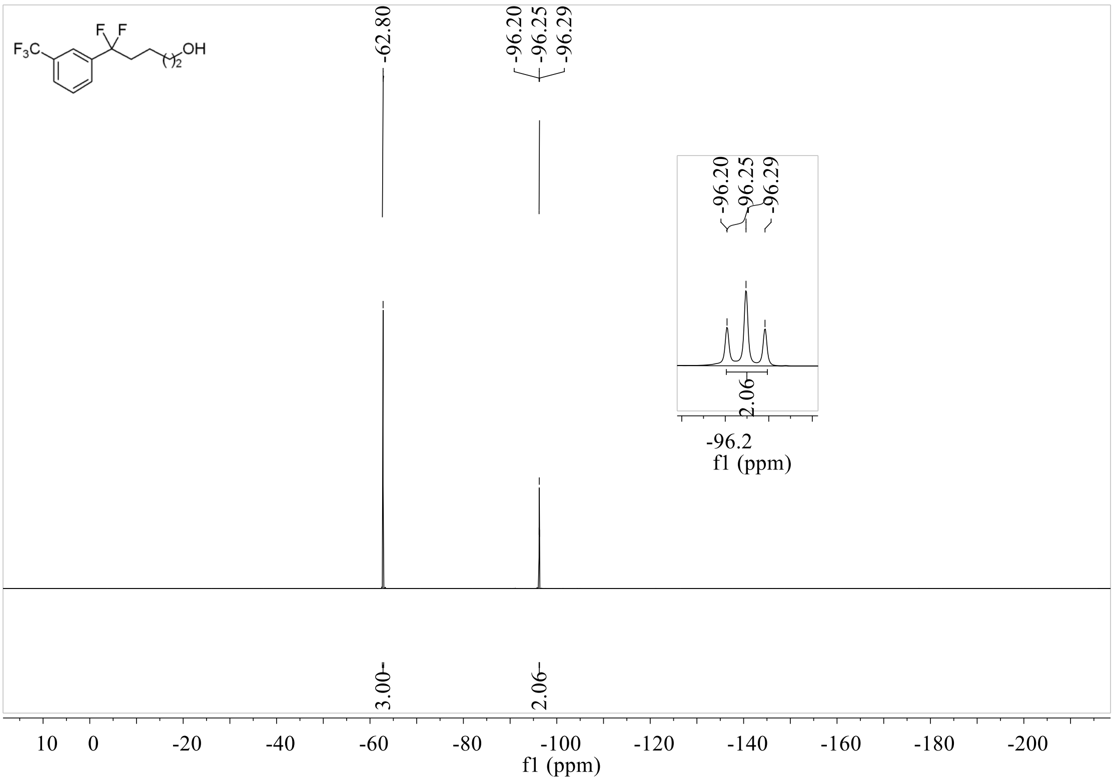

Supplementary Figure 131. ^{19}F NMR (376 MHz, $CDCl_3$) spectrum of **45**

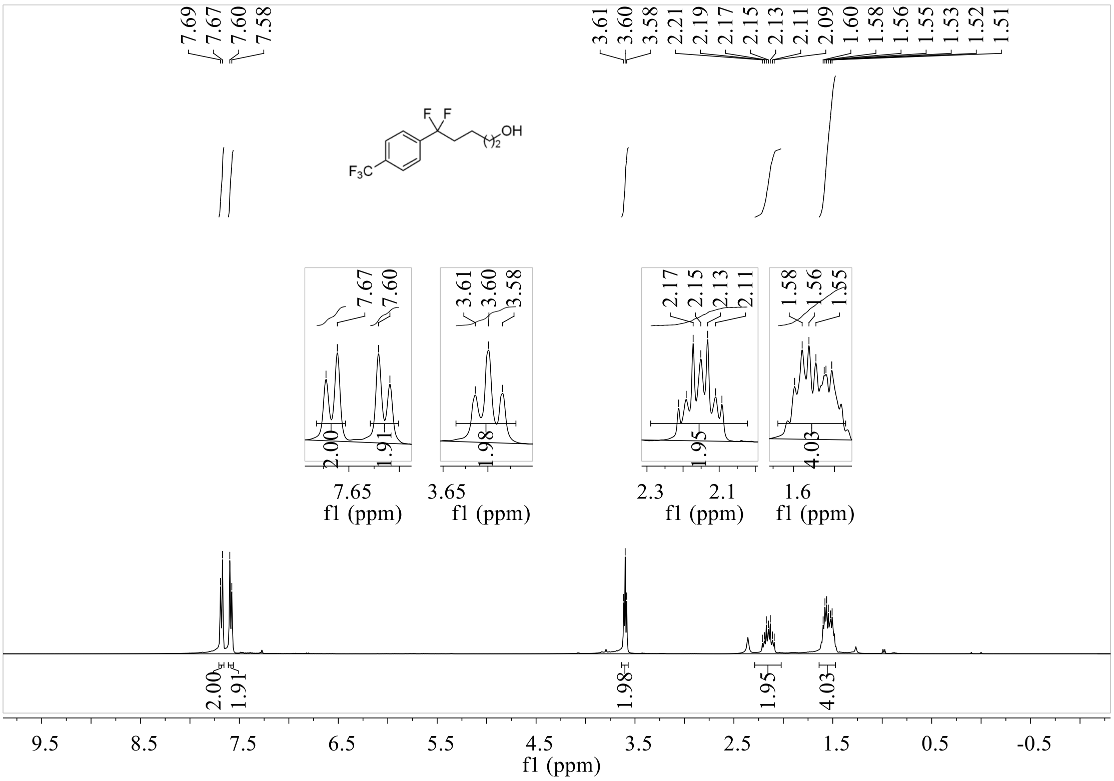

Supplementary Figure 132. ^{1}H NMR (400 MHz, $CDCl_3$) spectrum of **46**

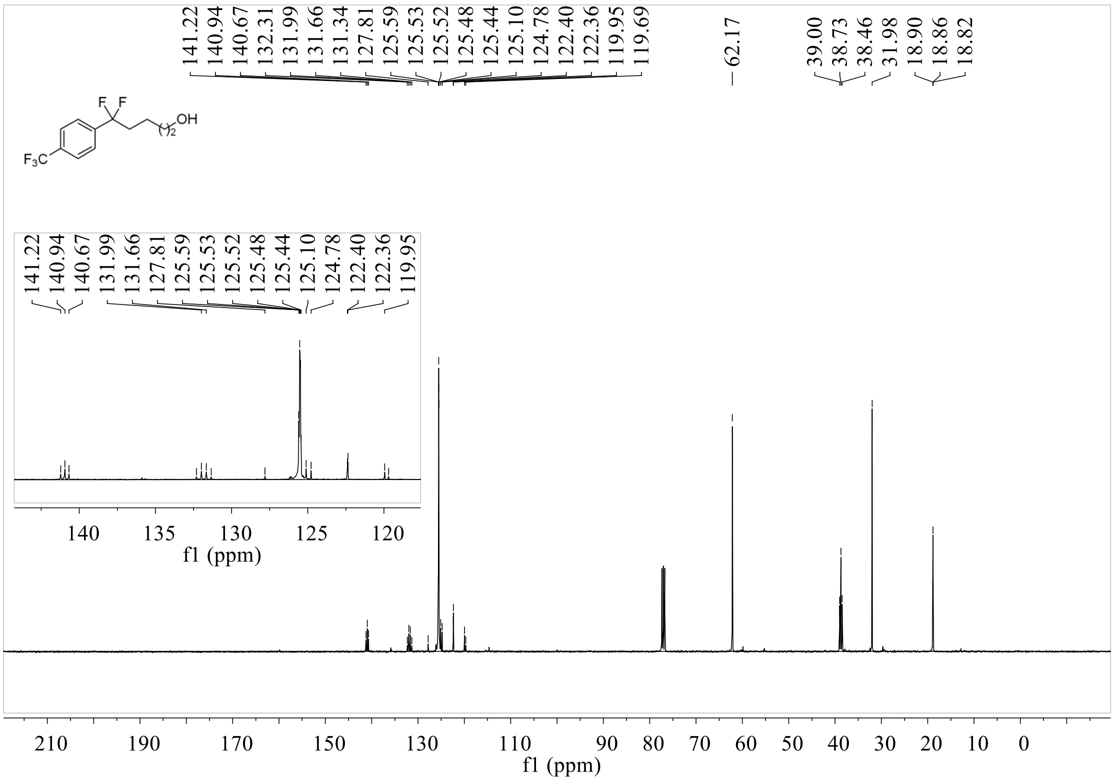

Supplementary Figure 133. ^{13}C NMR (101 MHz, $CDCl_3$) spectrum of **46**

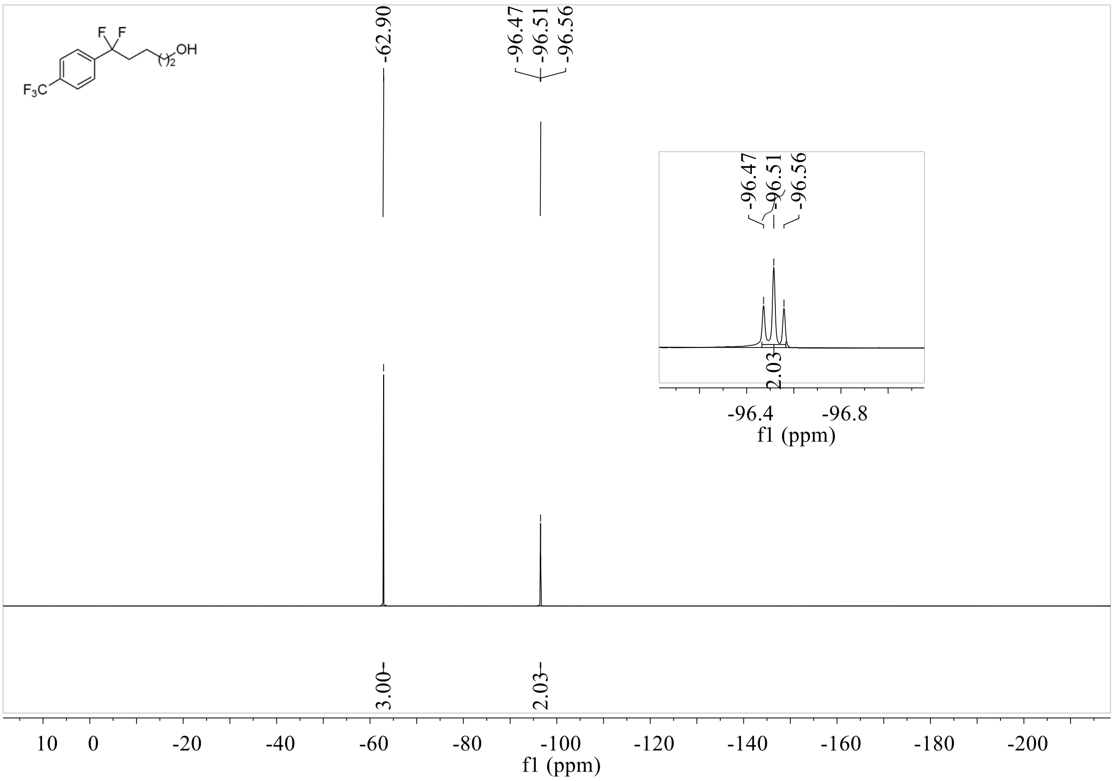

Supplementary Figure 134. ^{19}F NMR (376 MHz, $CDCl_3$) spectrum of **46**

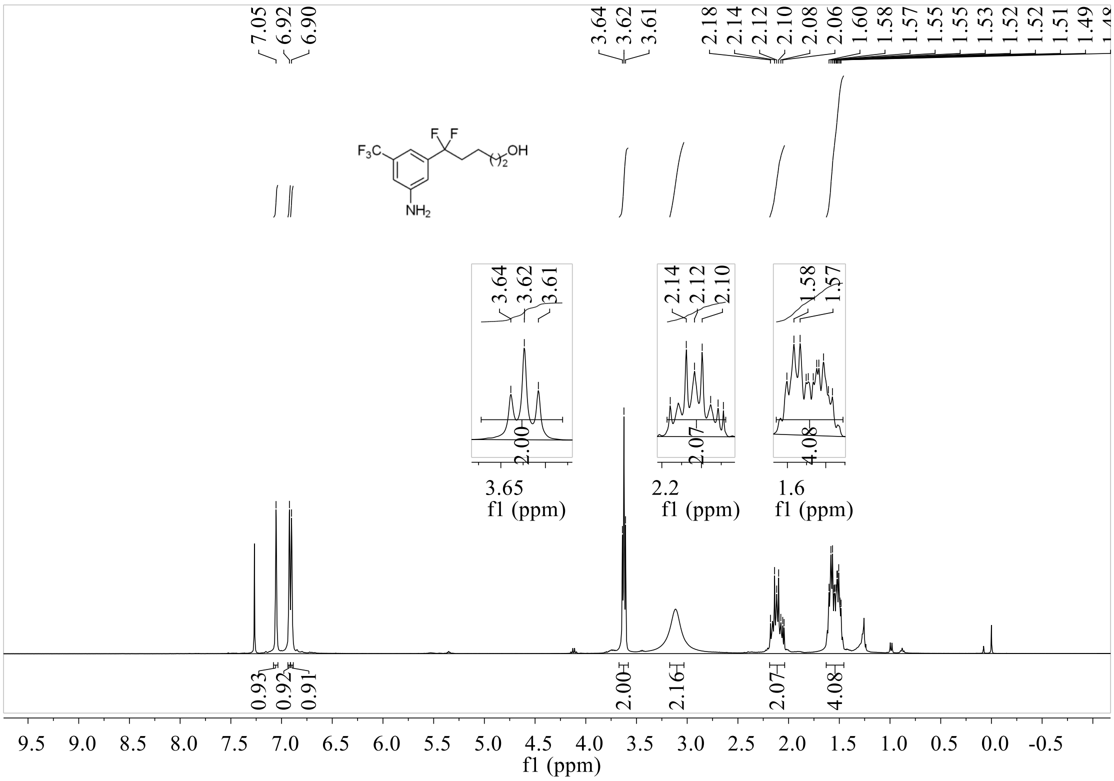

Supplementary Figure 135. ^{1}H NMR (400 MHz, $CDCl_3$) spectrum of **47**

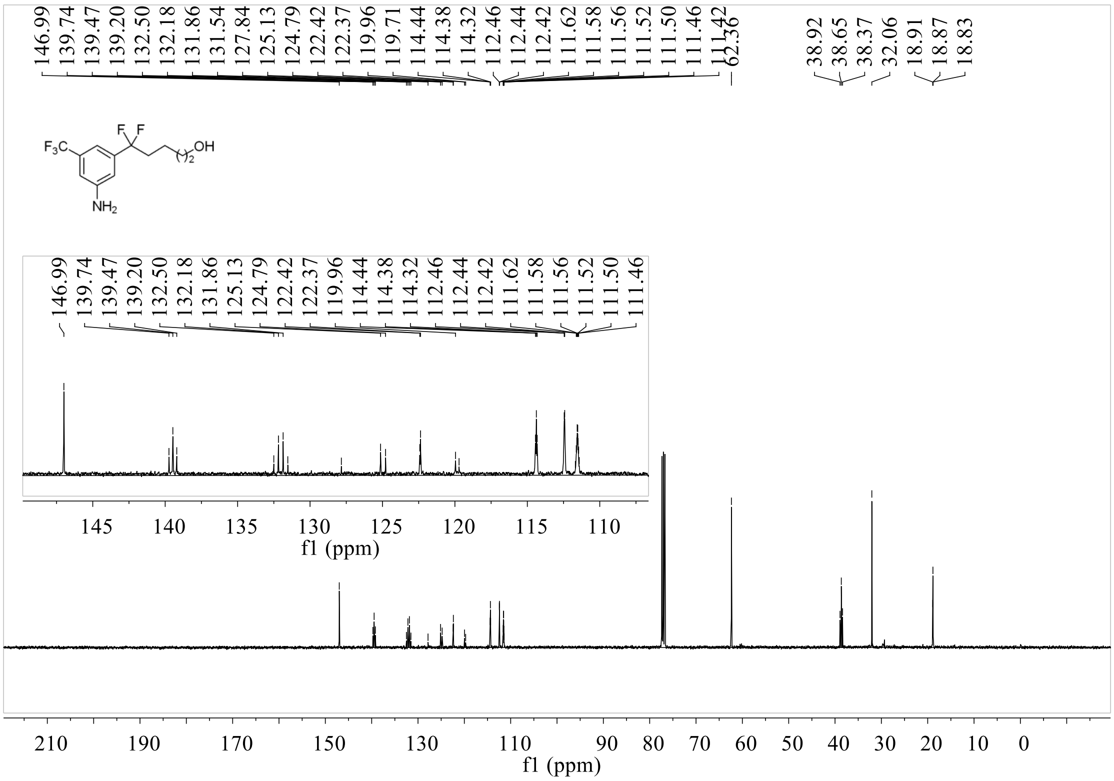

Supplementary Figure 136. ^{13}C NMR (101 MHz, $CDCl_3$) spectrum of **47**

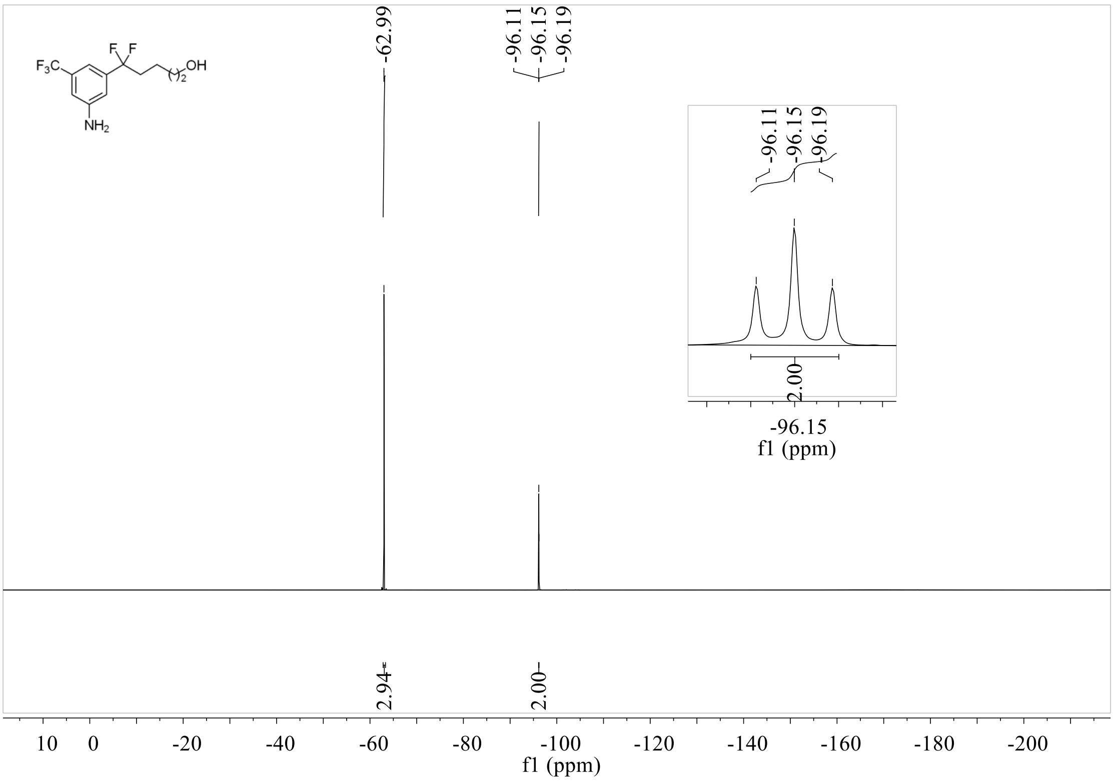

Supplementary Figure 137. ^{19}F NMR (376 MHz, $CDCl_3$) spectrum of **47**

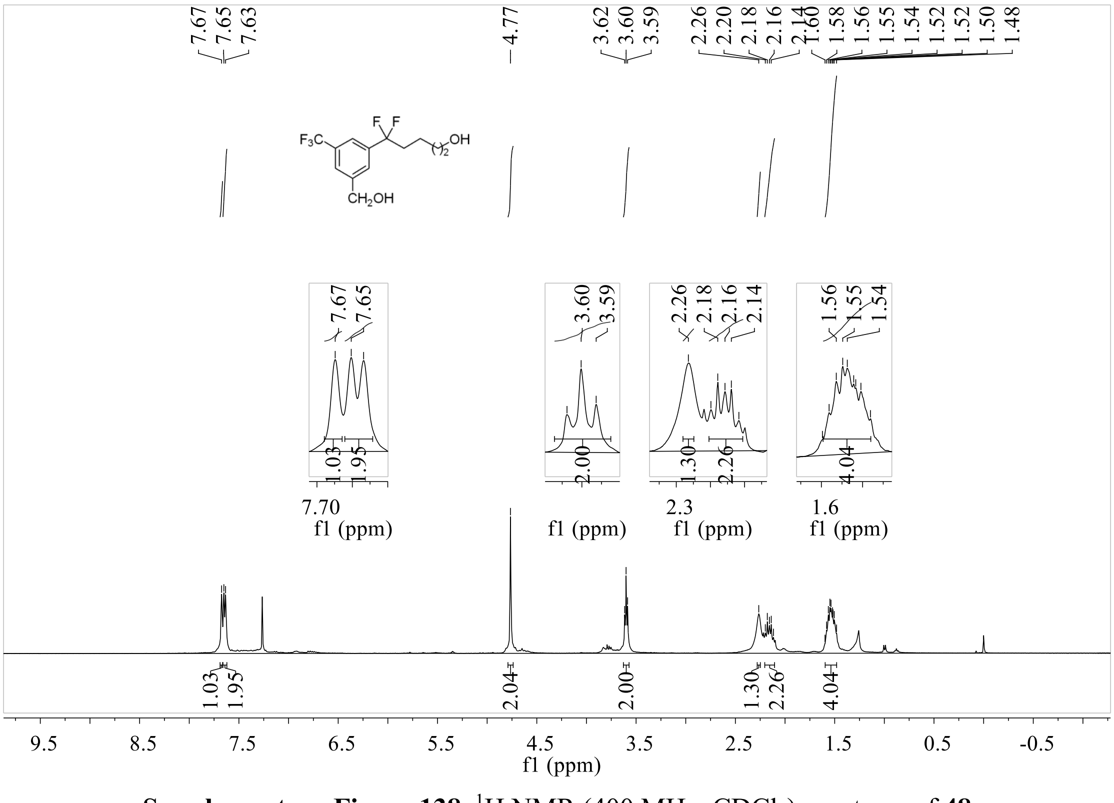

Supplementary Figure 138. ^{1}H NMR (400 MHz, $CDCl_3$) spectrum of **48**

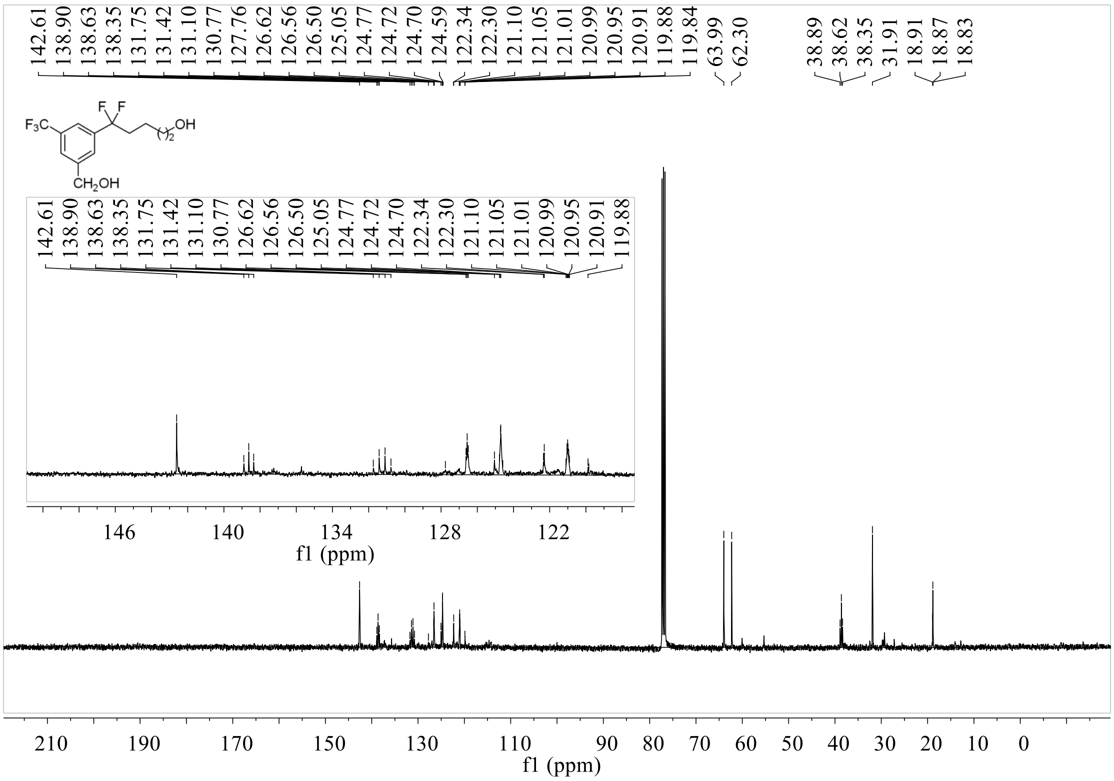

Supplementary Figure 139. ^{13}C NMR (101 MHz, $CDCl_3$) spectrum of **48**

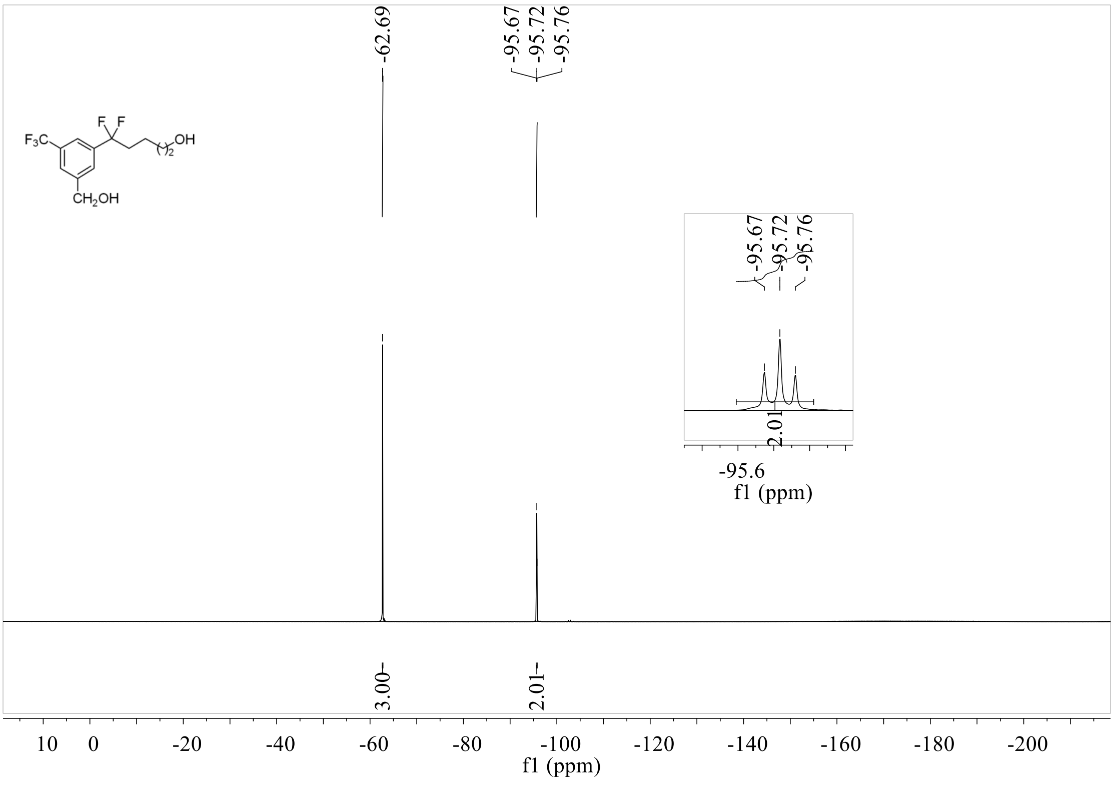

Supplementary Figure 140. ^{19}F NMR (376 MHz, $CDCl_3$) spectrum of **48**

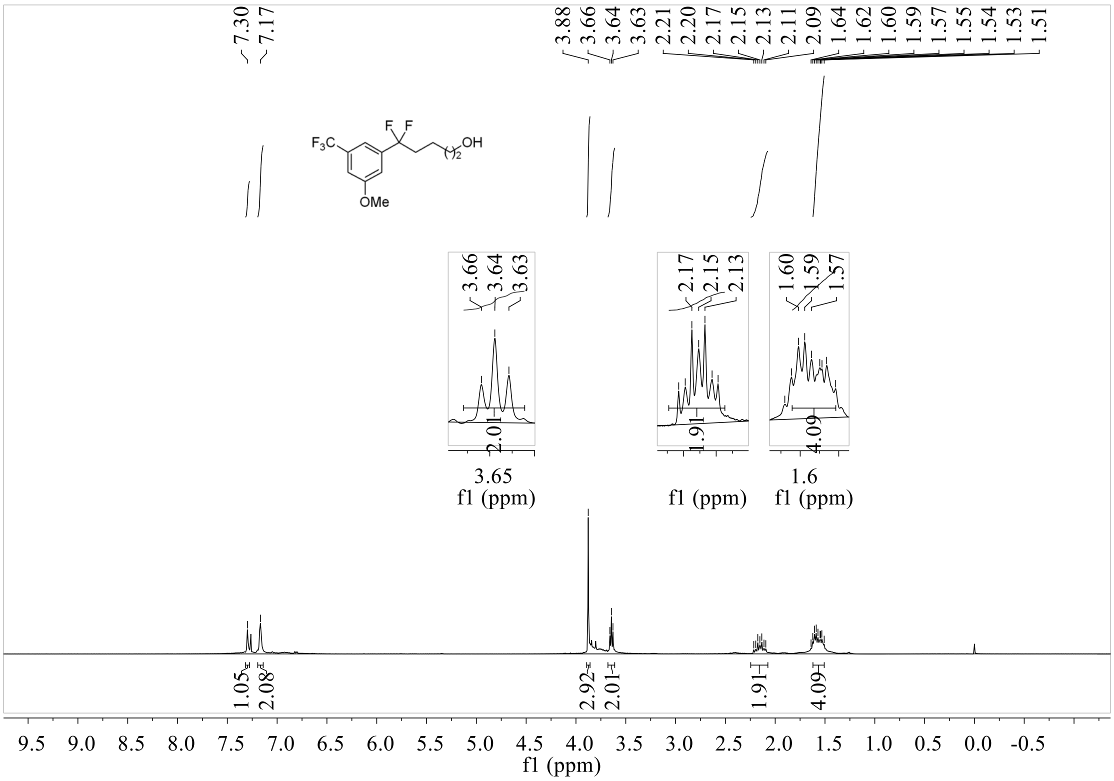

Supplementary Figure 141. ^{1}H NMR (400 MHz, $CDCl_3$) spectrum of **49**

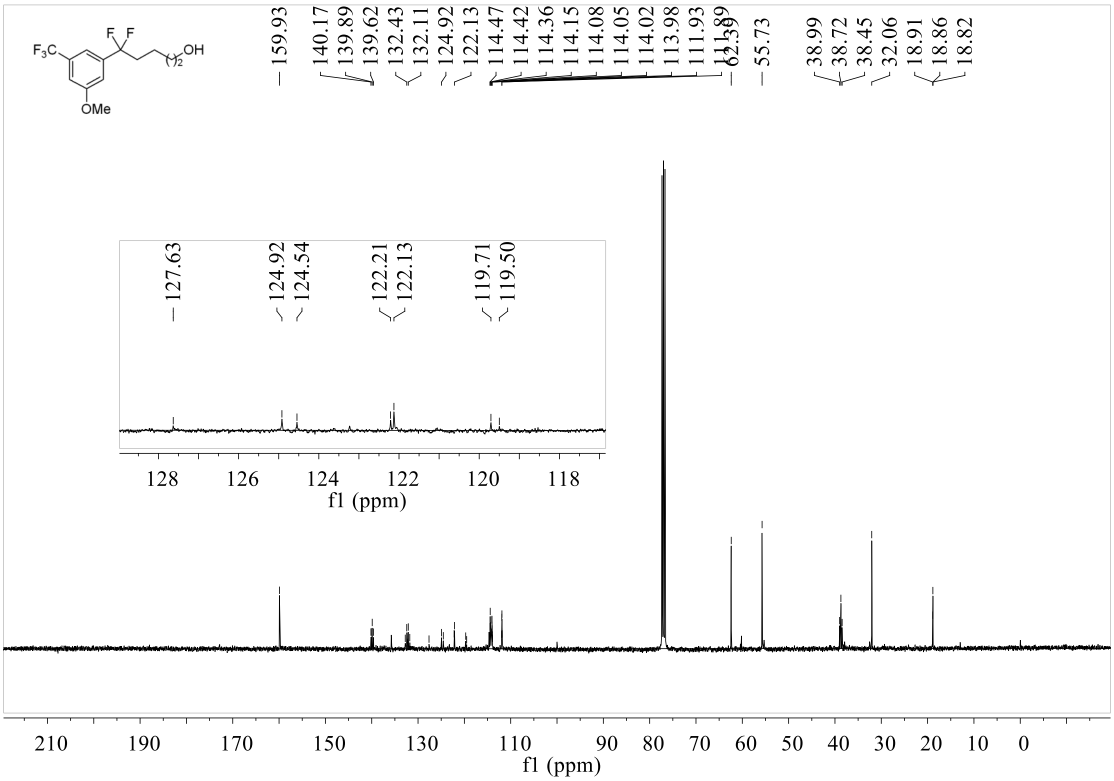

Supplementary Figure 142. ^{13}C NMR (101 MHz, $CDCl_3$) spectrum of **49**

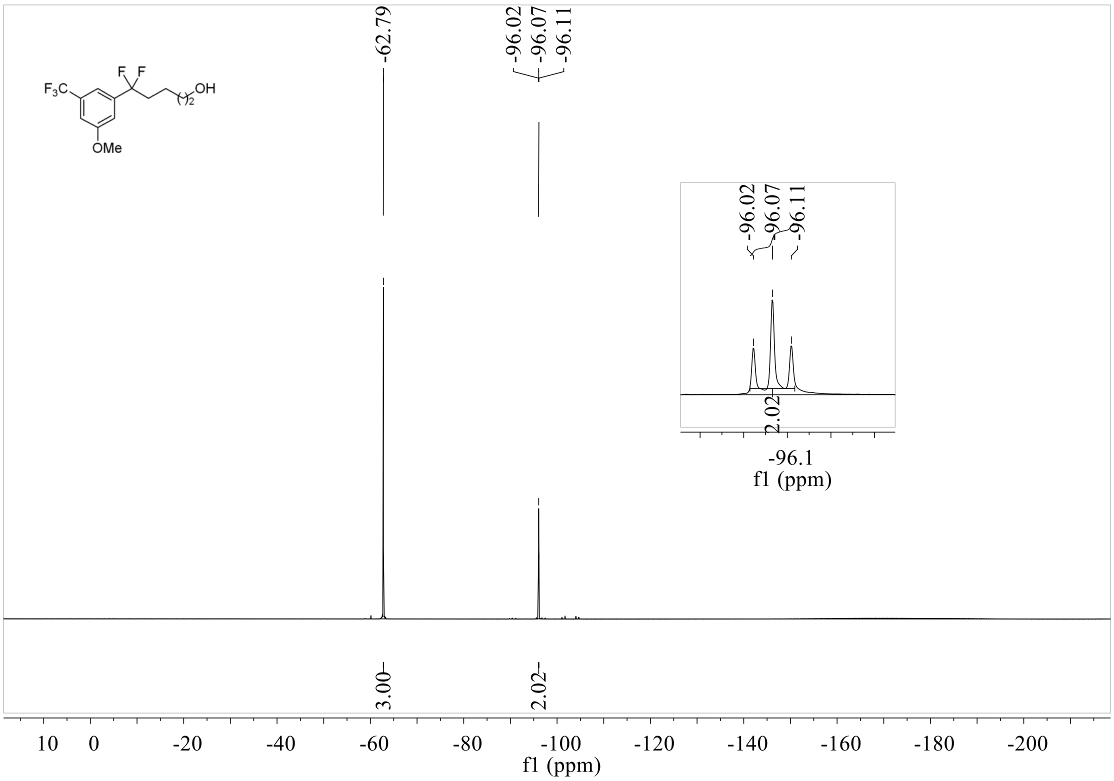

Supplementary Figure 143. ^{19}F NMR (376 MHz, $CDCl_3$) spectrum of **49**

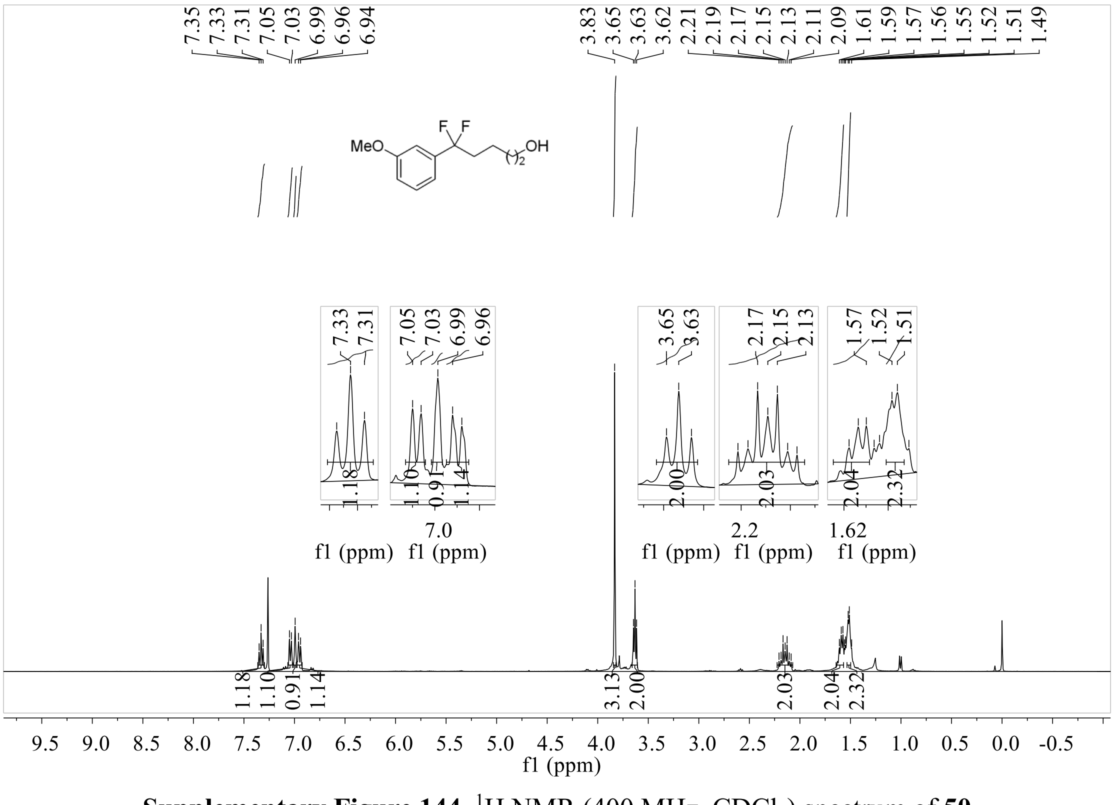

Supplementary Figure 144. ^{1}H NMR (400 MHz, $CDCl_3$) spectrum of **50**

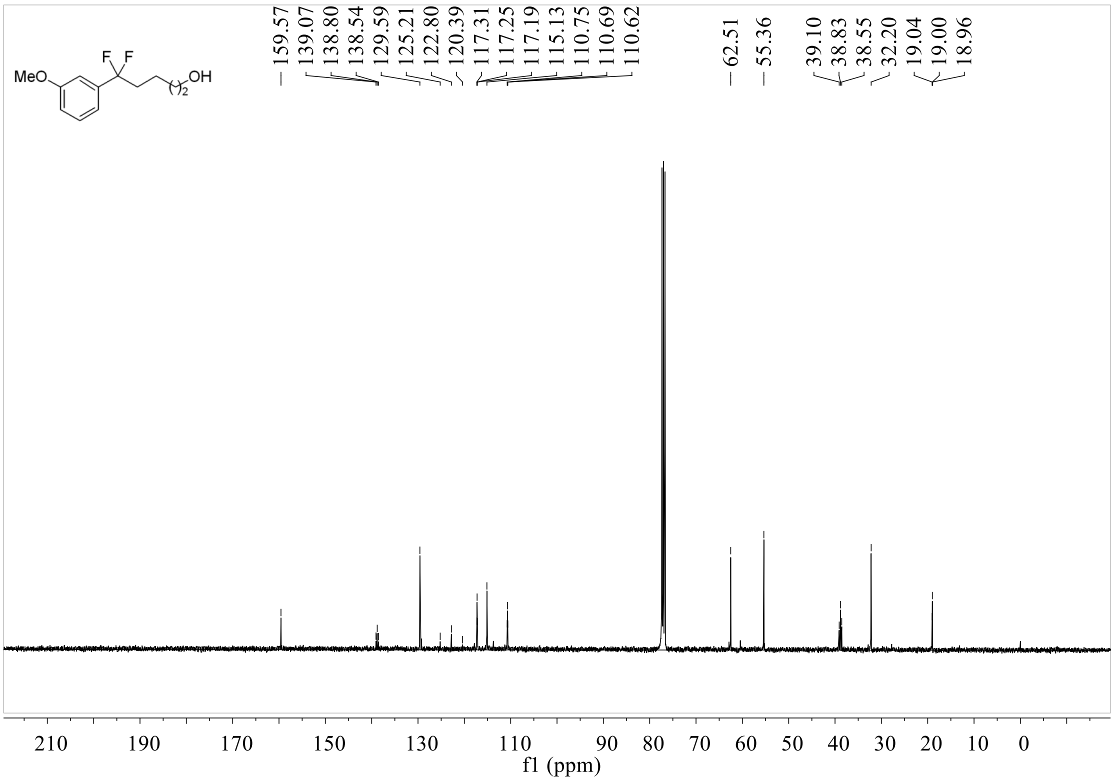

Supplementary Figure 145. ^{13}C NMR (101 MHz, $CDCl_3$) spectrum of **50**

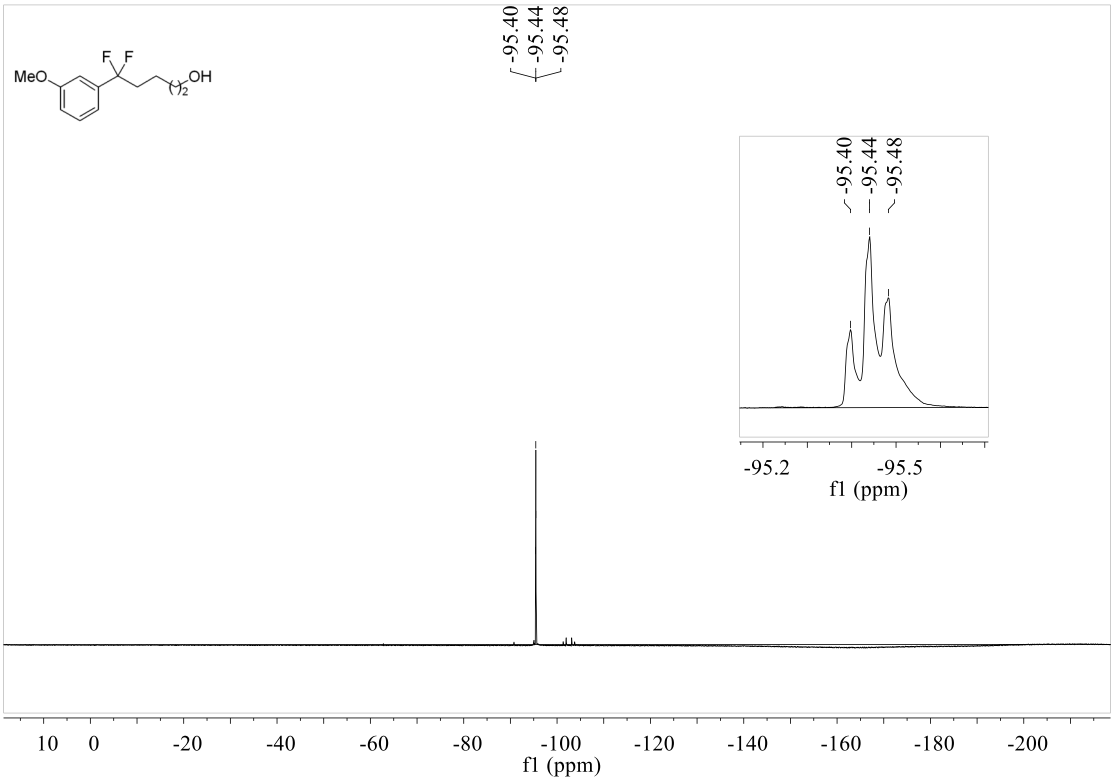

Supplementary Figure 146. ^{19}F NMR (376 MHz, $CDCl_3$) spectrum of **50**

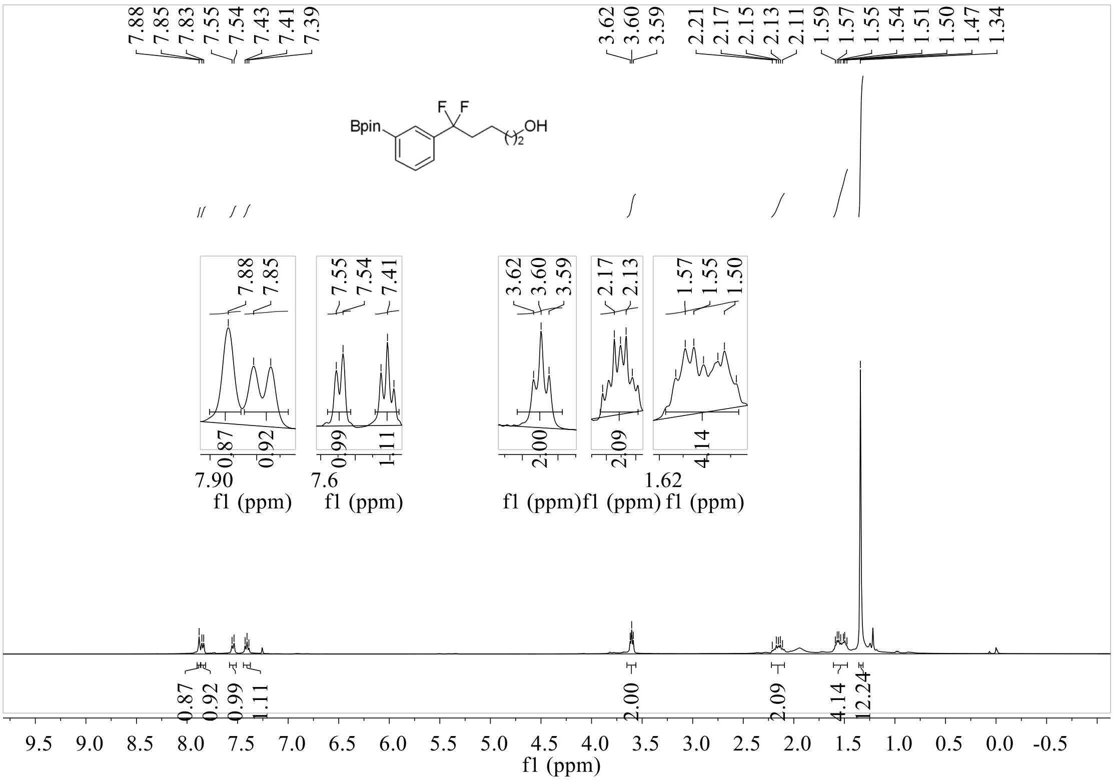

Supplementary Figure 147. ^{1}H NMR (400 MHz, $CDCl_3$) spectrum of **51**

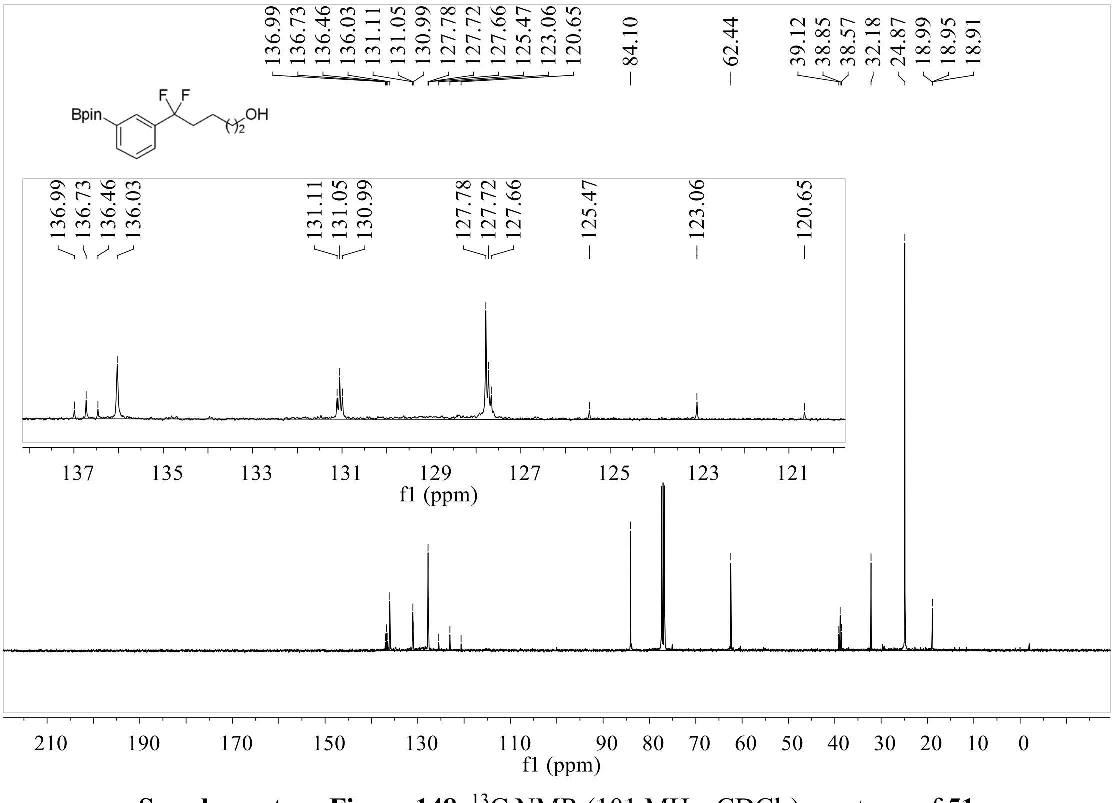

Supplementary Figure 148. ^{13}C NMR (101 MHz, $CDCl_3$) spectrum of **51**

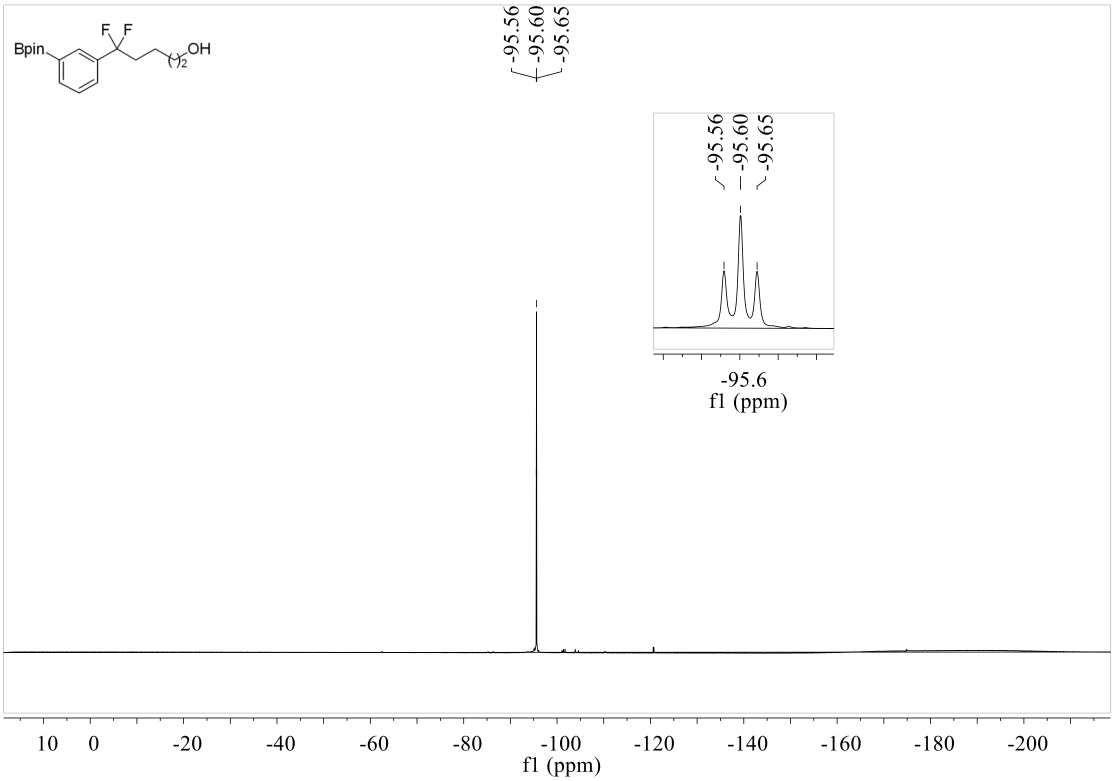

Supplementary Figure 149. ^{19}F NMR (376 MHz, $CDCl_3$) spectrum of **51**

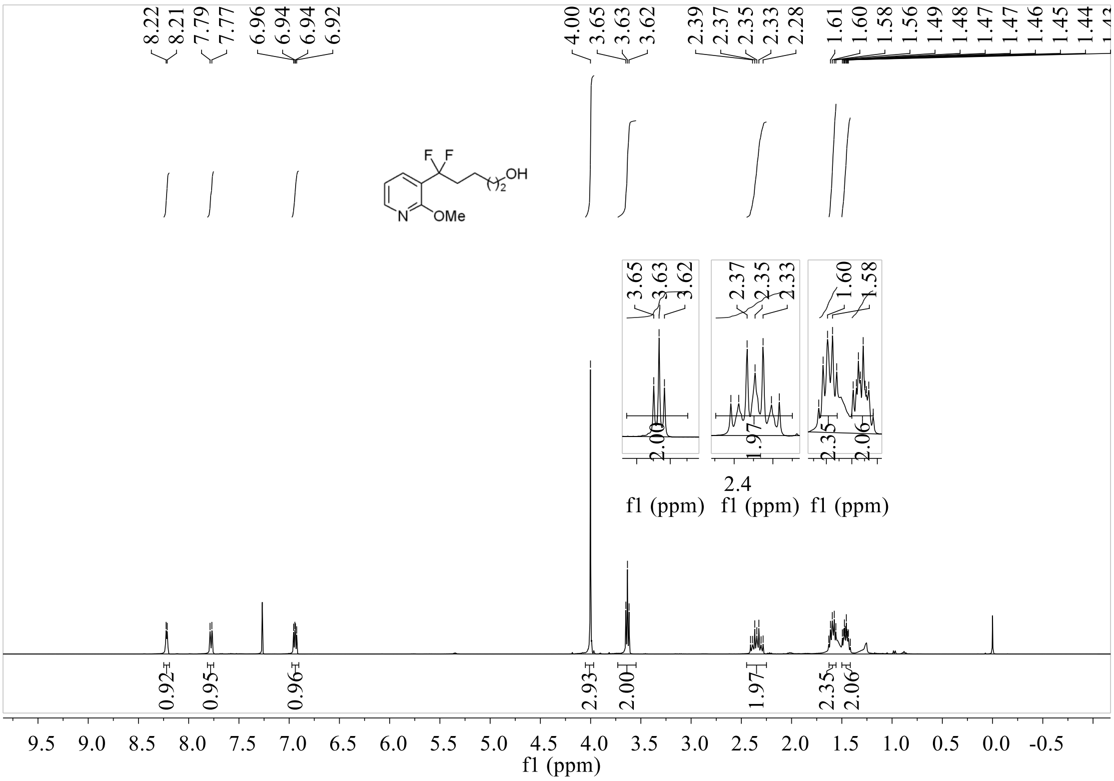

Supplementary Figure 150. ^{1}H NMR (400 MHz, $CDCl_3$) spectrum of **52**

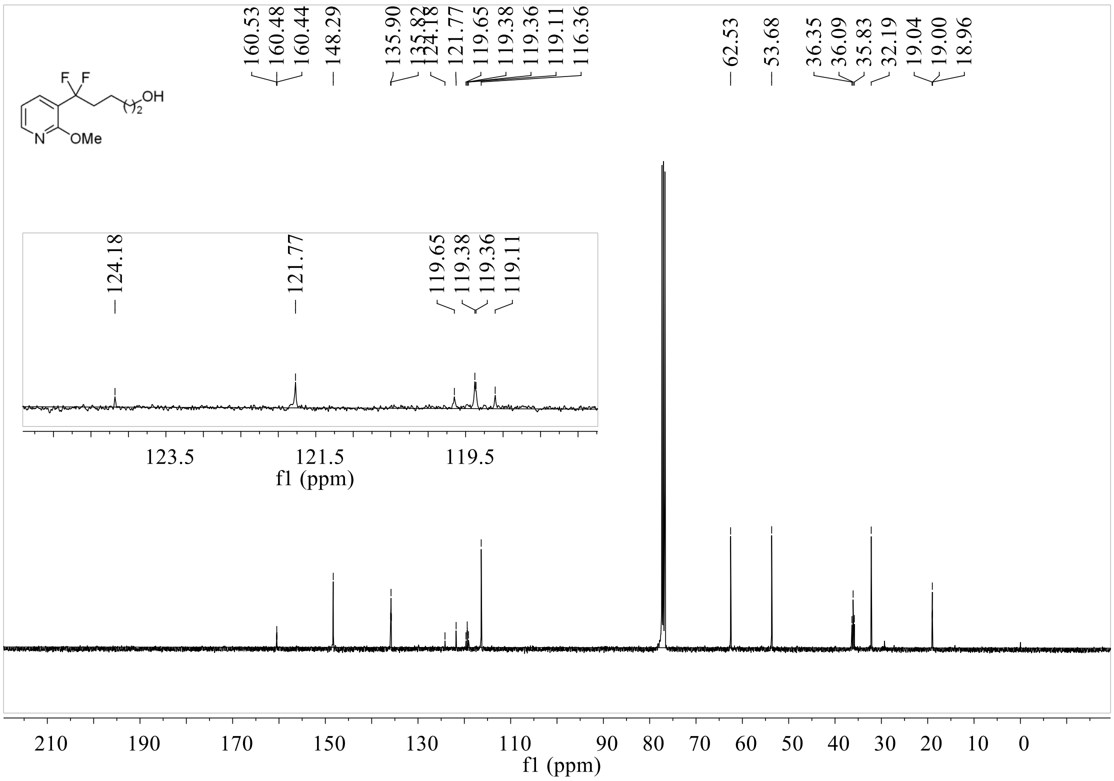

Supplementary Figure 151. ^{13}C NMR (101 MHz, $CDCl_3$) spectrum of **52**

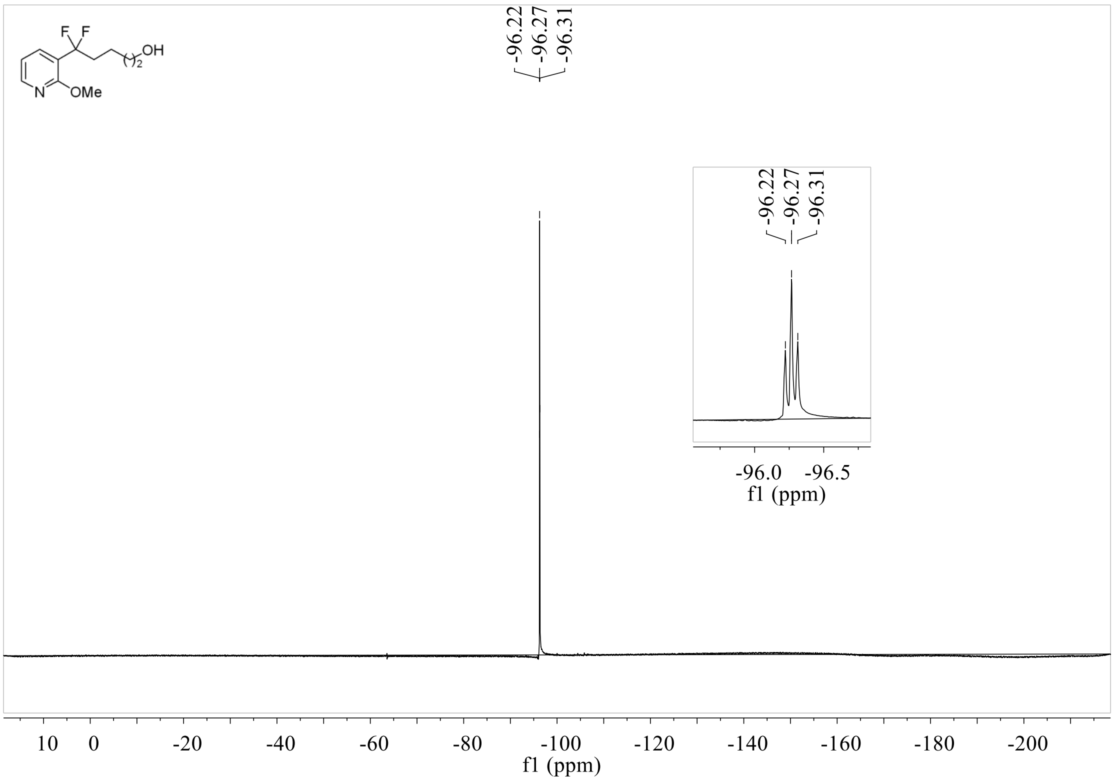

Supplementary Figure 152. ^{19}F NMR (376 MHz, $CDCl_3$) spectrum of **52**

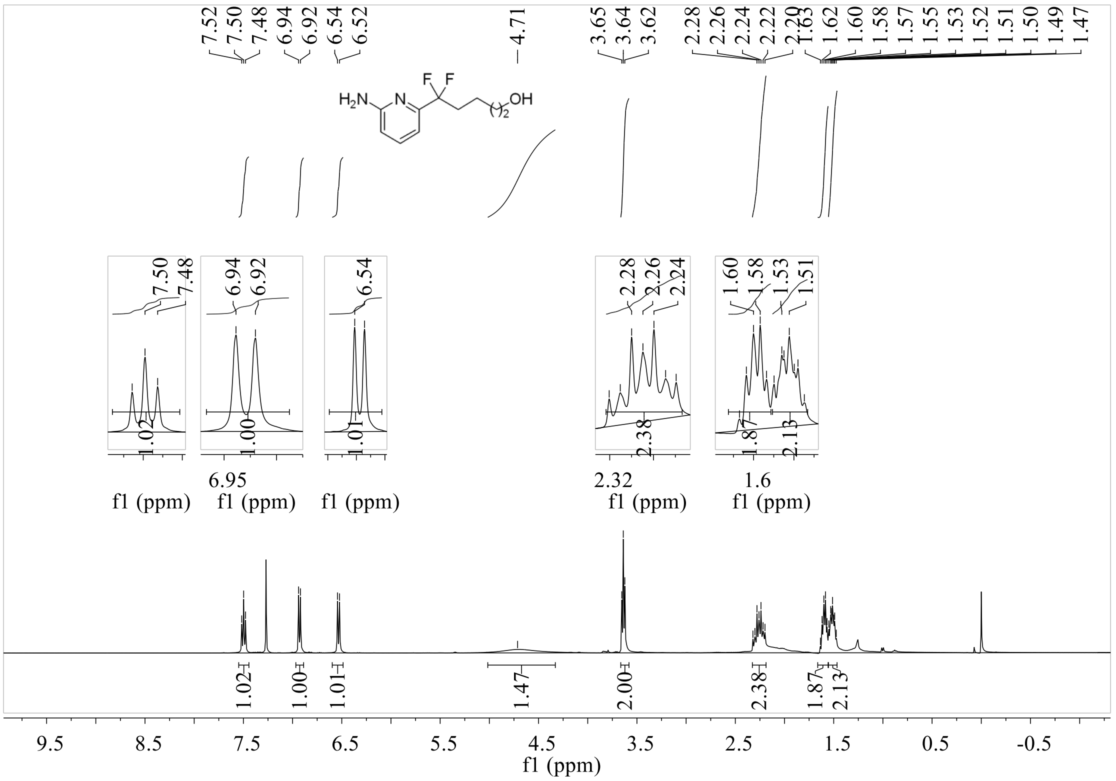

Supplementary Figure 153. ^{1}H NMR (400 MHz, $CDCl_3$) spectrum of **53**

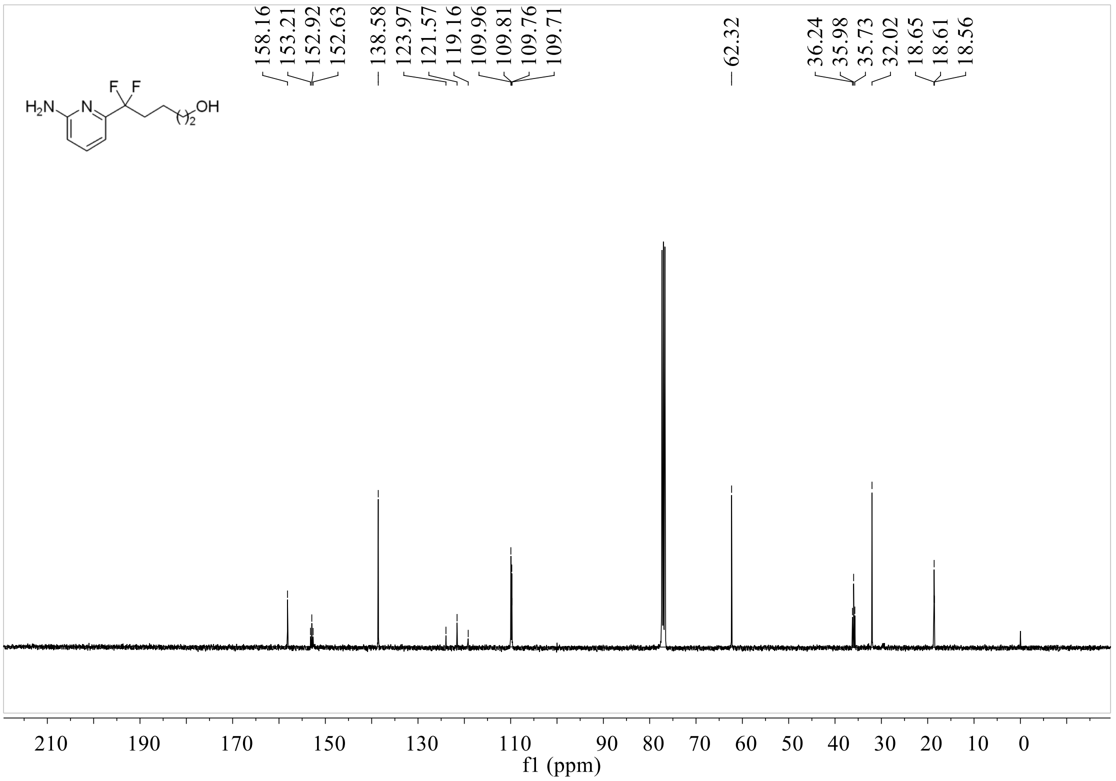

Supplementary Figure 154. ^{13}C NMR (101 MHz, $CDCl_3$) spectrum of **53**

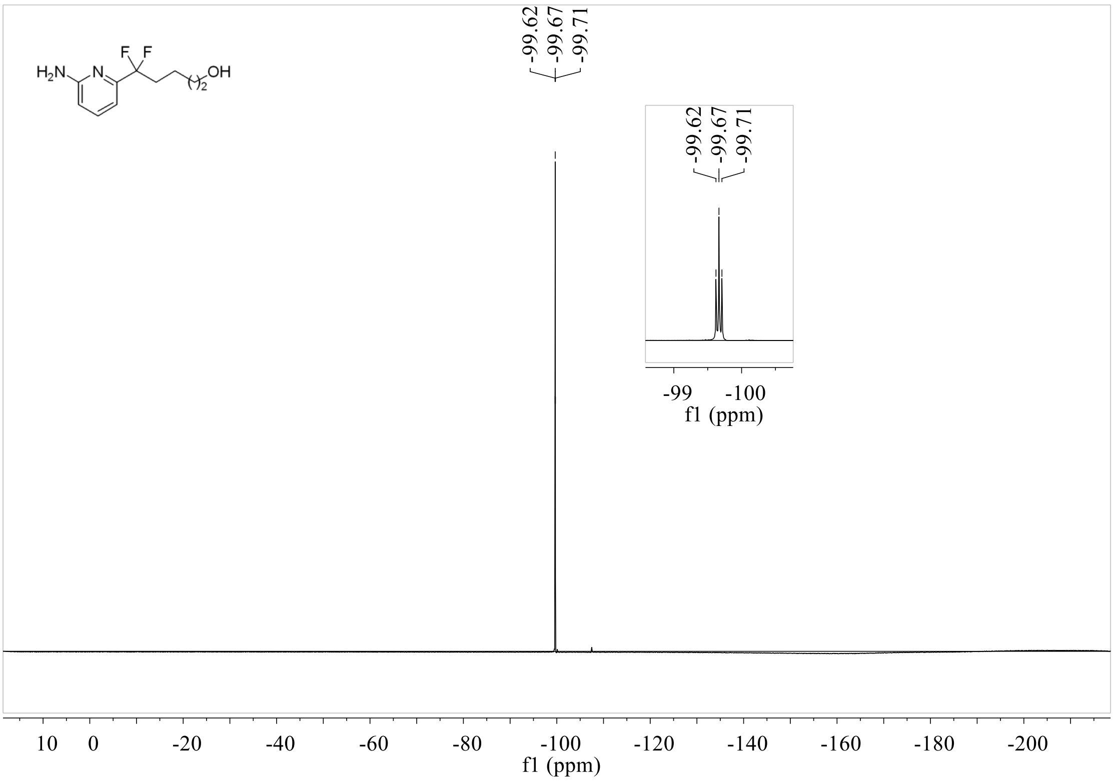

Supplementary Figure 155. ^{19}F NMR (376 MHz, $CDCl_3$) spectrum of **53**

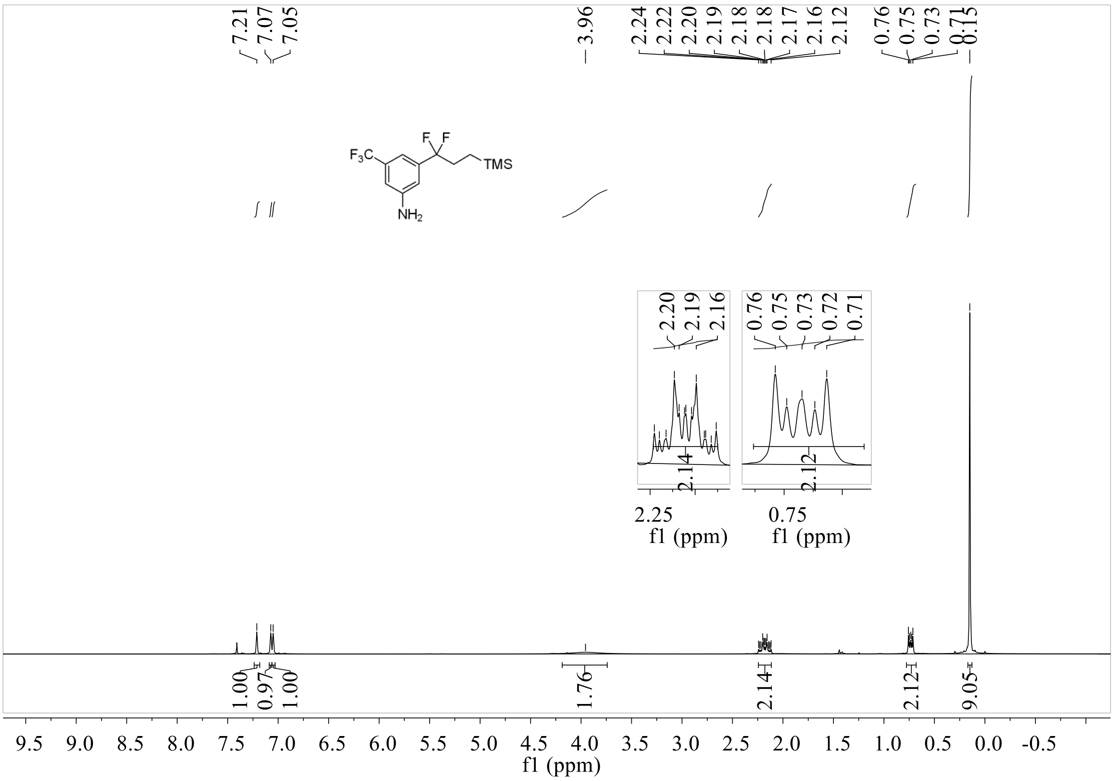

Supplementary Figure 156. ^{1}H NMR (400 MHz, $CDCl_3$) spectrum of **54**

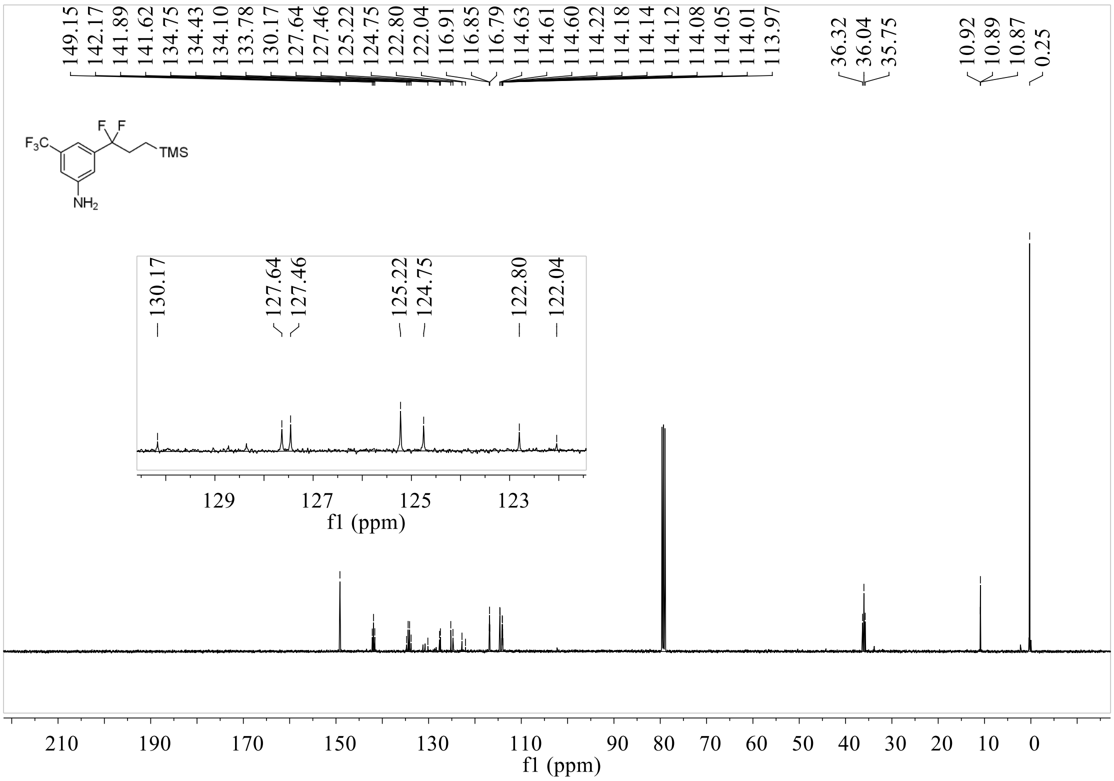

Supplementary Figure 157. ^{13}C NMR (101 MHz, $CDCl_3$) spectrum of **54**

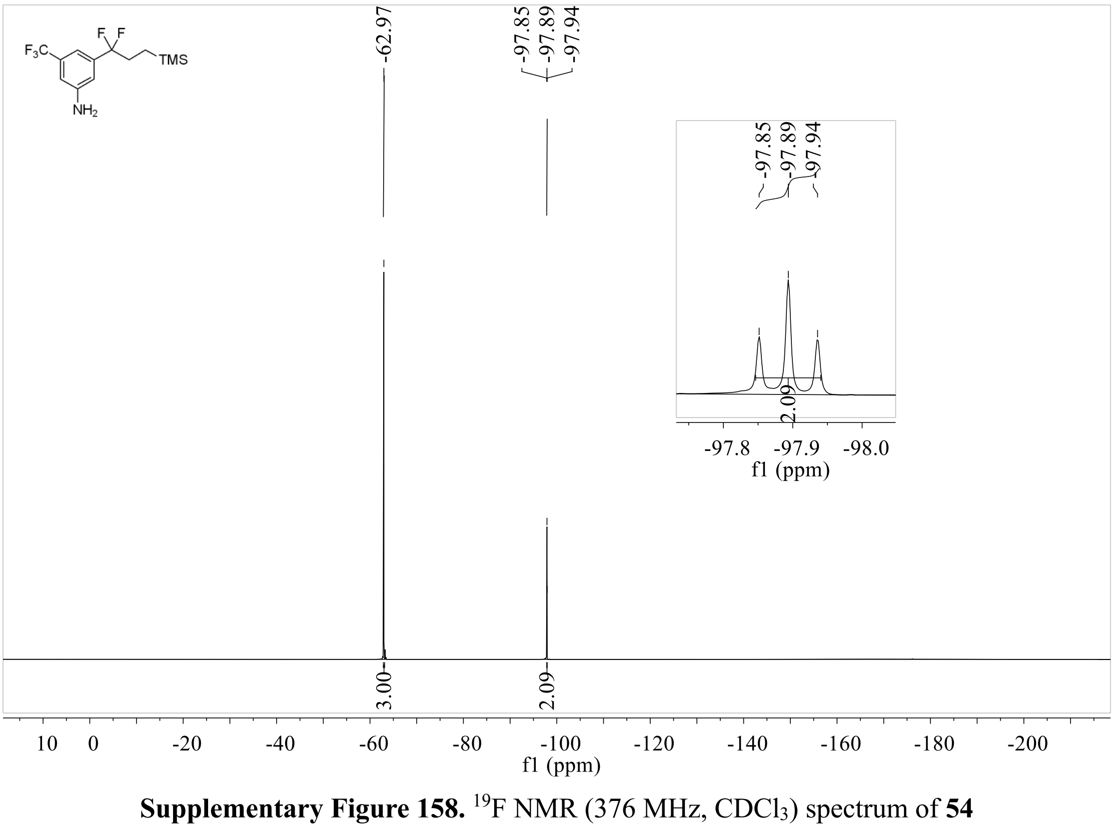

Supplementary Figure 158. ^{19}F NMR (376 MHz, $CDCl_3$) spectrum of **54**

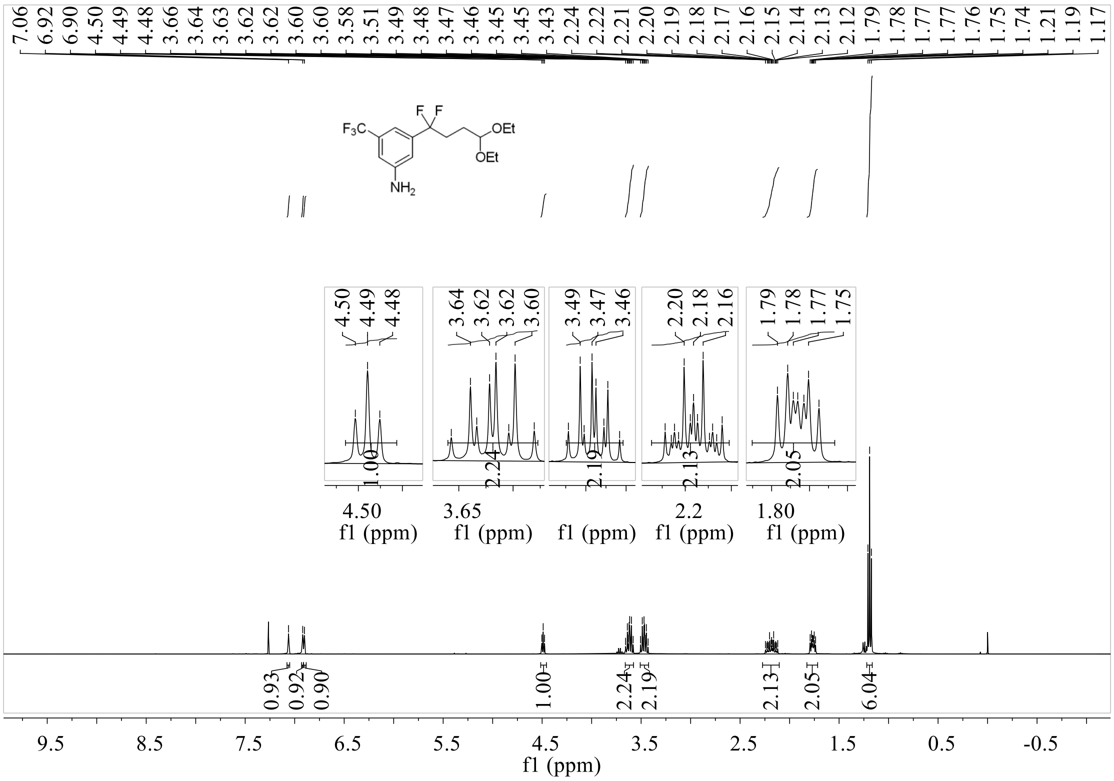

Supplementary Figure 159. ^{1}H NMR (400 MHz, $CDCl_3$) spectrum of **55**

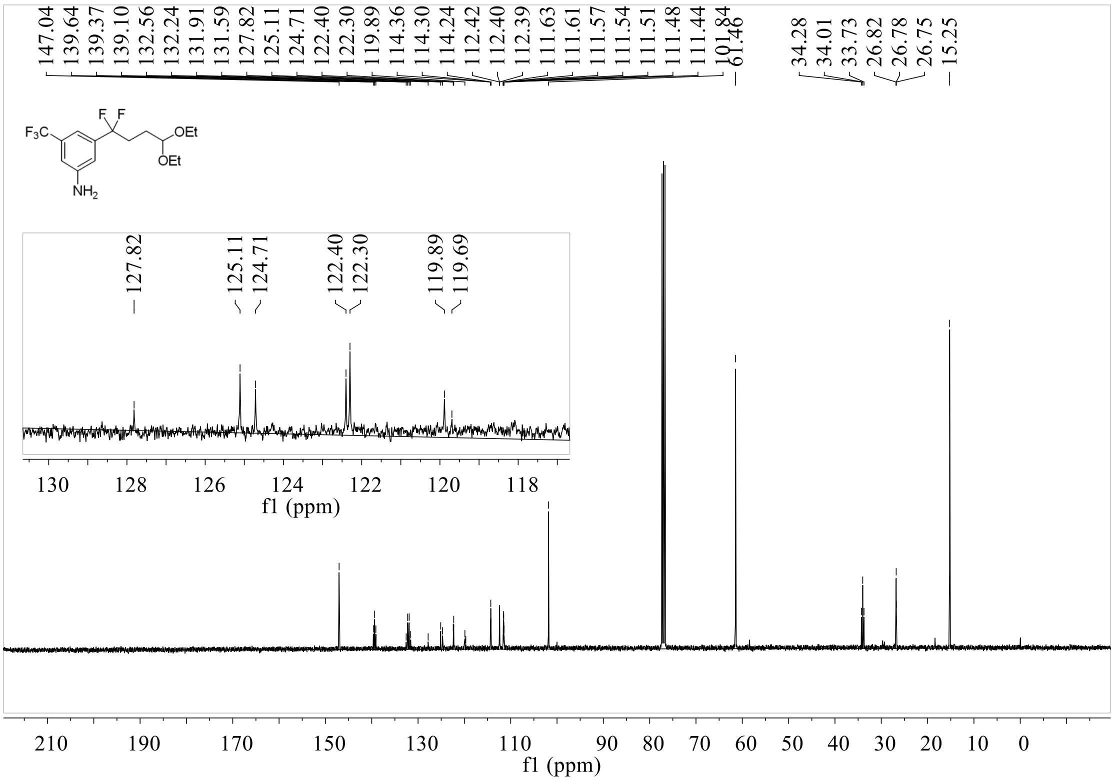

Supplementary Figure 160. ^{13}C NMR (101 MHz, $CDCl_3$) spectrum of **55**

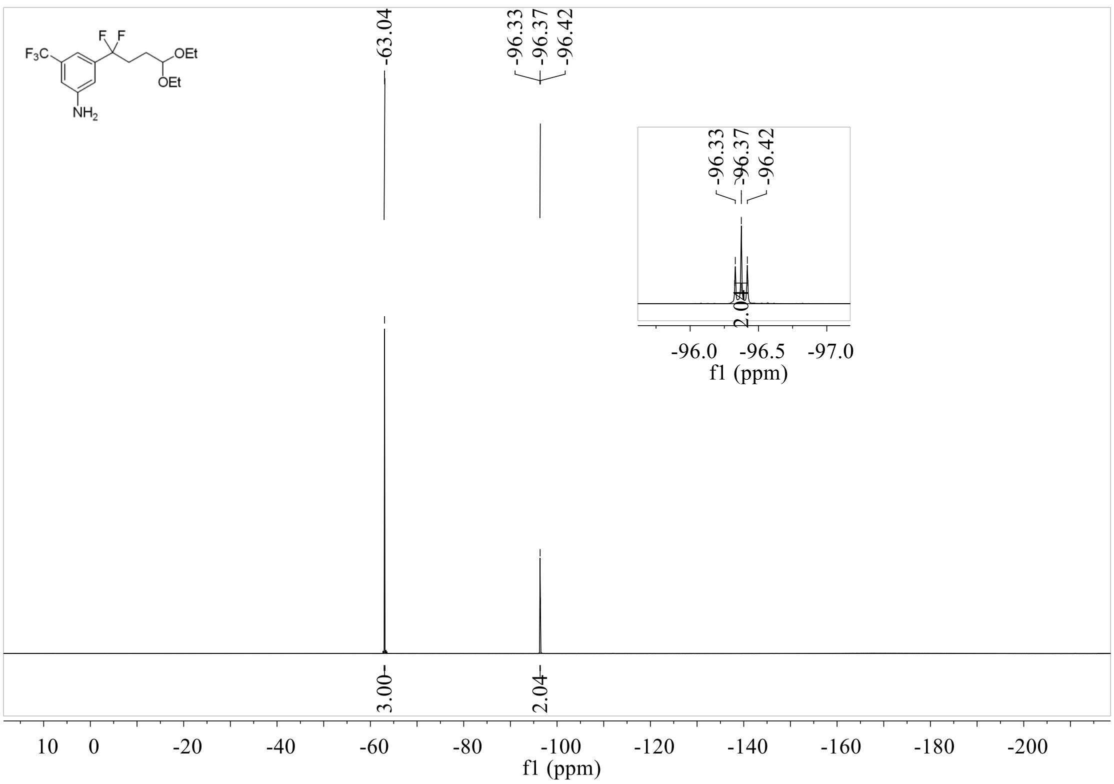

Supplementary Figure 161. ^{19}F NMR (376 MHz, $CDCl_3$) spectrum of **55**

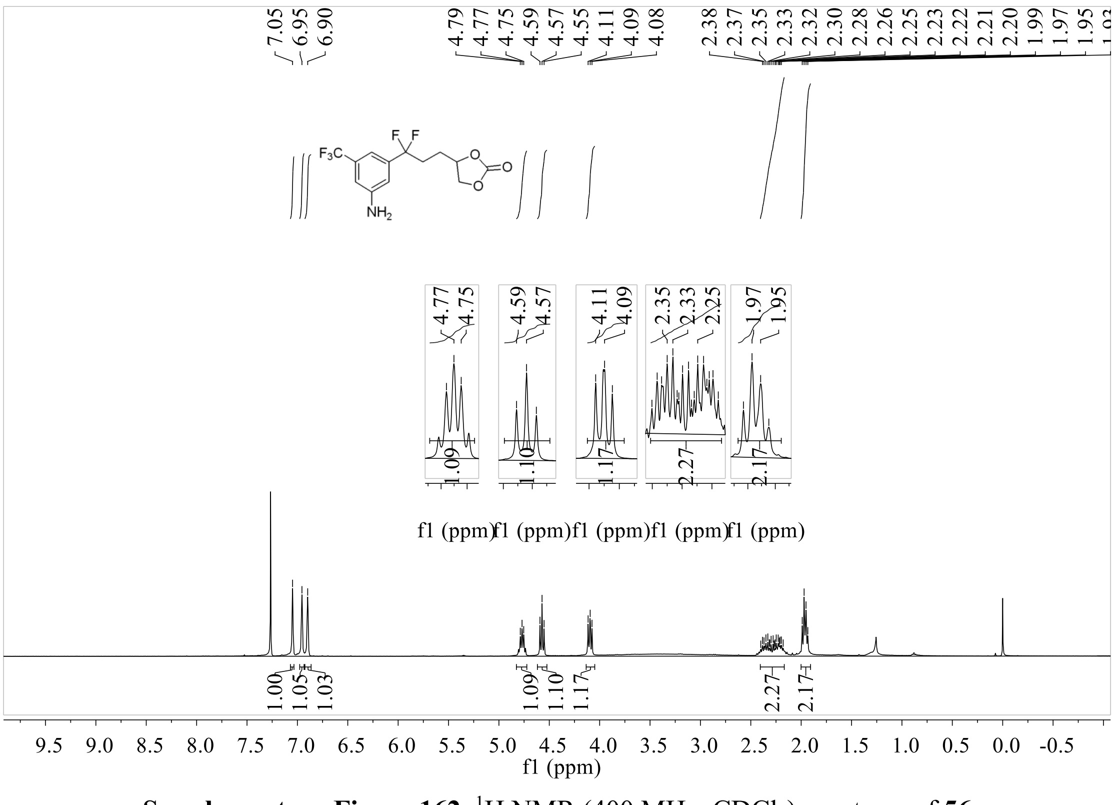

Supplementary Figure 162. ^{1}H NMR (400 MHz, $CDCl_3$) spectrum of **56**

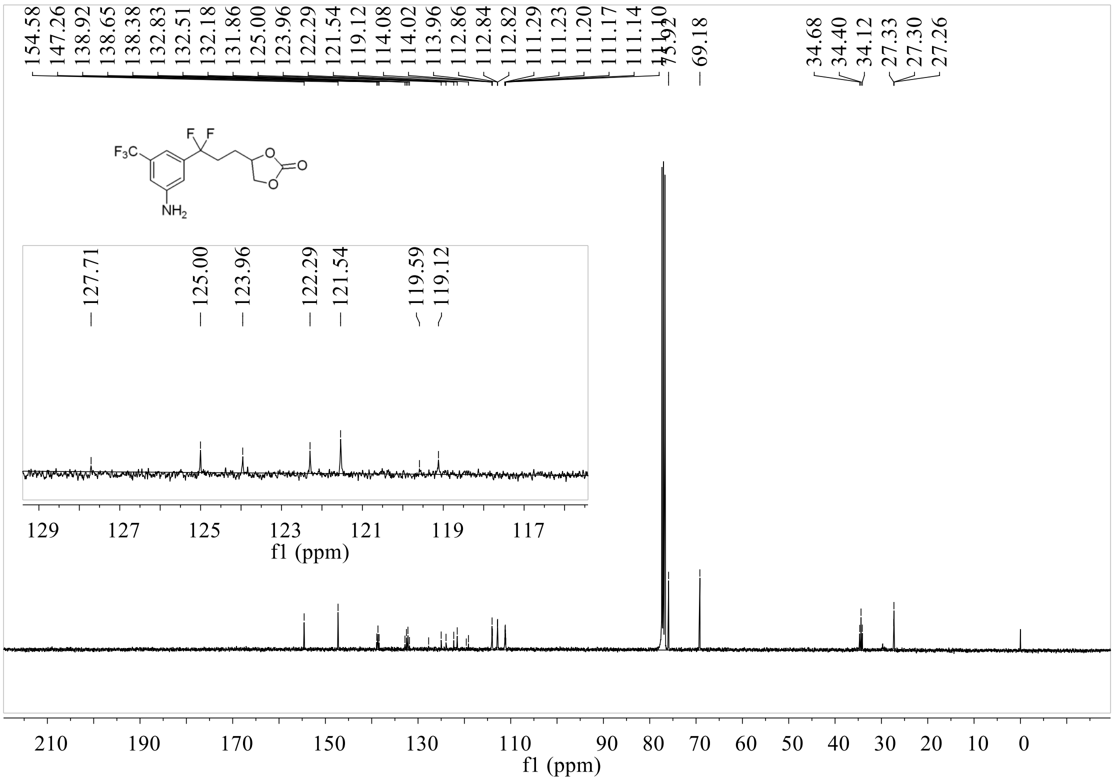

Supplementary Figure 163. ^{13}C NMR (101 MHz, $CDCl_3$) spectrum of **56**

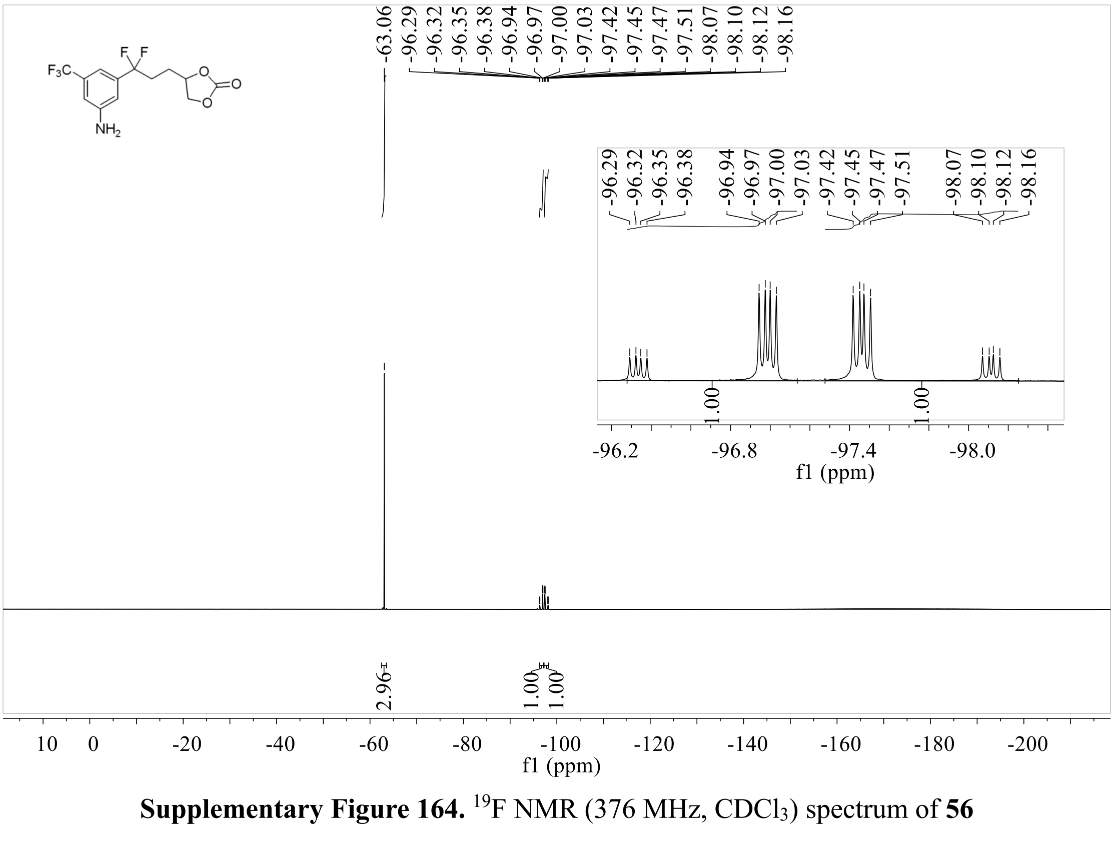

Supplementary Figure 164. ^{19}F NMR (376 MHz, $CDCl_3$) spectrum of **56**

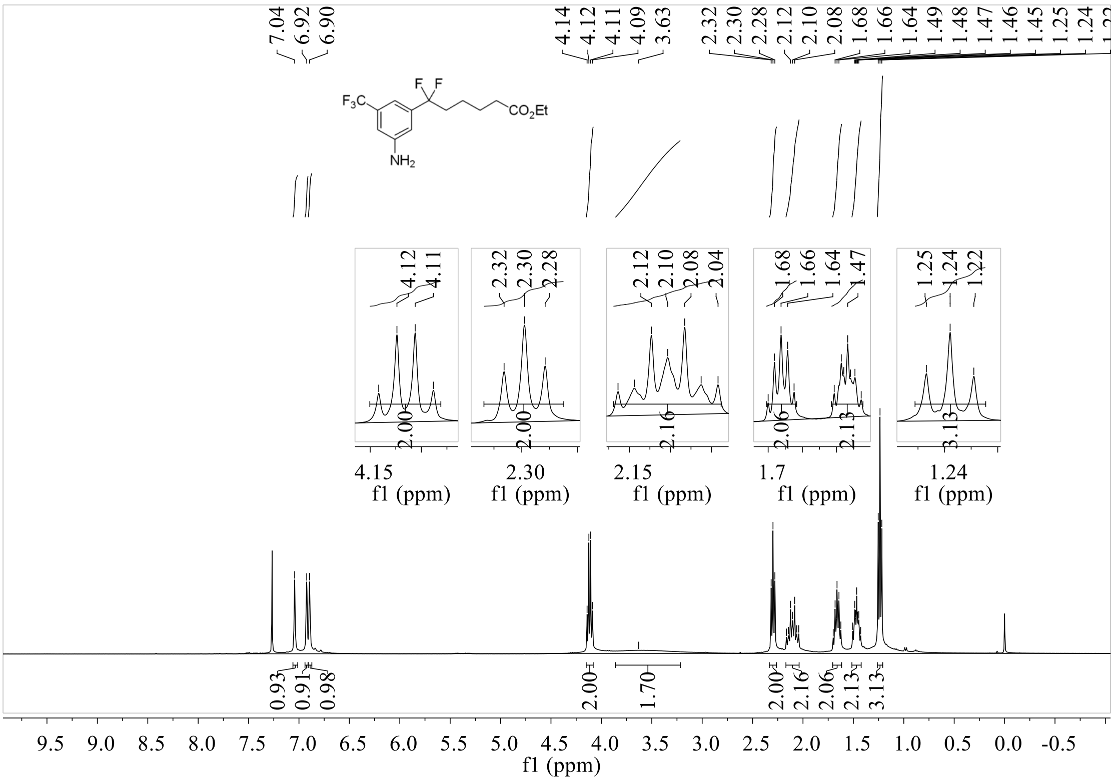

Supplementary Figure 165. ^{1}H NMR (400 MHz, $CDCl_3$) spectrum of **57**

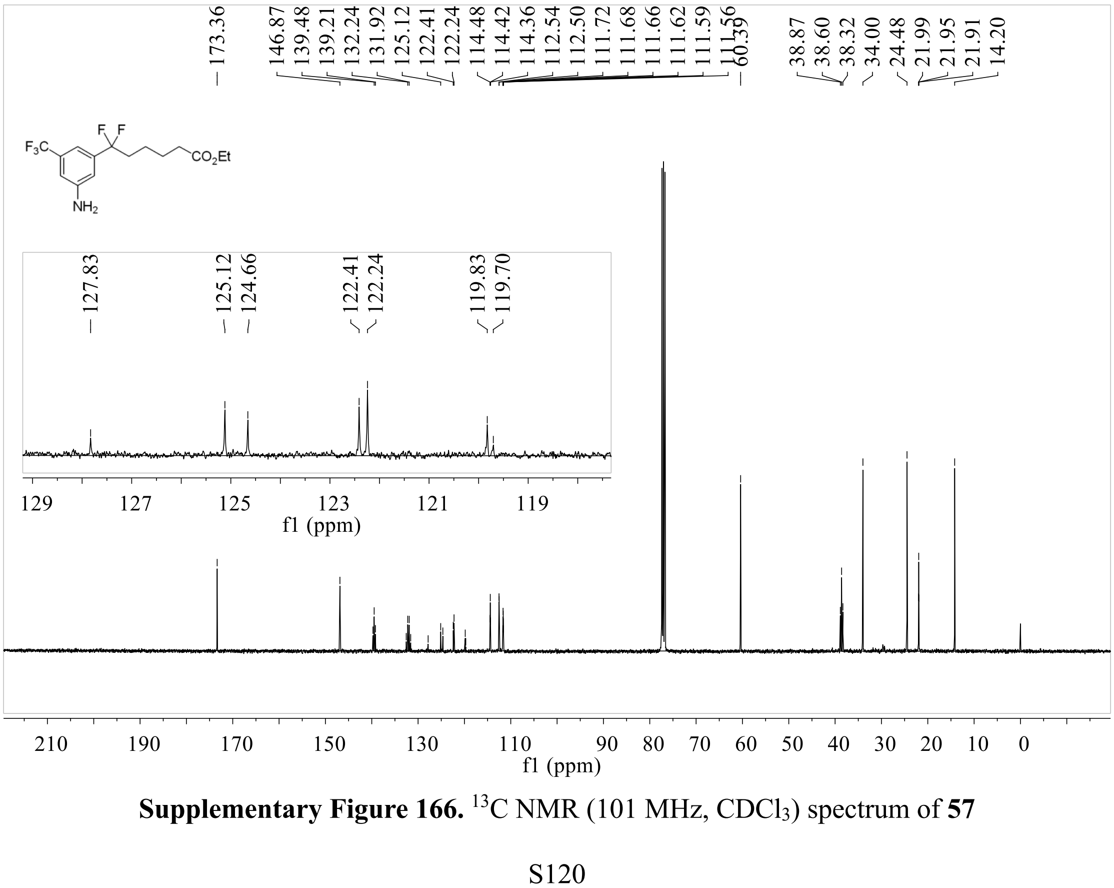

Supplementary Figure 166. ^{13}C NMR (101 MHz, $CDCl_3$) spectrum of **57**

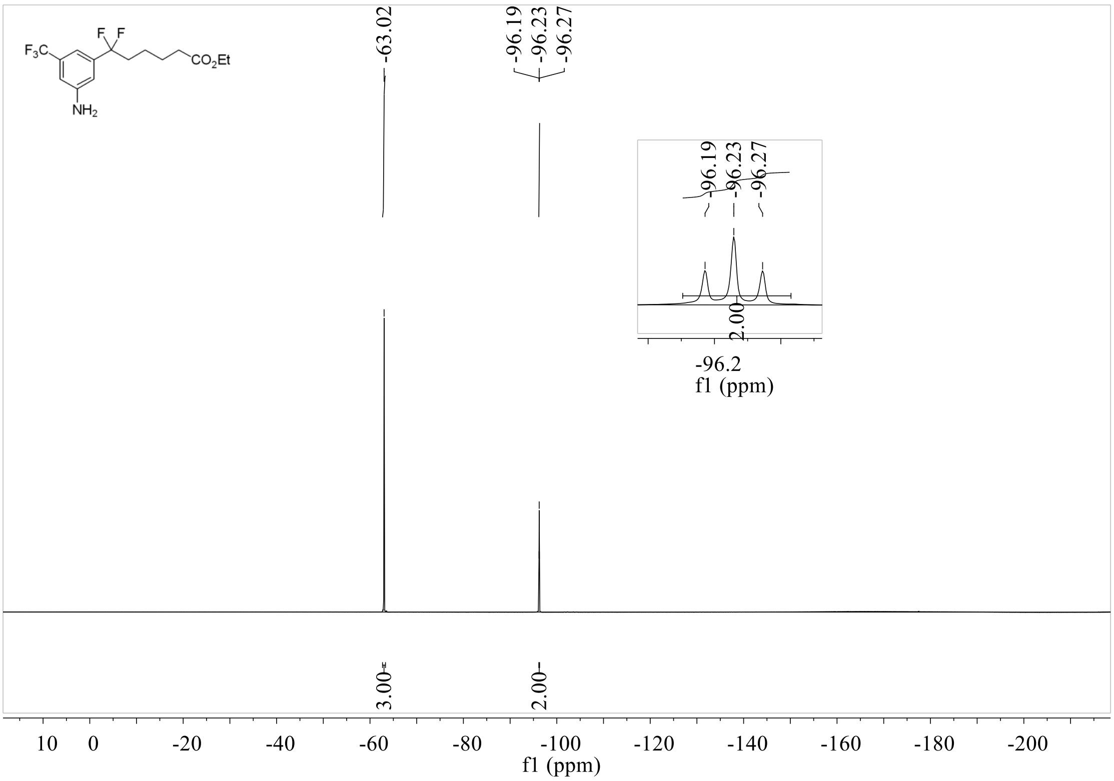

Supplementary Figure 167. ^{19}F NMR (376 MHz, $CDCl_3$) spectrum of **57**

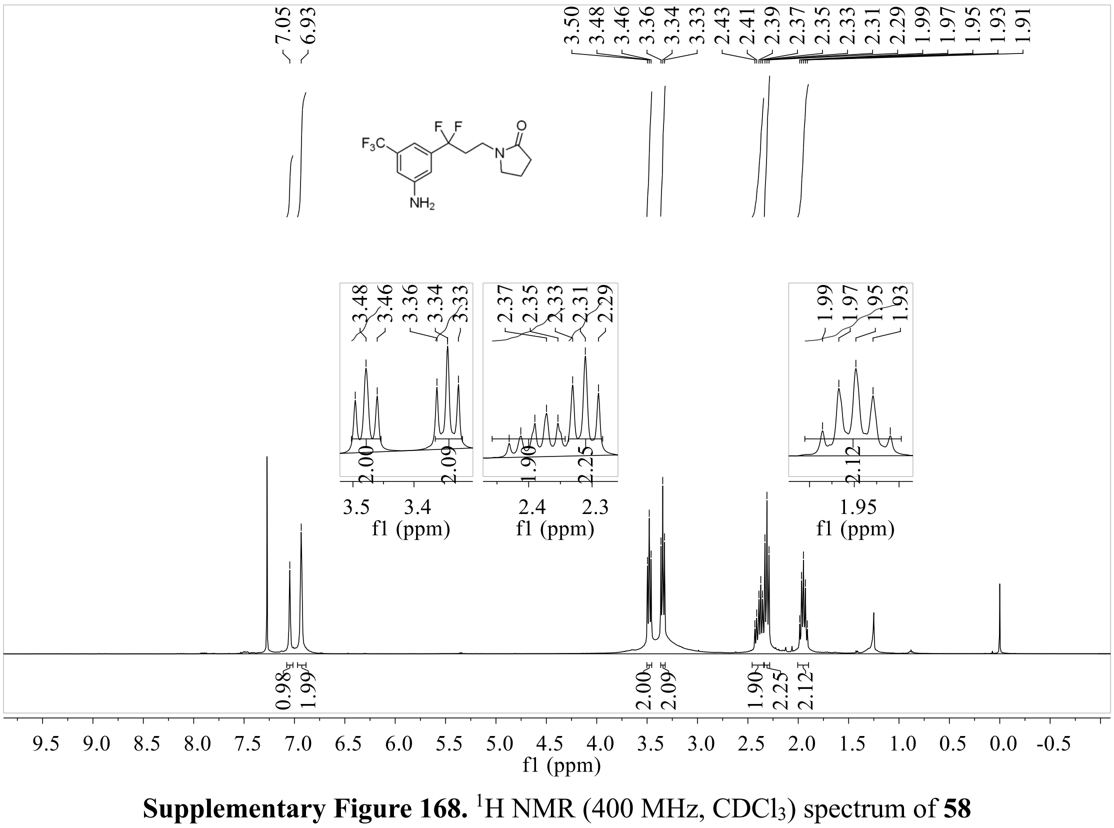

Supplementary Figure 168. ^{1}H NMR (400 MHz, $CDCl_3$) spectrum of **58**

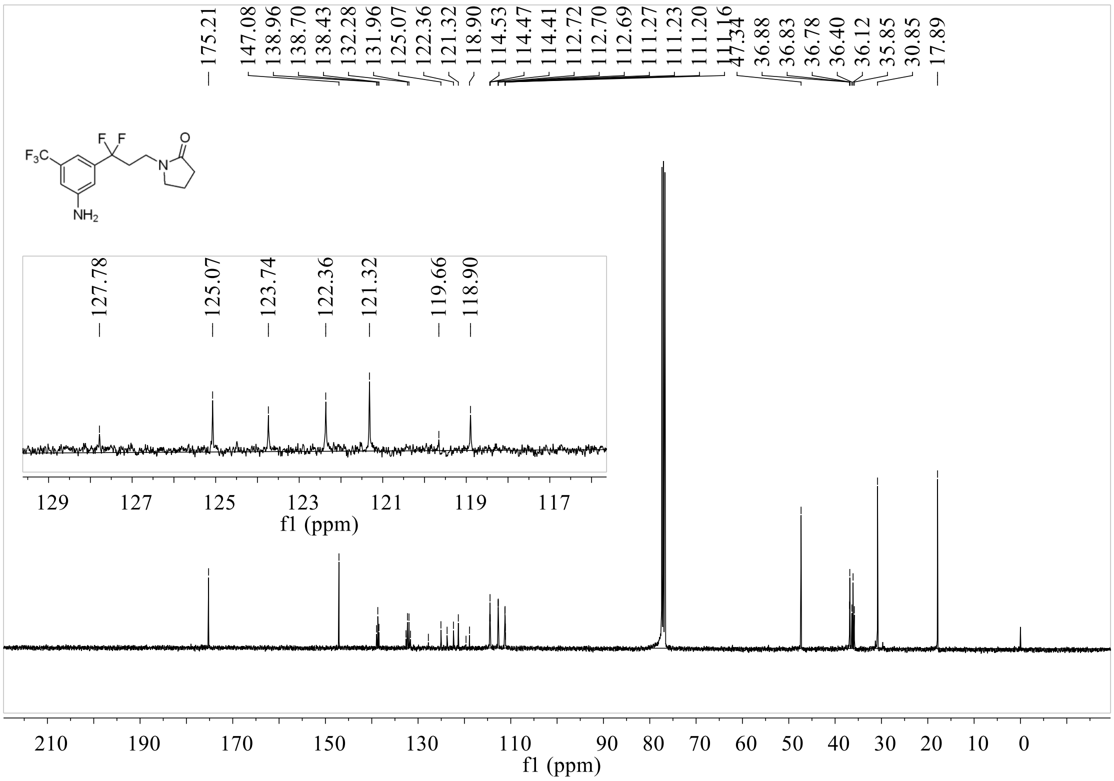

Supplementary Figure 169. ^{13}C NMR (101 MHz, $CDCl_3$) spectrum of **58**

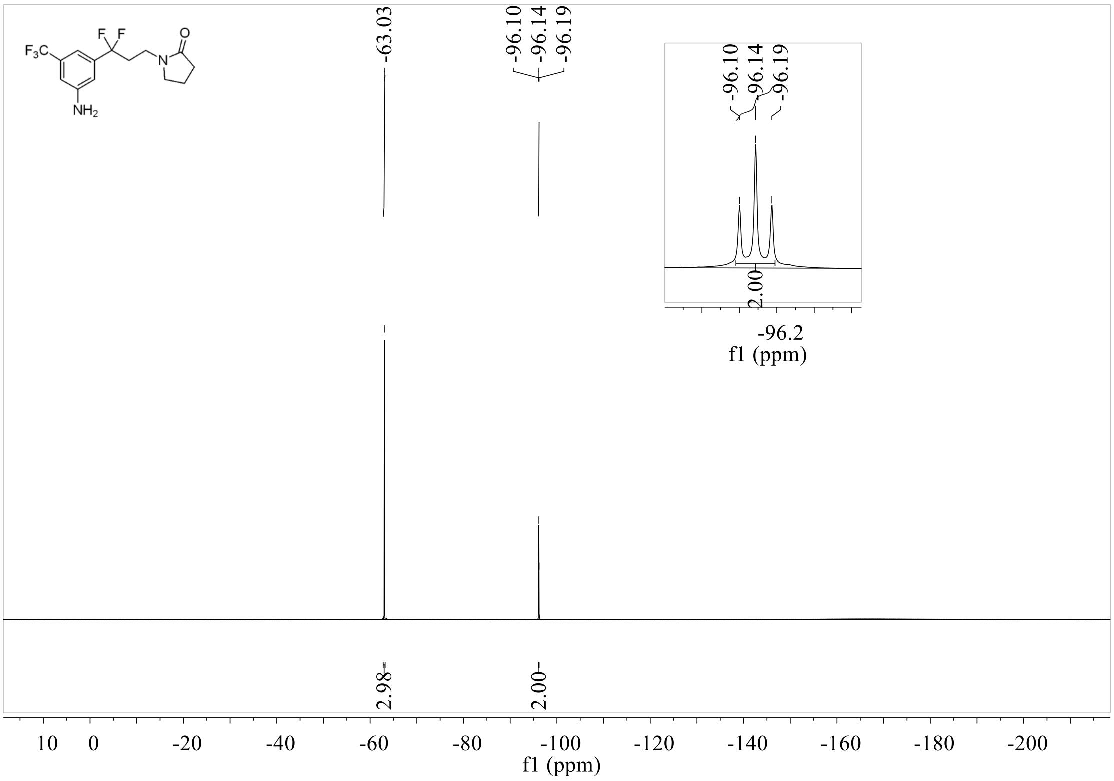

Supplementary Figure 170. ^{19}F NMR (376 MHz, $CDCl_3$) spectrum of **58**

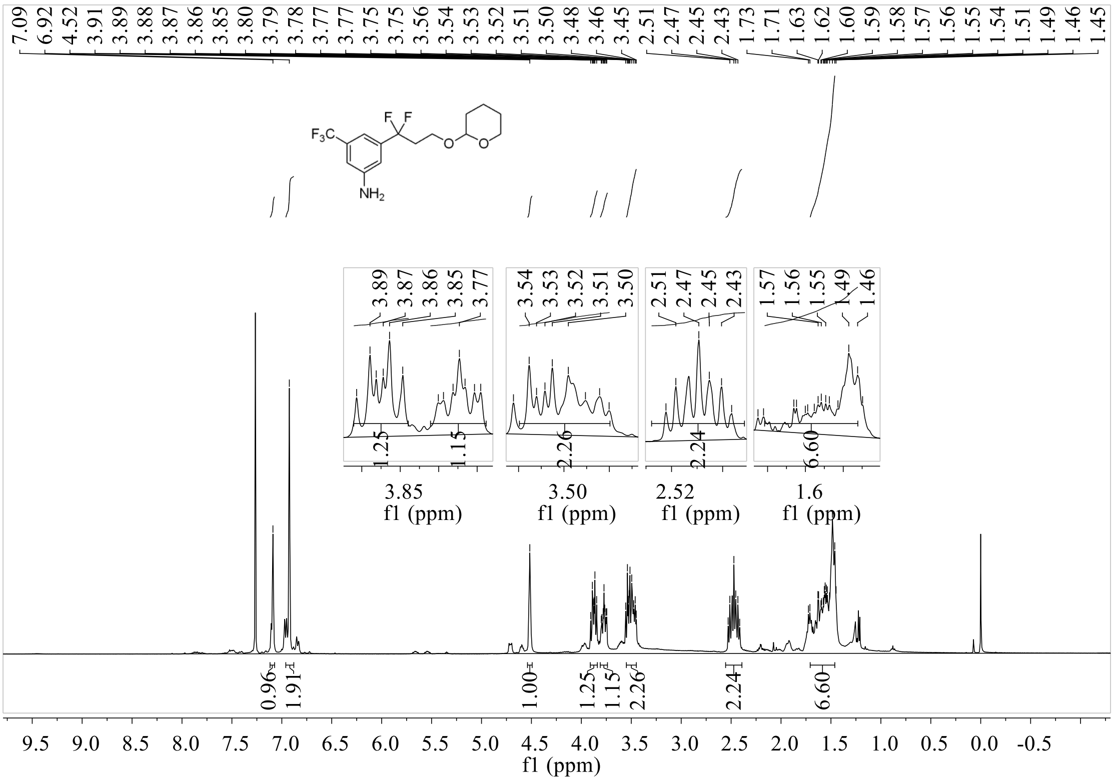

Supplementary Figure 171. ^{1}H NMR (400 MHz, $CDCl_3$) spectrum of **59**

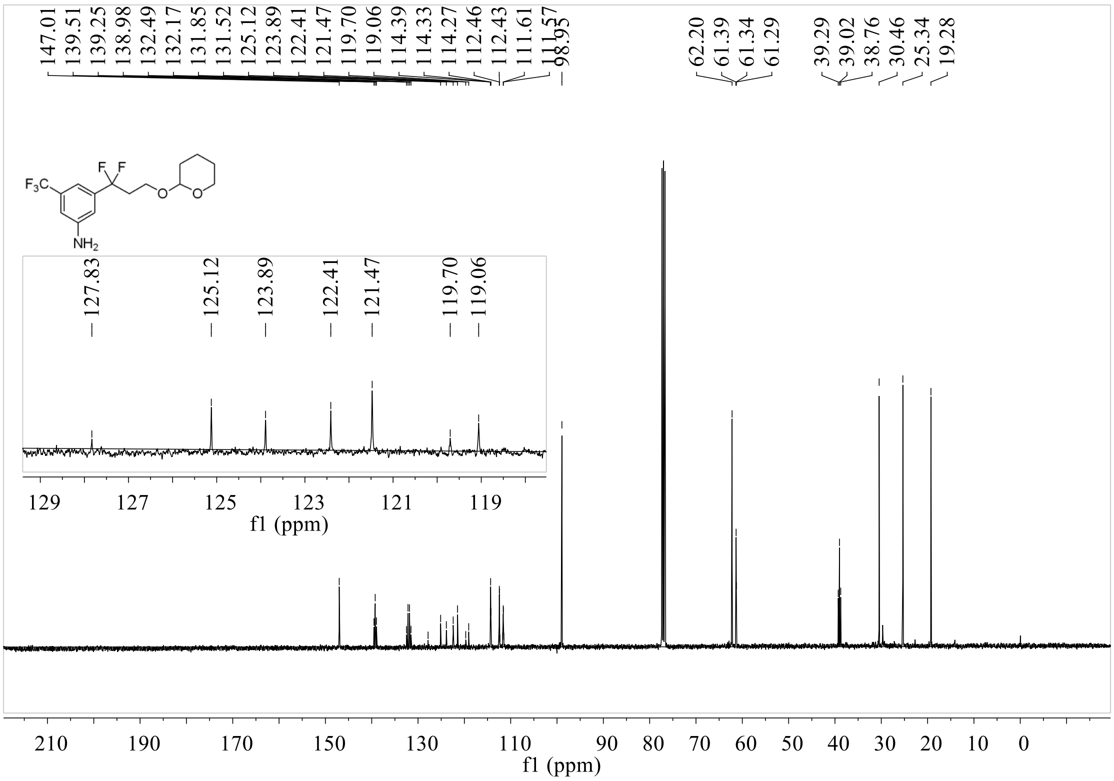

Supplementary Figure 172. ^{13}C NMR (101 MHz, $CDCl_3$) spectrum of **59**

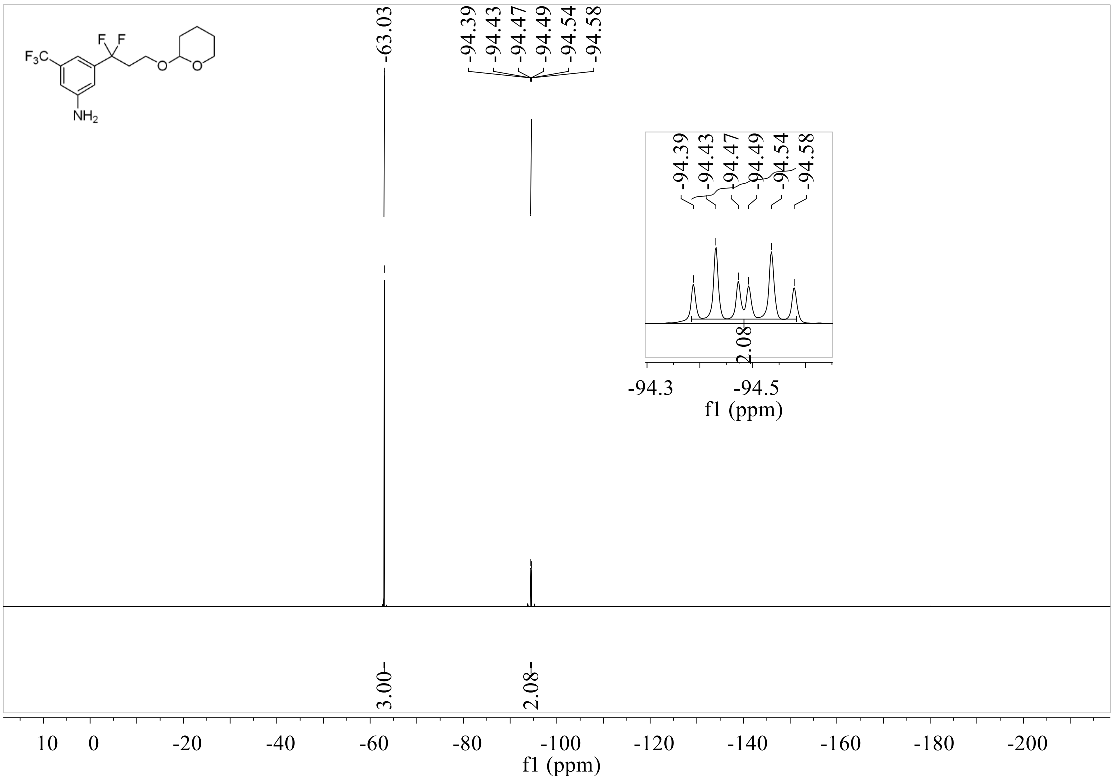

Supplementary Figure 173. ^{19}F NMR (376 MHz, $CDCl_3$) spectrum of **59**

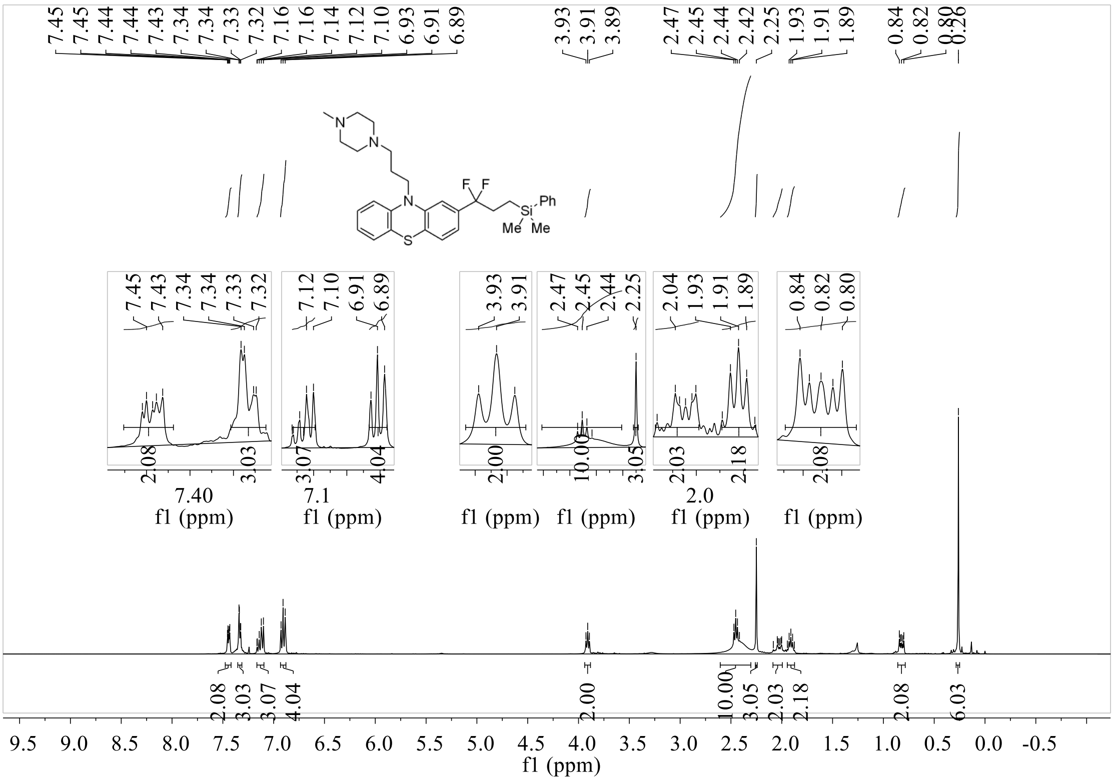

Supplementary Figure 174. ^{1}H NMR (400 MHz, $CDCl_3$) spectrum of **60**

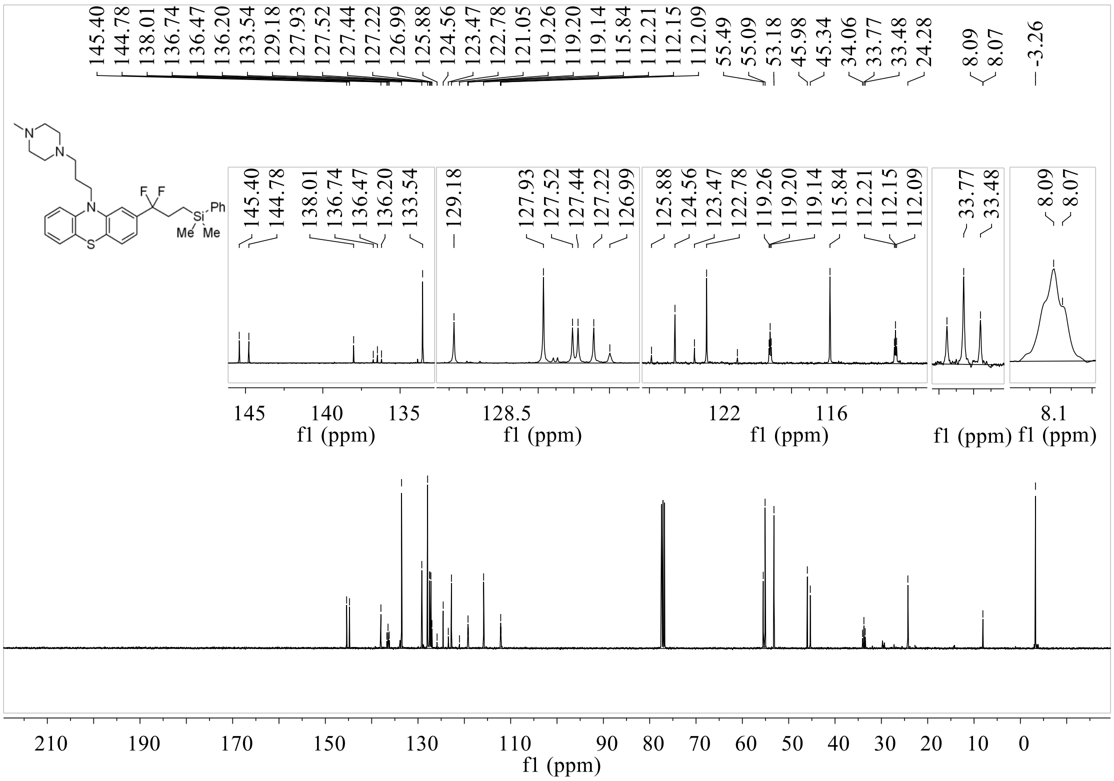

Supplementary Figure 175. ^{13}C NMR (101 MHz, $CDCl_3$) spectrum of **60**

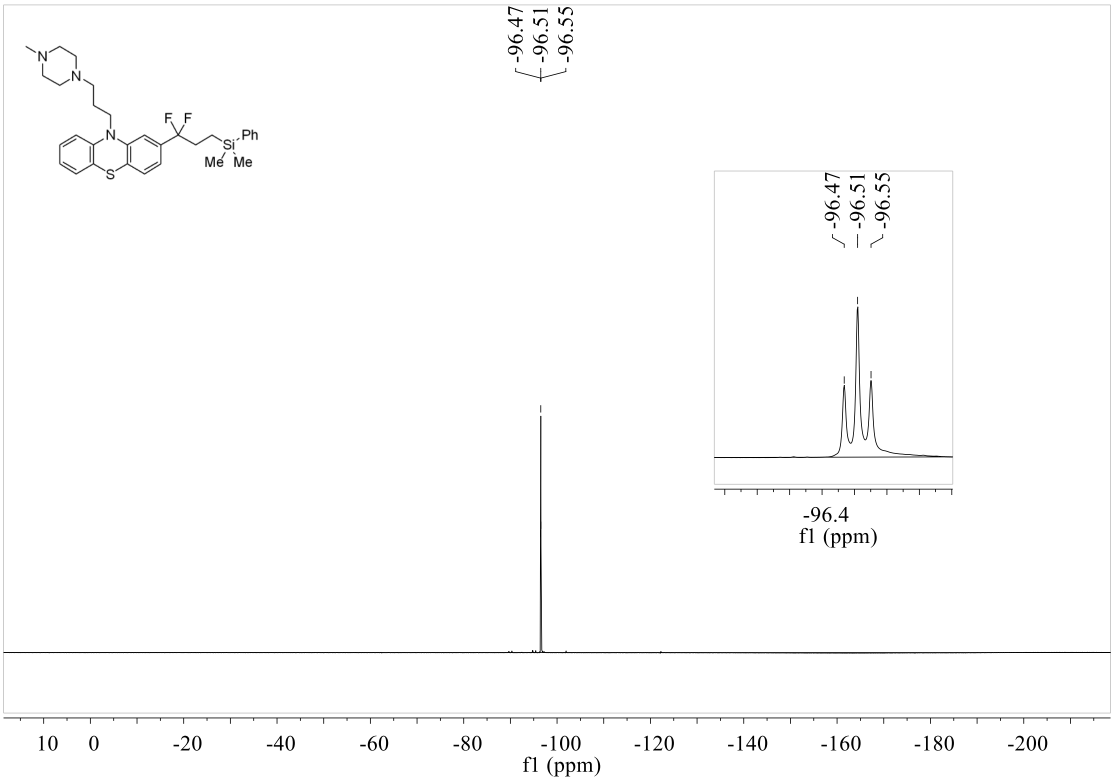

Supplementary Figure 176. ^{19}F NMR (376 MHz, $CDCl_3$) spectrum of **60**

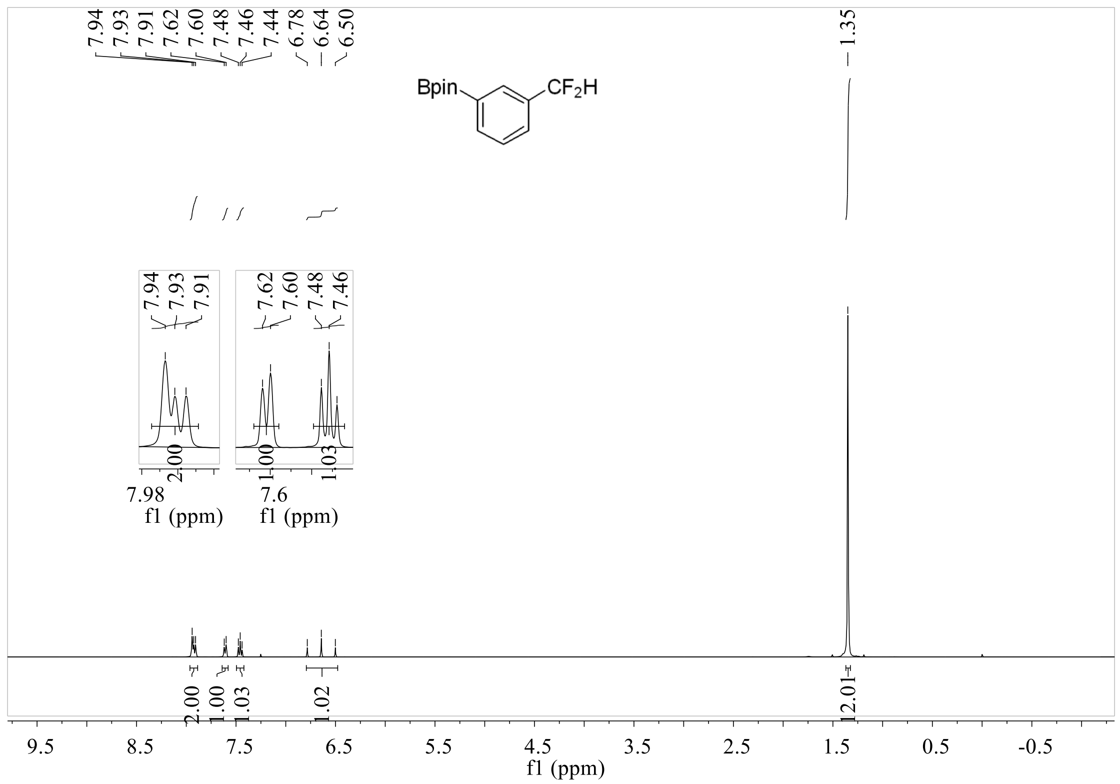

Supplementary Figure 177. ^{1}H NMR (400 MHz, $CDCl_3$) spectrum of **61**

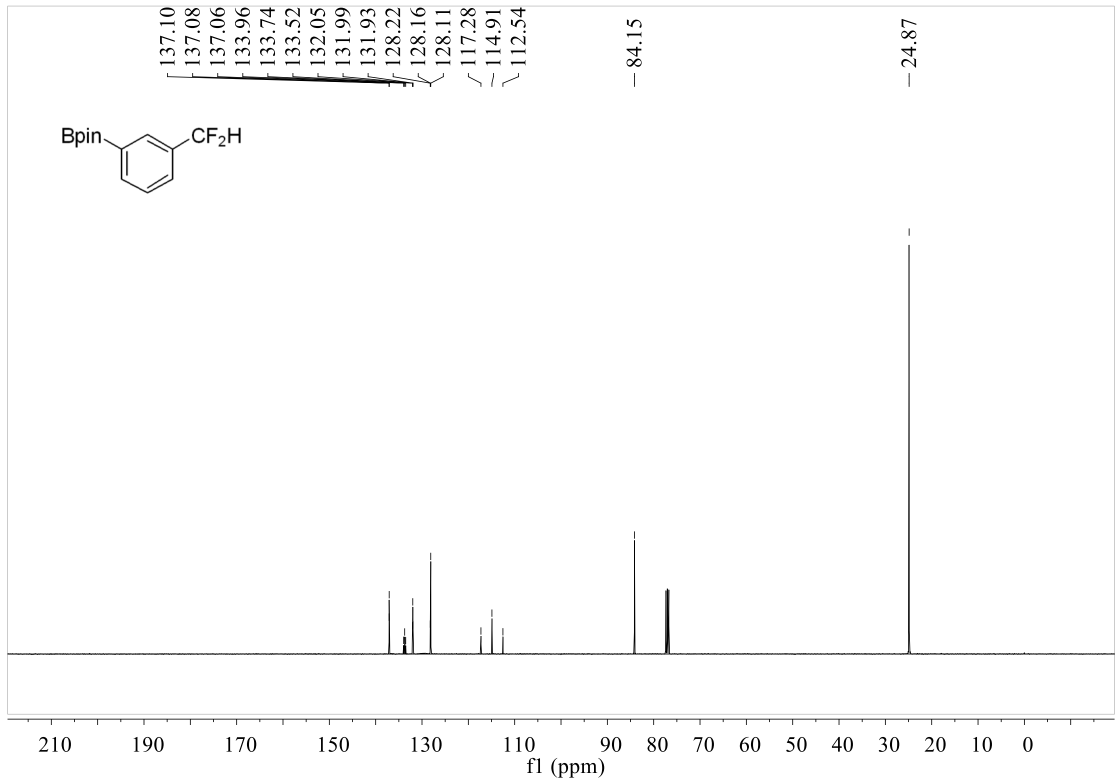

Supplementary Figure 178. ^{13}C NMR (101 MHz, $CDCl_3$) spectrum of **61**

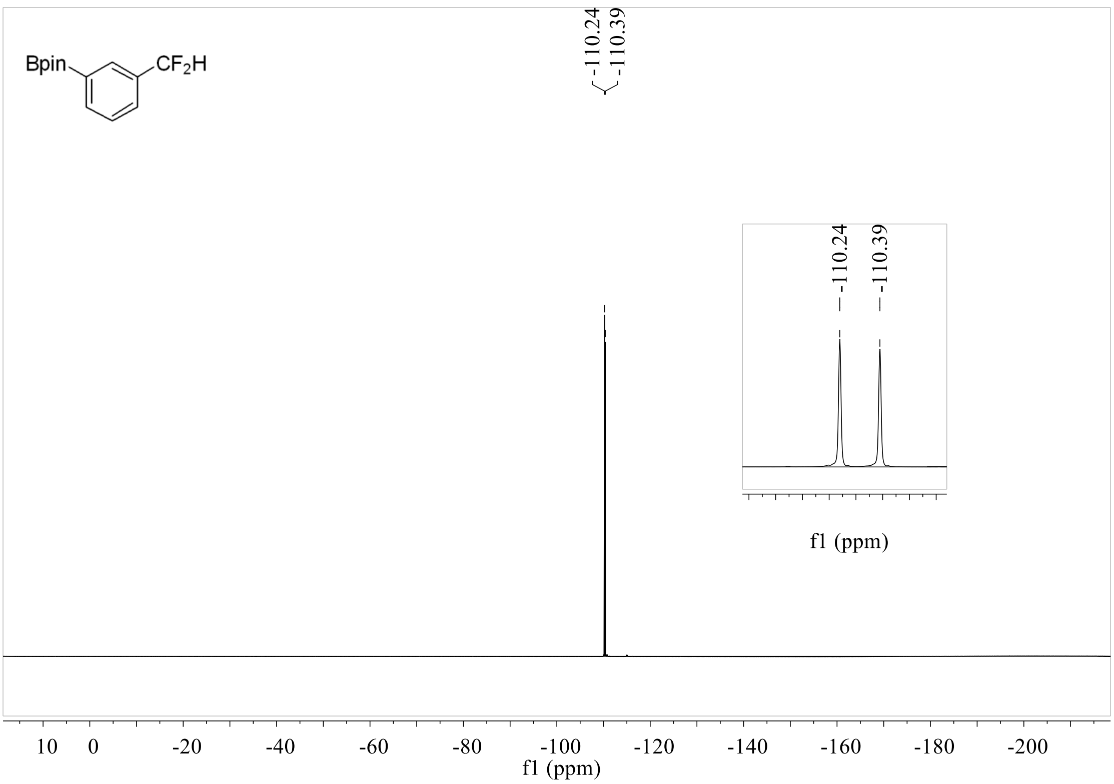

Supplementary Figure 179. ^{19}F NMR (376 MHz, $CDCl_3$) spectrum of **61**

3. Supplementary References

1. Kovacs, M. S., Hein, P., Sattarzadeh, S., Patrick, B. O., Emge, T. J. & Orvig, C. Complexes of phosphine–phenolate ligands with the $[Re{=}O]^{3+}$ and $[Re(HNNC_5H_4N)(NNC_5H_4N)]^{2+}$ cores. *J. Chem. Soc. Dalton Trans.* **2001**, 3015–3024 (2001).
2. Bakewell, C., Cao, T.-P.-A., Le Goff, X. F., Long, N. J., Auffrant, A. & Williams, C. K. Yttrium phosphasalen initiators for *rac*-lactide polymerization. *Organometallics* **32**, 1475−1483 (2013).
3. Fliedel, C., Rosa, V., Alves, F. M., Martins, A. M., Avilés, T. & Dagorne, S. *P,O*-Phosphinophenolate zinc(II) species: synthesis, structure and use in the ring-opening polymerization (ROP) of lactide, *ε*-caprolactone and trimethylene carbonate. *Dalton Trans.* **44**, 12376–12387 (2015).
4. Fischer, M., Schaper, R., Jaugstetter, M., Schmidtmann, M. & Beckhaus, R. Electrophilic d^0 cations of group 4 Metals (M = Ti, Zr, Hf) derived from monopentafulvene complexes: direct formation of tridentate *Cp,O,P*-ligands. *Organometallics* **37**, 1192−1205 (2108).
5. García-Márquez, A., Frontera, A., Roisnel, T. & Gramage-Doria, R. Ultrashort $H^{\delta+}\cdots H^{\delta-}$ intermolecular distance in a supramolecular system in the solid state. *Chem. Commun.* **57**, 7112–7115 (2021).
6. Heinicke, J., Köhler, M., Peulecke, N., He, M., Kindermann, M. K., Keim, W. & Fink, G. 2-Phosphanylphenolate nickel catalysts for the polymerization of ethylene. *Chem. Eur. J.* **9**, 6093–6107 (2003).
7. Bakewell, C., Cao, T.-P-A., Long, N., Le Goff, X. F., Auffrant, A. & Williams, C. K. Yttrium phosphasalen initiators for *rac*-lactide polymerization: excellent rates and high iso-selectivities. *J. Am. Chem. Soc.* **134**, 20577−20580 (2012).
8. Yu, Y.-J., Zhang, F.-L., Peng, T.-Y., Wang, C.-L., Cheng, J., Chen, C., Houk, K. N. & Wang, Y.-F. Sequential C–F bond functionalizations of trifluoroacetamides and acetates via spin-center shifts. *Science* **371**, 1232–1240 (2021).
9. Tlahuext-Aca, A., Garza-Sanchez, R. A., Schäfer, M. & Glorius, F. Visible-light-mediated synthesis of ketones by the oxidative alkylation of styrenes. *Org. Lett.* **20**, 1546−1549 (2018).

10. Woźniak, Ł., Murphy, J. J. & Melchiorre, P. Photo-organocatalytic enantioselective perfluoroalkylation of β-ketoesters. *J. Am. Chem. Soc.* **137**, 5678–5681 (2015).
11. Discekici, E. H., Treat, N. J., Poelma, S. O., Mattson, K. M., Hudson, Z. M., Luo, Y., Hawker, C. J. & de Alaniz, J. R. A highly reducing metal-free photoredox catalyst: design and application in radical dehalogenations. *Chem. Commun.* **51**, 11705−11708 (2015).
12. Silvi, M., Arceo, E., Jurberg, I. D., Cassani, C. & Melchiorre, P. Enantioselective organocatalytic alkylation of aldehydes and enals driven by the direct photoexcitation of enamines. *J. Am. Chem. Soc.* **137**, 6120−6123 (2015).
13. Buzzetti, L., Crisenza, G. E. M. & Melchiorre, P. Mechanistic studies in photocatalysis. *Angew. Chem. Int. Ed.* **58**, 3730–3747 (2019).
